# Stabilising Peptoid Helices Using Non‐Chiral Fluoroalkyl Monomers

**DOI:** 10.1002/anie.201804488

**Published:** 2018-06-27

**Authors:** Diana Gimenez, Juan A. Aguilar, Elizabeth H. C. Bromley, Steven L. Cobb

**Affiliations:** ^1^ Department of Chemistry Durham University South Road Durham DH1 3LE UK; ^2^ Department of Physics Durham University South Road Durham DH1 3LE UK

**Keywords:** fluorine, NMR spectroscopy, peptidomimetics, peptoids, secondary structure

## Abstract

Stability towards protease degradation combined with modular synthesis has made peptoids of considerable interest in the fields of chemical biology, medicine, and biomaterials. Given their tertiary amide backbone, peptoids lack the capacity to hydrogen‐bond, and as such, controlling secondary structure can be challenging. The incorporation of bulky, charged, or chiral aromatic monomers can be used to control conformation but such building blocks limit applications in many areas. Through NMR and X‐ray analysis we demonstrate that non‐chiral neutral fluoroalkyl monomers can be used to influence the K_cis/trans_ equilibria of peptoid amide bonds in model systems. The *cis*‐isomer preference displayed is highly unprecedented given that neither chirality nor charge is used to control the peptoid amide conformation. The application of our fluoroalkyl monomers in the design of a series of linear peptoid oligomers that exhibit stable helical structures is also reported.

Peptoids (Figure 1) are a class of foldamers that are being developed as potential therapeutics,^1^ biomaterials,^2^ chemical sensors,^3^ and organocatalysts.^4^ They represent an attractive platform for biological and pharmaceutical applications as they are highly resistant to protease degradation.^5^ However, given their tertiary amide backbone, peptoids lack the capacity to form hydrogen bonds so that their secondary structures are dominated by relatively weak interactions. Considerable efforts have been devoted to try and understand the relationships between a peptoid primary sequence and its folded structure.^6^, ^7^, ^8^, ^9^, ^10^ The *cis*/*trans* isomerization of the tertiary amide bond is the major cause of conformational heterogeneity in peptoid oligomers. Despite this, the groups of Zuckermann and Barron have demonstrated that α‐chiral aromatic monomers, such as *N*Spe (**1**), can stabilize the *cis* configuration of the peptoid amide bond largely through steric effects (Figure 1 b, c).^6^, ^7^ Peptoid oligomers of *N*Spe (**1**) fold into stable all *cis*‐amide helices, structurally similar to that of a peptide PPI helix.^6^, ^7^ Gorske and Blackwell found that the synergistic application of steric and non‐covalent n→π* interactions (NCIs) in aromatic systems could also be used to design stable *cis*‐amide peptoid monomers (e.g., *Ns*1npe, **2**).^8^ However, it is not possible to use the aforementioned NCIs to stabilize the *cis*‐amide conformation of alkyl peptoid monomers, and thus the design of stable peptoid helices remains dominated by the use of chiral aromatic residues (e.g., **1** and **2**).^9^ Recently, Faure, Taillefumier, and co‐workers exploited steric effects in the design of a non‐chiral *t*Bu alkyl monomer that has a clear *cis*‐amide preference (*Nt*Bu, **3**).^10^ Whereas **3** offers a route to control peptoid structure that avoids the use of aromatic building blocks, the design of non‐chiral but stable *cis*‐amide alkyl monomers is an area that is still highly underdeveloped.

**Figure 1:**
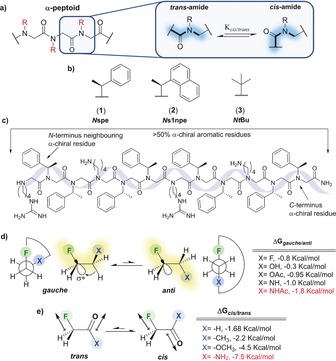
a) General structure of an α‐peptoid and the amide *cis/trans* isomerization process. b) The c*is*‐inducing α‐methyl chiral aromatic monomers **1** and **2** and the sterically demanding *t*Bu monomer **3**. c) Summary of the general sequence requirements for helical secondary structure induction in peptoids. d) The β‐fluorine *gauche* effect. e) Dipole interactions in α‐fluoroamides.

It is in this context that we sought to explore the potential application of fluorine incorporation as a tool to modulate the conformational preferences of alkyl peptoid monomers. Fluorine is a relatively small atom, close in size to hydrogen, but a H to F swap can give rise to significant changes in the electronic and structural properties of a molecule.^11^, ^12^ For example, fluorine may engage in stereoelectronic hyperconjugative interactions with neighbouring C−H bonds (σ(CH)→σ*(CF)). This ability of fluorine to enforce the preorganization of its local environment is most keenly observed when fluorine atoms are located β to electron‐withdrawing groups. In such an arrangement, the fluorine *gauche* effect is seen (Figure 1 d).^12^, ^13^ Notably, the fluorine *gauche* effect is more pronounced in β‐fluoroamides than in other related systems.^12^, ^13^ However, in α‐fluoroamides, CF/C=O dipolar interactions dominate, and the fluorine atom adopts a *trans*‐periplanar arrangement (Figure 1 e).^14^ The peptoid amide bond *cis/trans* equilibrium in our model systems (Figure 2; **10**–**14**) was analysed by a range of established NMR methods (see the Supporting Information for the synthesis of **10**–**14**).8b–8d The non‐fluorinated dipeptoid **10** exhibits a *cis/trans* equilibrium that highly favours the *trans* isomer (CD_3_CN; Δ*G*_*cis*/*trans*_=0.28, *K*_*cis*/*trans*_=0.66; Figure 3 a). Relative to this, all of the fluorinated dipeptoids (**11**–**13**) showed an enhanced preference for the *cis*‐amide conformation (Figure 3). Initial NMR analysis (in CD_3_CN) revealed that even the introduction of a single fluorine atom β to the amide bond enhanced the *cis*‐amide preference by 0.37 kcal mol^−1^ when compared to **10**. Incorporation of a second fluorine atom further increased the *cis*‐amide preference. Indeed, unlike **10** and **11**, the difluorinated dipeptoid **12** shows a highly predominant *cis*‐amide conformation in solution, with Δ*G*_*cis*/*trans*_=−0.42 kcal mol^−1^ and *K*_*cis*/*trans*_=2.05 (Figure 3 a). We were surprised to note that the *K*_*cis/trans*_ value exhibited by **12** is comparable to those seen when *cis*‐inducing chiral aromatic monomers are used (e.g., for **14**, *K*_*cis*/*trans*_=2.08 in CD_3_CN). Initial NMR analysis revealed a linear correlation between the Δ*G*_*cis/trans*_ values observed and the electron‐withdrawing character of the C_α_ carbon substituent when one or two fluorine atoms were incorporated (e.g., **10** to **12**; Figure 3 b, c). This correlation indicated a clear relationship between the inductive properties of the fluorinated groups and the *cis*/*trans* ratios produced (σ_I_; Figure 3 c, e).^15^ An even greater *cis*‐isomer preference was observed when the N3*f*Et‐containing dipeptoid **13** was analysed (CD_3_CN; *K*_*cis*/*trans*_=2.24).

**Figure 2:**
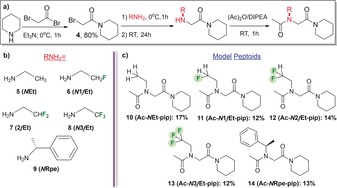
a) Synthesis of model piperinidyl acetamides **10**–**14**. b) Peptoid monomers used in this study. c) Reference model dipeptoids (**10**, **14**) and novel β‐fluoroethyl (**11**), β‐difluoroethyl (**12**), and β‐trifluoroethyl (**13**) systems.

**Figure 3:**
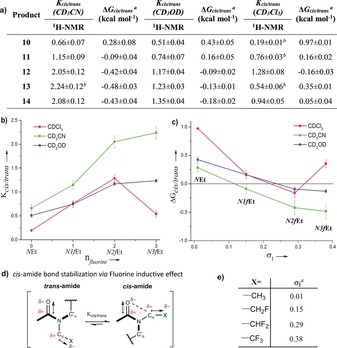
a) Average *K*_*cis*/*trans*_ values in model systems **10**–**14**. [a] From each replica, Δ*G*=−RT ln(*K*_*cis*/*trans*_) at 25 °C. Averages and standard deviation values are given for *n*=6 or *n*=4 (^*b*^). b) Average *K*_*cis*/*trans*_ values vs. the number of fluorine atoms present (*n*_f_). c) Correlation between Δ*G*_*cis/trans*_ and C_α_‐substituent field/inductive constants (*σ*_I_). d) Schematic representation of the proposed peptoid *cis*‐amide isomer stabilization by inductive factors. e) Inductive constants of C_α_ groups.^15^

To determine the nature of the interactions present within **11**–**13**, we next examined how the solvent polarity influenced the *cis*/*trans* ratios. When using protic MeOD, the *K*_*cis*/*trans*_ values observed were collectively lower than those found in CD_3_CN (Figure 3 a, b). However, the general increases in the *cis*‐isomer preference produced upon fluorine incorporation were still clearly maintained. This outcome indicates that hydrogen bonding is not involved in the *cis*‐isomer stabilization observed in **11**–**13** (Figure 3 a–c). The use of CDCl_3_ also reduced the *K*_*cis*/*trans*_ values recorded, and this general trend is in good agreement with previous observations reported for other model peptoid systems.8b–8d Despite this general decrease, the *cis*‐amide preferences of **11** and **12** in CDCl_3_ were still significantly greater than that of the control **10**. Upon moving from no fluorine atoms (**10**) to either one (**11**) or two (**12**), relative changes in the free energy of −0.81 kcal mol^−1^ and −1.13 kcal mol^−1^, respectively, were seen.

These relative Δ*G*_*cis*/*trans*_ changes are in fact larger in CDCl_3_ (non‐polar) than in CD_3_CN (polar), and this finding supports the hypothesis that an electronic *cis*‐stabilizing effect is occurring. Remarkably, in CDCl_3_, the *N2f*Et monomer (**7**) actually has a greater ability to stabilise a *cis*‐amide preference than the chiral aromatic *NR*pe monomer (**9**; *K*_*cis*/*trans*_=1.28 vs. 0.94; Figure 3 a). The N3*f*Et‐containing dipeptoid **13** was found to be more affected in CDCl_3_, and it produced a strong out‐of‐trend shift to the *trans* isomer (Figure 3 a, b). Given this observation, we hypothesised that the energetic penalty that **13** experiences in the *cis* conformation may arise from an increased solvation barrier as non‐polar solvents are well‐known to disfavour structures where large dipoles are present. As depicted in Figure 3 d, the overall dipolar moment within *trans*‐**13** is likely to be lower than that within the corresponding *cis* isomer as the carbonyl and side‐chain dipoles are opposed. This solvation effect should be less pronounced in **11** and **12** as they have weaker dipoles (Figure 3 e).

Next, we explored the role that fluorine/amide *gauche* interactions could play in enhancing the *cis*‐isomer preferences. The vicinal (three‐bond coupling) ^3^ *J*_HF_ coupling constants were thus analysed (Figure 4 a).^17^, ^18^ In **11**, a ^3^ *J*_HF,cal_ value of 20.0 Hz was calculated for an ideal fluorine/amide *gauche* conformation of the side chain (*g*). A significantly lower value of 8.0 Hz was obtained for the alternative fluorine/amide *anti* configuration (*a*). The experimental value found within the predominant *cis*‐**11** isomer was ^3^ *J*_HF,obs_=25.7 Hz (CD_3_CN). This result strongly suggested an overall fluorine/amide *gauche* orientation within the side chain. Two staggered conformations for **12** were also examined, and the experimental value of ^3^ *J*_HF,obs_=14.9 Hz was in perfect agreement with an *anti*/*gauche* conformation (Figure 4 b). This finding indicates that only one F atom may be actually located *gauche* to the peptoid amide group, and this is contrary to the more intuitive (+*g*/−*g*) configuration that would be expected. No significant variations in the experimental ^3^ *J*_HF,obs_ values were seen within each *cis/trans* pair in any of the solvents tested, indicating that the fluorine/amide relative arrangement is retained between conformers. The NMR results suggest that fluorine *gauche* effects are not solely responsible for the *cis*‐isomer preferences observed in **11** and **12**. In **13**, fast rotation of the CF_3_ group was inferred as the experimental ^3^ *J*_HF,obs_ value greatly deviated from the calculated value, which assumes a static fluorine/amide arrangement (Figure 4 c).

**Figure 4:**
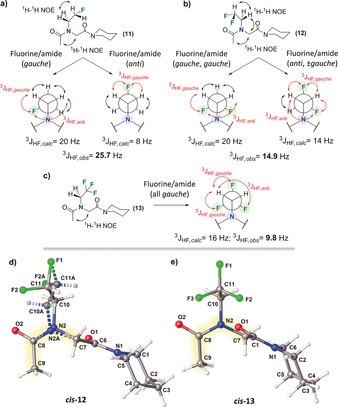
Theoretical versus experimental vicinal ^3^ *J*_H‐F_ coupling constants within a) **11**, b) **12**, and c) **13** in their preferred *cis* conformations (CD_3_CN). Ball‐and‐stick representations of the crystal structures of *cis*‐amides d) **12** and e) **13**.^16^

We were able to crystallize dipeptoids **12** and **13** from their EtOAc saturated solutions.^19^ The solid‐state structures for **12** and **13** and the conformations suggested by NMR analysis were in perfect agreement (Figure 4 d, e). It is worth noting that from the crystal structures of **12** and **13**, it would appear that neither fluorine–oxygen repulsive interactions nor unfavourable steric clashes contribute substantially to the *cis*/*trans* conformation preferences observed in these systems. As shown in Figure 4 d, e, the fluorinated groups in **12** and **13** display a well‐defined orthogonal orientation relative to the amide bond planes. This orientation minimizes the potential steric clashes and/or electronic repulsion imposed by the CHF_2_/CF_3_ groups. Overall, our findings support the hypothesis that the enhanced *cis*‐amide preferences observed in **11**–**13** arise from the inductive effects imposed by the fluorine atom(s). As the polarization at C_α_ increases, the peptoid *cis*‐amide preference also increases. We propose that this is due to the fact that the δ+ on C_α_ can form a *syn*‐periplanar stabilising dipolar interaction with the amide C=O (Figure 3 d).

Encouraged by the *cis*/*trans* ratios achieved in the model systems (**11**–**13**), we then moved to see if the non‐chiral fluoroalkyl monomers could be exploited to design stable peptoid helices. To this end, we designed a control 15‐mer peptoid, Pep.1, using non‐chiral alkyl ethylamine monomers (Figure 5). A single *N*Spe residue was introduced as a chiral reporter for circular dichroism (CD) spectroscopy. The Pep.1 sequence was then altered by substituting in the various fluorinated monomers (**6**–**8**) in place of some, but not all, of the *N*Et residues (group 1, Pep.2–4). In a second group of fluorinated peptoids, all of the *N*Et residues present were replaced (group 2, Pep.5–7). We were pleased to see that structural analysis of the peptoid oligomers Pep.2–Pep.7 by CD spectroscopy revealed the presence of stable peptoid helices (Figure 5 b, c). In all of the peptoids studied, substitution of the *N*Et residues by any of the fluorinated monomers clearly enhanced the CD minima at 218 nm (*M*_*θ*,218_), which is characteristic of an increase in helicity. When five substitutions (*N*Et for a fluoroalkyl monomer) were made in non‐consecutive positions (group 1, Pep.2–4, *n*_f_=5), the overall increases in molar ellipticity were found to correlate with the number of fluorine atoms within the side chain. For example, upon going from the non‐fluorinated peptoid (Pep.1) to the *N*1*f*Et‐based analogue (Pep.2), a change in molar ellipticity of Δ*M*_*θ*,218_=6660 deg cm^2^ dmol^−1^ was observed. Similarly, incorporation of N2*f*Et (**7**) and N3*f*Et (**8**) produced approximately two‐ and threefold higher increases in *M*_*θ*,218_ (Pep.3, Δ*M*_*θ*,218_=12 640; Pep.4, Δ*M*_*θ*,218_=17 000 deg cm^2^ dmol^−1^).

**Figure 5:**
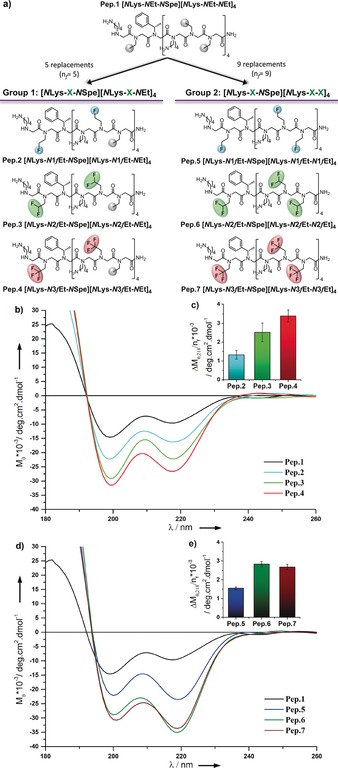
a) Peptoid oligomers Pep.1–Pep.7. b, d) Average CD spectra and c, e) average absolute increases in *M*_*θ*,218_ per fluorine residue incorporated (Δ*M*_*θ*,218_/*n*_f_) in peptoid sequences from group 1 (Pep.1–Pep.4, *n*_f_=5; parts b, c) and group 2 (Pep.5–Pep.7, *n*_f_=9; parts d, e).

When the more heavily substituted peptoids from group 2 were analysed, higher values of *M*_*θ*,218_ were found, indicating that the secondary structure enhancement induced by the incorporation of fluorinated side chains has an overall accumulative behaviour (Pep.5–7; Figure 5 d, e). In fact, the average increases in *M*_*θ*,218_ produced by each *N*1*f*Et (**6**) and *N*2*f*Et (**7**) monomer introduced in these sequences were higher than those observed when only five replacements were made (Δ*M*_*θ*,218_/*n*_f_; Pep.2 vs. Pep.5 and Pep.3 vs. Pep.6; Figure 5 c–e). These results revealed a broadly cooperative effect between neighbouring fluorinated side chains. Interestingly, this synergy between consecutive monomers did not occur when *N*3*f*Et monomers were used (Pep.4 vs. Pep.7). Based on our crystal structure data, we could evaluate that the volumes of the CHF_2_ and CF_3_ groups are 29.63 and 40.47 Å^3^ respectively. Based on this, we hypothesise that the behaviour seen for Pep.7 may be related to unfavourable steric and/or repulsive interactions between the CF_3_ groups of adjacent *N*3*f*Et monomers. Overall, the results from the CD studies (Figure 5) are highly unprecedented owing to the fact that none of the fluorinated monomers investigated are either chiral, aromatic, or charged, and yet they can support the formation of stable peptoid helices.

In summary, we have shown that the selective and strategic incorporation of fluorine atom(s) offers a new route to control the amide bond isomerism in peptoids containing alkyl side chains. Through NMR and X‐ray analysis we demonstrated that simple non‐chiral fluoroalkyl monomers can be used to influence the key *K*_*cis*/*trans*_ equilibria of a peptoid amide bond and induce a remarkable degree of *cis*‐amide preference. The *cis*‐isomer preference is highly unprecedented given that neither chirality nor charge was being used to control the peptoid amide conformation. The data gathered support the hypothesis that inductive effects imparted by the fluorine atom(s) and not fluorine *gauche* effects underpin the *cis*‐isomer stabilization observed. The novel fluoroalkyl monomers were also used to prepare a series of peptoid oligomers that exhibited stable helical structures despite only having one chiral aromatic residue. The application of fluorine in the design of alkyl monomers offers a new approach to control amide bond isomerism in peptoid sequences, overcoming the current need for high levels of chiral side chains. Given the lack of alternatives available, the *N1f*Et, *N2f*Et, and *N3f*Et alkyl monomers offer exciting new tools to design structurally stable peptoid systems with applications in a range of areas, including medicine and biomaterials.

## Conflict of interest

The authors declare no conflict of interest.

## Supporting information

As a service to our authors and readers, this journal provides supporting information supplied by the authors. Such materials are peer reviewed and may be re‐organized for online delivery, but are not copy‐edited or typeset. Technical support issues arising from supporting information (other than missing files) should be addressed to the authors.

Supplementary
