# Supplementary material for: Stabilising Peptoid Helices Using Non‐Chiral Fluoroalkyl Monomers
Source: Angew Chem Int Ed Engl. 2018 Jun 27;57(33):10549–53. doi: 10.1002/anie.201804488 (PMC6099454; doi:10.1002/anie.201804488)
Supplement: Supplementary file 1 — Supplementary [file ANIE-57-10549-s001.pdf]

Angewandte Chemie
Eine Zeitschrift der Gesellschaft Deutscher Chemiker

Supporting Information

Stabilising Peptoid Helices Using Non-Chiral Fluoroalkyl Monomers

*Diana Gimenez, Juan A. Aguilar, Elizabeth H. C. Bromley, and Steven L. Cobb**

anie_201804488_sm_miscellaneous_information.pdf

Table of Contents

Materials and general methods **S4**

Model piperinidyl monomer synthesis **S5**

Synthesis of 2-bromo-1-piperidinyl ethanone *S5*

Bromine displacement and monomer acetylation *S5*

Product purification *S5*

Model piperinidyl-acetamide characterization and NMR analysis **S6**

General procedure for NMR characterization and $K_{cis/trans}$ determination *S6*

NMR data and characterization of model compounds 10-14 .... *S7*

***10:** Ac-NEt-pip.* *S7*

Structure and HR QToF-LC/MS *S7*

Characterization in CD_3CN *S8*

Characterization in CD_3OD *S13*

Characterization in $CDCl_3$ *S18*

***11:** Ac-N ^{1f}Et-pip.* *S22*

Structure and HR QToF-LC/MS *S22*

Characterization in CD_3CN *S23*

Characterization in CD_3OD *S28*

Characterization in $CDCl_3$ *S33*

***12:** Ac-N^{2f}Et-pip.* *S38*

Structure and HR QToF-LC/MS *S38*

Characterization in CD_3CN *S39*

Characterization in CD_3OD *S44*

Characterization in $CDCl_3$ *S49*

***13**: Ac-N^{3f}Et-pip.* *S54*

Structure and HR QToF-LC/MS *S54*

Characterization in CD_3CN *S55*

Characterization in CD_3OD *S60*

Characterization in $CDCl_3$ *S65*

***14**: Ac-NRpe-pip.* *S70*

Structure and HR QToF-LC/MS *S70*

Characterization in CD_3CN *S71*

Characterization in CD_3OD *S76*

Characterization in $CDCl_3$ *S81*

$K_{cis/trans}$ numeric evaluation *S86*

Vicinal $^3J_{H-F}$ coupling constants *S87*

X-ray Crystal structure data ***S87***

Sample crystallization S87

Crystal data of peptoid monomer 12 .... S88

Crystal data of peptoid monomer 13 .... S89

Model peptoid synthesis and characterization: **S90**

Synthesis of peptoid oligomers pep.1-7 .... S90

HPLC, Maldi-TOF and HR-MS Characterization S91

Pep.1 *[NLysNEtNSpe] [NLysNEtNEt]*$_4$ S92

Pep.2 *[NLysN*^{1f}*EtNSpe] [NLysN*^{1f}*EtNEt]*$_4$ S93

Pep.3 *[NLysN*^{2f}*EtNSpe] [NLysN*^{2f}*EtNEt]*$_4$ S94

Pep.4 *[NLysN*^{3f}*EtNSpe] [NLysN*^{3f}*EtNEt]*$_4$ S95

Pep.5 *[NLysN*^{1f}*EtNSpe] [NLysN*^{1f}*EtN*^{1f}*Et]*$_4$ S96

Pep.6 *[NLysN*^{2f}*EtNSpe] [NLysN*^{2f}*EtN*^{2f}*Et]*$_4$ S97

Pep.7 *[NLysN*^{3f}*EtNSpe] [NLysN*^{3f}*EtN*^{3f}*Et]*$_4$ S98

CD Spectra Studies: **S99**

^{19}F-NMR evaluation of the TFA content S99

General method for sample preparation and analysis S99

CD spectra data S101

Pep.1 *[NLysNEtNSpe] [NLysNEtNEt]*$_4$ S101

Pep.2 *[NLysN*^{1f}*EtNSpe] [NLysN*^{1f}*EtNEt]*$_4$ S102

Pep.3 *[NLysN*^{2f}*EtNSpe] [NLysN*^{2f}*EtNEt]*$_4$ S103

Pep.4 *[NLysN*^{3f}*EtNSpe] [NLysN*^{3f}*EtNEt]*$_4$ S104

Pep.5 *[NLysN*^{1f}*EtNSpe] [NLysN*^{1f}*EtN*^{1f}*Et]*$_4$ S105

Pep.6 *[NLysN*^{2f}*EtNSpe] [NLysN*^{2f}*EtN*^{2f}*Et]*$_4$ S106

Pep.7 *[NLysN*^{3f}*EtNSpe] [NLysN*^{3f}*EtN*^{3f}*Et]*$_4$ S107

Supplementary References: **S108**

Materials and general methods

All chemicals and solvents were analytical grade and used without further purification.

Liquid chromatography (LC)-mass spectrometry (ESI-MS) analyses were performed on a Acquity UPLC BEH C_{18} 1.7μm (2.1mm x 50mm) columm using a Waters Acquity UPLC system equipped with a photodiode array detector, providing absorbance data from 210 nm to 400 nm. A gradient with eluent I (0.1% HCOOH in water) and eluent II (0.1% HCOOH in acetonitrile) rising linearly from 5 to 95% of II during *t*=0.2–4.0 min was applied at a flow rate of 0.6 ml min^{-1} after 0.2 min of 95% solvent I equilibration. Analytical HPLC was performed on a X-Bridge C_{18} column (5.3μm, 4.6 x 100 mm, 40°C) using a Pelking-Elmer 200 series lc system supplied with auto-sampler, UV/Vis detector and a Peltier column oven. A linear gradient rising from eluent III (95:5:0.05% H_2O:MeCN:TFA) to 100% of eluent IV (5:95:0.03% H_2O:MeCN:TFA) during *t*=30 min plus 5 min of isocratic IV elution from *t*=30–35 min was applied at a flow rate of 1 ml min^{-1}. Detection was performed in all cases at λ=220nm.

Semi-preparative HPLC purification was performed on a Discovery Bio wide pore C_{18}-5 column from Supelco (5 μm, 25cm × 10 mm), using a Pelking-Elmer 200 lc pump coupled to a Waters 486 tunable absorbance detector settled up at λ=220nm. A gradient with eluent V (95:5:0.1% H_2O:MeCN:TFA) and eluent VI (5:95:0.1% H_2O:MeCN:TFA) was applied where solvent VI was firstly rose linearly from 0 to 100% during *t*=60 min and finally maintained isocratically for 5 min at a flow rate of 2 ml min^{-1}.

High-resolution QToF-LC/MS analyses were performed on a Acquity UPLC BEH C_{18} 1.7μm (2.1mm x 50mm) columm using a Waters Acquity UPLC system coupled to Micromass QToF Premier mass spectrometer, also equipped with a photodiode array detector providing absorbance data from 210 nm to 400 nm. A gradient with eluent I (0.1% HCOOH in water) and eluent II (0.1% HCOOH in acetonitrile) rising linearly from 0 to 99% of II during *t*=0.0–5.0 min was applied at a flow rate of 0.6 ml min^{-1}.

NMR spectra studies were recorded on a Varian VNMRS-700 NMR spectrometer at 298 K. Depending on the sample, ^{1}H-NMR has been obtained at 600-700MHz using 2-8 scans with a relaxation delay of 10s between them. ^{13}C-NMR has been obtained at 151-176MHz (1000-1208 repetitions, 2.5-3.0s of relax. delay) and bi-dimensional ^{1}H-^{1}H NOESY experiments have been run with a minimum mixing time of 150 ms, a spectral width between 6,000-8,000Hz in both dimensions and a minimum of 2 transients with 2x200 increments. Final minimum FT size = 2048 x 2048 points. Independent ^{19}F-NMR has been performed in at 376MHz in a Bruker Advance spectrometer. All data has been processed using Mestrenova® software, and chemical shifts are reported in p.p.m., relative to deuterated solvent peaks as internal standards (δH, $CDCl_3$: 7.26 p.p.m.; δC, $CDCl_3$ 77.16 p.p.m., δH, CD_3CN 1.94 p.p.m.; δC, CD_3CN 1.32 p.p.m.; δH, CD_3OD: 3.34 p.p.m.; δC, CD_3OD 49.00 p.p.m.).

The X-ray single crystal data for *cis*-**12** and *cis*-**13** has been collected using λMoKα radiation (λ =0.71073Å) on a Bruker D8Venture (Photon100 CMOS detector, IμS-microsource, focusing mirrors) diffractometer equipped with a Cryostream (Oxford Cryosystems) open-flow nitrogen cryostats at the temperature 120.0(2)K. Both structures were solved by direct methods and refined by full-matrix least squares on F2 for all data using Olex2[1] and SHELXTL software.[2] All non-disordered non-hydrogen atoms were refined anisotropically, hydrogen atoms were placed in the calculated positions and refined in riding mode. Disordered atoms of minor disorder component in structure *cis*-**12** were refined isotropically. Occupation factors of atoms of disordered fragment were fixed as 0.8 and 0.2, structure *cis*-**12** was refined as a merohedral twin (0 1 0 1 0 0 0 0 -1). Crystal data and parameters of refinement are listed in Supplementary Tables 5 and 6. Crystallographic data for the structures have been deposited with the Cambridge Crystallographic Data Centre as supplementary publications CCDC-1567326-1567327.

CD spectra studies have been carried out at 24°C in a Jasco J-1500 spectrometer, provided with a MCB-100 mini circulation bath from the same brand. All samples were recorded as the average of 2 scans (1.00 nm data pitch, continuous scanning mode, 50 nm min^{-1} scanning speed, 3 nm bandwidth) using a QS high precision cell with 0.1cm of path length from Hellma Analytics.

Model piperinidyl monomer synthesis

Synthesis of 2-bromo-1-piperidinyl ethanone: 0.5g of bromoacetyl bromide (2.73 mmol) in anhydrous $CHCl_2$ (5mL) were added drop-wise to a pre-chilled solution of piperidine in dry $CHCl_2$ (2.48 mmol, 20mL) placed under Argon. Once the addition was completed the final mixture was allowed to warm up to room temperature for 1 hour before being washed twice with water (20 mL), with a 10% Citric Acid solution (20 mL) and $NaHCO_3$ sat.(20

mL). The final organic layer was dried over $MgSO_4$ and the solvent evaporated under reduced pressure, leading to the desired 2-bromo-1-piperidinyl ethanone as a dark brown oil (80% yield). No further purification was done before the next step. ^{1}H NMR (400 MHz, Chloroform-*d*): δ 3.86 (s, 2H), 3.59 – 3.53 (m, 2H), 3.47 – 3.41 (m, 2H), 1.76 – 1.52 (m, 6H). ^{13}C NMR (101 MHz, $CDCl_3$): δ 165.24, 48.08, 43.44, 26.33, 26.22, 25.50, 24.40. HR-ESI/MS: Expected *m/z*: 205.01 (100%), 207.08 (97.03%), observed: *m/z*: 206.02, 207.96 [M+H$^+$].

Bromine displacement and monomer acetylation: 300 mg of 2-bromo-1-piperidinyl ethanone (1.46 mmol) were dissolved in anhydrous CH_3CN (20 mL) and the solution cooled to 0^0C under Argon. 1.2 equivalents of the desired amines in the same solvent (A1-A5, 1.75 mmol, 10 mL) were then slowly drop-wise added, the final mixture allowed to warm up to room temperature and finally let to react overnight. When amines were employed as hydrochloride salts, triethylamine was previously added to their solutions (Et_3N, 3.5 mmol). CH_3CN was evaporated and the oily residue dissolved in 10 mL of $CHCl_2$. Then, 0.5 mL of acetic anhydride (5.28 mmol) and 0.475 mL of N,N-diisopropylethylamine ($(i\text{-}Pr)_2EtN$, 2.77 mmol) were added and let to react for 30 minutes. Once accomplished, the mixture was washed with 10% citric acid (15 mL), $NaHCO_3$ sat. (15 mL) and dried over $MgSO_4$. Removal of the solvents in vacuum yielded to a yellow-to-brown oil that was directly deposited on silica for appropriate purification.

Product purification: Final purification of the desired products was achieved by silica gel flash chromatography with a gradient of AcOEt in hexane from 0-100%. Column elution was followed by TLC chromatography. Fractions containing single spots corresponding to the same retention times were joined together, evaporated under reduced pressure and analysed by LC-MS, ^{19}F-NMR and ^{1}H-NMR in order to verify their identity. In all cases between 25 and 60mg of the oily pure target products were collected for NMR analysis. Final isolated yields: **10**: 37mg, 95.4% purity, 16.6% yield; **11**: 44mg, 95.9% purity, 12.5% yield; **12**: 54mg, 97.5% purity, 14.5% yield; **13**: 49mg, 96.2% purity, 12.2% yield; **14**: 59mg, 95.62% purity, 13.4% yield.

Model piperinidyl-acetamide characterization and NMR analysis

General procedure for NMR characterization: For NMR analysis, typically 20 mg of the pure compounds were dissolved in 0.7 mL of the corresponding deutered solvent, $CDCl_3$, CD_3CN or CD_3OD and then filtered and degassed prior to analysis. For each sample a full set of experiments was done, where ^{1}H-NMR, ^{1}H-^{1}H NOESY, ^{1}H-^{1}H COSY and ^{1}H-*psyche* were used in order to assign independently the proton signals arising from the *cis* and the *trans* isomers, as well as for the analysis of spatial contacts. In fluorinated systems ^{19}F-NMR was further employed to obtain a second independent estimation of $K_{cis/trans}$. Characterization for both isomers was completed by ^{13}C-NMR, ^{1}H-^{13}C HSQCAD and when needed ^{1}H-^{13}C HMBCAD in order to achieve full carbon assignation.

NMR data and characterization of model compounds 10-14:

10: Ac-Et-pip.

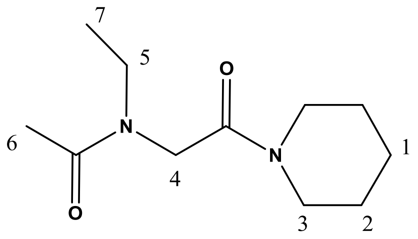

HRMS: calculated for $C_{11}H_{21}N_2O_2$ 213.1603 [M+H]+, found: 213.1616 Da.

.

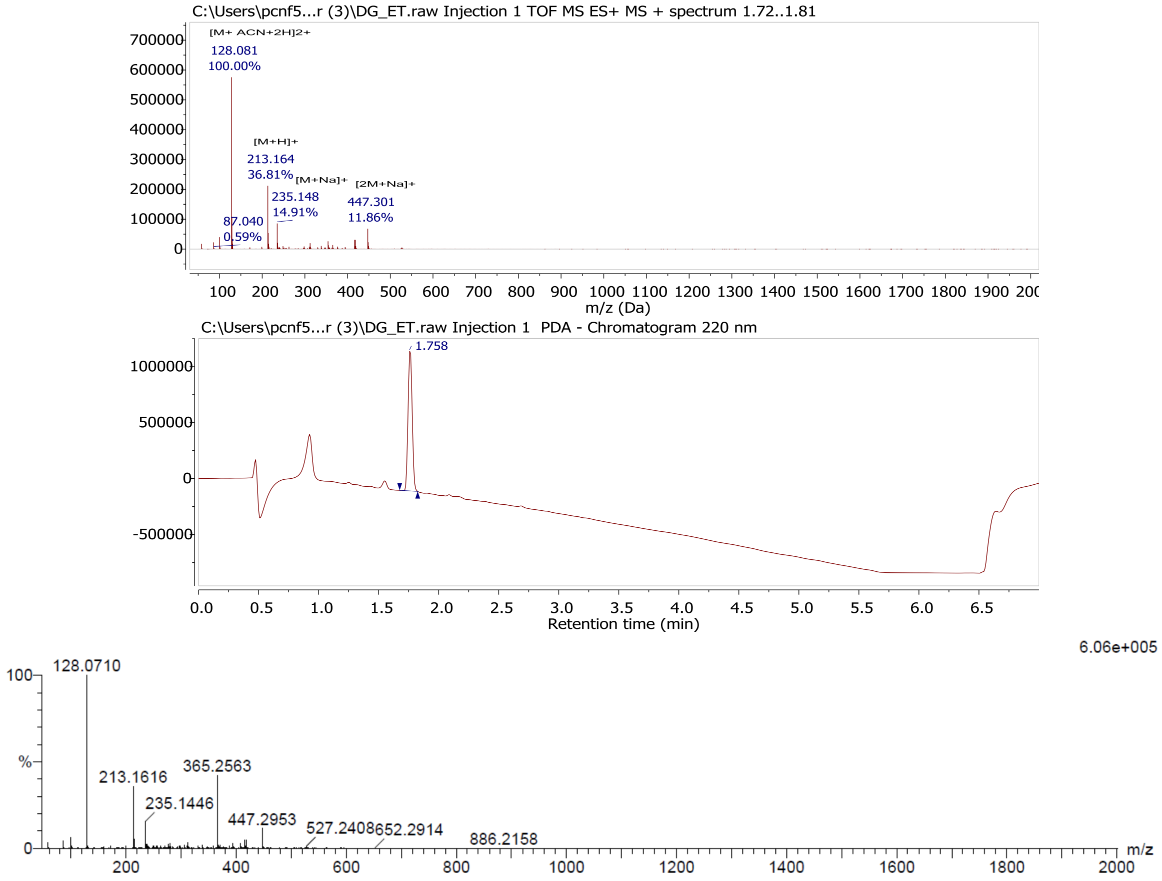

Figure S-1. Structure, HR (QToF)-LC/MS trace and elemental composition of model piperinidyl acetamide **10**.

^{1}H NMR (700 MHz, Acetonitrile-*d*$_3$) Rotamer *trans*: δ ^{1}H NMR: 4.09 (s, 2H, $H_{4,trans}$), 3.51-3.36 (m, 4H, $H_{3,ax+eq,trans}$), 3.33 (q, *J* = 7.2 Hz, 2H, $H_{5,trans}$), 2.04 (s, 3H, $H_{6,trans}$), 1.66-1.43 (m, 6H, $H_{1-2,ax+eq,trans}$), 1.12 (t, *J*=7.2 Hz, 3H, $H_{7,trans}$); Rotamer *cis*: δ ^{1}H NMR: 4.10 (s, 2H, $H_{4,cis}$), 3.51-3.36 (m, 4H, $H_{3,ax+eq,cis}$), 3.27 (q, *J* = 7.2 Hz, 2H, $H_{5,cis}$), 1.84 (s, 3H, $H_{6,cis}$), 1.66-1.43 (m, 6H, $H_{1-2,ax+eq,cis}$), 1.00 (t, *J*=7.2 Hz, 3H, $H_{7,cis}$).
^{13}C NMR (176 MHz, Acetonitrile-*d*$_3$) Rotamer *trans*: δ ^{13}C NMR: 46.37, 43.50 (CH_2, $C_{3,trans}$); 46.93 (CH_2, $C_{4,trans}$), 44.80 (CH_2, $C_{5,trans}$); 27.02, 26.40, 25.19 (CH_2, $C_{1-2,trans}$); 21.26 (CH_3, $C_{6,trans}$); 13.88 (CH_3, $C_{7,trans}$); Rotamer *cis*: δ ^{13}C NMR: 50.35(CH_2, $C_{4,cis}$), 46.28, 43.79 (CH_2, $C_{3,cis}$), 42.47 (CH_2, $C_{5,cis}$); 27.07, 26.44, 25.13 (CH_2, $C_{1-2,cis}$); 21.86 (CH_3, $C_{6,cis}$); 13.12 (CH_3, $C_{7,cis}$); Quaternary carbons: δ ^{13}C NMR: 171.71, 170.84, 167.31, 167.25.

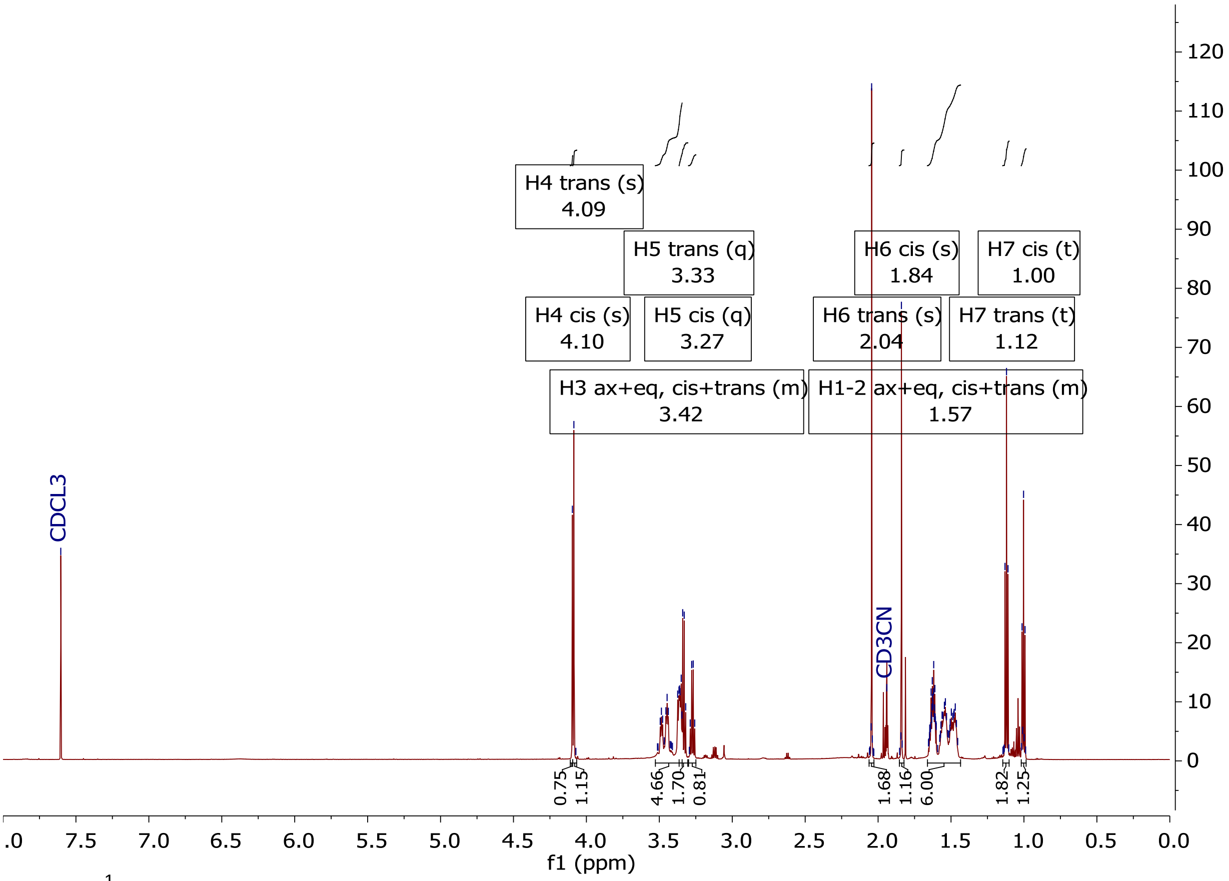

Figure S-2. ^{1}H-NMR spectrum of compound **10** recorded in *acetonitrile-d$_3$* at room temperature.

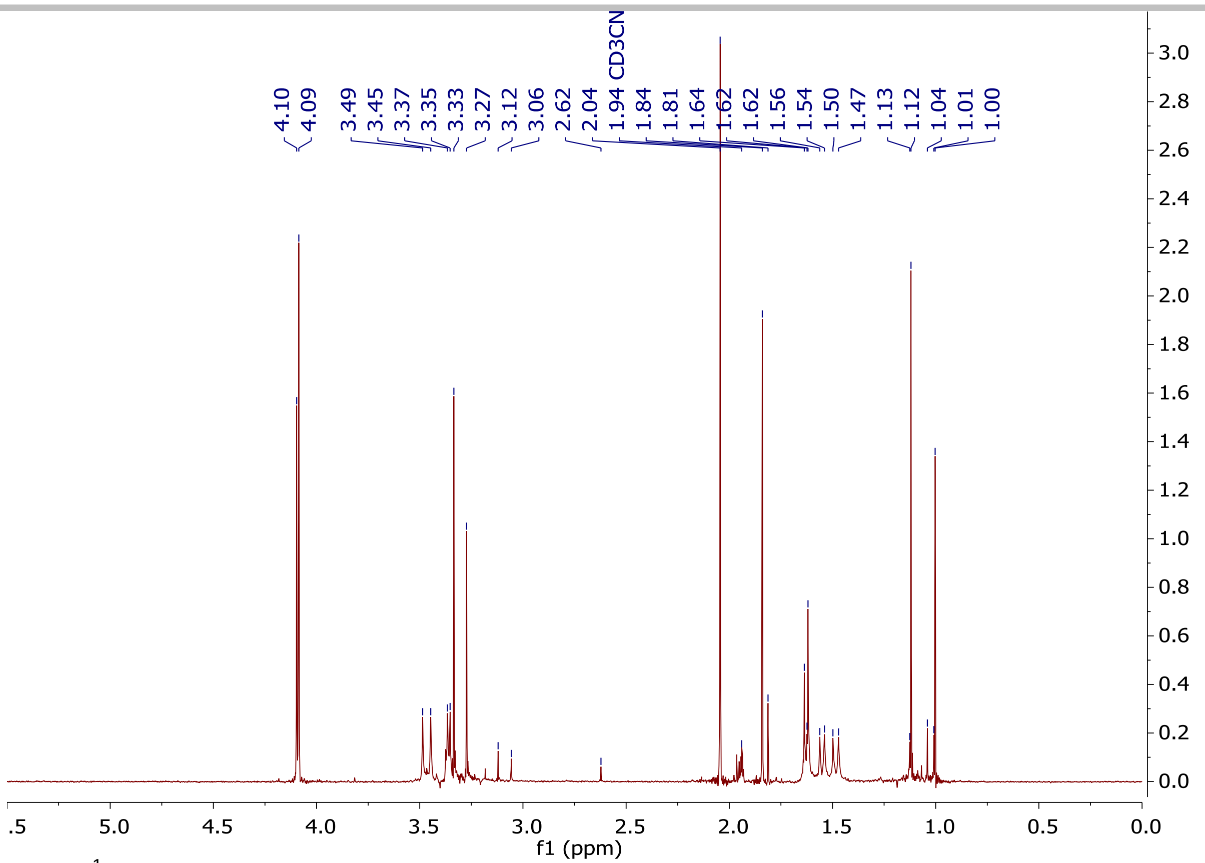

Figure S-3. ^{1}H-*psyche* spectrum of compound **10** recorded in *acetonitrile-d_3* at room temperature.

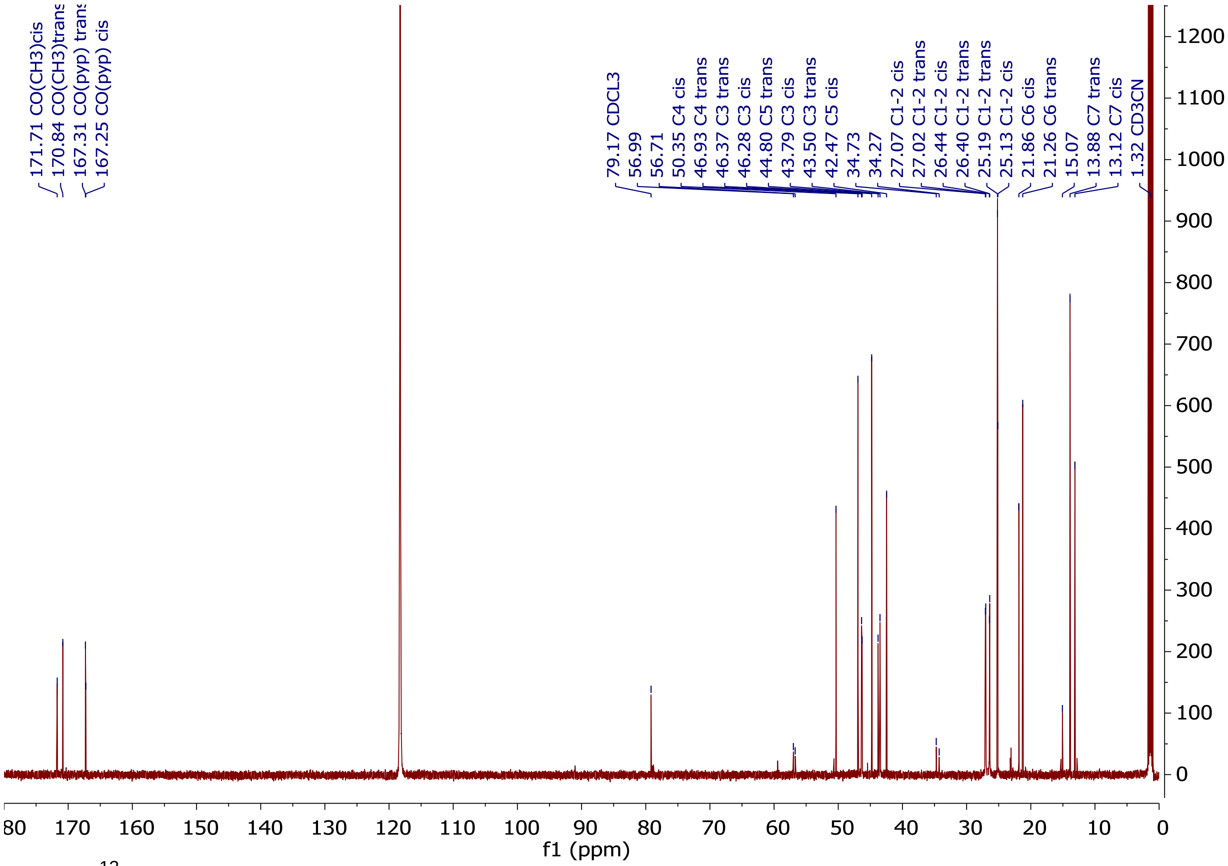

Figure S-4. ^{13}C NMR spectrum of compound **10** recorded in *acetonitrile-d_3* at room temperature.

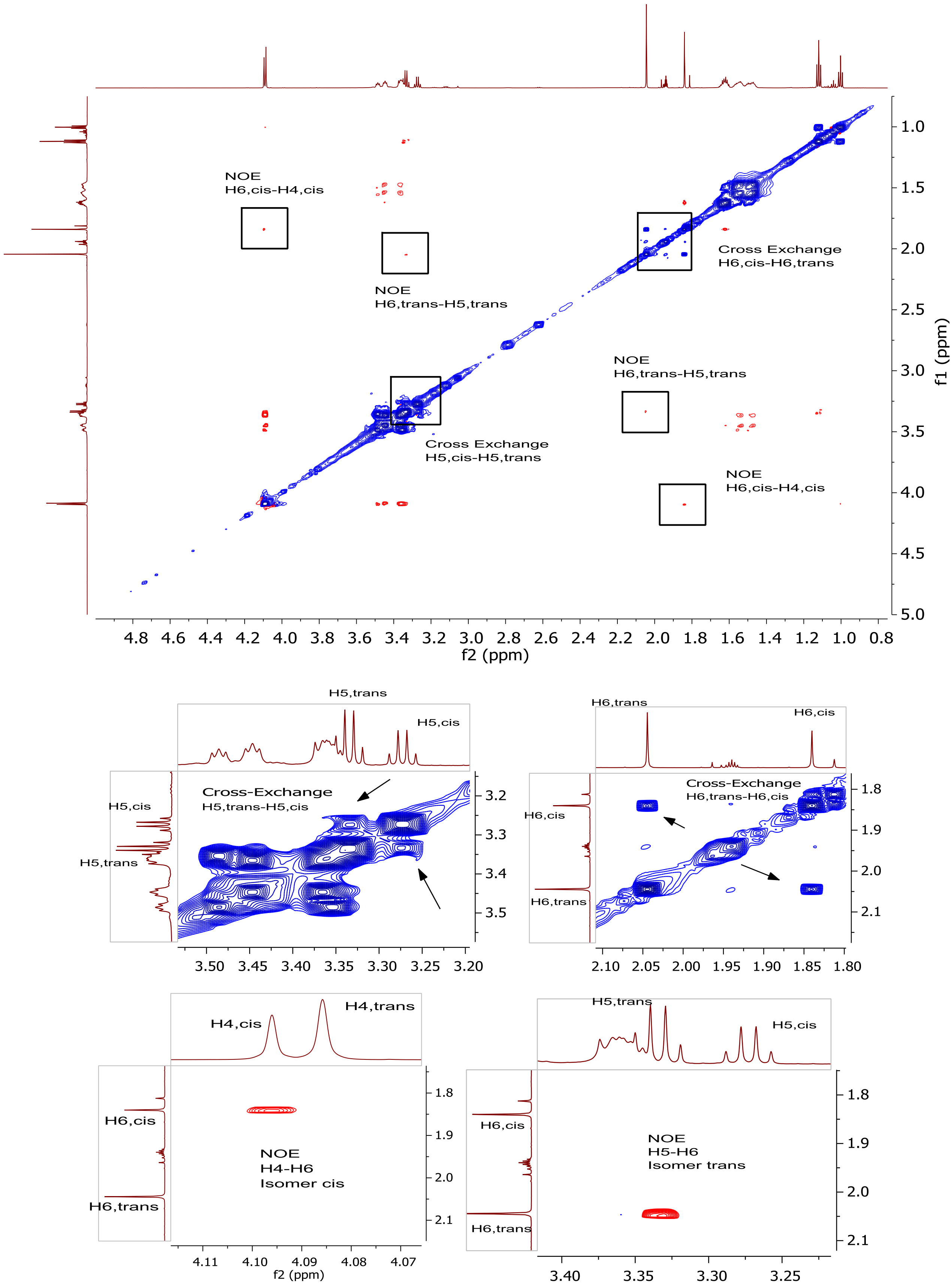

Figure S-5. ^1H-^1H NOESY spectrum of compound **10** recorded in *acetonitrile-d$_3$* at room temperature. Insets showing the specific spatial contacts that are characteristic of each isomer and allowed the identification of *cis* and *trans* signals.

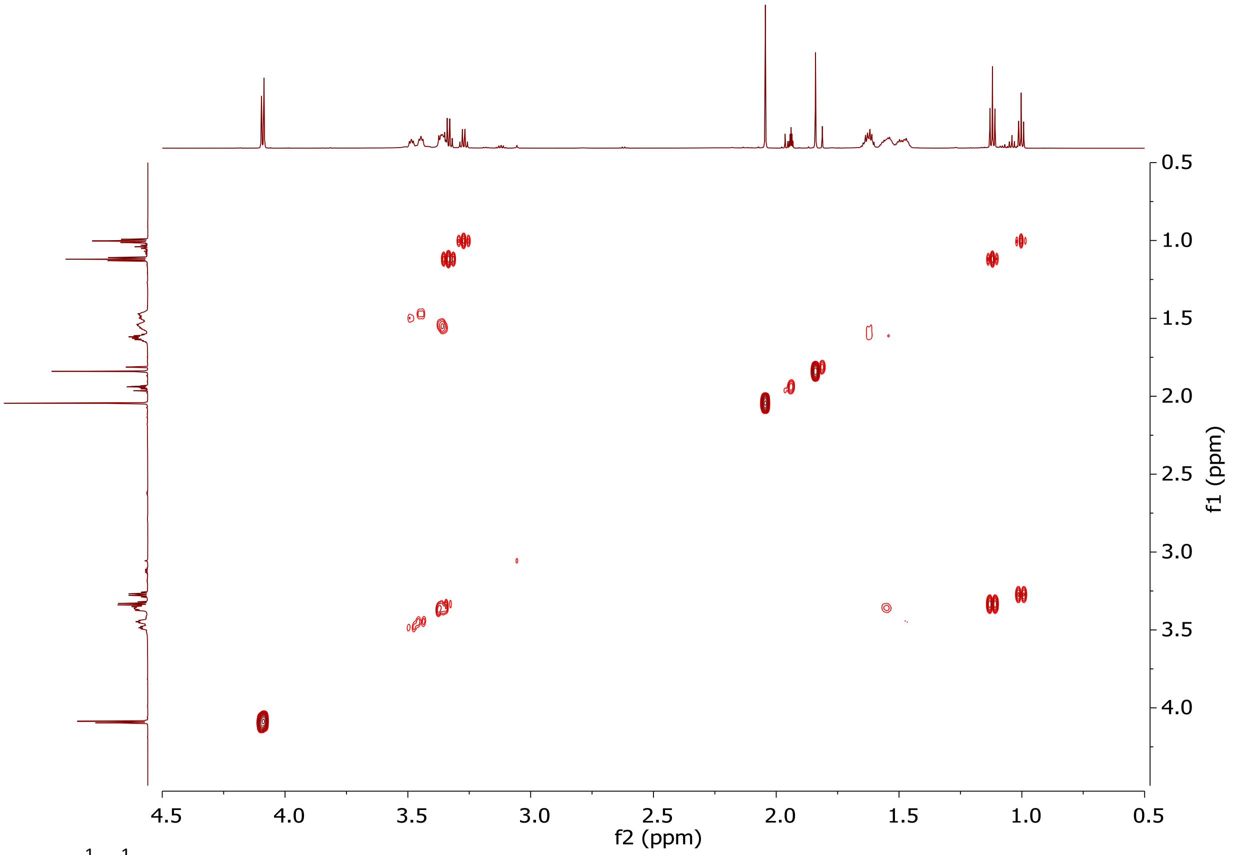

Figure S-6. ^{1}H-^{1}H COSY spectrum of compound **10** recorded in *acetonitrile-d_3* at room temperature.

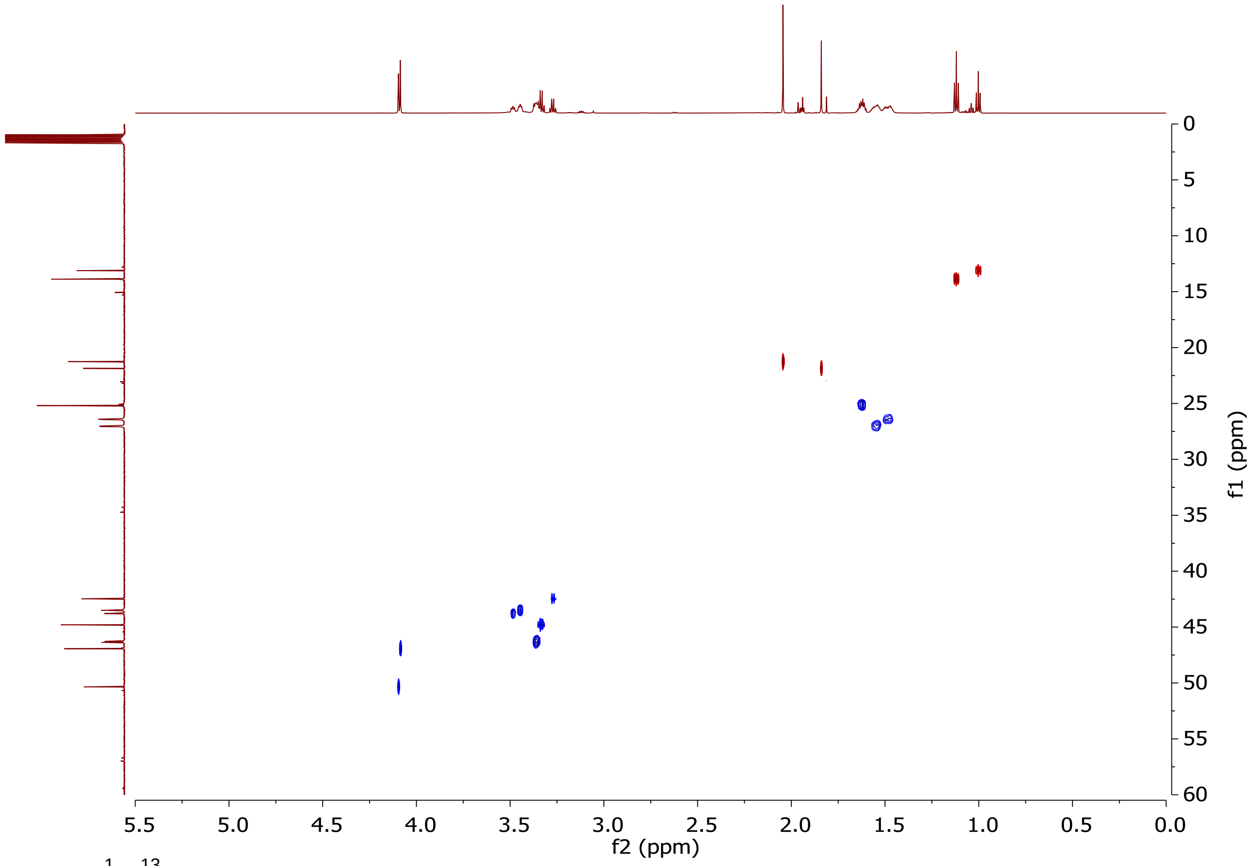

Figure S-7. ^{1}H-^{13}C HSQC spectrum of compound **10** recorded in *acetonitrile-d_3* at room temperature

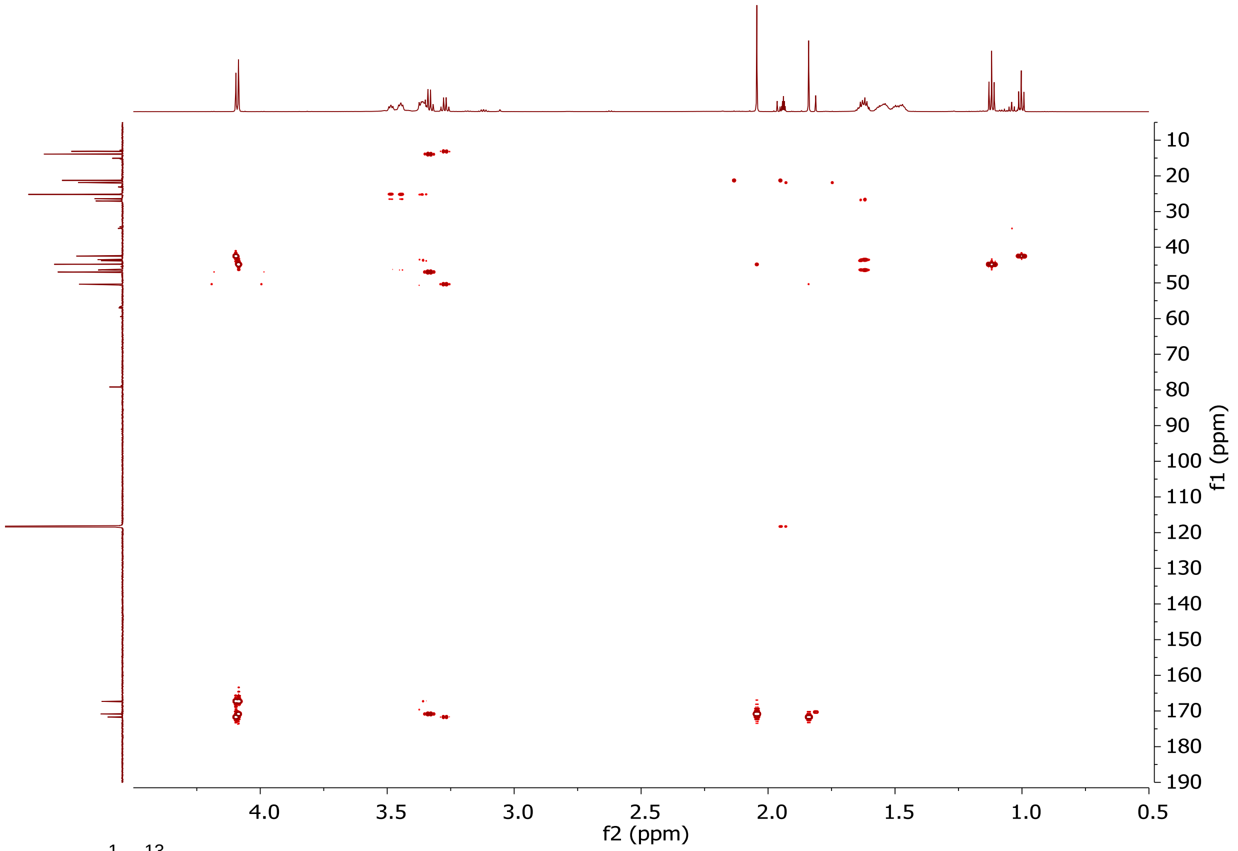

Figure S-8. ^{1}H-^{13}C HMBC spectrum of compound **10** recorded in *acetonitrile-d$_3$* at room temperature.

^{1}H NMR (600 MHz, Methanol-*d$_4$*) Rotamer *trans*: δ ^{1}H NMR: 4.21 (s, 2H, $H_{4,trans}$), 3.57-3.45 (m, 4H, $H_{3,ax+eq,trans}$), 3.43 (q, *J* = 7.2 Hz, 2H, $H_{5,trans}$), 2.16 (s, 3H, $H_{6,trans}$), 1.76-1.52 (m, 6H, $H_{1\text{-}2,ax+eq,trans}$), 1.19 (t, *J*=7.2 Hz, 3H, $H_{7,trans}$); Rotamer *cis*: δ ^{1}H NMR: 4.30 (s, 2H, $H_{4,cis}$), 3.57-3.45 (m, 4H, $H_{3,ax+eq,cis}$), 3.37 (q, *J* = 7.3 Hz, 2H, $H_{5,cis}$), 1.96 (s, 3H, $H_{6,cis}$), 1.76-1.52 (m, 6H, $H_{1\text{-}2,\ ax+eq,cis}$), 1.09 (t, *J*=7.2 Hz, 3H, $H_{7,cis}$).
^{13}C NMR (151 MHz, Methanol-*d$_4$*) Rotamer *trans*: δ ^{13}C NMR: 47.65 (CH_2, $C_{4,trans}$), 46.99, 44.34 (CH_2, $C_{3,trans}$), 45.84 (CH_2, $C_{5,trans}$), 27.25, 26.63, 25.40 (CH_2, $C_{1\text{-}2,trans}$), 20.88 (CH_3, $C_{6,trans}$), 13.67 (CH_3, $C_{7,trans}$); Rotamer *cis*: δ ^{13}C NMR: 50.87 (CH_2, $C_{4,cis}$), 46.84, 44.52 (CH_2, $C_{3,cis}$), 43.65 (CH_2, $C_{5,cis}$), 27.44, 26.74, 25.34 (CH_2, $C_{1\text{-}2,cis}$), 21.49 (CH_3, $C_{6,cis}$), 12.87 (CH_3, $C_{7,cis}$); Quaternary carbons: δ ^{13}C NMR: 174.14 (**C**O-$CH_{3,cis}$), 173.40 (**C**O-$CH_{3,trans}$), 168.30 (**C**O-$pip_{,trans}$), 168.16 (**C**O-$pip_{,cis}$) ppm.

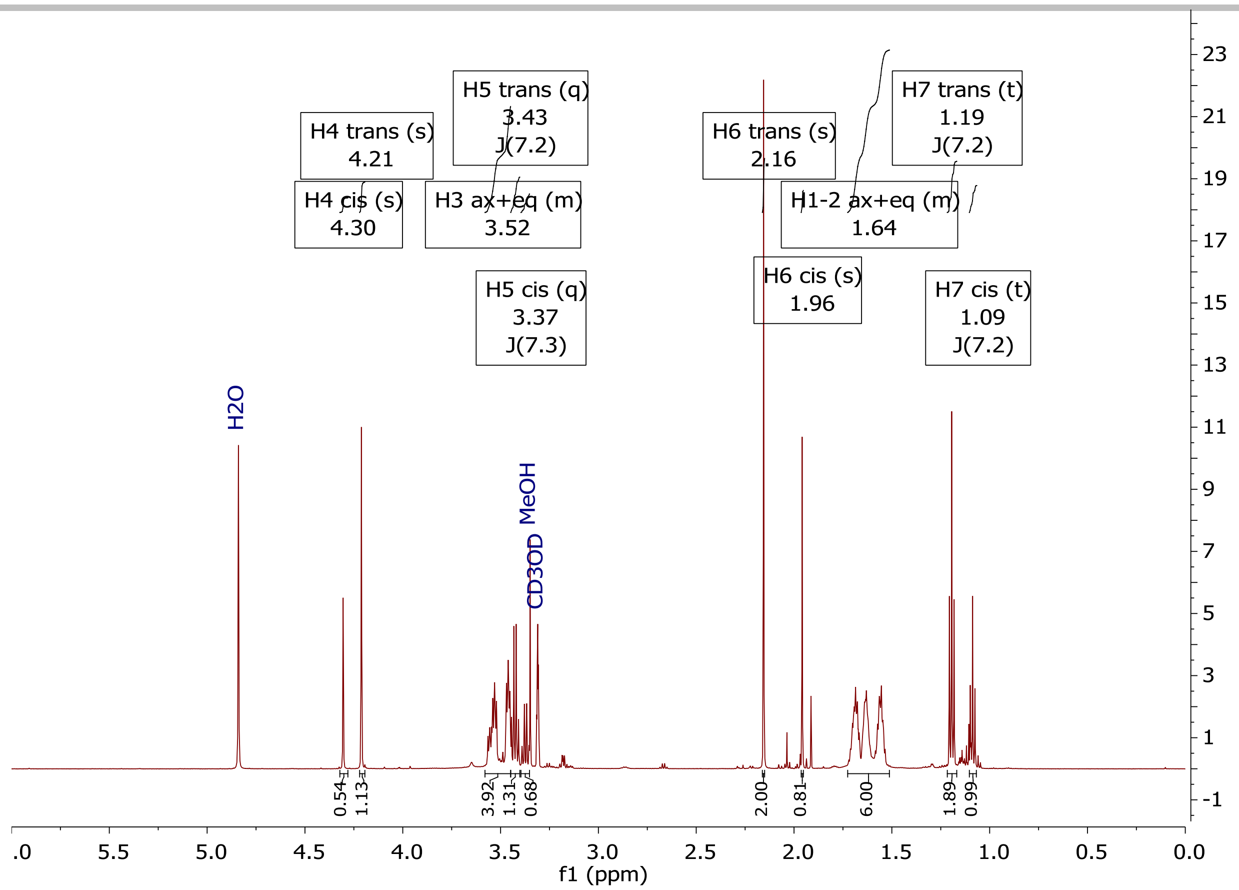

Figure S-9. 1H-NMR spectrum of compound **10** recorded in *methanol-d_4* at room temperature.

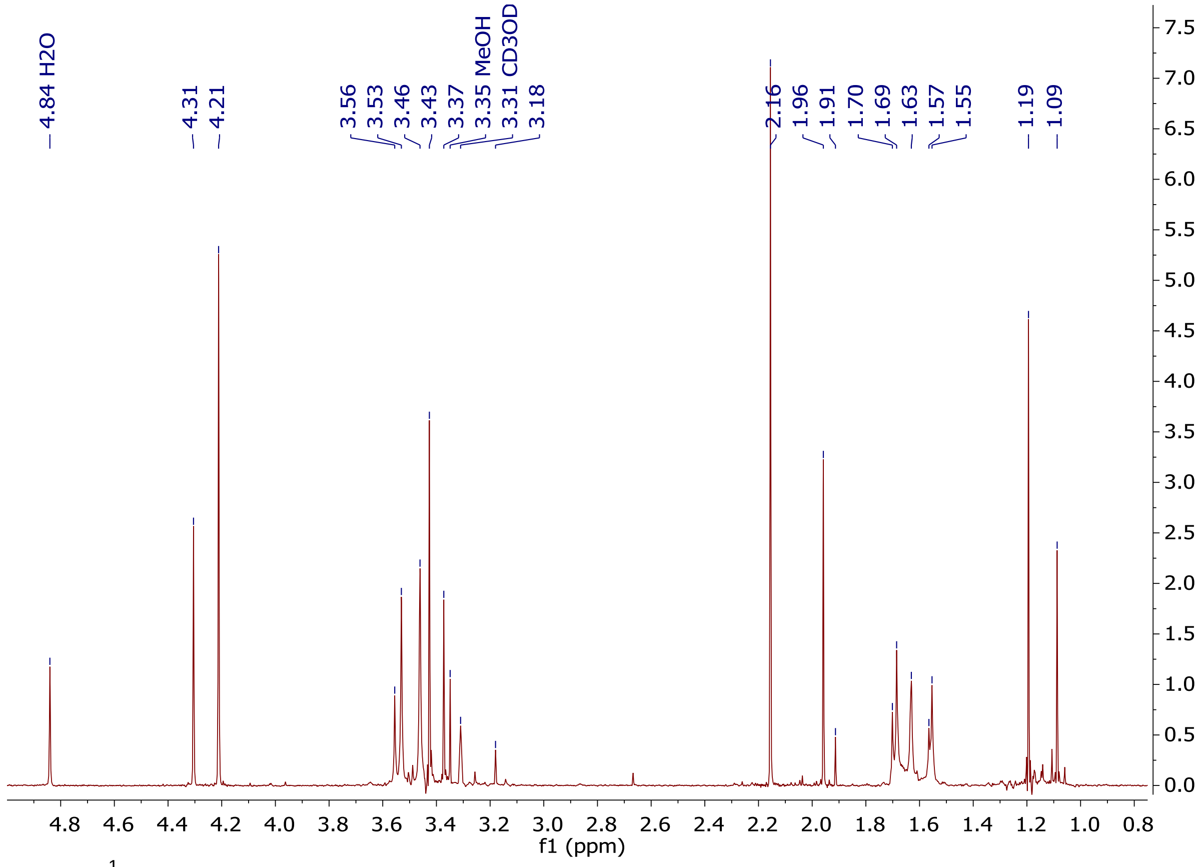

Figure S-10. ^{1}H-*psyche* spectrum of compound **10** recorded in *methanol-d_4* at room temperature.

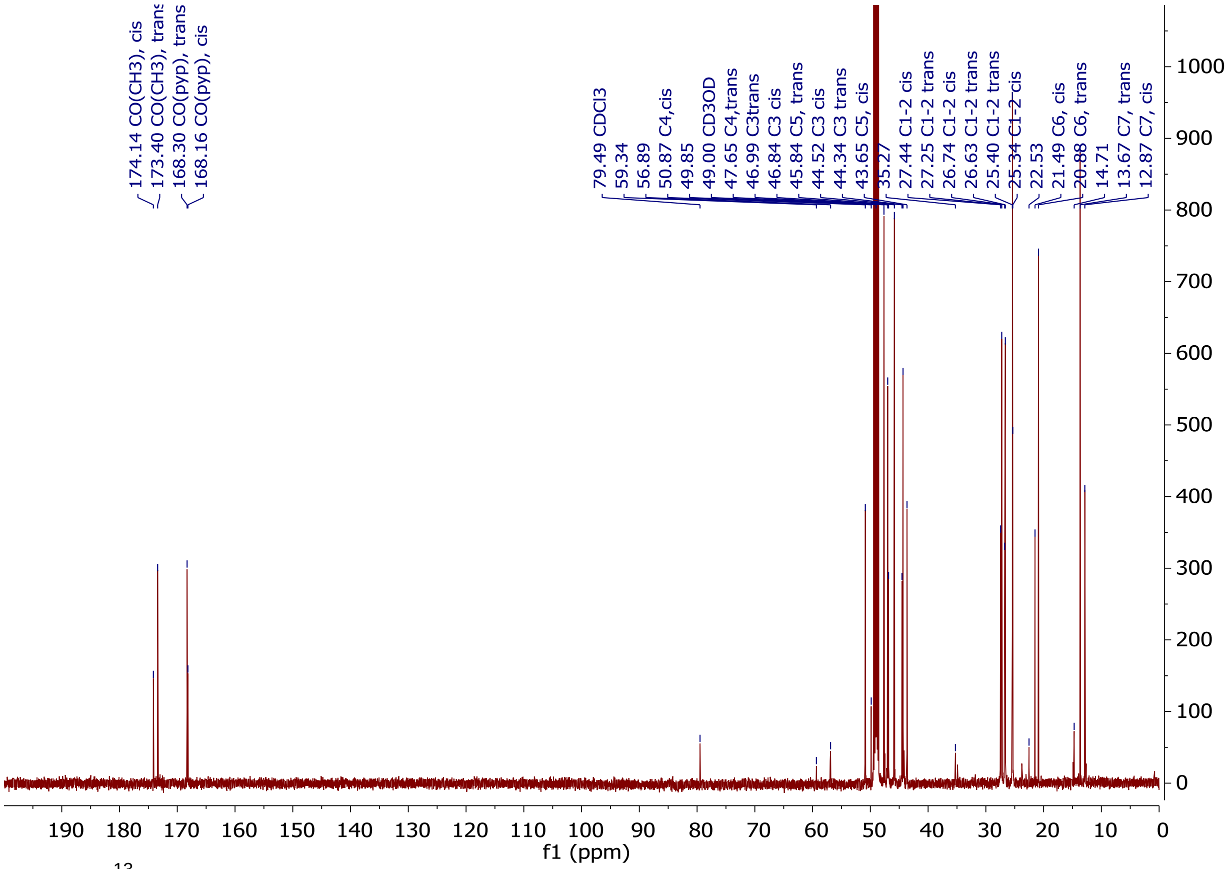

Figure S-11. ^{13}C NMR spectrum of compound **10** recorded in *methanol-d_4* at room temperature.

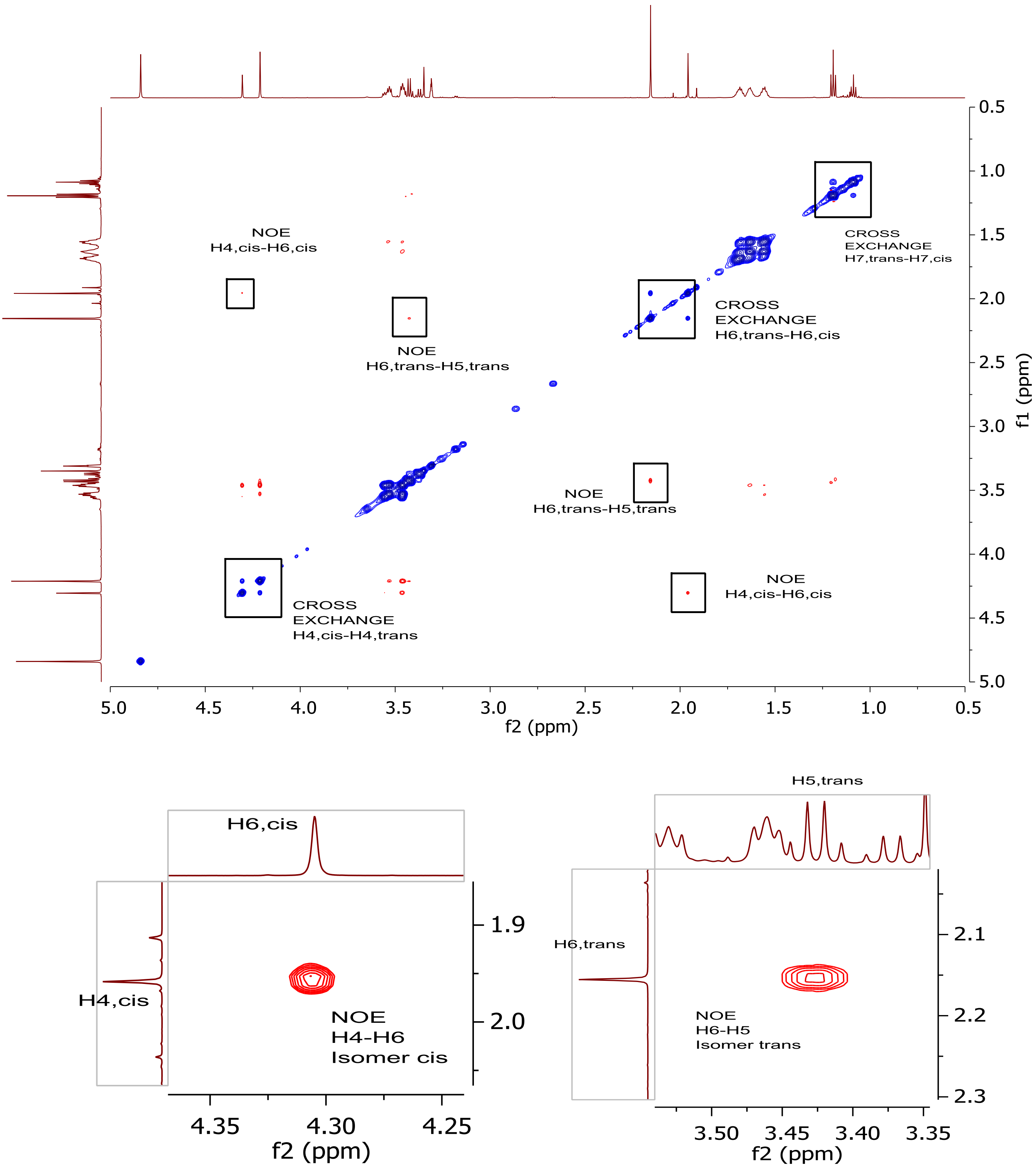

Figure S-12. ^{1}H-^{1}H NOESY spectrum of compound **10** recorded in *methanol-d$_4$* at room temperature. Insets showing the specific spatial contacts that are characteristic of each isomer and allowed the identification of *cis* and *trans* signals.

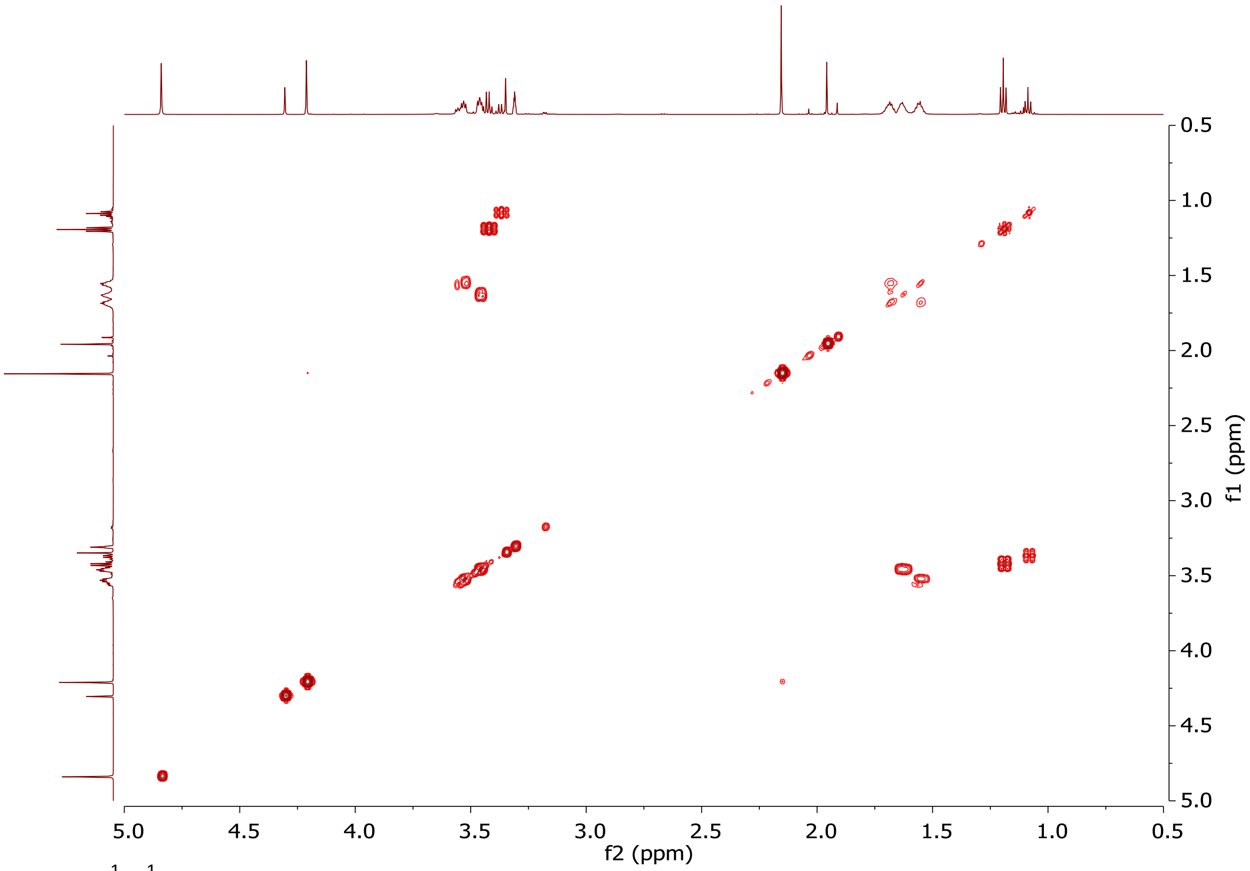

Figure S-13. ^1H-^1H COSY spectrum of compound **10** recorded in *methanol-d_4* at room temperature.

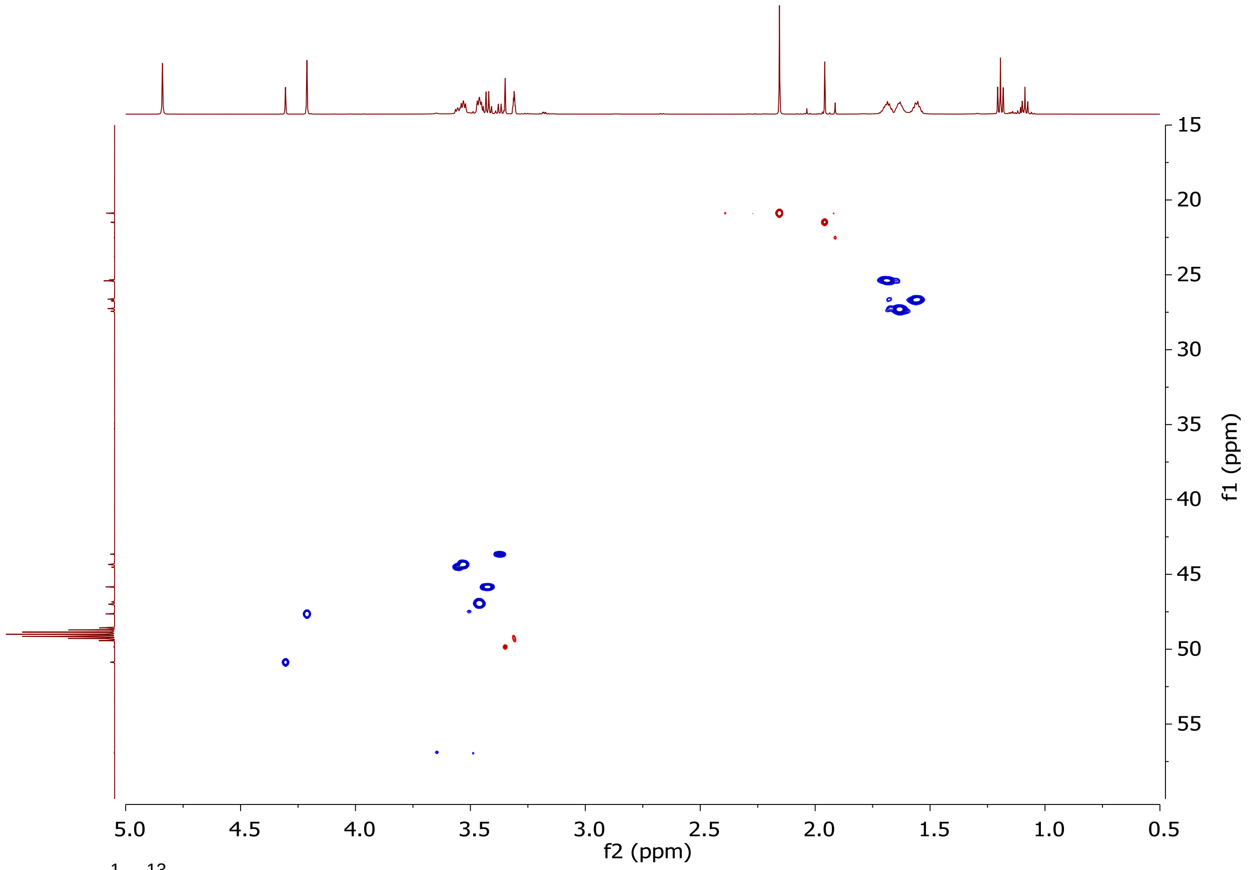

Figure S-14. ^1H-^{13}C HSQC spectrum of compound **10** recorded in *methanol-d_4* at room temperature.

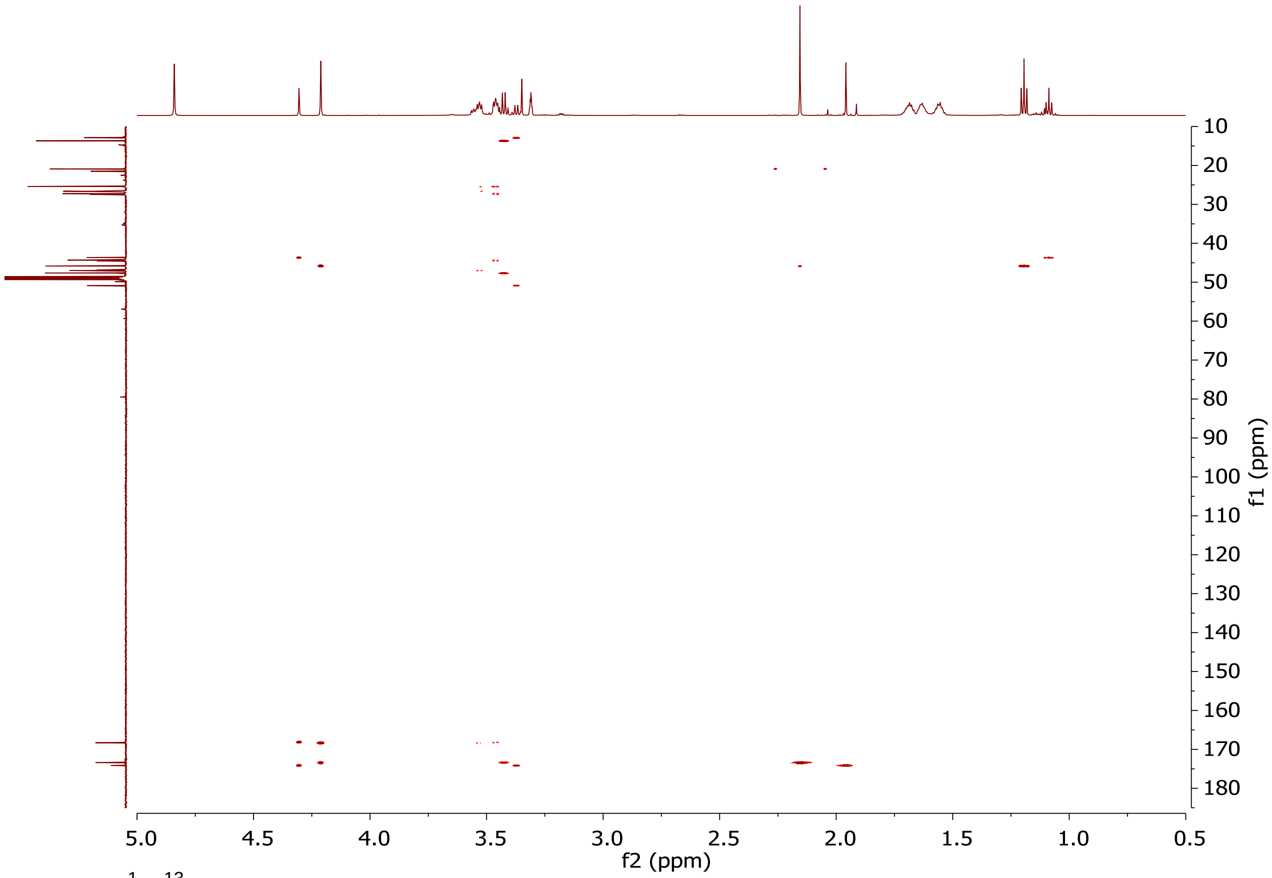

Figure S-15. ^{1}H-^{13}C HMBC spectrum of compound **10** recorded in *methanol-d$_4$* at room temperature.

^{1}H NMR (700MHz, Chloroform-*d*) <u>Rotamer *trans*</u>: δ ^{1}H NMR: 4.13 (s, 2H, $H_{4,trans}$), 3.52-3.48 (m, 2H, $H_{3,eq,trans}$), 3.44-3.38 (m, 2H, $H_{5,trans}$), 3.38-3.34 (m, 2H, $H_{3,ax,trans}$), 2.13 (s, 3H, $H_{6,trans}$), 1.63-1.49 (m, 6H, $H_{1\text{-}2,ax+eq,trans}$), 1.15 (t, *J*=7.2 Hz, 3H, $H_{7,trans}$); <u>Rotamer *cis*</u>: δ ^{1}H NMR: 4.01 (s, 2H, $H_{4,cis}$), 3.52-3.48 (m, 2H, $H_{3,eq,cis}$), 3.44-3.38 (m, 2H, $H_{5,cis}$), 3.38-3.34 (m, 2H, $H_{3,ax,cis}$), 1.96 (s, 3H, $H_{6,cis}$), 1.63-1.49 (m, 6H, $H_{1\text{-}2,ax+eq,cis}$), 1.06 (t, *J*=7.2 Hz, 3H, $H_{7,cis}$).

^{13}C NMR (176 MHz, Chloroform-*d*) <u>Rotamer *trans*</u>: δ ^{13}C NMR: 46.11, 43.18 (CH_2, $C_{3,trans}$); 46.02 (CH_2, $C_{4,trans}$), 44.05 (CH_2, $C_{5,trans}$); 26.37, 25.54, 24.53 (CH_2, $C_{1\text{-}2,trans}$); 21.05 (CH_3, $C_{6,trans}$); 13.6 (CH_3, $C_{7,trans}$); <u>Rotamer *cis*</u>: δ ^{13}C NMR: 49.61 (CH_2, $C_{4,cis}$), 45.84, 43.48 (CH_2, $C_{3,cis}$); 42.17 (CH_2, $C_{5,cis}$); 26.58, 25.62, 24.43 (CH_2, $C_{1\text{-}2,cis}$); 21.67 (CH_3, $C_{6,cis}$); 12.81 (CH_3, $C_{7,cis}$); <u>Quaternary carbons</u>: δ ^{13}C NMR: 171.11, 170.62, 166.51, 165.82.

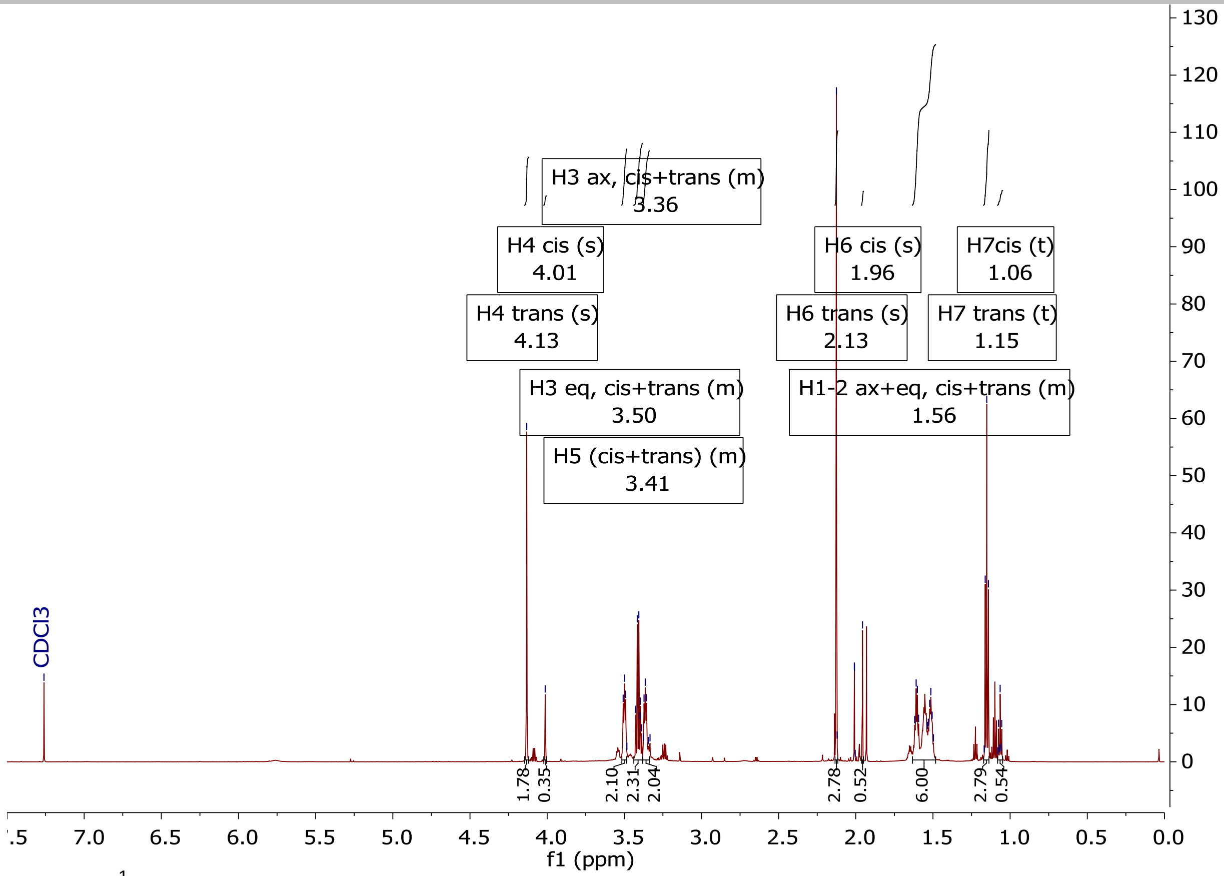

Figure S-16. ^{1}H-NMR spectrum of compound **10** recorded in chloroform-*d* at room temperature.

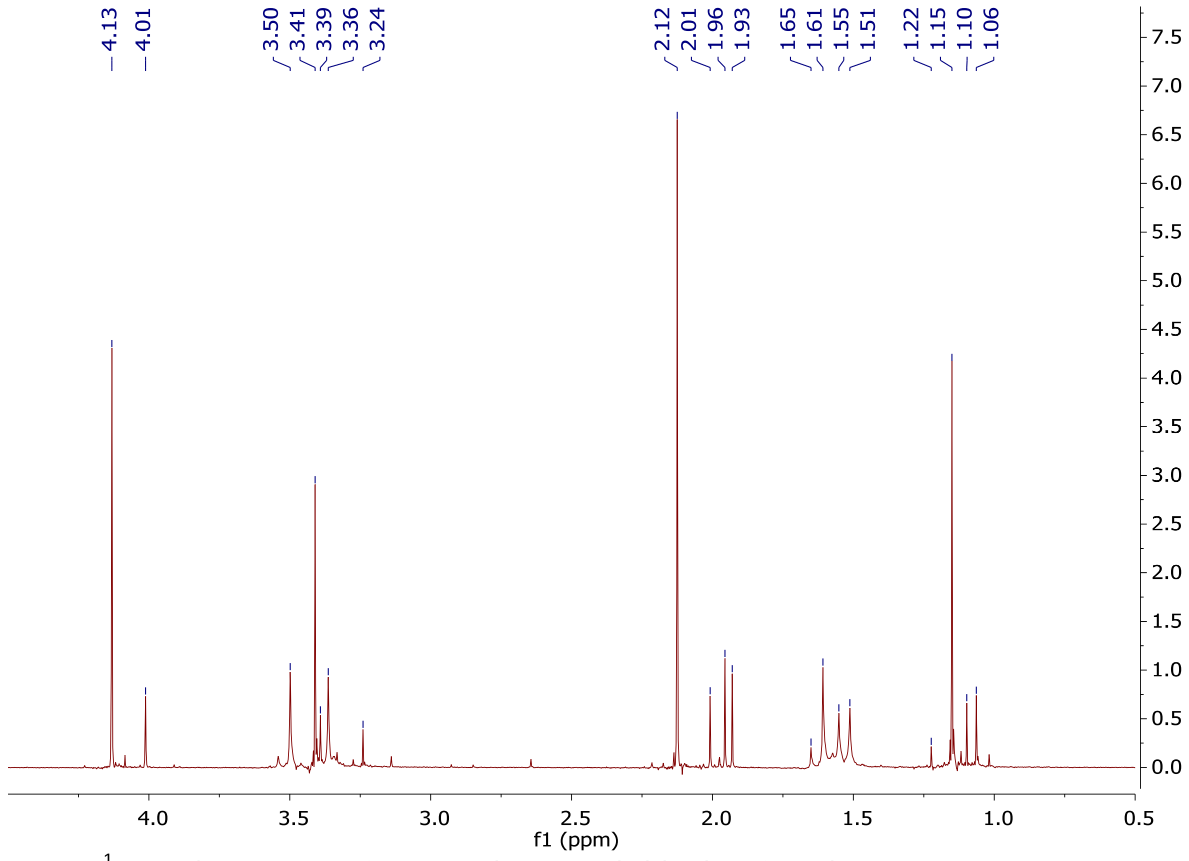

Figure S-17. ^{1}H-*psyche* spectrum of compound **10** recorded in chloroform-*d* at room temperature.

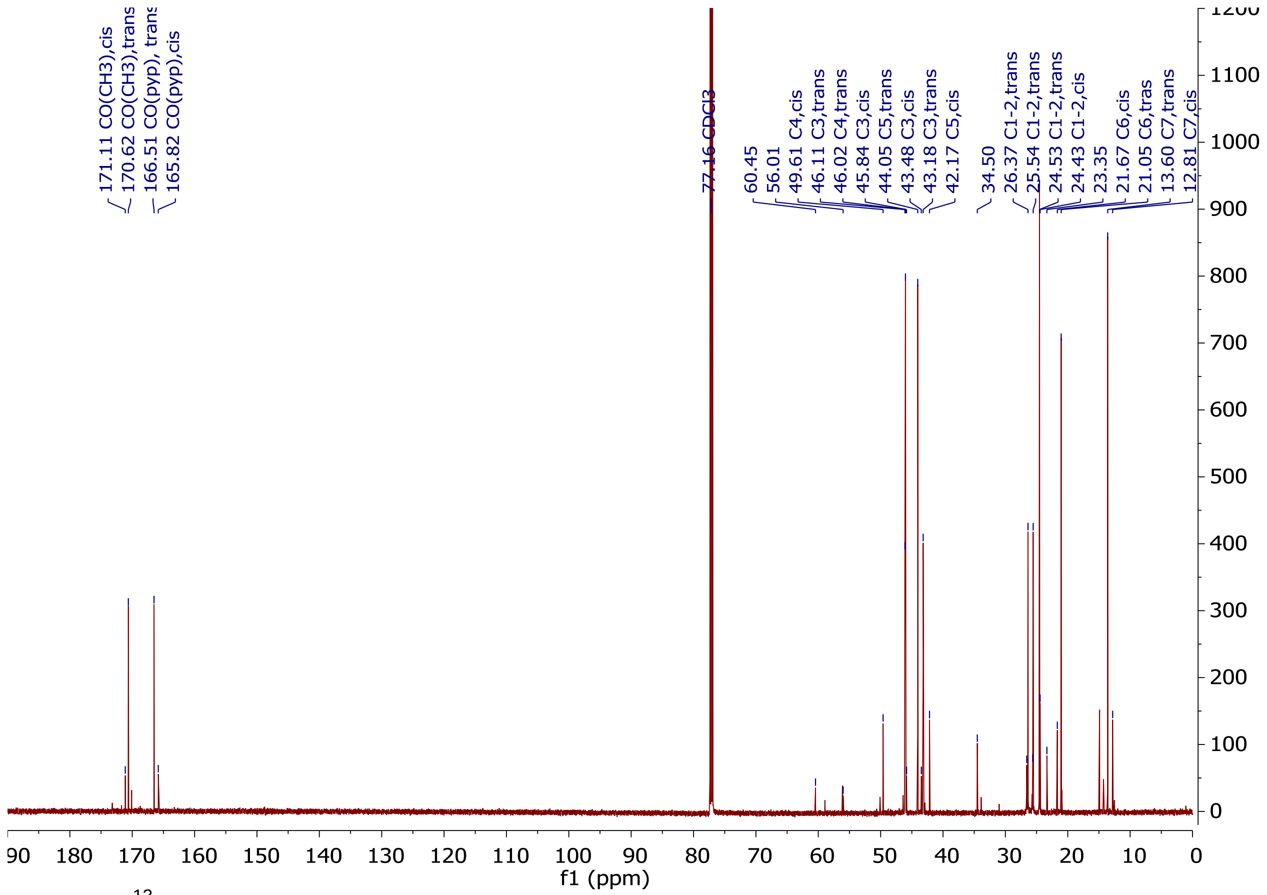

Figure S-18. ^{13}C NMR spectrum of compound **10** recorded in chloroform-*d* at room temperature.

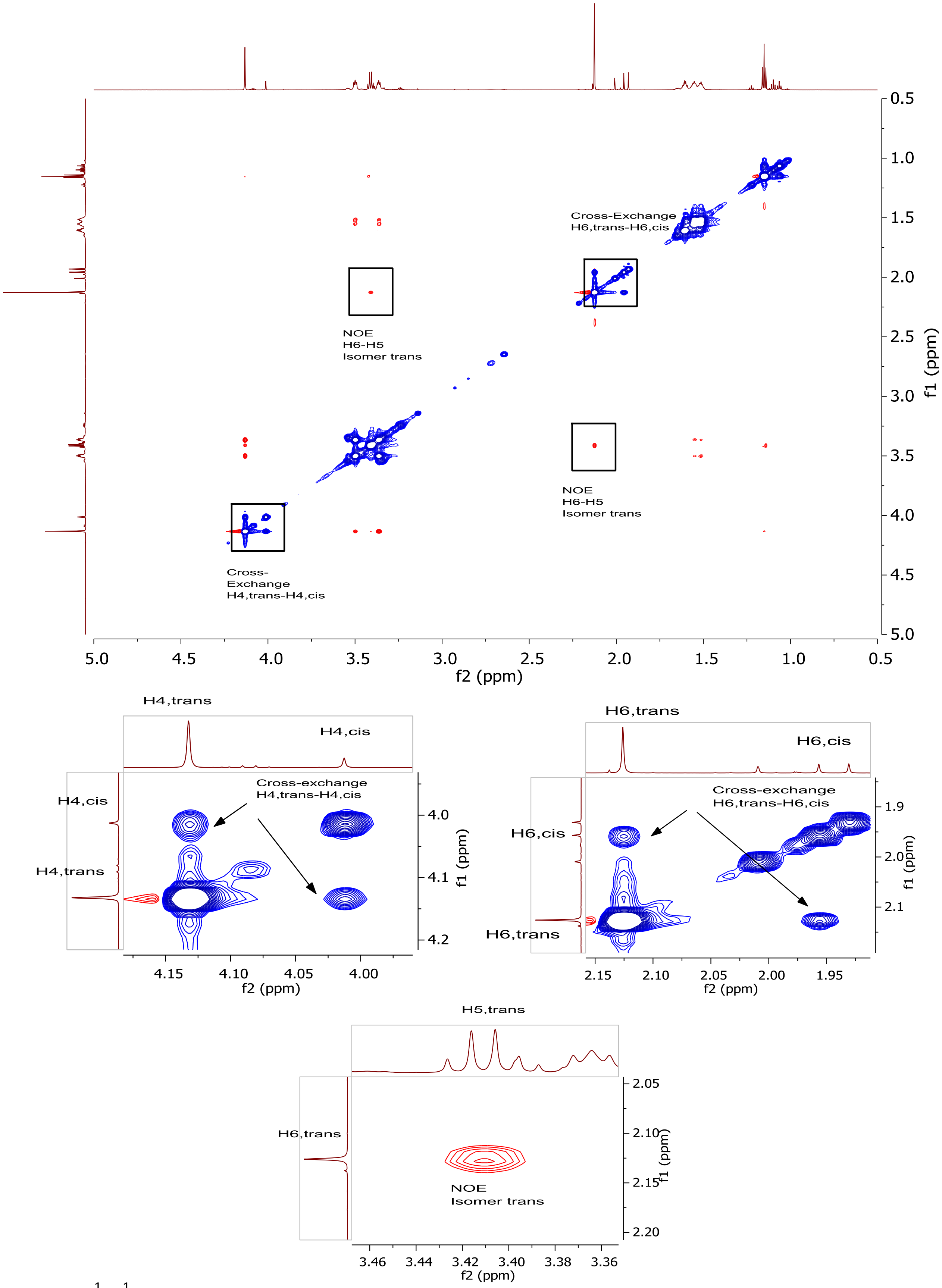

Figure S-19. ^{1}H-^{1}H NOESY spectrum of compound **10** recorded in chloroform-*d* at room temperature. Insets showing the specific spatial contacts that are characteristic of each isomer and allowed the identification of *cis* and *trans* signals.

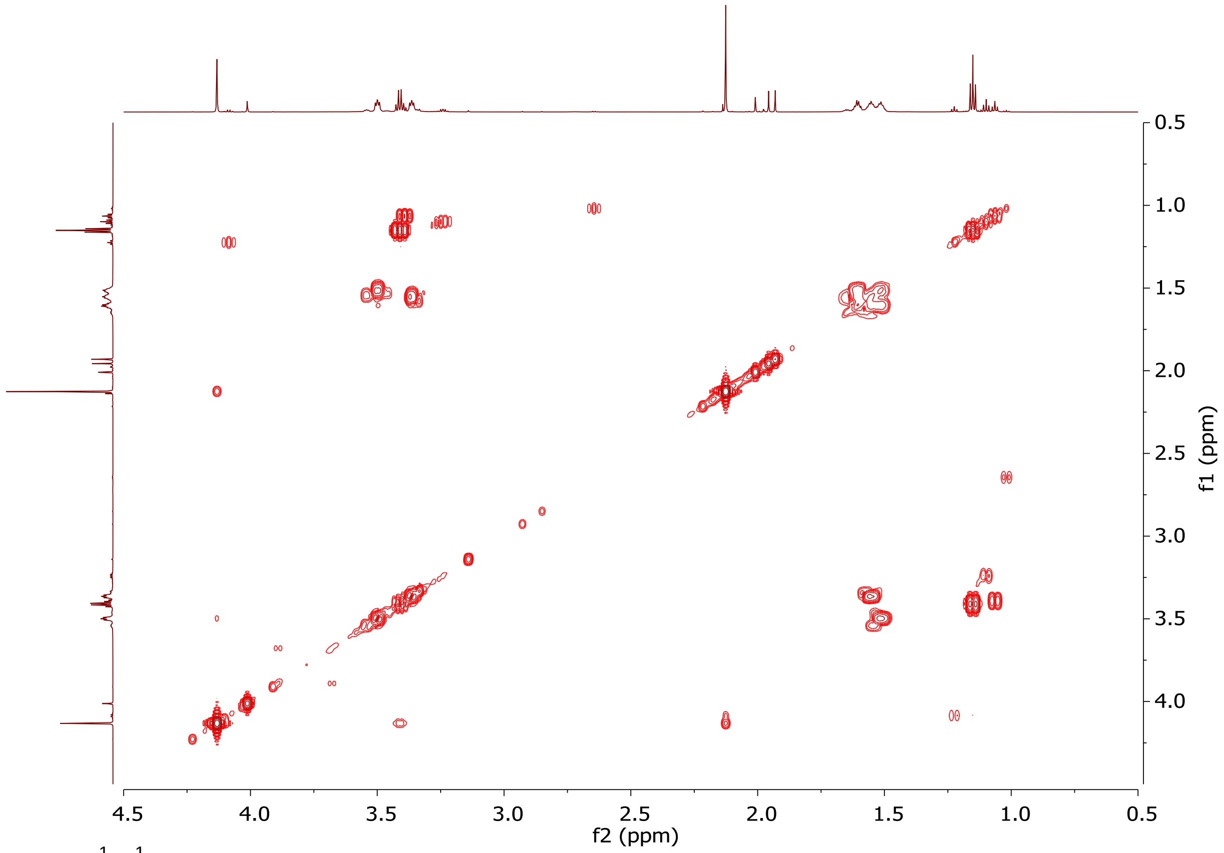

Figure S-20. ^{1}H-^{1}H COSY spectrum of compound **10** recorded in chloroform-*d* at room temperature.

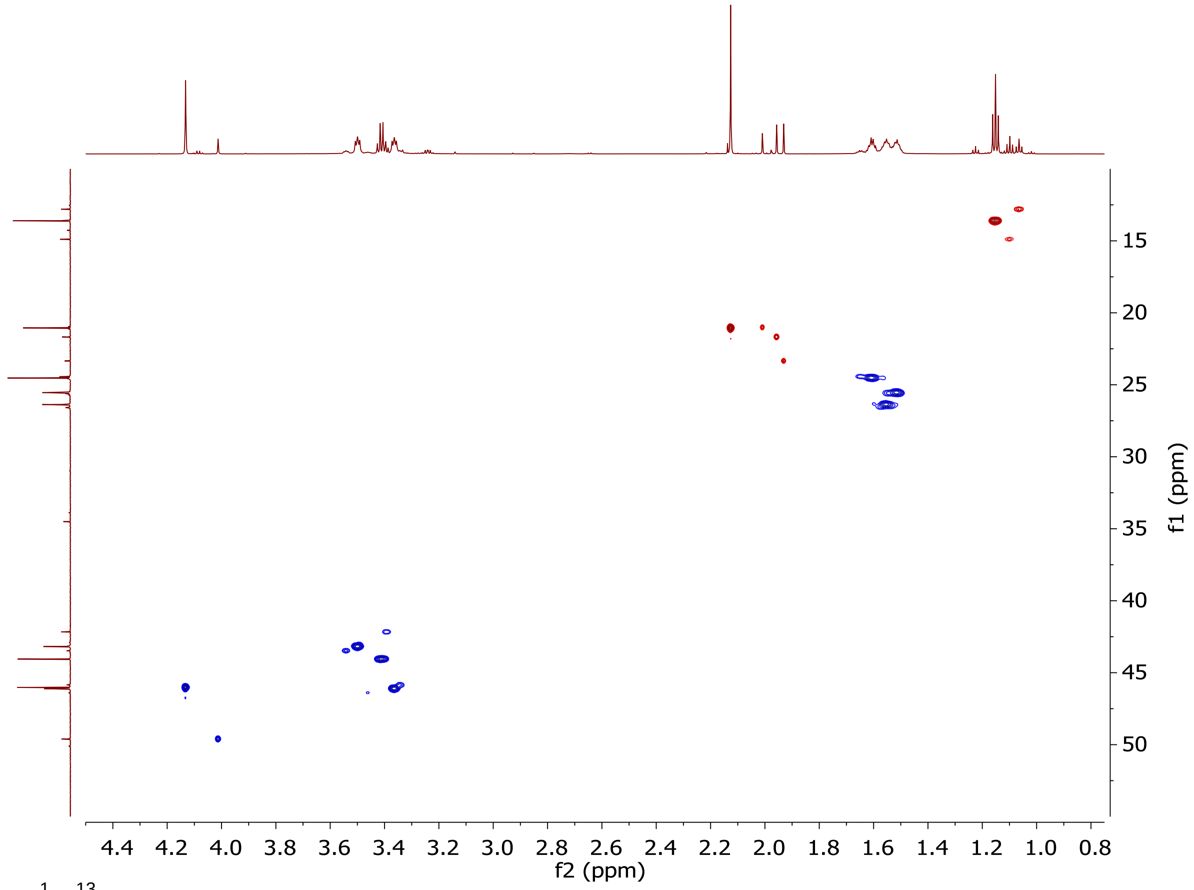

Figure S-21. ^{1}H-^{13}C HSQC spectrum of compound **10** recorded in chloroform-*d* at room temperature.

11: Ac-^{1f}Et-pip.

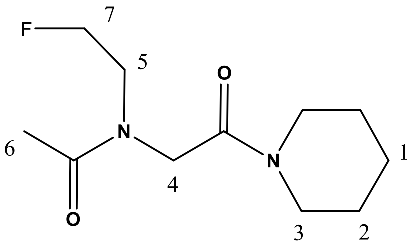

HRMS: calculated for $C_{11}H_{20}FN_2O_2$ 231.1509 [M+H]+, found: 231.1525 Da.

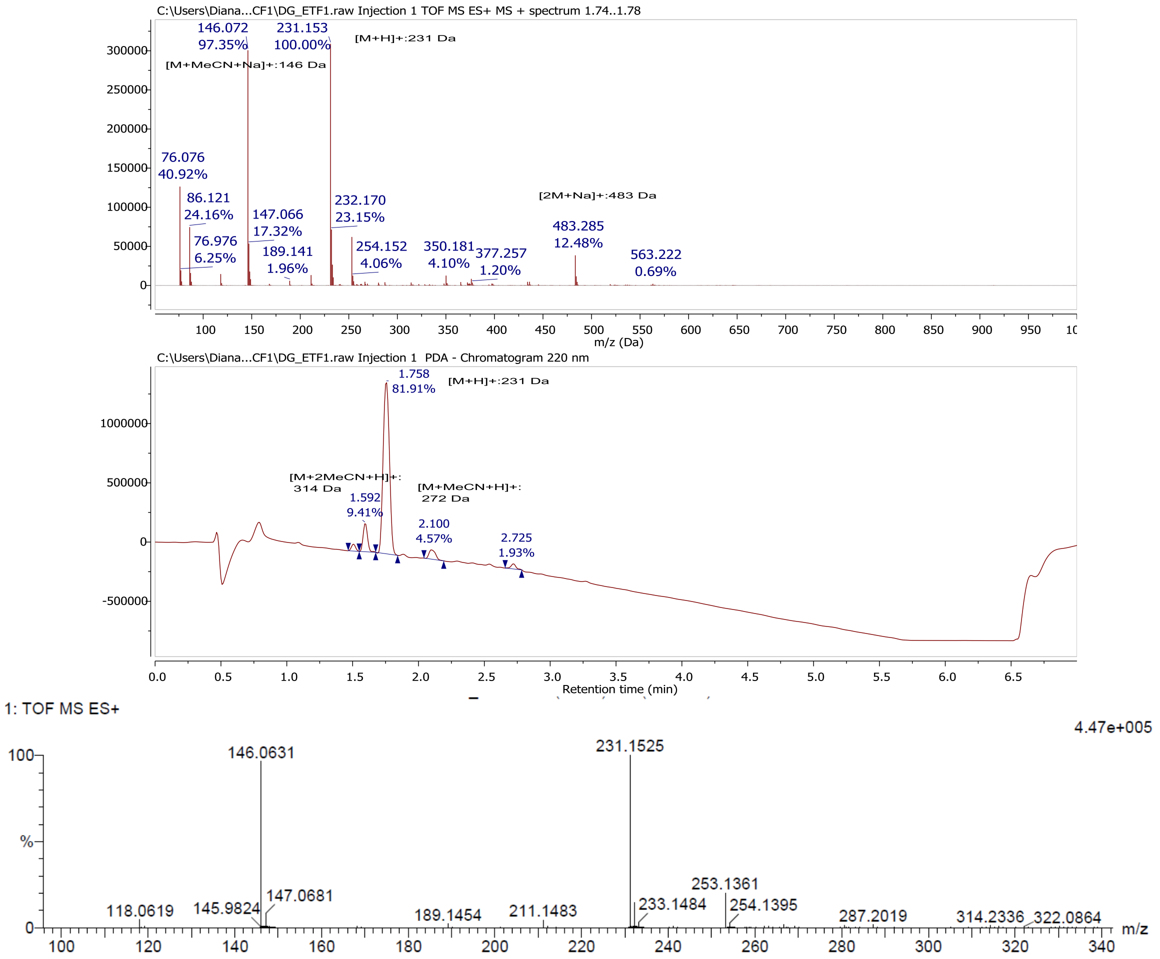

Figure S-22. Structure, HR (QToF)-LC/MS trace and elemental composition of model piperinidyl acetamide **11**.

^{1}H NMR (700MHz, Acetonitrile-*d*$_3$) <u>Rotamer *trans*</u>: δ ^{1}H NMR: 4.55 (dt, *J*= 47.3, 5.0 Hz, 2H, $H_{7,trans}$), 4.15 (s, 2H, $H_{4,trans}$), 3.63 (dt, *J* = 25.9, 5.0 Hz, 2H, $H_{5,trans}$), 3.47-3.34 (m, 4H, $H_{3,ax+eq,trans}$), 2.07 (s, 3H, $H_{6,trans}$), 1.74-1.37 (m, 6H, $H_{1\text{-}2,ax+eq,trans}$); <u>Rotamer *cis*</u>: δ ^{1}H NMR: 4.48 (dt, *J* = 47.5, 5.2 Hz, 2H, $H_{7,cis}$), 4.19 (s, 2H, $H_{4,cis}$), 3.55 (dt, *J* = 25.6, 5.2 Hz, 2H, $H_{5,cis}$), 3.51-3.30 (m, 4H, $H_{3,ax+eq,cis}$), 1.89 (s, 3H, $H_{6,cis}$), 1.74-1.37 (m, 6H, $H_{1\text{-}2,\ ax+eq,cis}$).
^{13}C NMR (176 MHz, Acetonitrile-*d*$_3$) <u>Rotamer *trans*</u>: δ ^{13}C NMR: 83.06 (d, *J* = 166.3 Hz, CH_2, $C_{7,trans}$); 50.31 (d, *J* = 20.3 Hz, CH_2, $C_{5,trans}$), 47.70 (CH_2, $C_{4,trans}$), 46.33, 43.54 (CH_2, $C_{3,trans}$), 26.98, 26.38, 25.16 (CH_2, $C_{1\text{-}2,trans}$), 21.59 (CH_3, $C_{6,trans}$); <u>Rotamer *cis*</u>: δ ^{13}C NMR: 83.46 (d, *J* = 164.7 Hz, CH_2, $C_{7,cis}$), 51.94 (CH_2, $C_{4,cis}$), 48.22 (d, *J* = 21.0 Hz, CH_2, $C_{5,cis}$), 46.26, 43.83 (CH_2, $C_{3,cis}$), 27.01, 26.41, 25.1 (CH_2, $C_{1\text{-}2,cis}$), 21.69(CH_3, $C_{6,cis}$); <u>Quaternary carbons</u>: δ ^{13}C NMR: 172.70 (**C**O-$CH_{3,cis}$), 171.66 (**C**O-$CH_{3,trans}$), 167.02 (**C**O-$pip_{,trans}$), 166.99 (**C**O-$pip_{,cis}$).
^{19}F NMR (376 MHz, Acetonitrile-*d*$_3$) <u>Rotamer *trans*</u>: δ: -223.21 (tt, *J* = 47.3, 25.9 Hz); <u>Rotamer *cis*</u>: δ: -222.72 (tt, *J* = 47.5, 25.7 Hz).

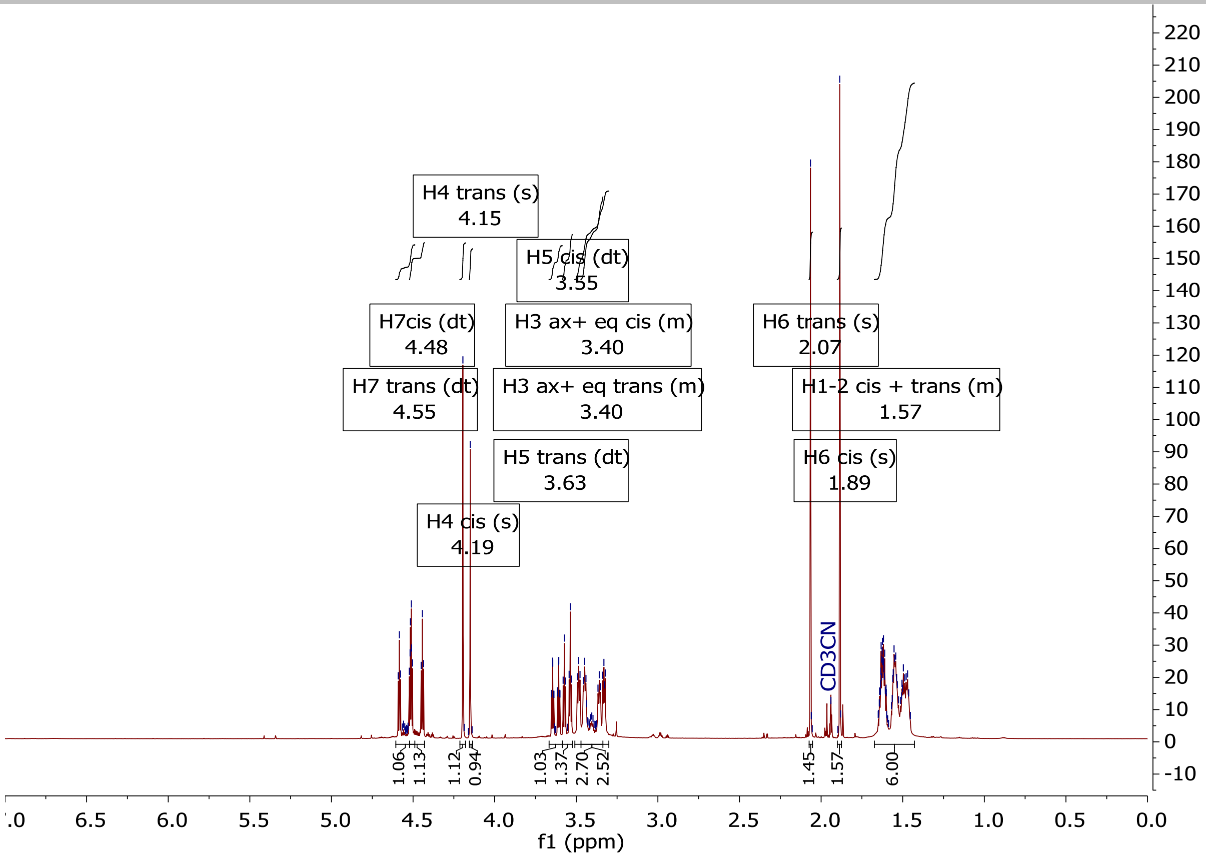

Figure S-23. 1H-NMR spectrum of compound **11** recorded in *acetonitrile-d*$_3$ at room temperature.

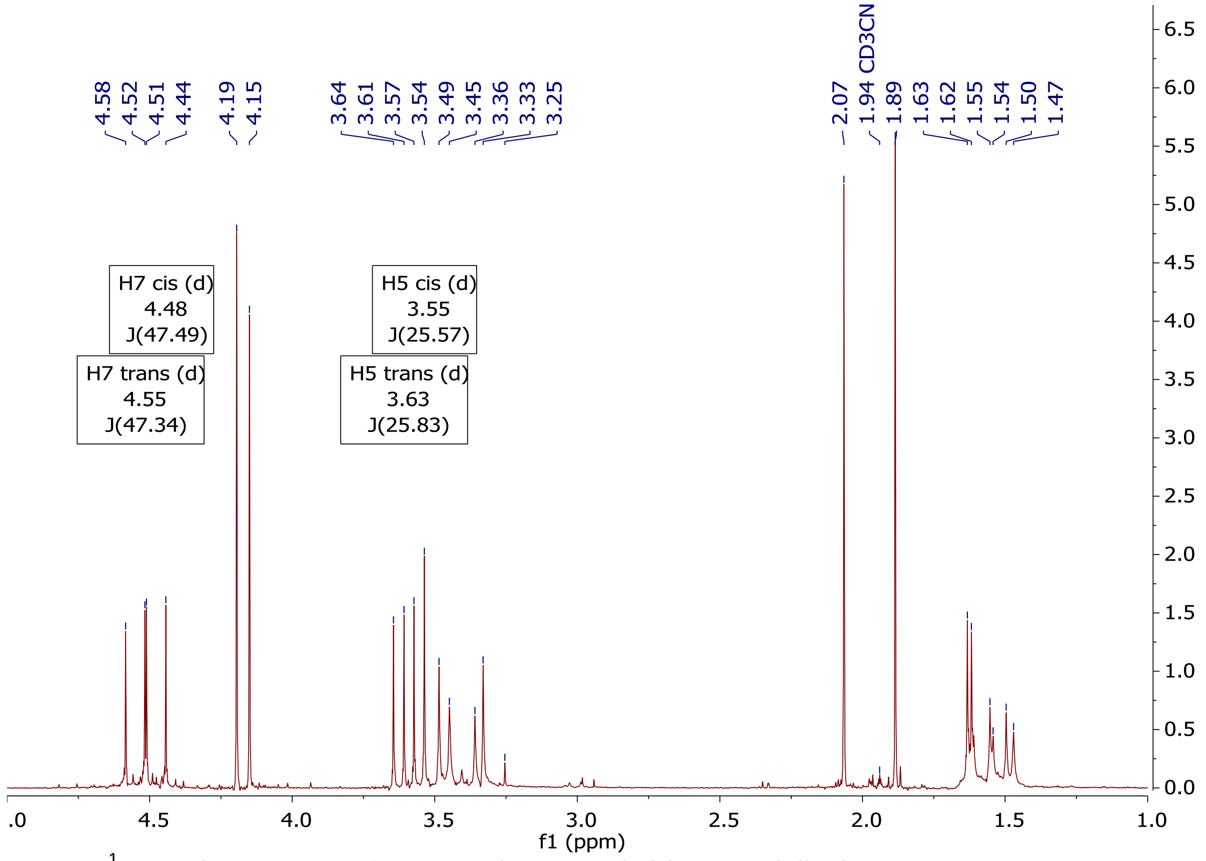

Figure S-24. ^{1}H-*psyche* spectrum of compound **11** recorded in *acetonitrile-d*$_3$ at room temperature.

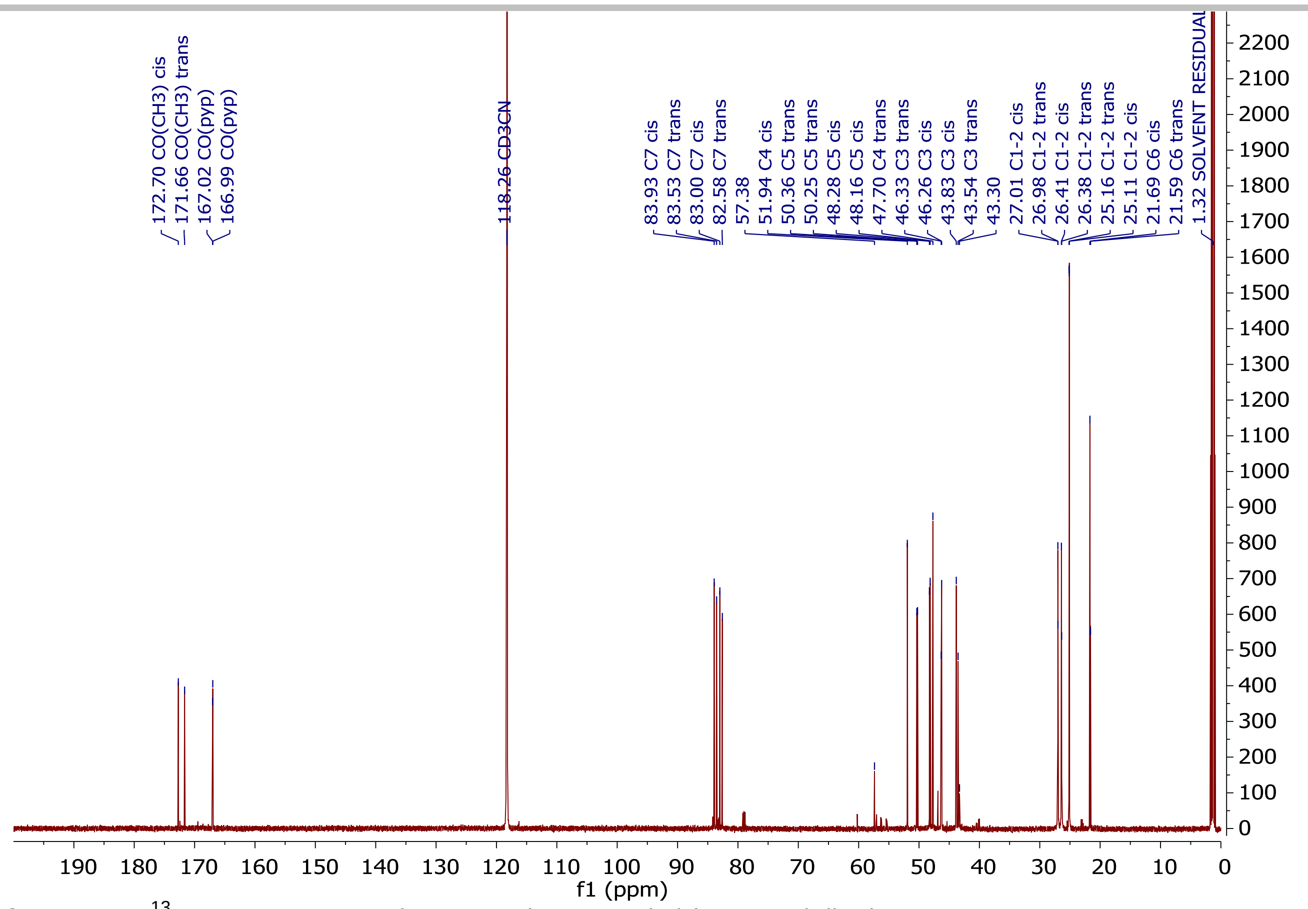

Figure S-25. ^{13}C NMR spectrum of compound **11** recorded in *acetonitrile-d$_3$* at room temperature.

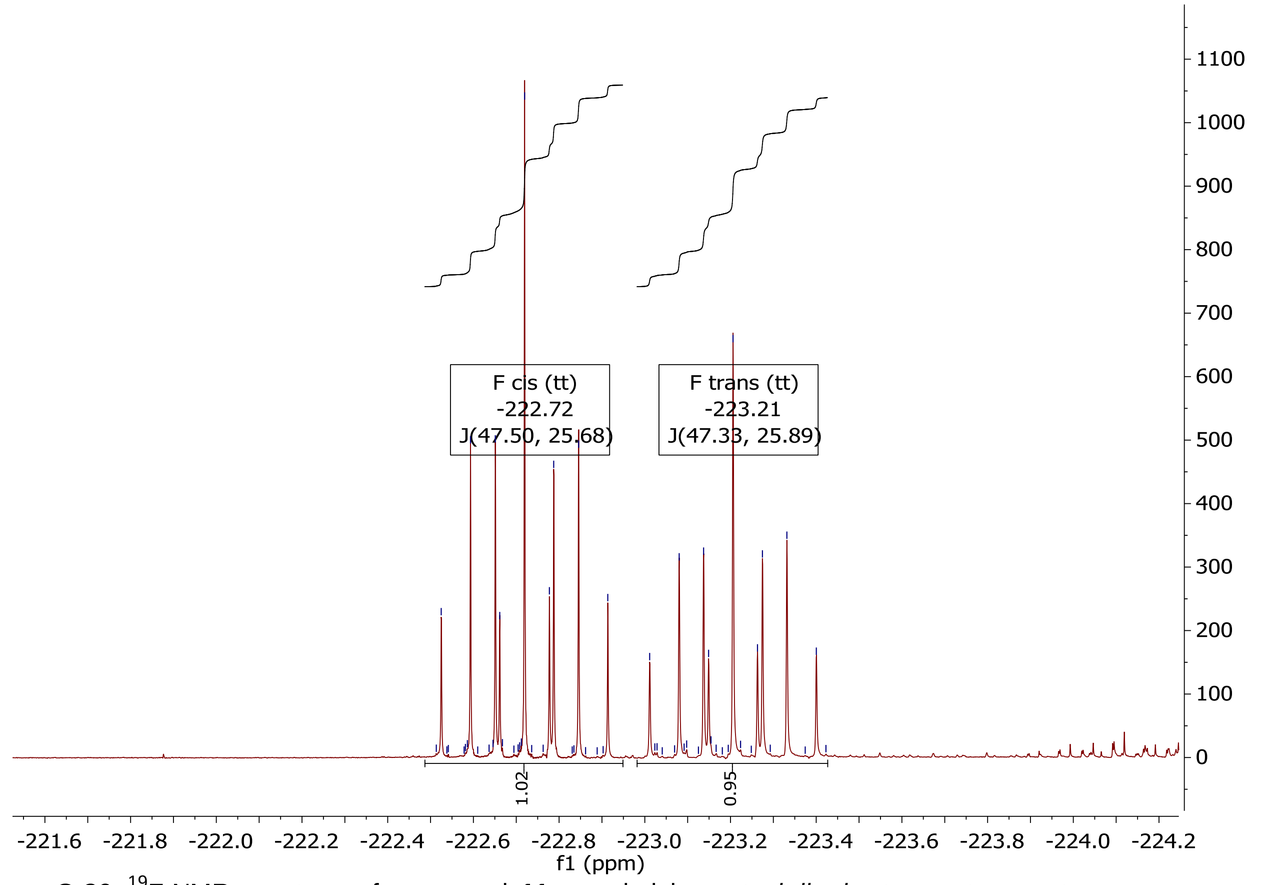

Figure S-26. ^{19}F-NMR spectrum of compound **11** recorded in *acetonitrile-d$_3$* at room temperature.

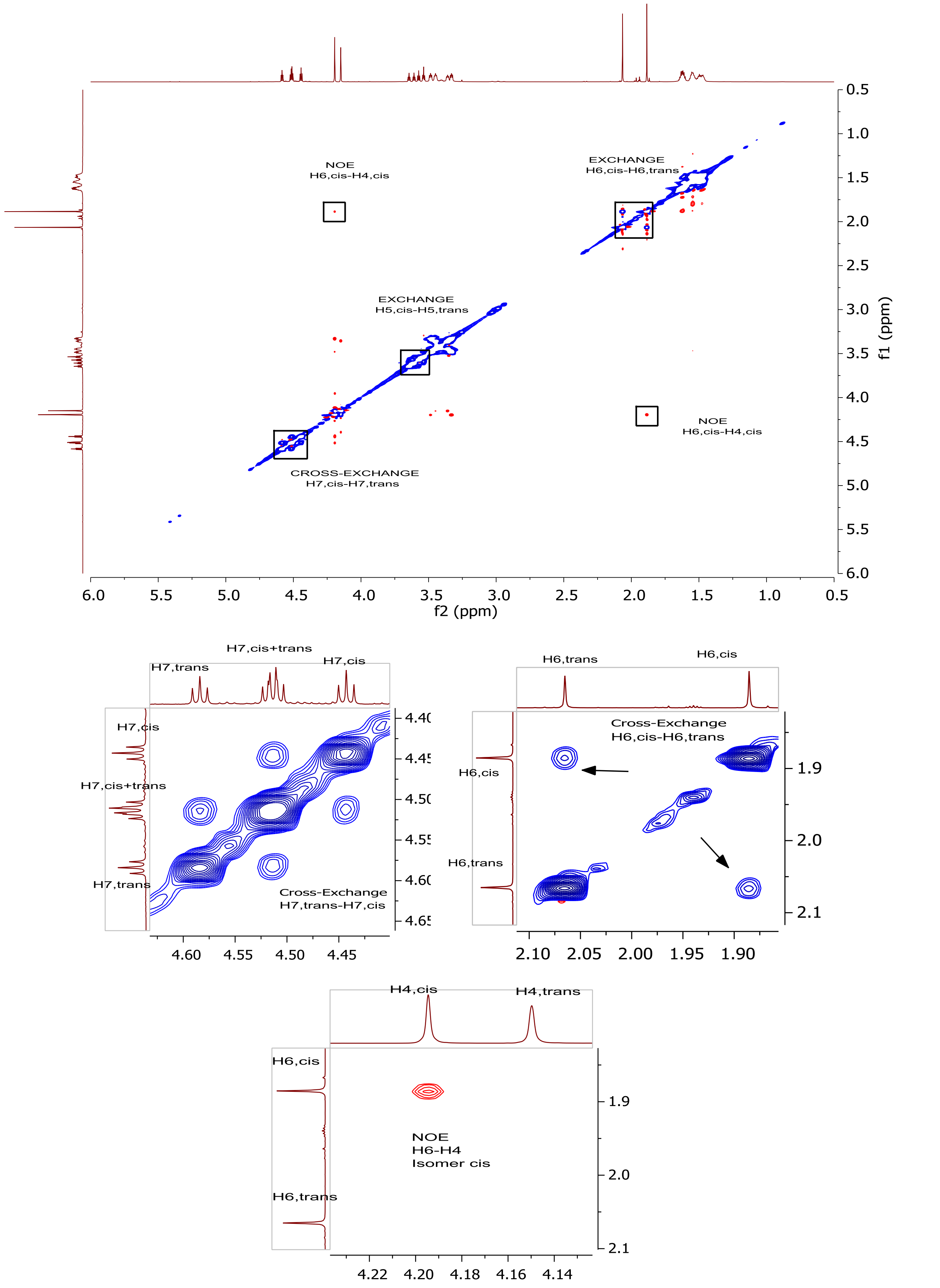

Figure S-27. ^{1}H-^{1}H NOESY spectrum of compound **11** recorded in *acetonitrile-d*$_3$ at room temperature. Insets showing the specific spatial contacts that are characteristic of each isomer and allowed the identification of *cis* and *trans* signals.

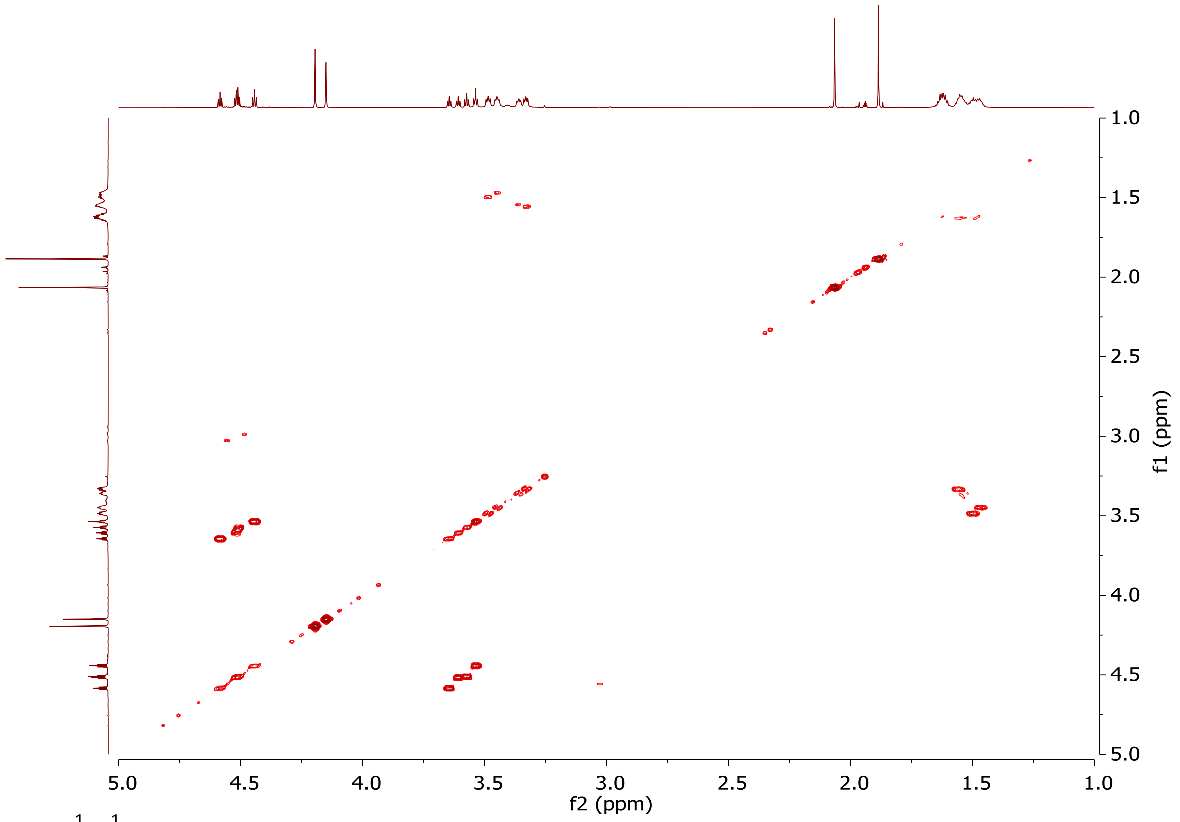

Figure S-28. ^{1}H-^{1}H COSY spectrum of compound **11** recorded in *acetonitrile-d_3* at room temperature.

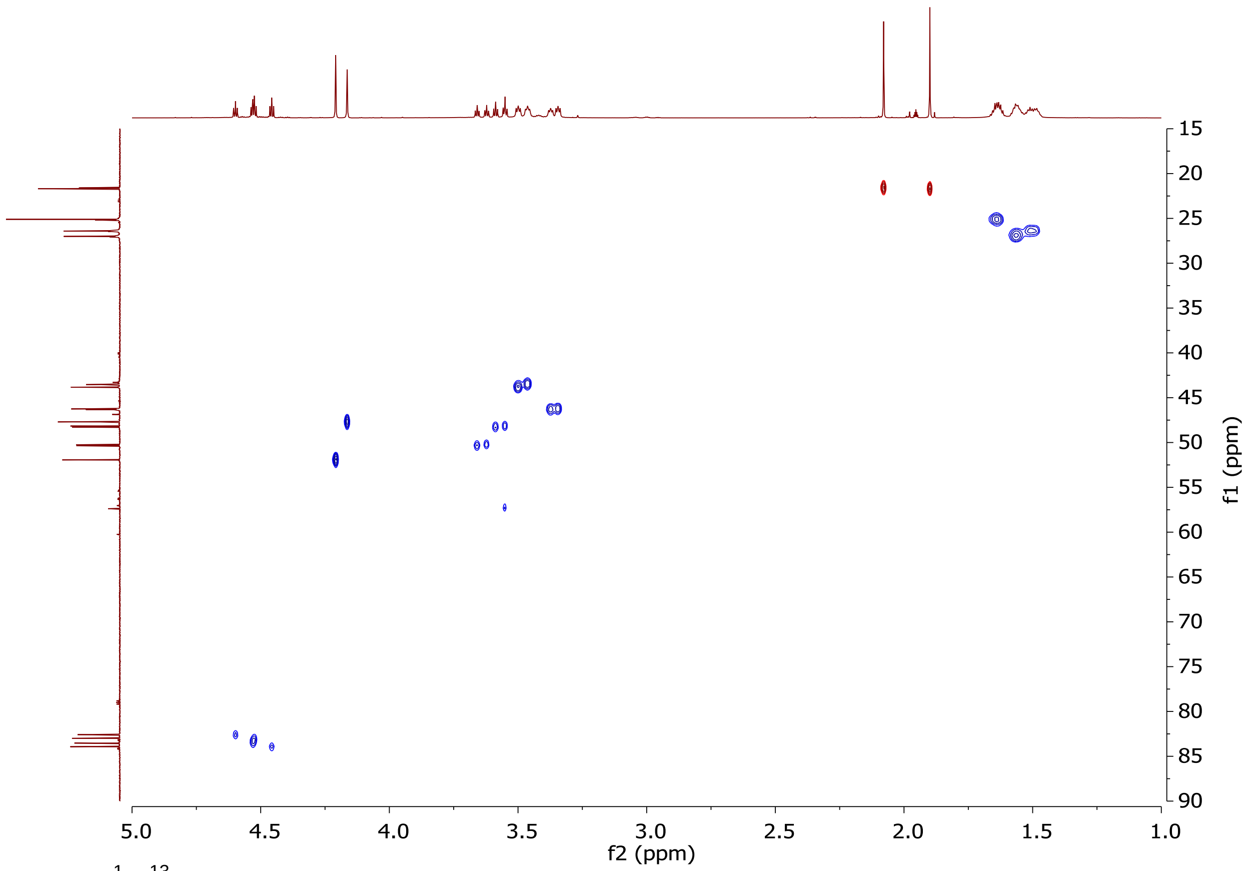

Figure S-29. ^{1}H-^{13}C HSQC spectrum of compound **11** recorded in *acetonitrile-d_3* at room temperature

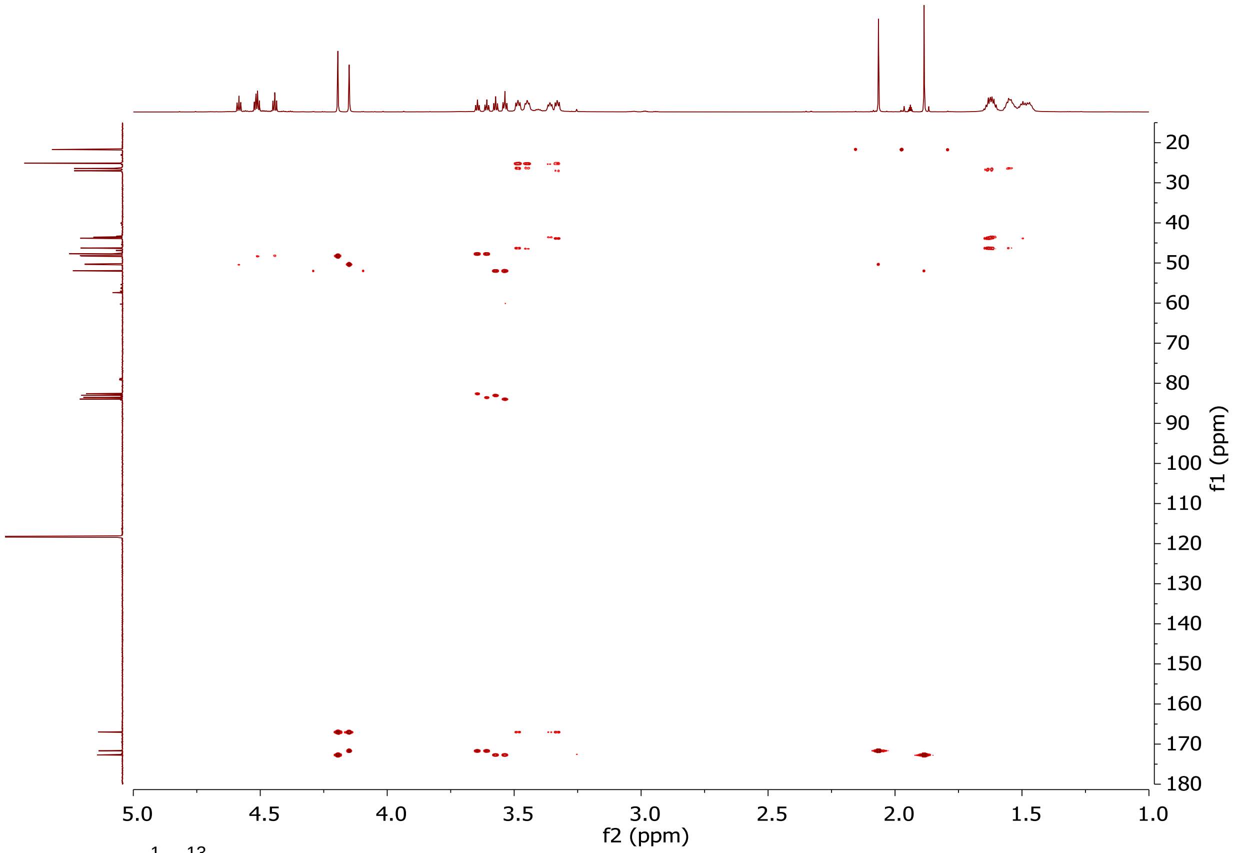

Figure S-30. 2D ^{1}H-^{13}C HMBC spectrum of compound **11** recorded in *acetonitrile-d_3* at room temperature.

^{1}H NMR (700MHz, Methanol-d_4) Rotamer *trans*: δ ^{1}H NMR: 4.60 (dt, J = 47.3, 4.9 Hz, 2H, $H_{7,trans}$), 4.29 (s, 2H, $H_{4,trans}$), 3.72 (dt, J = 26.1, 4.8 Hz, 2H, $H_{5,trans}$), 3.55-3.43 (m, 4H, $H_{3,ax+eq,trans}$), 2.18 (s, 3H, $H_{6,trans}$), 1.74-1.51 (m, 6H, $H_{1\text{-}2,ax+eq,trans}$); Rotamer *cis*: δ ^{1}H NMR: 4.53 (dt, J = 47.5, 5.0 Hz, 2H, $H_{7,cis}$), 4.40 (s, 2H, $H_{4,cis}$), 3.65 (dt, J = 26.1, 5.0 Hz, 2H, $H_{5,cis}$), 3.58, 3.42 (m, 4H, $H_{3,ax+eq,cis}$), 2.00 (s, 3H, $H_{6,cis}$), 1.74-1.51(m, 6H, $H_{1\text{-}2,ax+eq,cis}$).
^{13}C NMR (176 MHz, Methanol-d_4) Rotamer *trans*: δ ^{13}C NMR: 82.95 (d, J = 167.8 Hz, CH_2, $C_{7,trans}$); 51.18 (d, J = 20.0 Hz, CH_2, $C_{5,trans}$), 48.29 (CH_2, $C_{4,trans}$), 46.96, 44.34 (CH_2, $C_{3,trans}$), 27.22, 26.61, 25.39 (CH_2, $C_{1\text{-}2,trans}$), 21.34 (CH_3, $C_{6,trans}$); Rotamer *cis*: δ ^{13}C NMR: 83.51 (d, J = 166.0 Hz, CH_2, $C_{7,cis}$), 52.46 (CH_2, $C_{4,cis}$), 49.16 (d, J = 20.6 Hz, CH_2, $C_{5,cis}$), 46.83, 44.55 (CH_2, $C_{3,cis}$), 27.35, 26.71, 25.33 (CH_2, $C_{1\text{-}2,cis}$), 21.27 (CH_3, $C_{6,cis}$); Quaternary carbons: δ ^{13}C NMR: 174.90 (**C**O-$CH_{3,cis}$), 174.17 (**C**O-$CH_{3,trans}$), 168.07 (**C**O-pip$_{,trans}$), 167.95 (**C**O-pip$_{,cis}$).
^{19}F NMR (376 MHz, Methanol-d_4) Rotamer *trans*: δ: -224.24 (tt, J = 47.4, 26.1 Hz); Rotamer *cis*: δ: -223.51 (tt, J = 47.4, 26.1Hz).

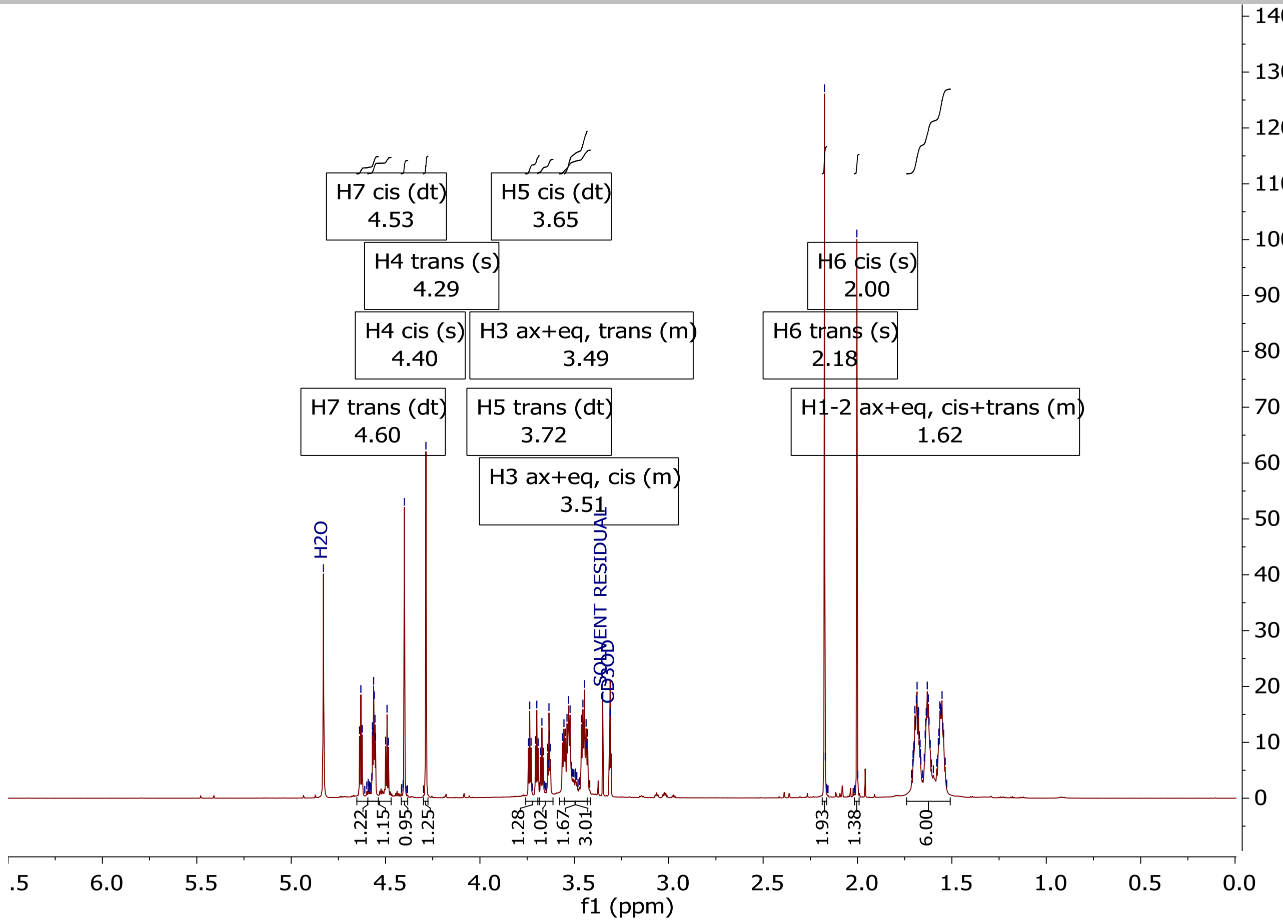

Figure S-31. 1H-NMR spectrum of compound **11** recorded in *methanol-d$_4$* at room temperature.

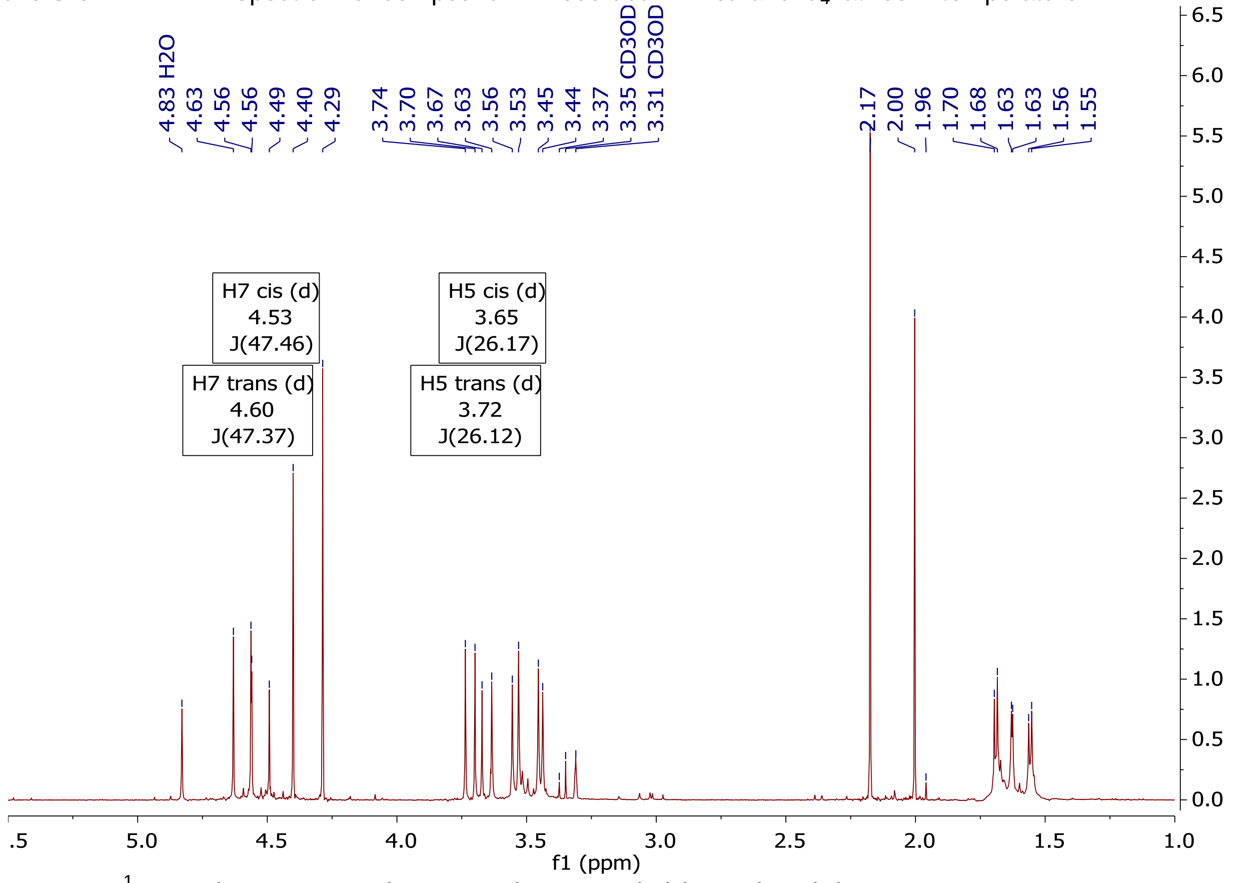

Figure S-32. ^{1}H-*psyche* spectrum of compound **11** recorded in *methanol-d$_4$* at room temperature.

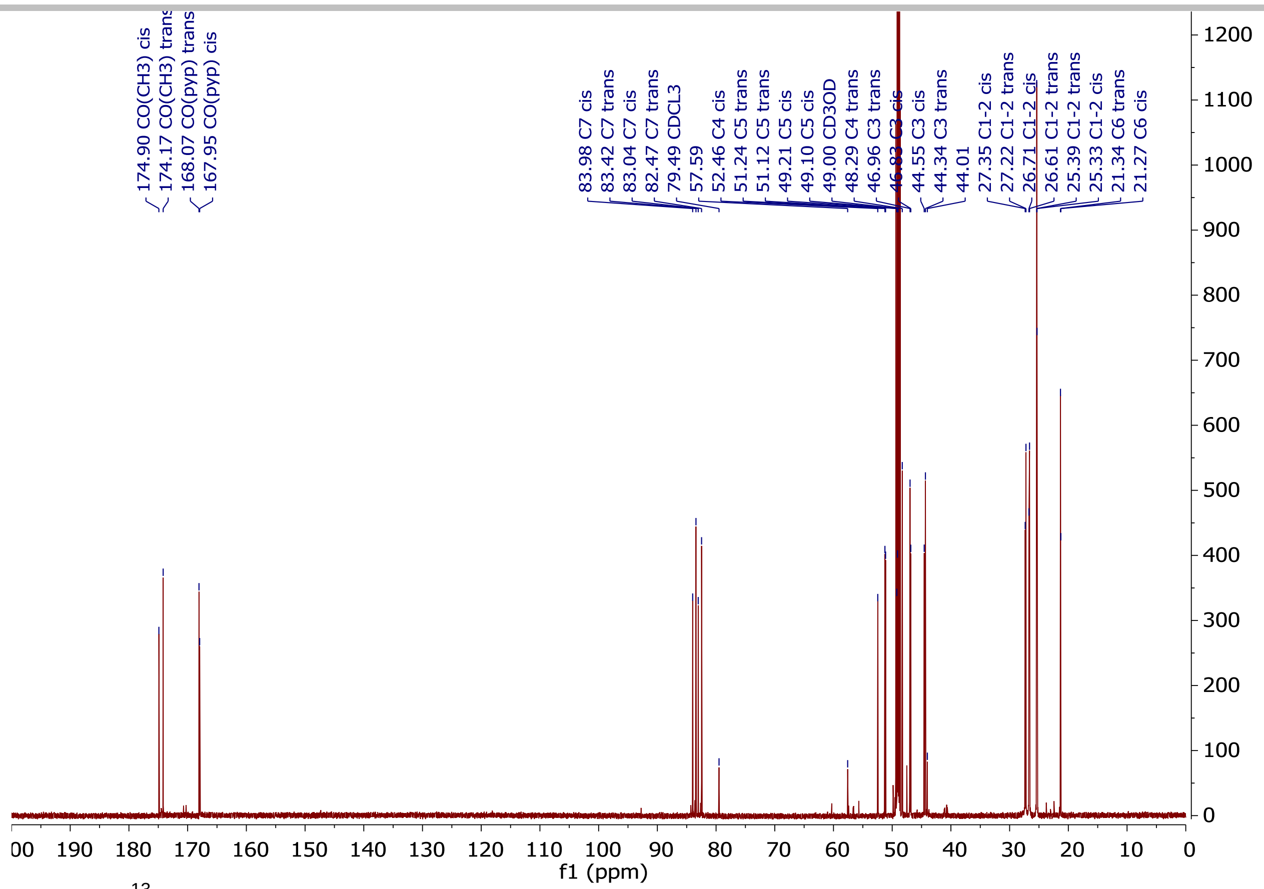

Figure S-33. ^{13}C NMR spectrum of compound **11** recorded in *methanol-d_4* at room temperature.

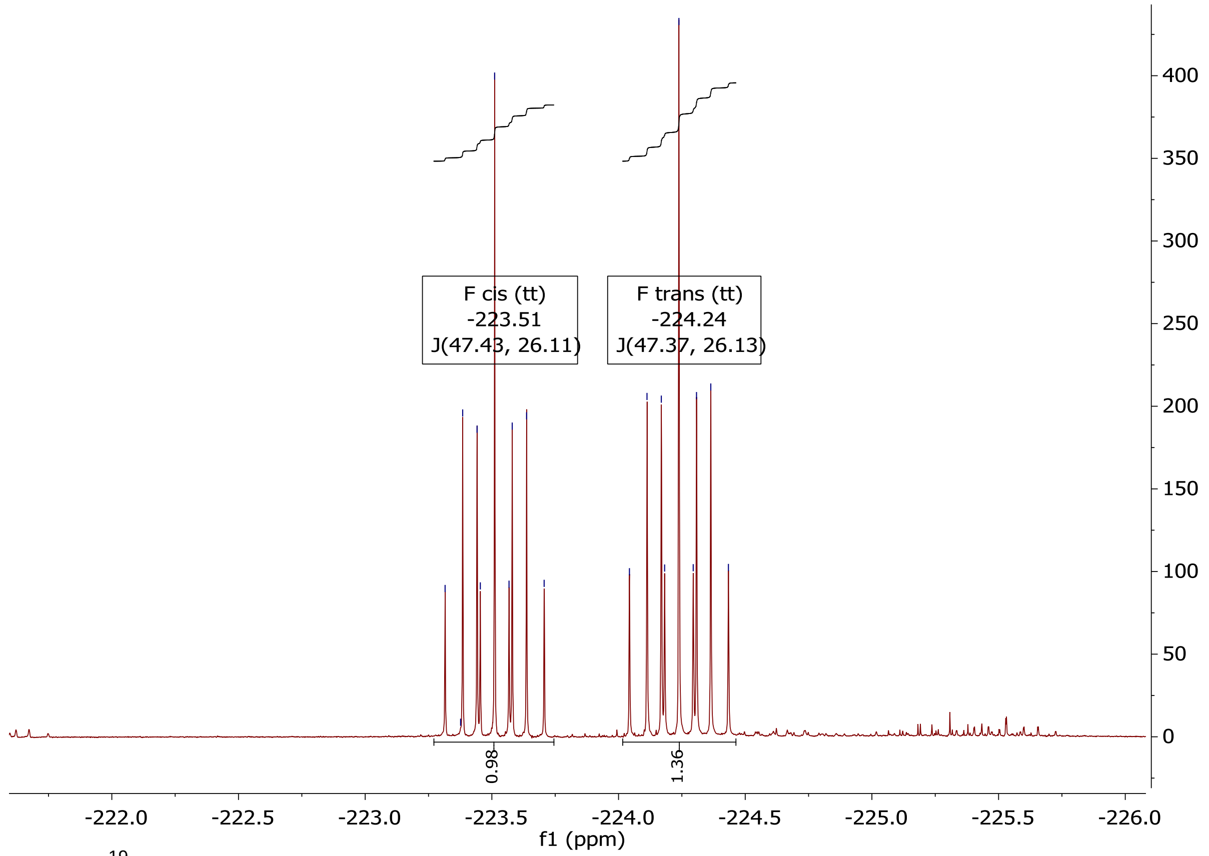

Figure S-34. ^{19}F-NMR spectrum of compound **11** recorded in *methanol-d_4* at room temperature.

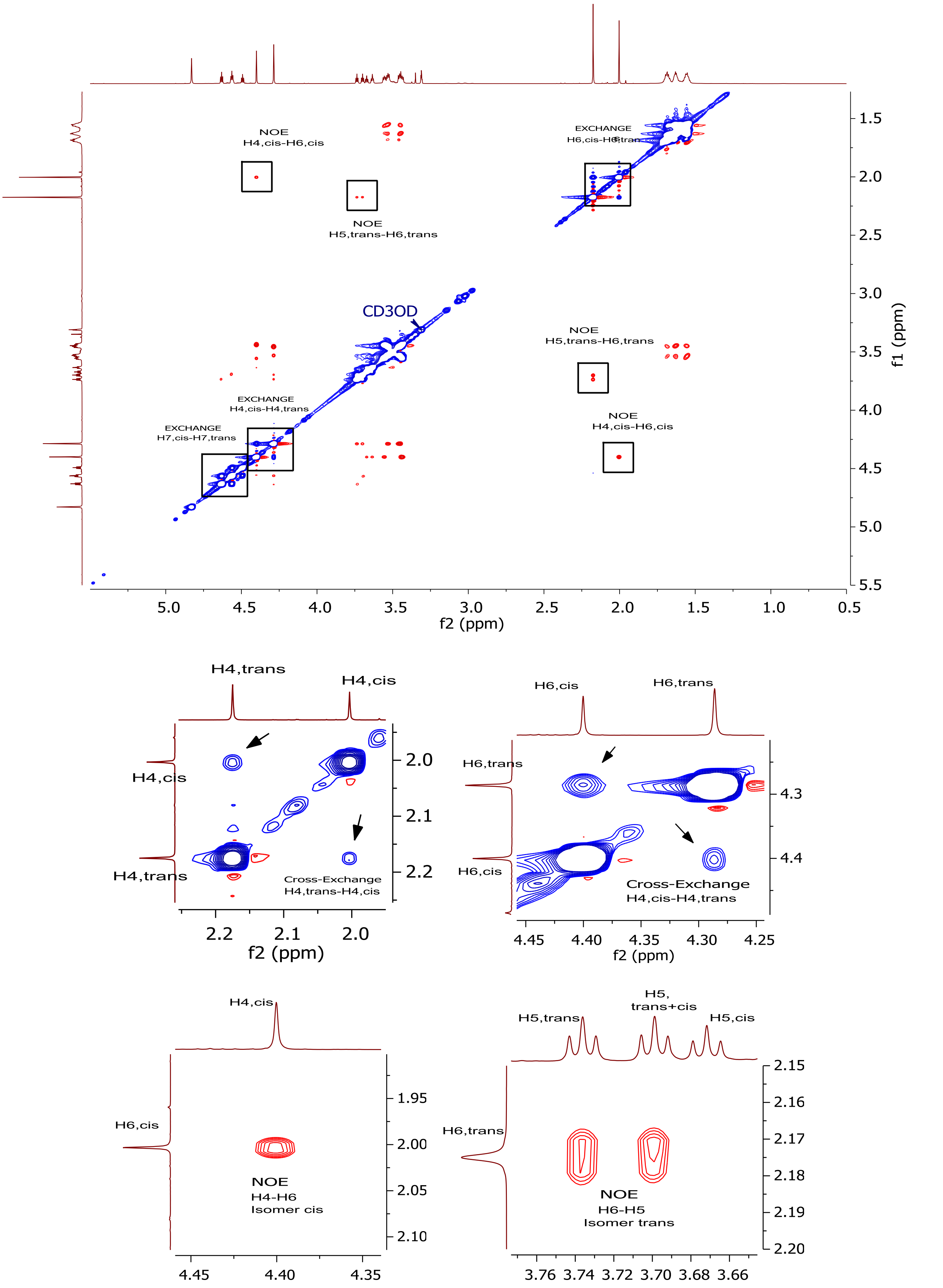

Figure S-35. ^{1}H-^{1}H NOESY spectrum of compound **11** recorded in *methanol-d$_4$* at room temperature. Insets showing the specific spatial contacts that are characteristic of each isomer and allowed the identification of *cis* and *trans* signals.

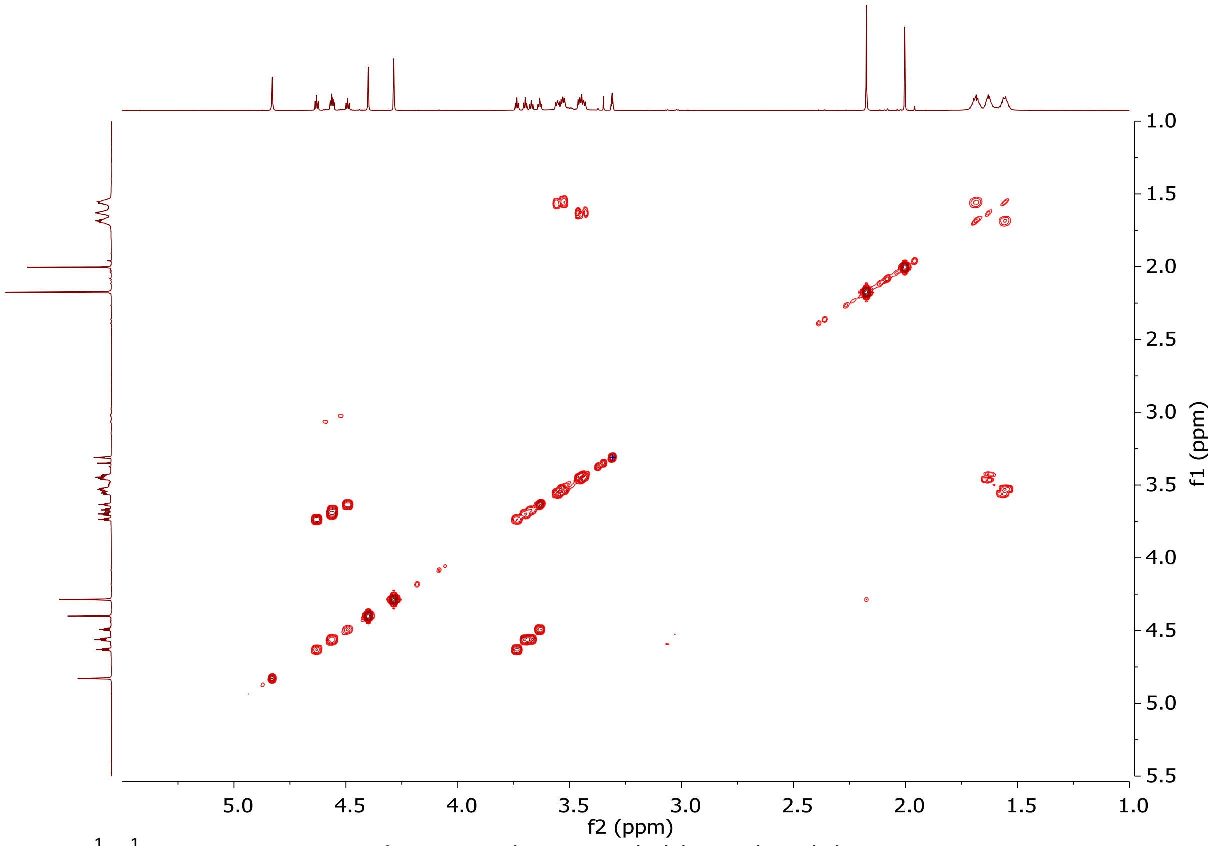

Figure S-36. ^{1}H-^{1}H COSY spectrum of compound **11** recorded in *methanol-d$_4$* at room temperature.

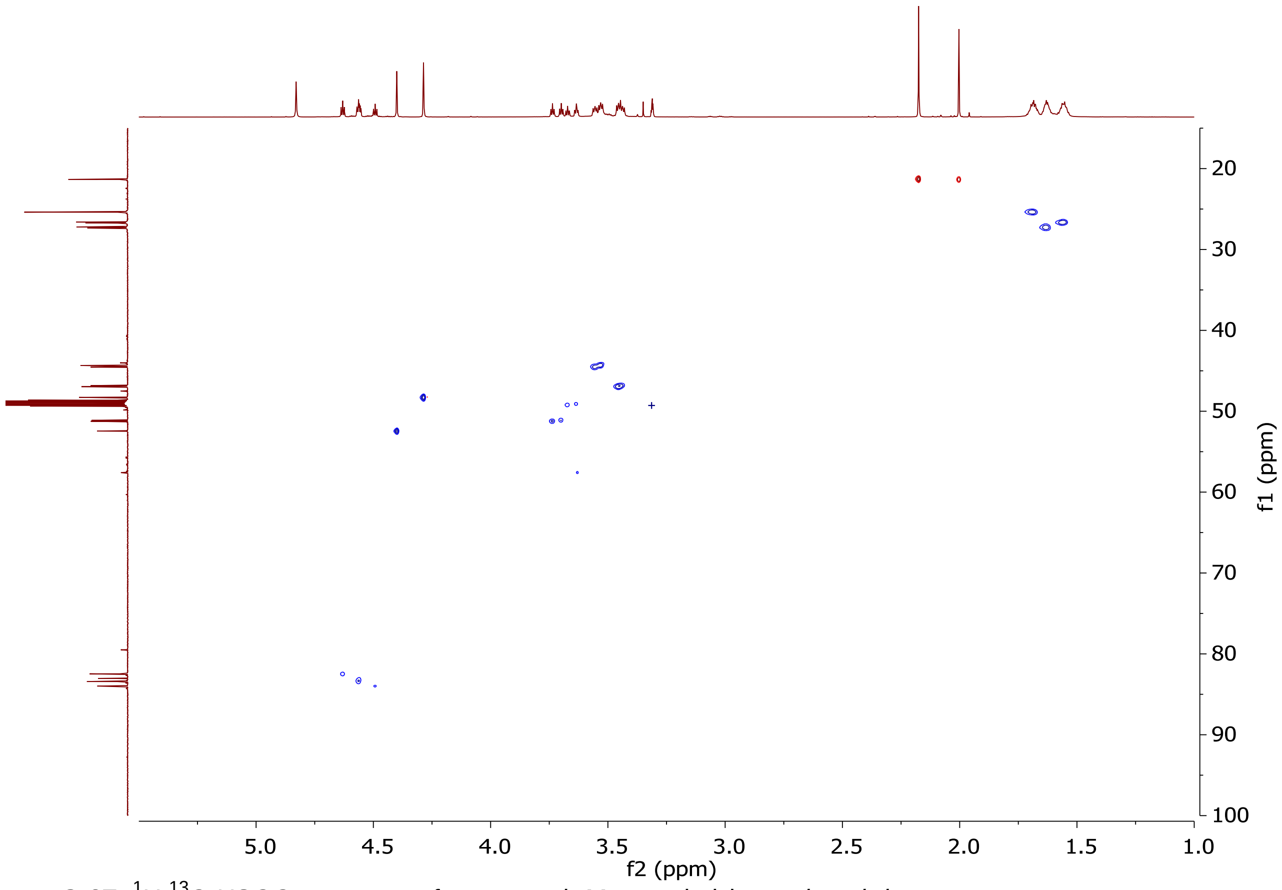

Figure S-37. ^{1}H-^{13}C HSQC spectrum of compound **11** recorded in *methanol-d$_4$* at room temperature.

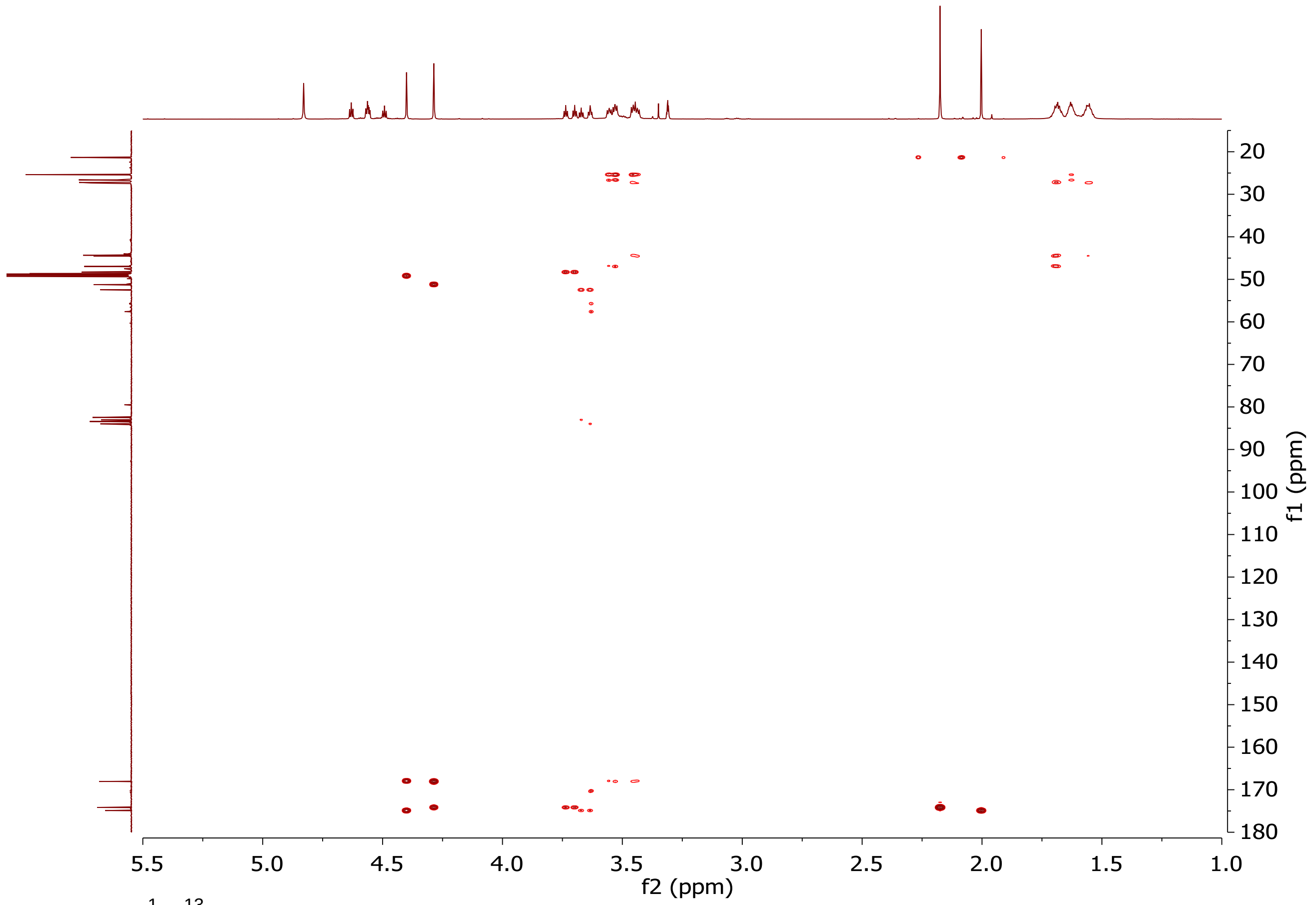

Figure S-38. ^{1}H-^{13}C HMBC spectrum of compound **11** recorded in *methanol-d$_4$* at room temperature.
.

^{1}H NMR (700MHz, Chloroform-*d*) <u>Rotamer *trans*</u>: δ ^{1}H NMR: 4.53 (dt, *J*=47.2, 5.0 Hz, 2H, $H_{7,trans}$), 4.19 (s, 2H, $H_{4,trans}$), 3.68 (dt, *J* = 25.9, 5.0 Hz, 2H, $H_{5,trans}$), 3.54-3.27 (m, 4H, $H_{3,ax+eq,trans}$), 2.12 (s, 3H, $H_{6,trans}$), 1.74-1.37 (m, 6H, $H_{1\text{-}2,ax+eq,trans}$); <u>Rotamer *cis*</u>: δ ^{1}H NMR: 4.54 (dt, *J* = 47.2, 5.0 Hz, 2H, $H_{7,trans}$), 4.14 (s, 2H, $H_{4,cis}$), 3.62 (dt, *J* = 25.9, 5.0 Hz, 2H, $H_{5,cis}$), 3.54-3.27 (m, 4H, $H_{3,ax+eq,cis}$), 1.97 (s, 3H, $H_{6,cis}$), 1.74-1.37 (m, 6H, $H_{1\text{-}2,\ ax+eq,cis}$).
^{13}C NMR (176 MHz, Chloroform-*d*) <u>Rotamer *trans*</u>: δ ^{13}C NMR: 81.79 (d, *J* = 170.4 Hz, CH_2, $C_{7,trans}$); 49.56 (d, *J* = 20.3 Hz, CH_2, $C_{5,trans}$), 46.82 (CH_2, $C_{4,trans}$), 45.98, 43.13 (CH_2, $C_{3,trans}$), 26.26, 25.46, 24.42 (CH_2, $C_{1\text{-}2,trans}$), 21.31 (CH_3, $C_{6,trans}$); <u>Rotamer *cis*</u>: δ ^{13}C NMR: 83.84 (d, *J* = 165.5 Hz, CH_2, $C_{7,cis}$), 51.52 (CH_2, $C_{4,cis}$), 47.97 (d, *J* = 19.0 Hz, CH_2, $C_{5,cis}$), 45.75, 43.43, (CH_2, $C_{3,cis}$), 26.41, 25.55, 24.36 (CH_2, $C_{1\text{-}2,cis}$), 21.35 (CH_3, $C_{6,cis}$); <u>Quaternary carbons</u>: δ ^{13}C NMR: 171.80 (**C**O-$CH_{3,cis}$), 171.18 (**C**O-$CH_{3,trans}$), 166.14 (**C**O-$pip_{,trans}$), 165.63 (**C**O-$pip_{,cis}$).
^{19}F NMR (376 MHz, Chloroform-*d*) <u>Rotamer *trans*</u>: δ: -222.12 (tt, *J* = 47.1, 25.8 Hz); <u>Rotamer *cis*</u>: δ: -221.42 (tt, *J* = 47.6, 28.80 Hz).

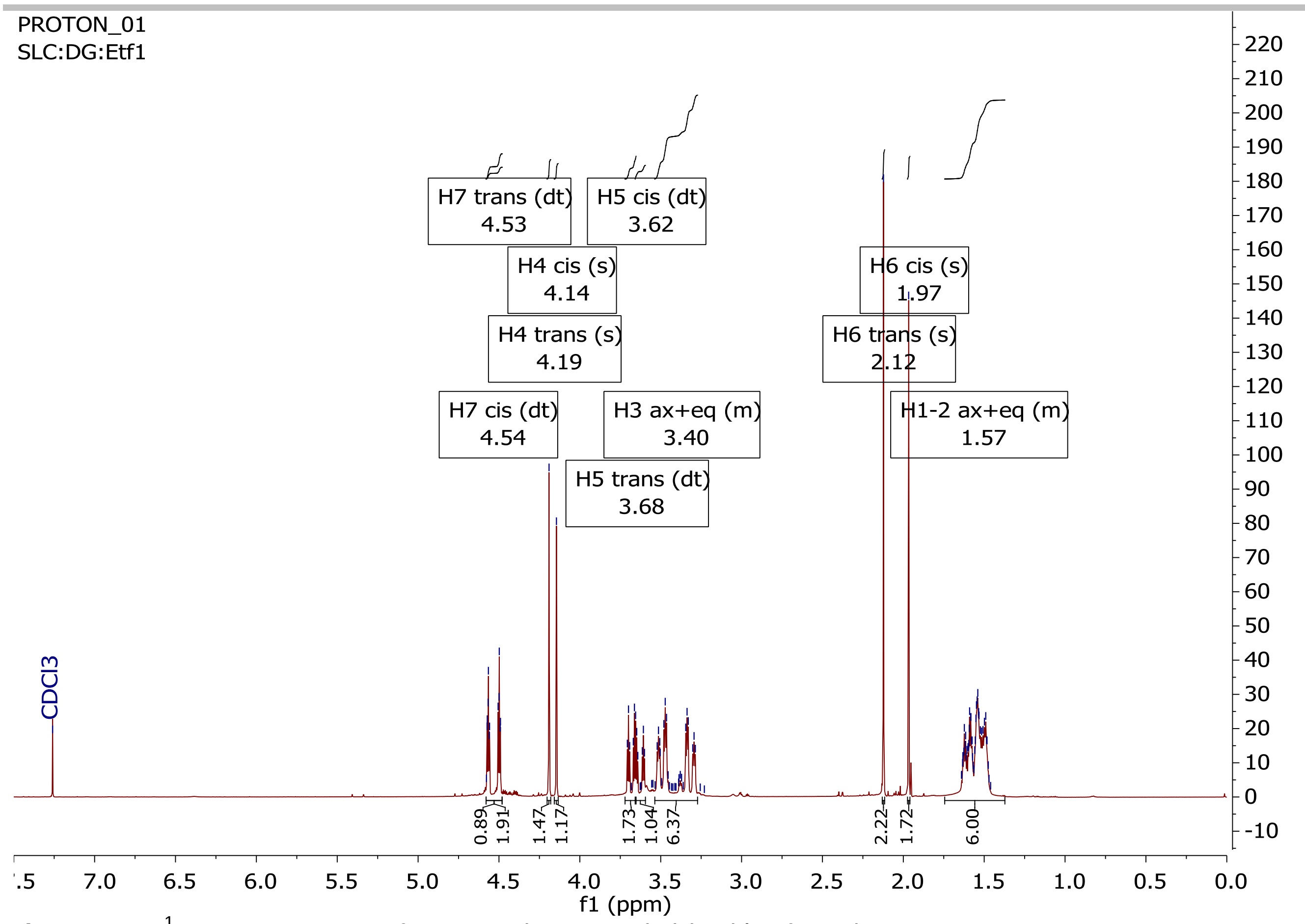

Figure S-39. ^{1}H-NMR spectrum of compound **11** recorded in chloroform-*d* at room temperature.

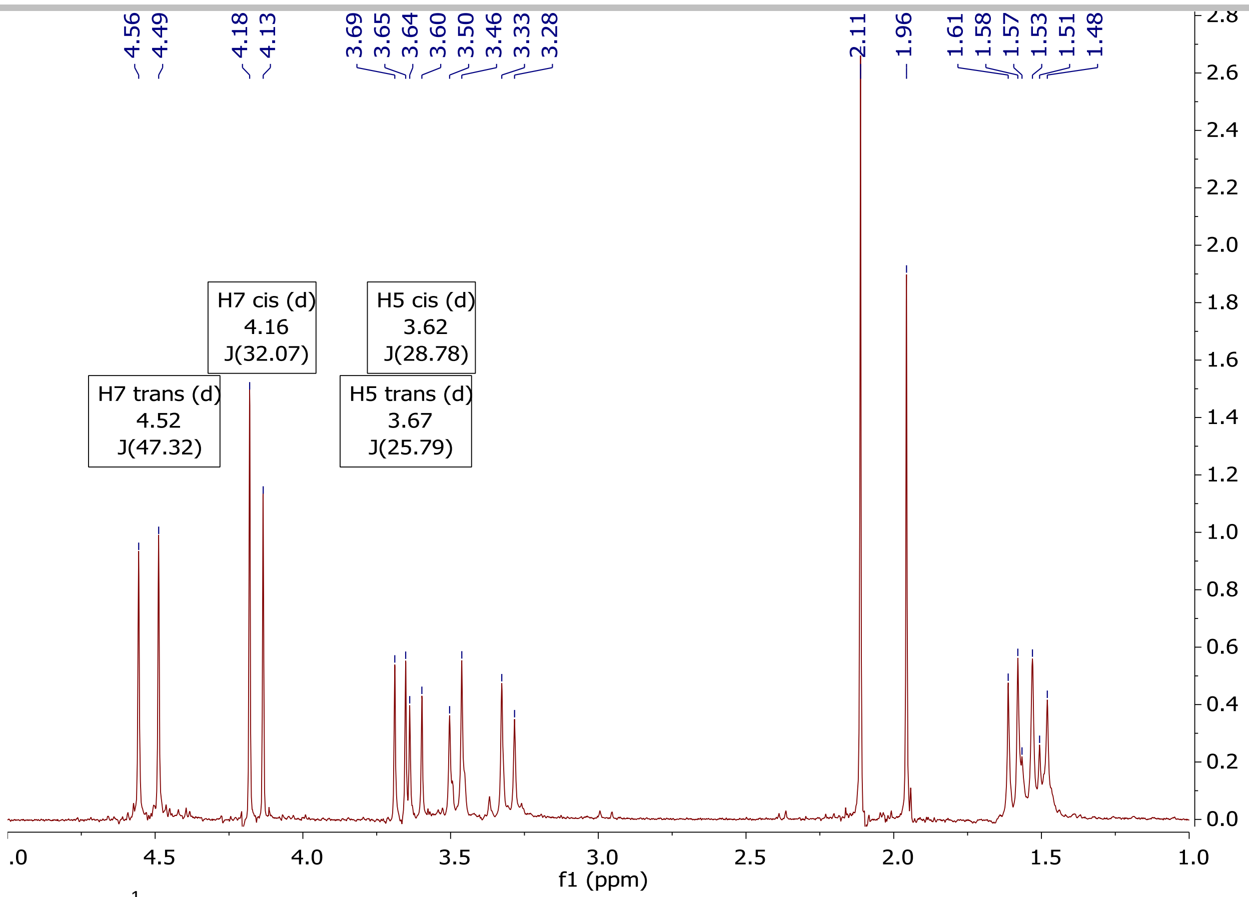

Figure S-40. ^{1}H-*psyche* spectrum of compound **11** recorded in chloroform-*d* at room temperature.

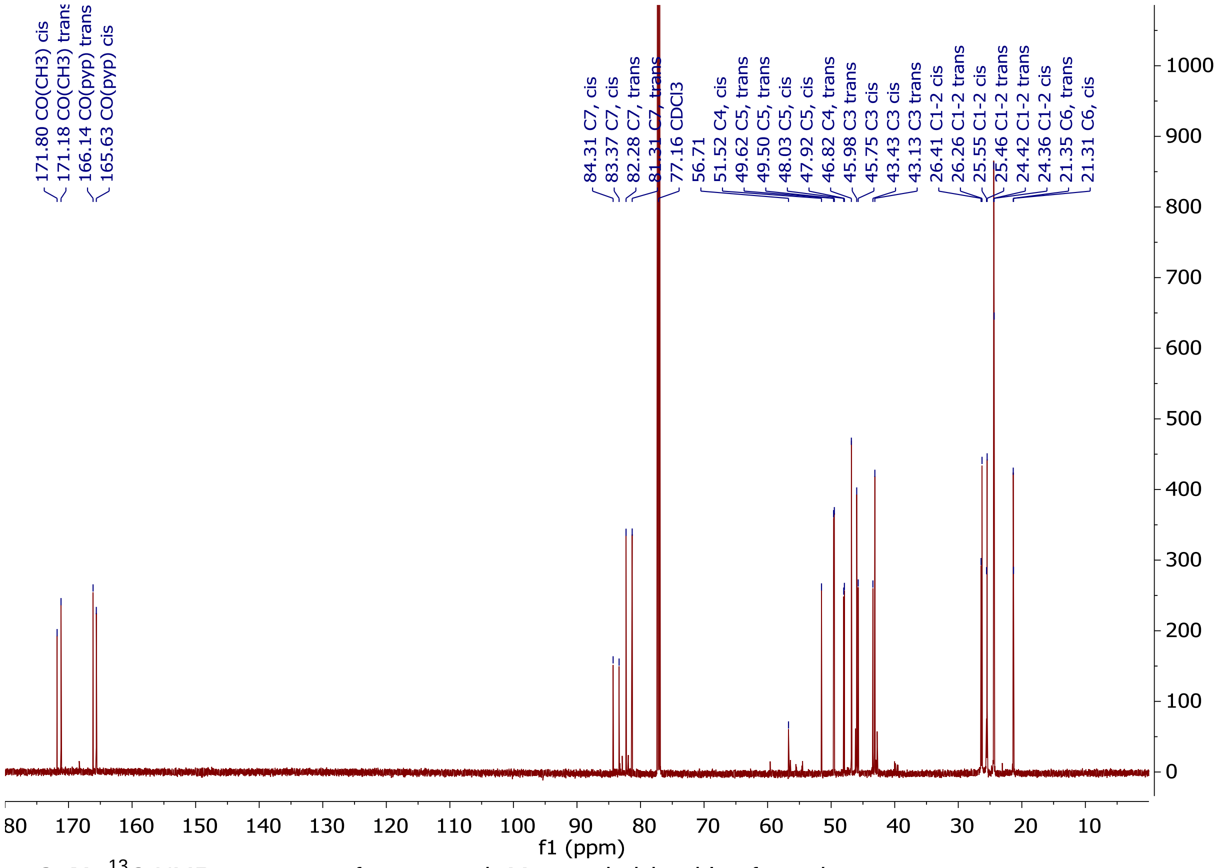

Figure S-41. ^{13}C NMR spectrum of compound **11** recorded in chloroform-*d* at room temperature.

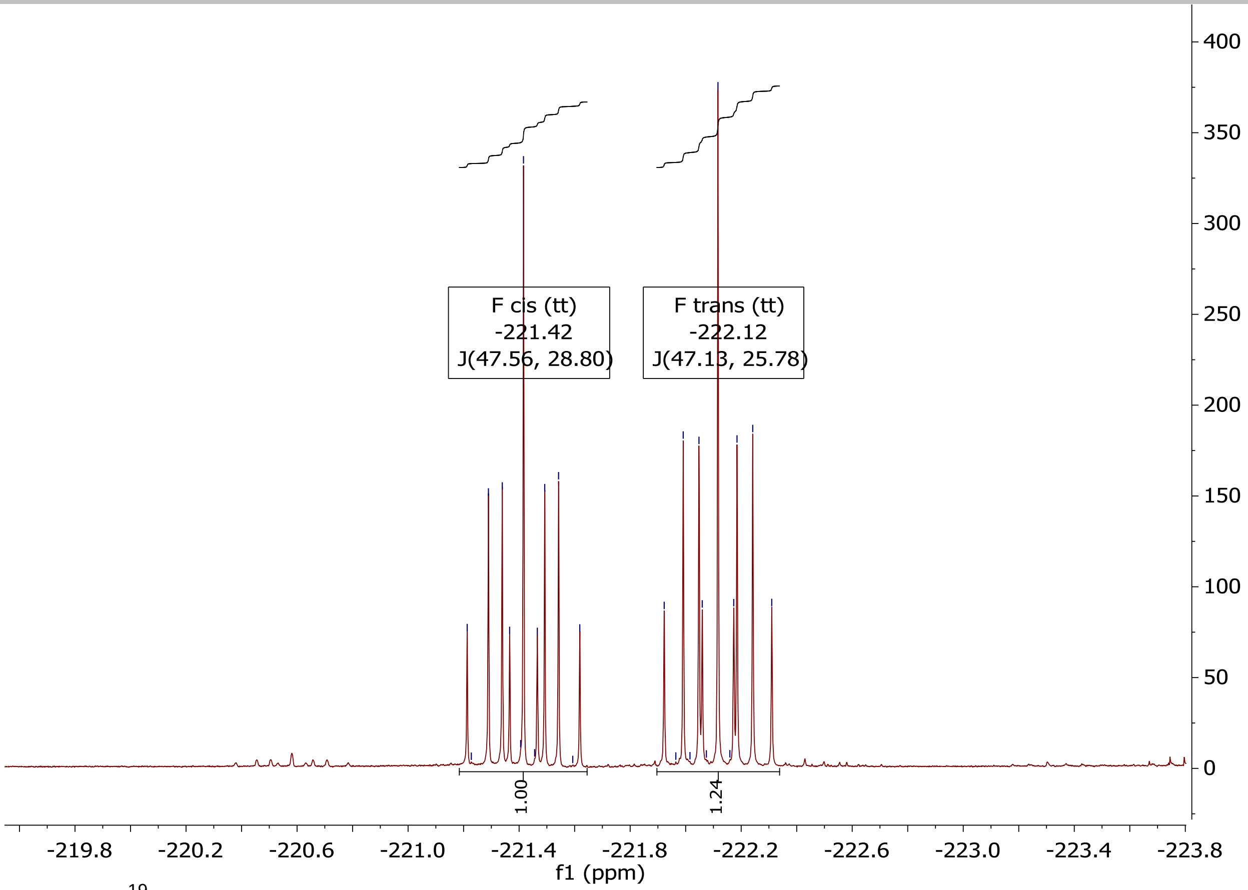

Figure S-42. ^{19}F-NMR spectrum of compound **11** recorded in chloroform-*d* at room temperature.

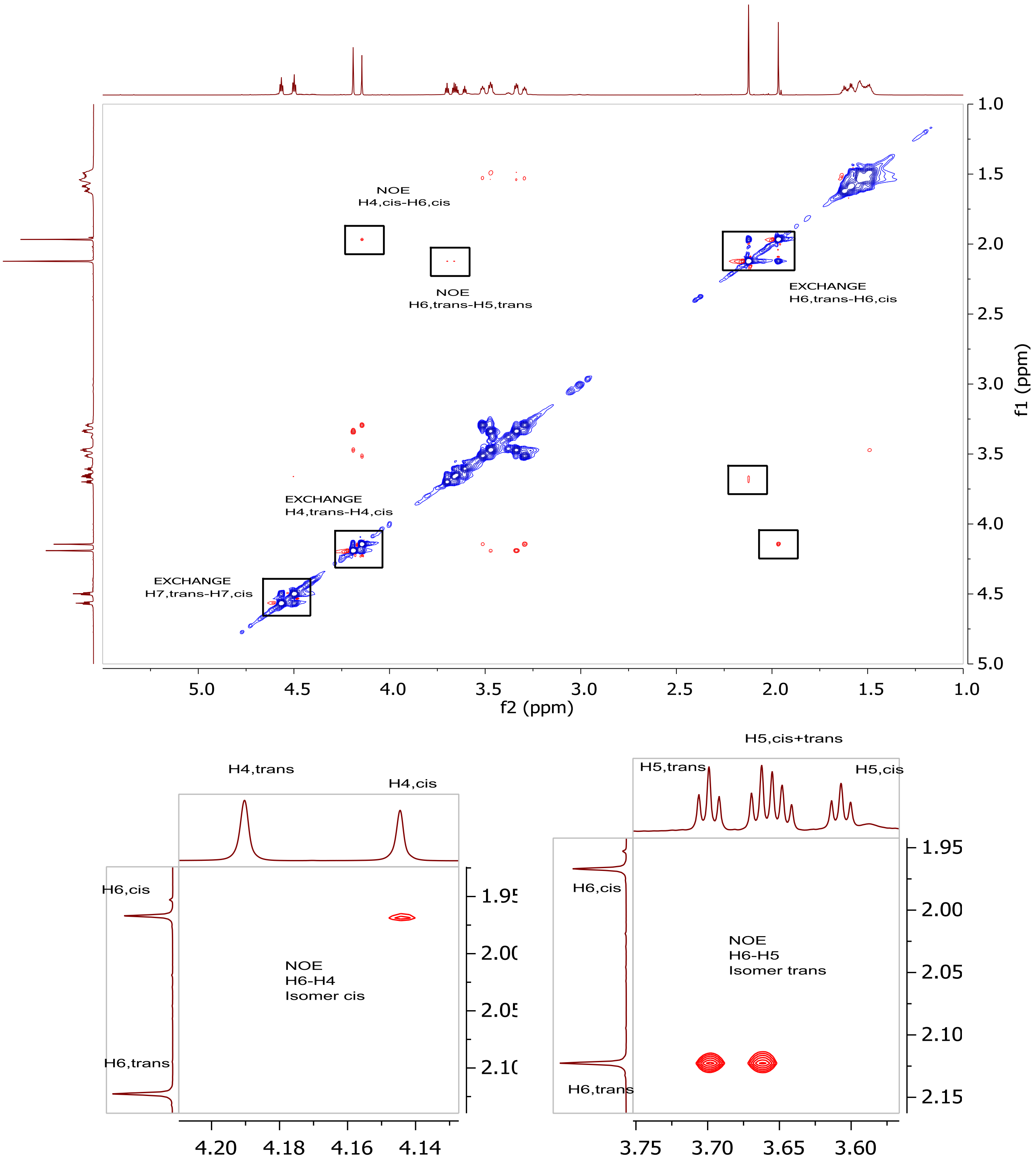

Figure S-43. 2D ^{1}H-^{1}H NOESY spectrum of compound **11** recorded in chloroform-*d* at room temperature. Inset showing the specific spatial contacts that are characteristic of each isomer and allowed the identification of *cis* and *trans* signals.

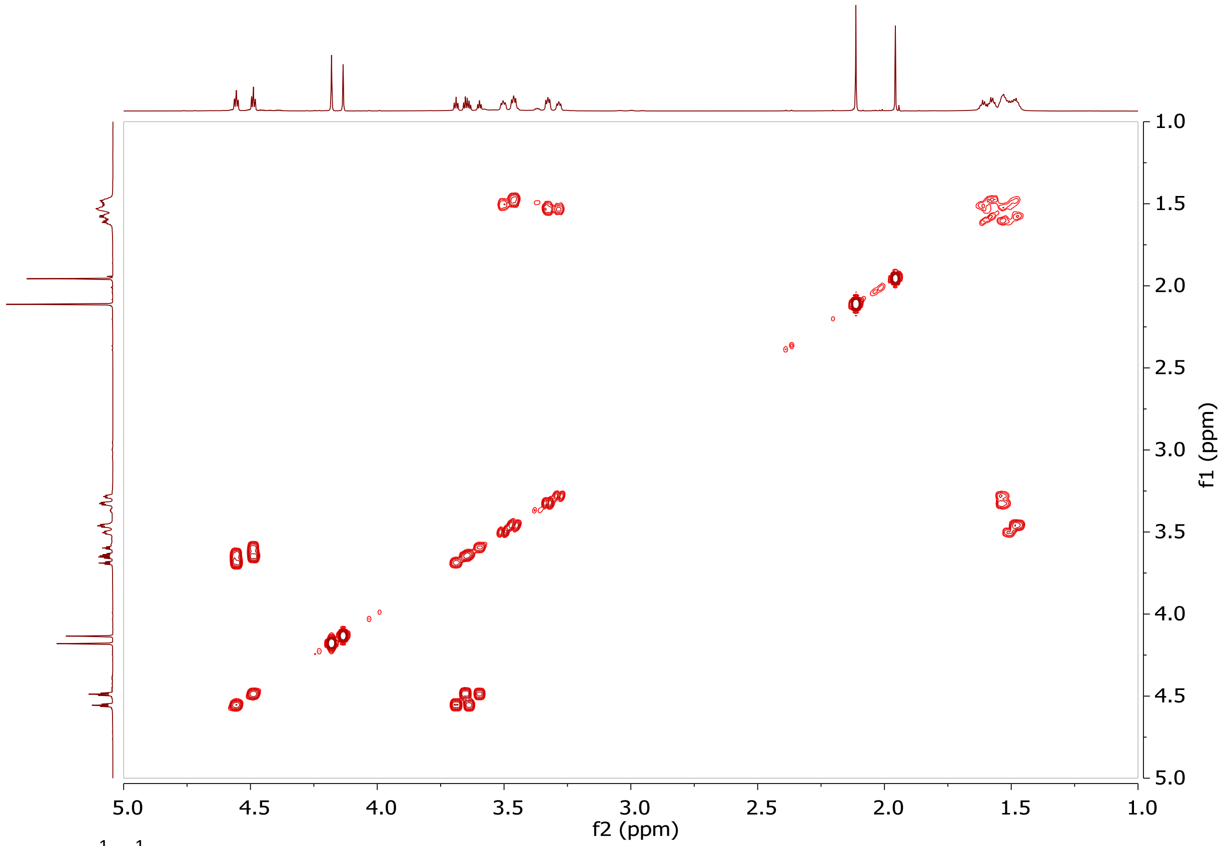

Figure S-44. ^{1}H-^{1}H COSY spectrum of compound **11** recorded in chloroform-*d* at room temperature.

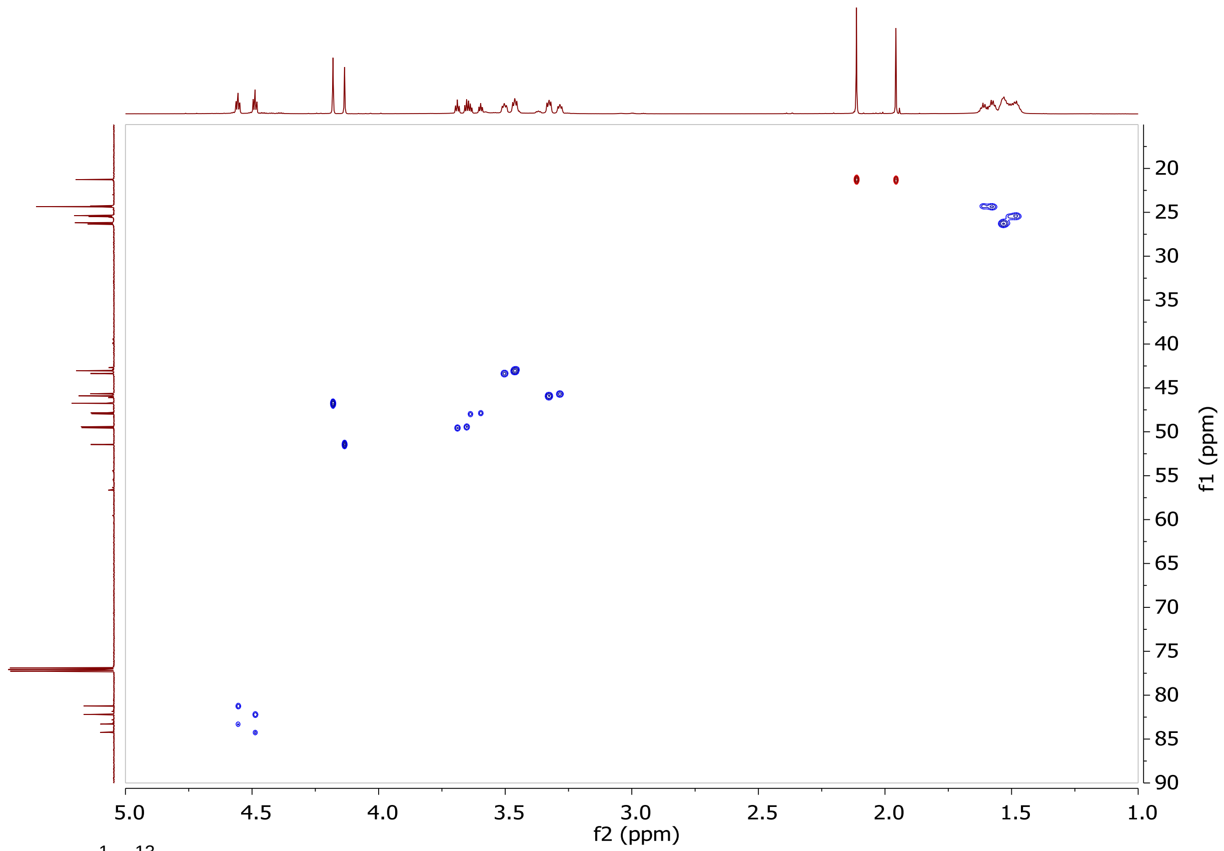

Figure S-45. ^{1}H-^{13}C HSQC spectrum of compound **11** recorded in chloroform-*d* at room temperature

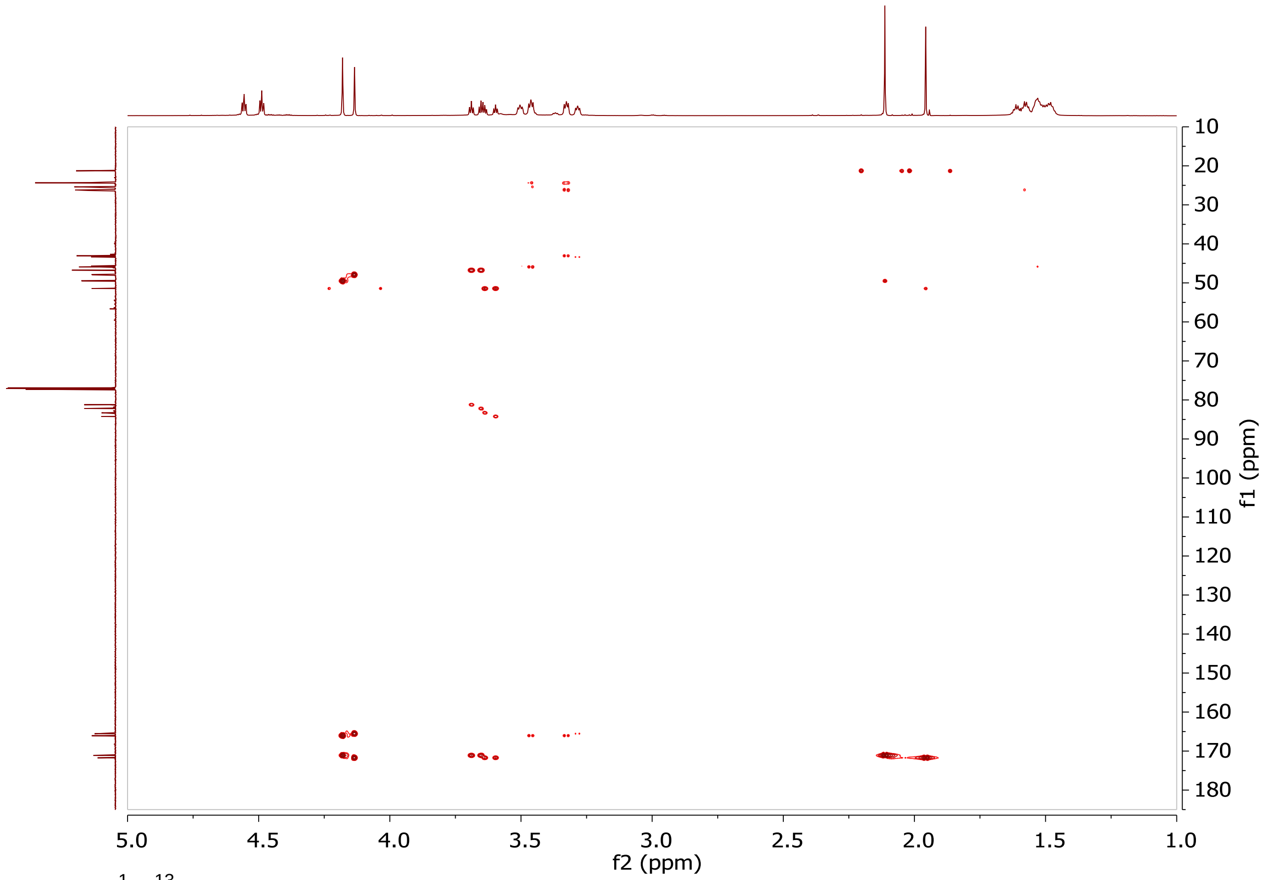

Figure S-46. ^{1}H-^{13}C HMBC spectrum of compound **11** recorded in chloroform-*d* at room temperature.

12: Ac-^{2f}Et-pip.

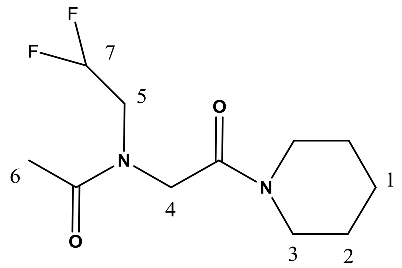

HRMS: calculated for $C_{11}H_{19}F_2N_2O_2$ 249.1415 [M+H]+, found: 249.1424 Da.

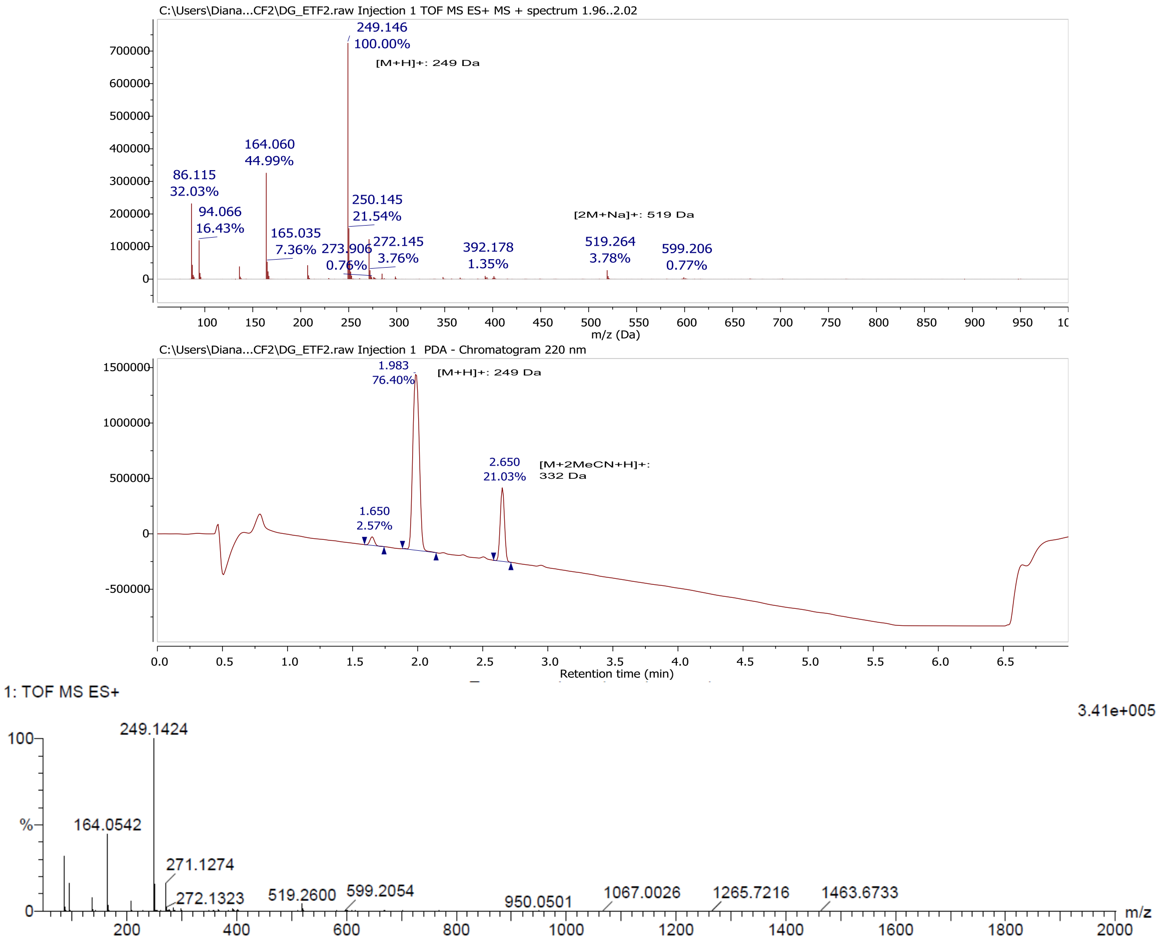

Figure S-47. Structure, HR (QToF)-LC/MS trace and elemental composition of model piperinidyl acetamide **12**.

^{1}H NMR (400 MHz, Acetonitrile-*d*$_3$) <u>Rotamer *trans*</u>: δ ^{1}H NMR: 6.05 (tt, *J* = 55.3, 3.9 Hz, 1H, $H_{7,trans}$), 4.17 (s, 2H, $H_{4,trans}$), 3.72 (td, *J* = 14.8, 3.9 Hz, 2H, $H_{5,trans}$), 3.52-3.28 (m, 4H, $H_{3,ax+eq,trans}$), 2.09 (s, 3H, $H_{6,trans}$), 1.68-1.43 (m, 6H, $H_{1\text{-}2,ax+eq,trans}$); <u>Rotamer *cis*</u>: δ ^{1}H NMR: 5.93 (tt, *J* = 56.3, 4.3 Hz, 1H, $H_{7,cis}$), 4.22 (s, 2H, $H_{4,cis}$), 3.61 (td, *J* = 14.8, 4.3 Hz, 2H, $H_{5,cis}$), 3.52-3.28 (m, 4H, $H_{3,ax+eq,cis}$), 1.91 (s, 3H, $H_{6,cis}$), 1.68-1.43 (m, 6H, $H_{1\text{-}2,\ ax+eq,cis}$).
^{13}C NMR (176 MHz, Acetonitrile-*d*$_3$) <u>Rotamer *trans*</u>: δ ^{13}C NMR: 116.04 (t, *J* = 240.8 Hz, CH, $C_{7,trans}$); 52.21 (t, *J* = 25.8 Hz, CH_2, $C_{5,trans}$), 48.89 (CH_2, $C_{4,trans}$), 46.29, 43.58 (CH_2, $C_{3,trans}$), 26.93, 26.33, 25.12 (CH_2, $C_{1\text{-}2,trans}$), 21.58 (CH_3, $C_{6,trans}$); <u>Rotamer *cis*</u>: δ ^{13}C NMR: 115.94 (t, *J* = 240.3 Hz, CH, $C_{7,cis}$), 52.28 (CH_2, $C_{4,cis}$), 50.27 (t, *J* = 27.1 Hz, CH_2, $C_{5,cis}$), 46.23, 43.84 (CH_2, $C_{3,cis}$), 26.96, 26.37, 25.06 (CH_2, $C_{1\text{-}2,cis}$), 21.50 (CH_3, $C_{6,cis}$); <u>Quaternary carbons</u>: δ ^{13}C NMR: 173.47 (**C**O-$CH_{3,cis}$), 172.12 (**C**O-$CH_{3,trans}$), 166.81 (**C**O-$pip_{,trans}$), 166.66 (**C**O-$pip_{,cis}$).
^{19}F NMR (376 MHz, Acetonitrile-*d*$_3$) <u>Rotamer *trans*</u>: δ ^{19}F NMR: -123.18 (dt, *J* = 55.3, 14.8 Hz); <u>Rotamer *cis*</u>: δ: -121.92 (dt, *J* = 56.3, 14.9 Hz).

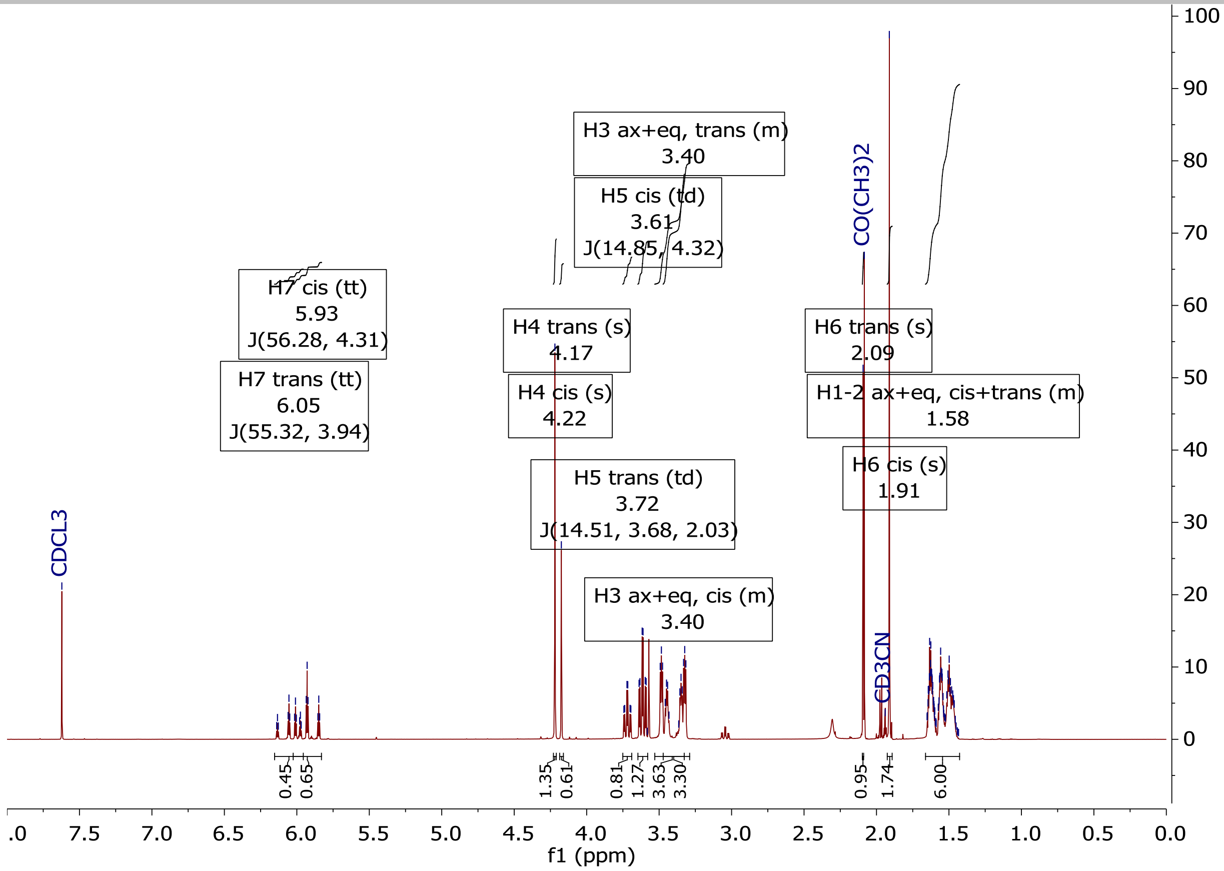

Figure S-48. 1H-NMR spectrum of compound **12** recorded in *acetonitrile-d_3* at room temperature.

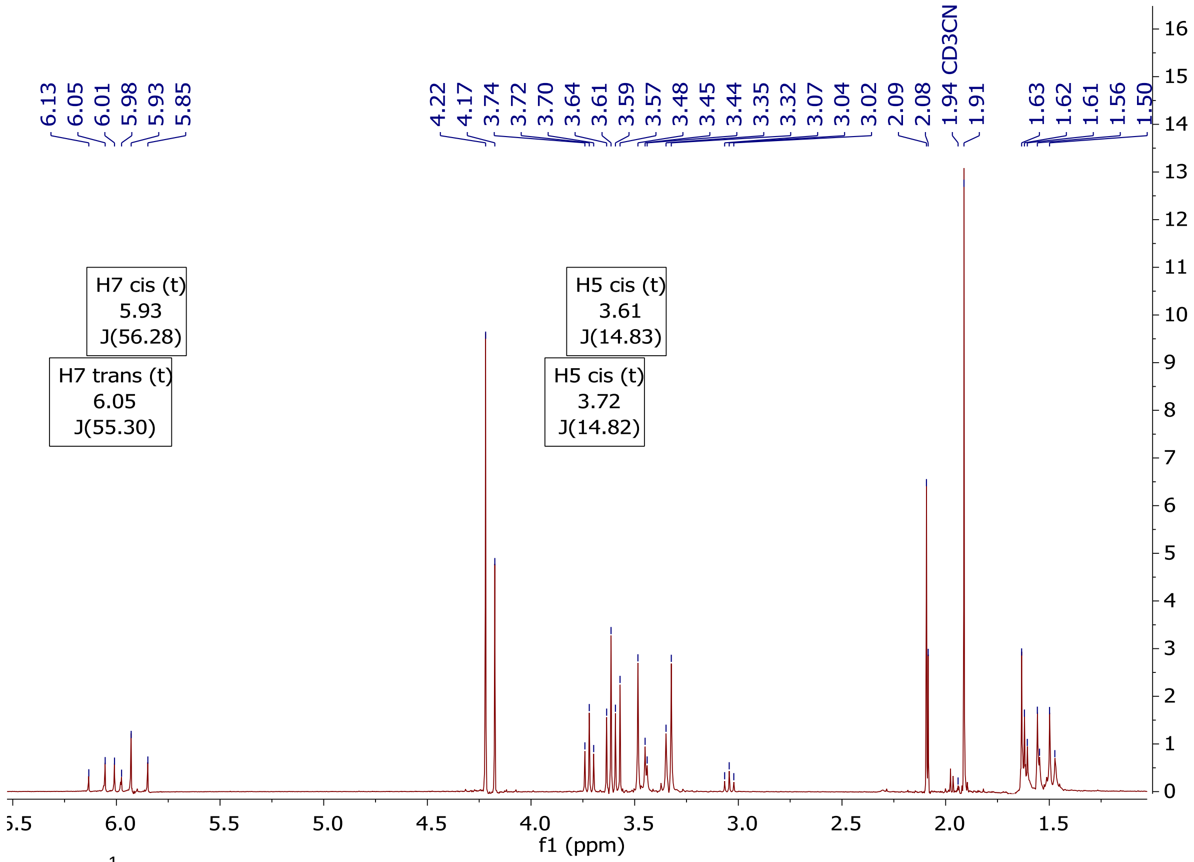

Figure S-49. ^{1}H-*psyche* spectrum of compound **12** recorded in *acetonitrile-d_3* at room temperature.

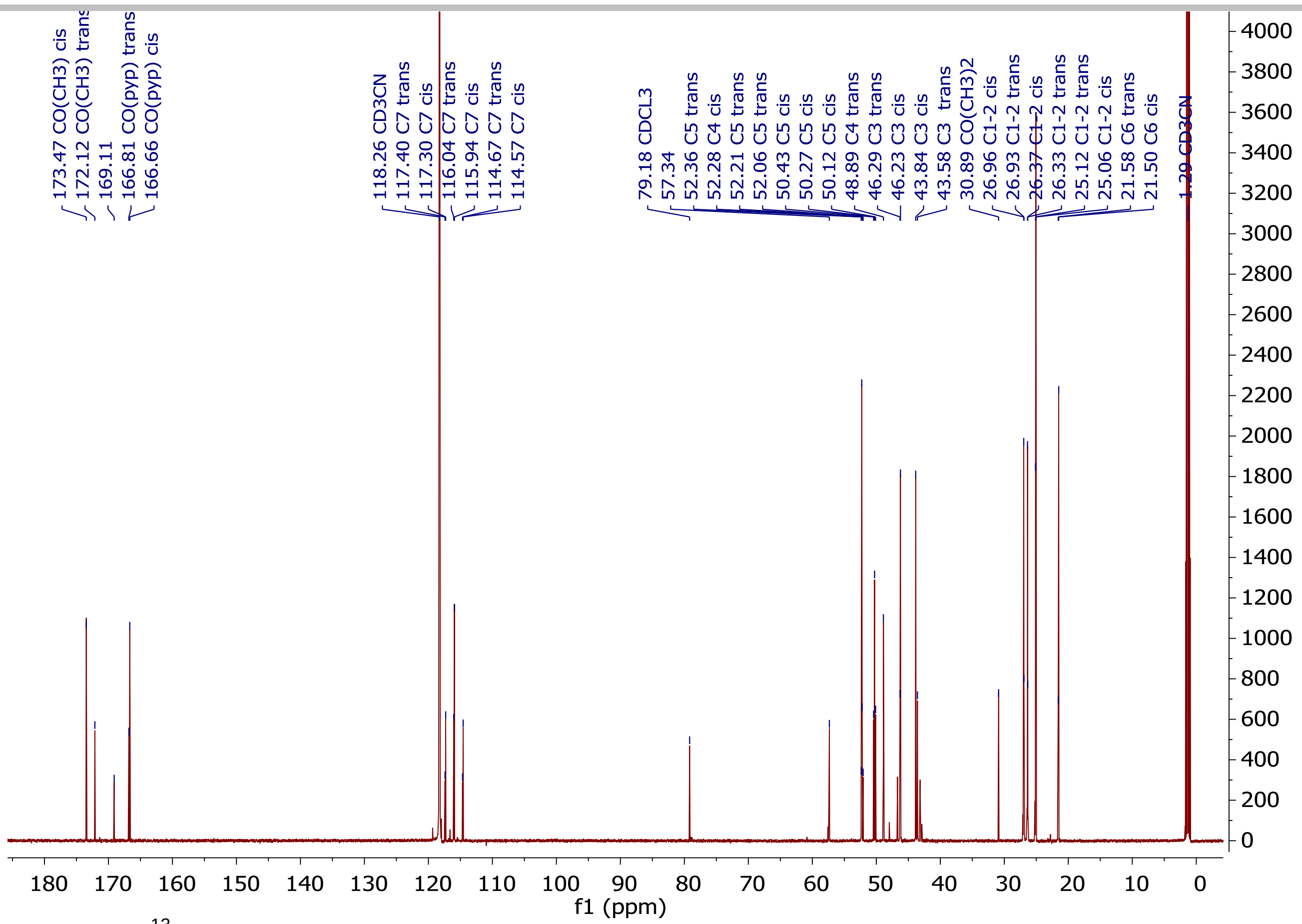

Figure S-50. ^{13}C NMR spectrum of compound **12** recorded in *acetonitrile-d_3* at room temperature.

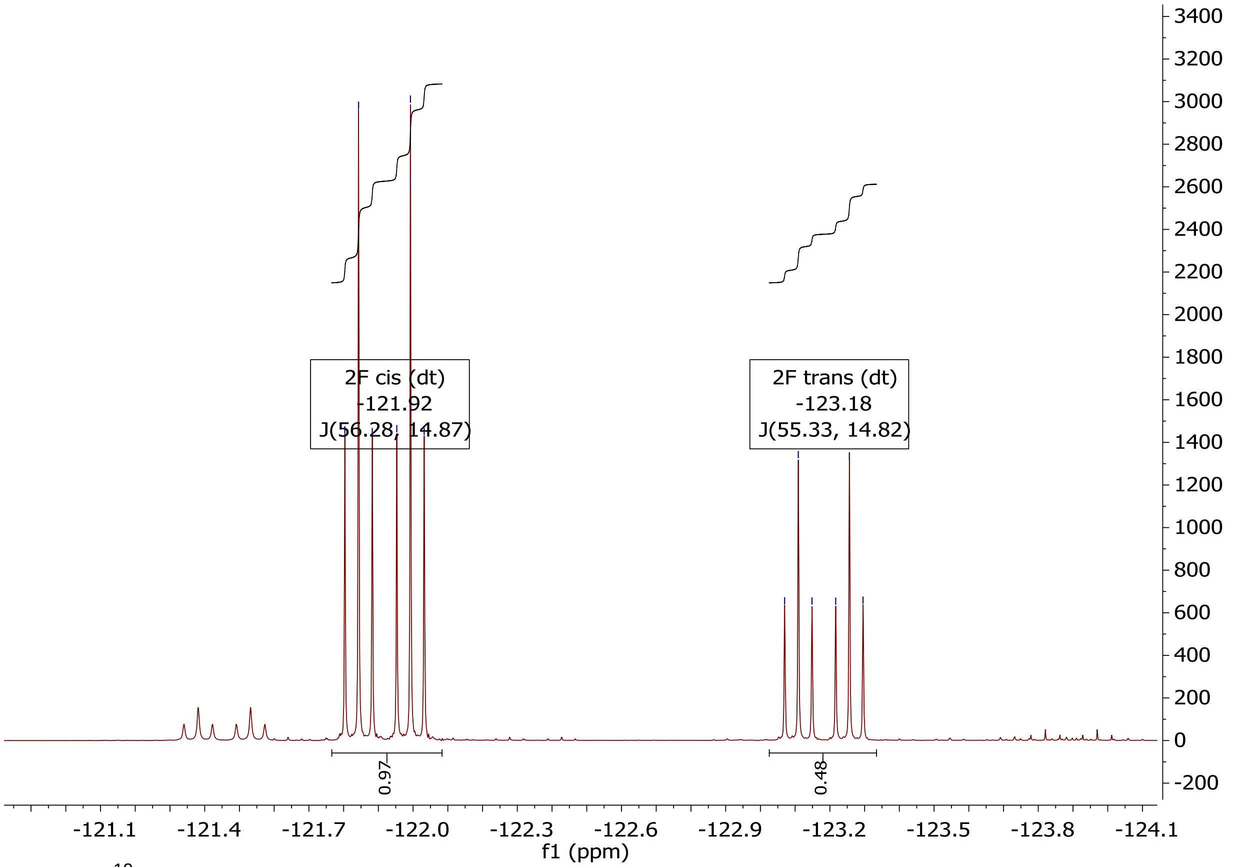

Figure S-51. ^{19}F-NMR spectrum of compound **12** recorded in *acetonitrile-d_3* at room temperature.

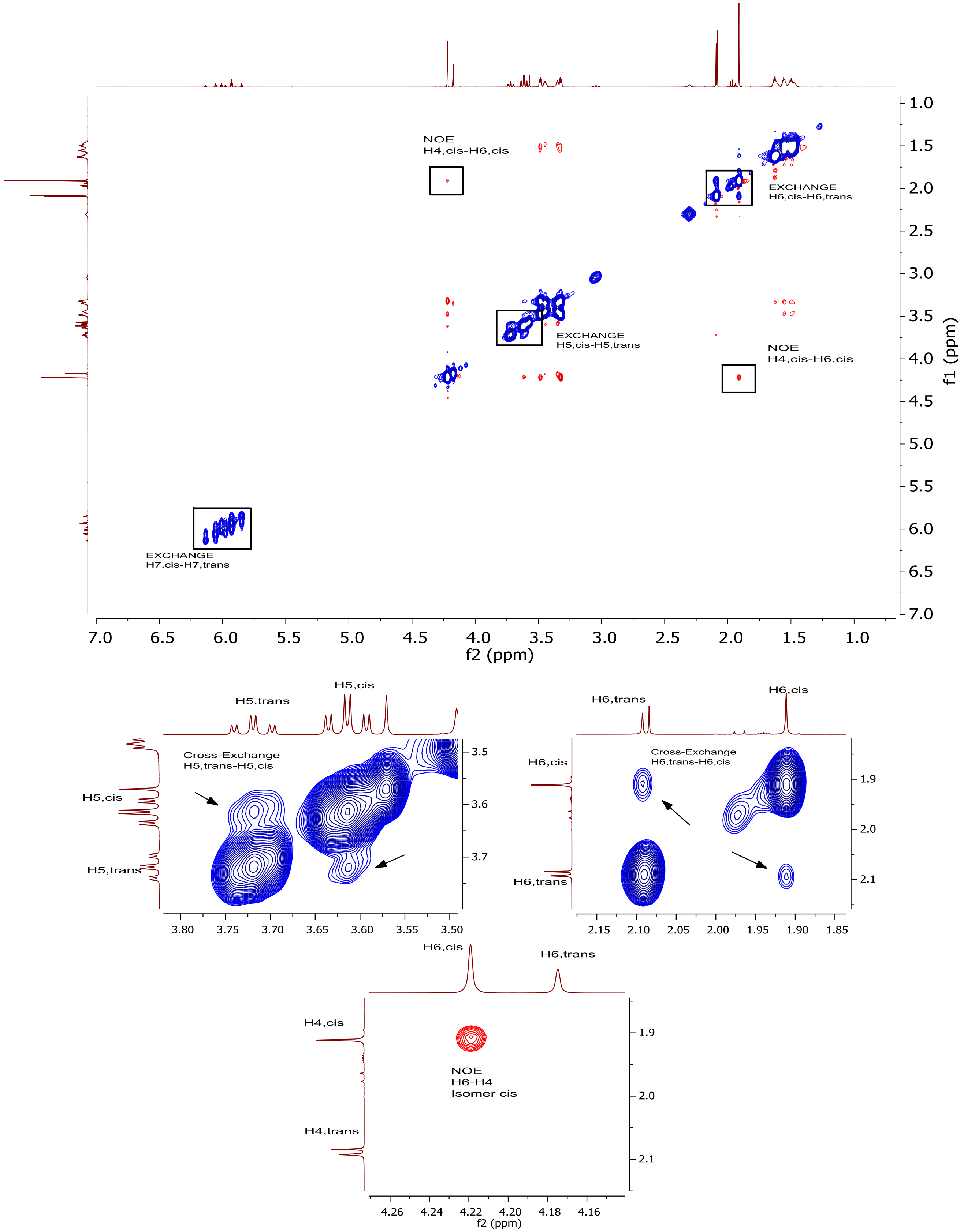

Figure S-52. ^{1}H-^{1}H NOESY spectrum of compound **12** recorded in *acetonitrile-d_3* at room temperature. Inset showing the specific spatial contacts that are characteristic of each isomer and allowed the identification of *cis* and *trans* signals.

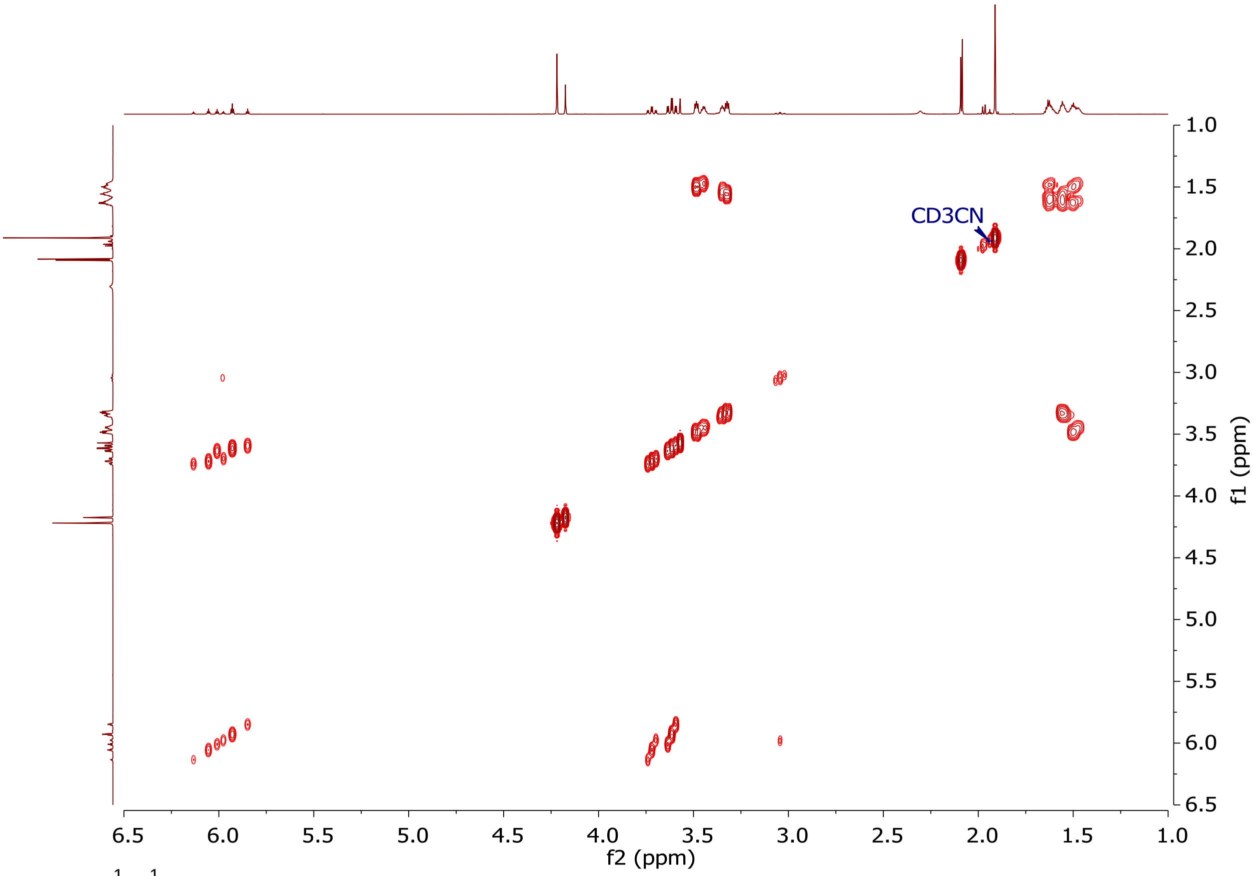

Figure S-53. ^{1}H-^{1}H COSY spectrum of compound **12** recorded in *acetonitrile-d_3* at room temperature.

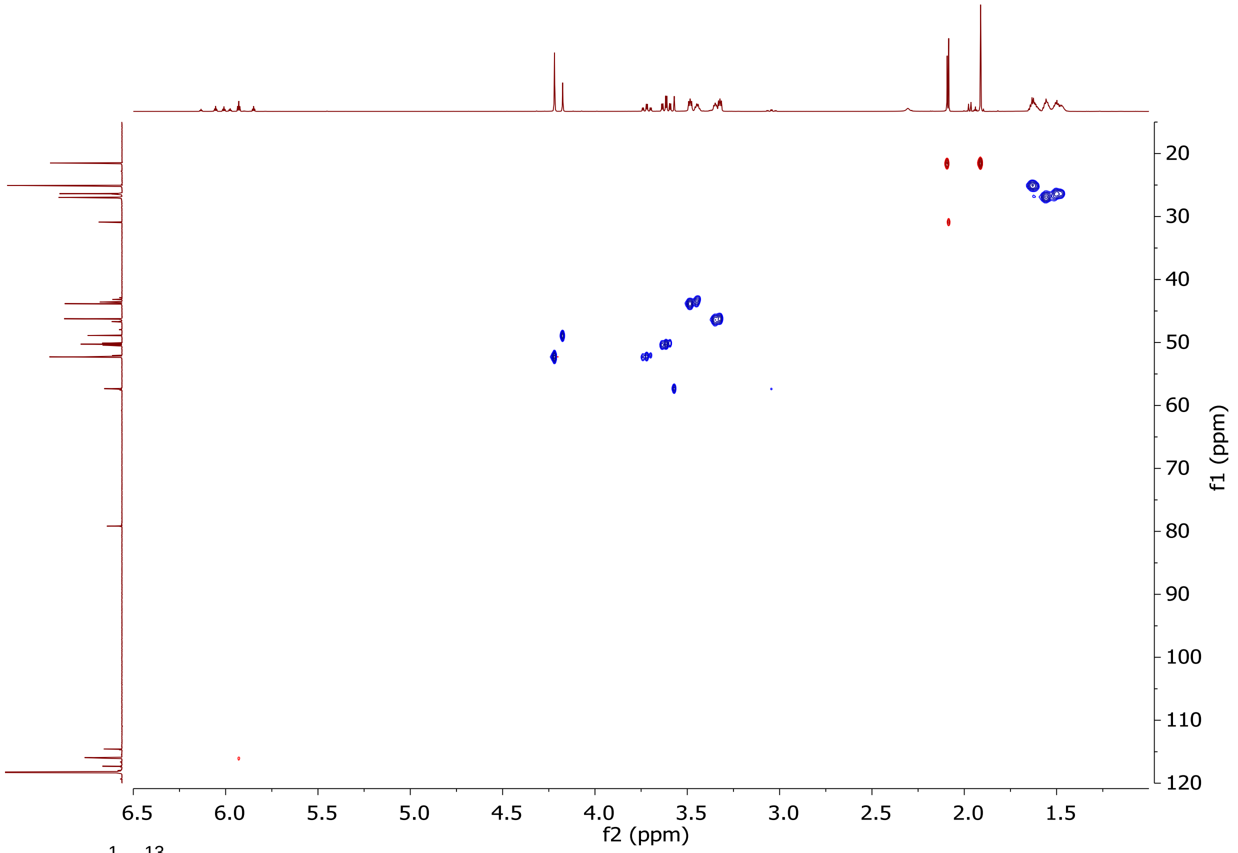

Figure S-54. ^{1}H-^{13}C HSQC spectrum of compound **12** recorded in *acetonitrile-d_3* at room temperature

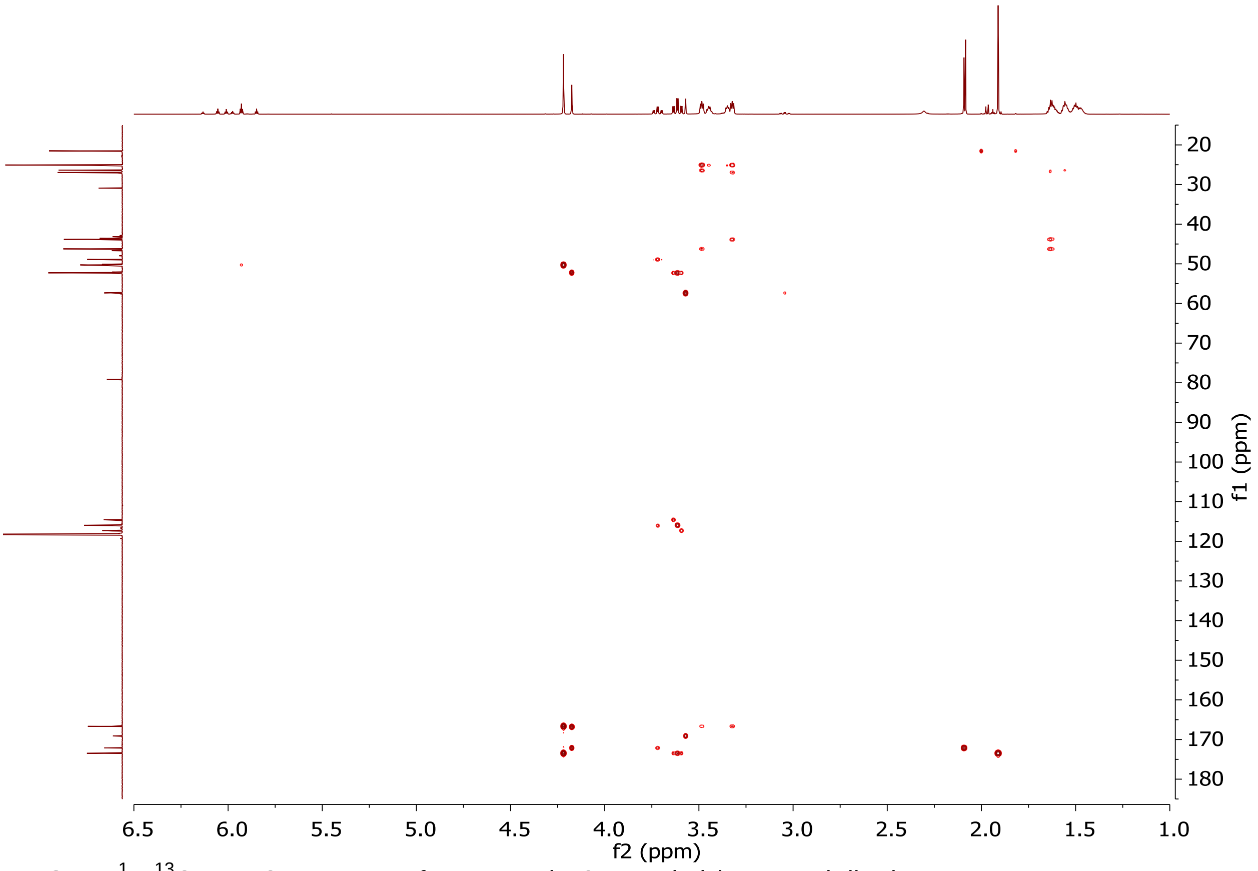

Figure S-55. ^{1}H-^{13}C HMBC spectrum of compound **12** recorded in *acetonitrile-d_3* at room temperature.

^{1}H NMR (700 MHz, Methanol-d_4) Rotamer *trans*: δ ^{1}H NMR: 6.12 (tt, J = 55.2, 3.7 Hz, 1H, $H_{7,trans}$), 4.31 (s, 2H, $H_{4,trans}$), 3.81 (td, J = 14.8, 3.8 Hz, 2H, $H_{5,trans}$), 3.53-3.41 (m, 4H, $H_{3,ax+eq,trans}$), 2.20 (s, 3H, $H_{6,trans}$), 1.75-1.48 (m, 6H, $H_{1\text{-}2,ax+eq,trans}$); Rotamer *cis*: δ ^{1}H NMR: 5.95 (tt, J = 56.2, 4.3 Hz, 1H, $H_{7,cis}$), 4.41 (s, 2H, $H_{4,cis}$), 3.70 (td, J = 14.5, 4.3 Hz, 2H, $H_{5,cis}$), 3.57-3.41 (m, 4H, $H_{3,ax+eq,cis}$), 2.02 (s, 3H, $H_{6,cis}$), 1.75-1.48 (m, 6H, $H_{1\text{-}2,ax+eq,cis}$).
^{13}C NMR (176 MHz, Methanol-d_4) Rotamer *trans*: δ ^{13}C NMR: 116.01 (t, J = 241.2 Hz, CH, $C_{7,trans}$); 52.71 (t, J = 25.6 Hz, CH_2, $C_{5,trans}$), 49.39 (CH_2, $C_{4,trans}$), 46.93, 44.35 (CH_2, $C_{3,trans}$), 27.19, 26.59, 25.36 (CH_2, $C_{1\text{-}2,trans}$), 21.28 (CH_3, $C_{6,trans}$); Rotamer *cis*: δ ^{13}C NMR: 115.81 (t, J = 240.8 Hz, CH, $C_{7,cis}$), 52.56 (CH_2, $C_{4,cis}$), 50.92 (t, J = 27.4, CH_2, $C_{5,cis}$), 46.78, 44.35 (CH_2, $C_{3,cis}$), 27.33, 26.68, 25.30 (CH_2, $C_{1\text{-}2,cis}$), 21.20 (CH_3, $C_{6,cis}$); Quaternary carbons: δ ^{13}C NMR: 175.44 (**CO**-$CH_{3,cis}$), 174.44 (**CO**-$CH_{3,trans}$), 167.84 (**CO**-$pip_{,trans}$), 167.67 (**CO**-$pip_{,cis}$).
^{19}F NMR (376 MHz, Methanol-d_4) Rotamer *trans*: δ ^{19}F NMR: -123.77 (dt, J = 55.2, 14.8 Hz); Rotamer *cis*: δ: -122.36 (dt, J = 56.2, 14.5).

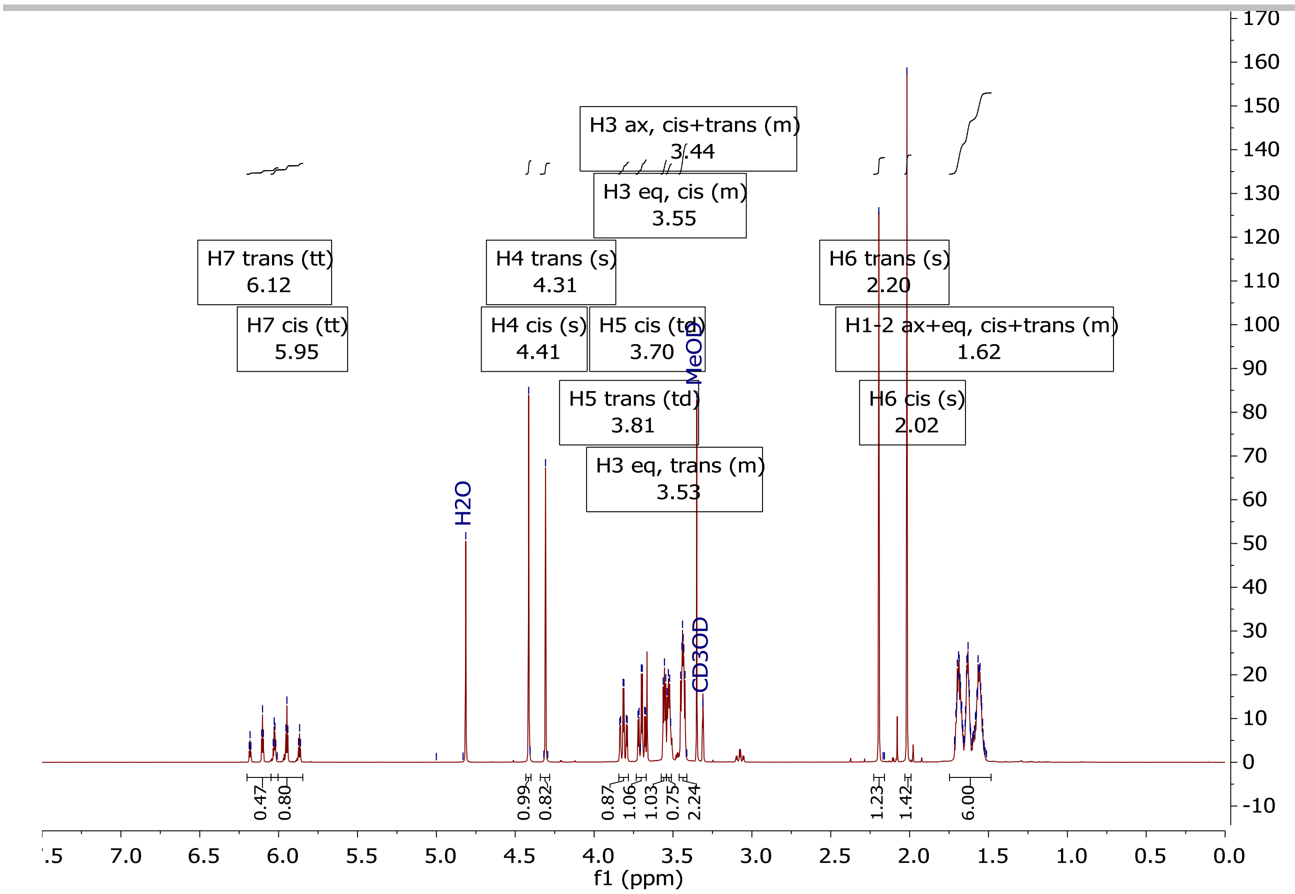

Figure S-56. 1H-NMR spectrum of compound **12** recorded in *methanol-d_4* at room temperature.

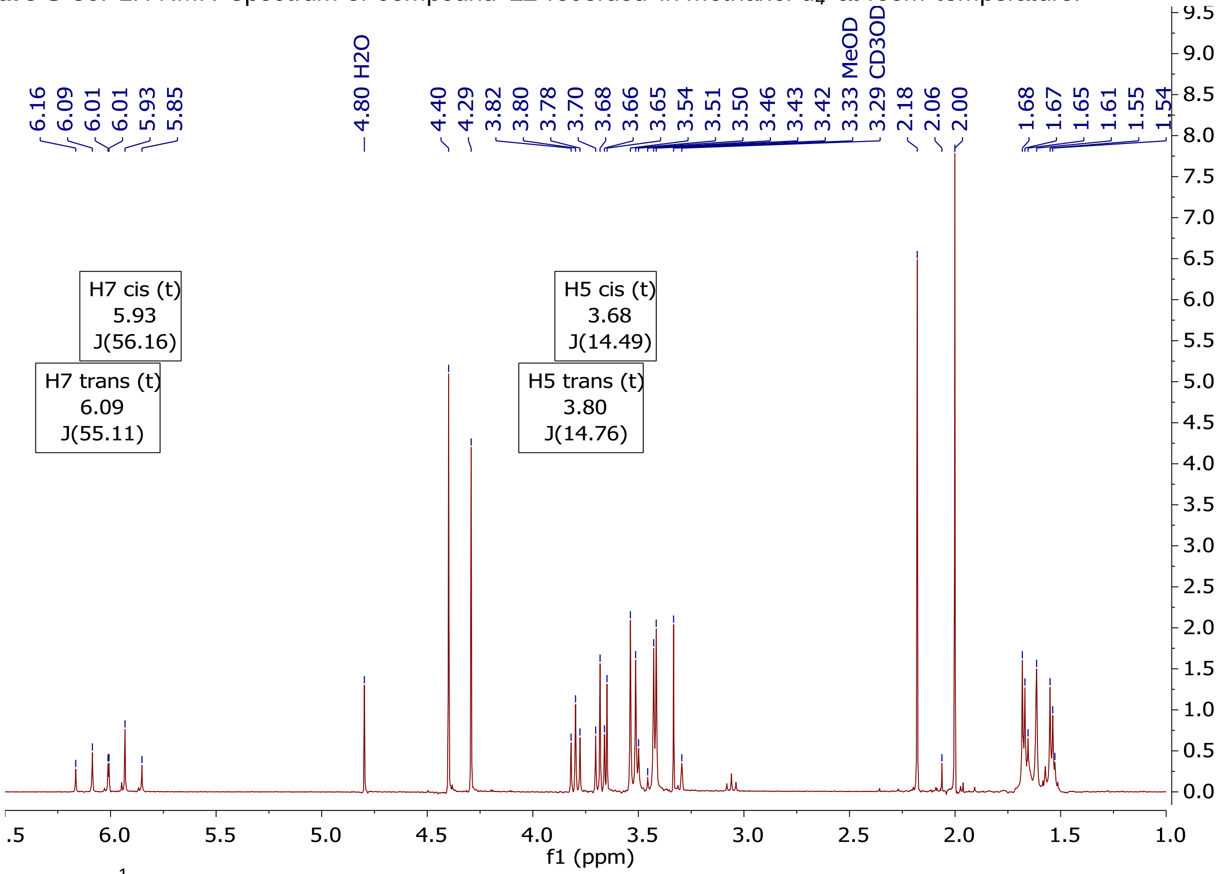

Figure S-57. ^{1}H-*psyche* spectrum of compound **12** recorded in *methanol-d_4* at room temperature.

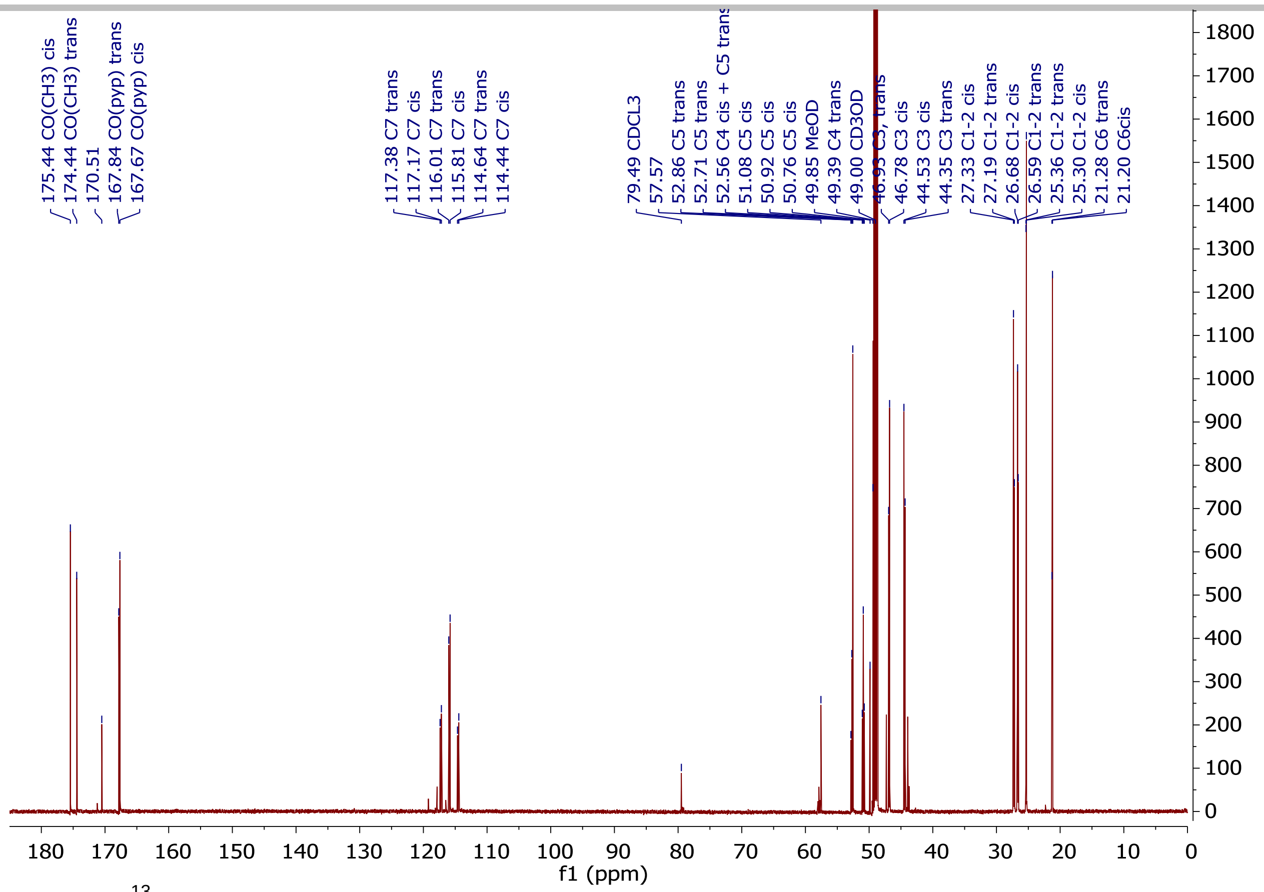

Figure S-58. ^{13}C NMR spectrum of compound **12** recorded in *methanol-d$_4$* at room temperature.

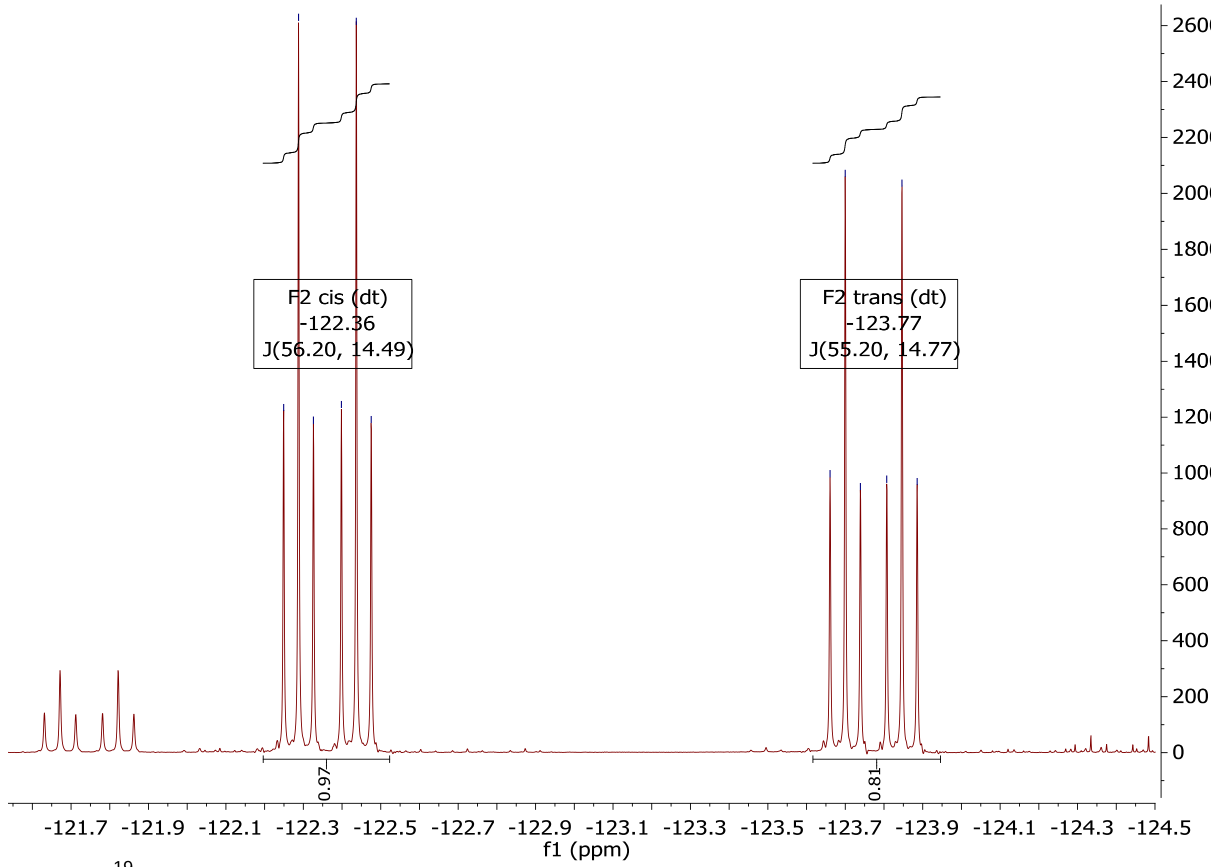

Figure S-59. ^{19}F-NMR spectrum of compound **12** recorded in *methanol-d$_4$* at room temperature.

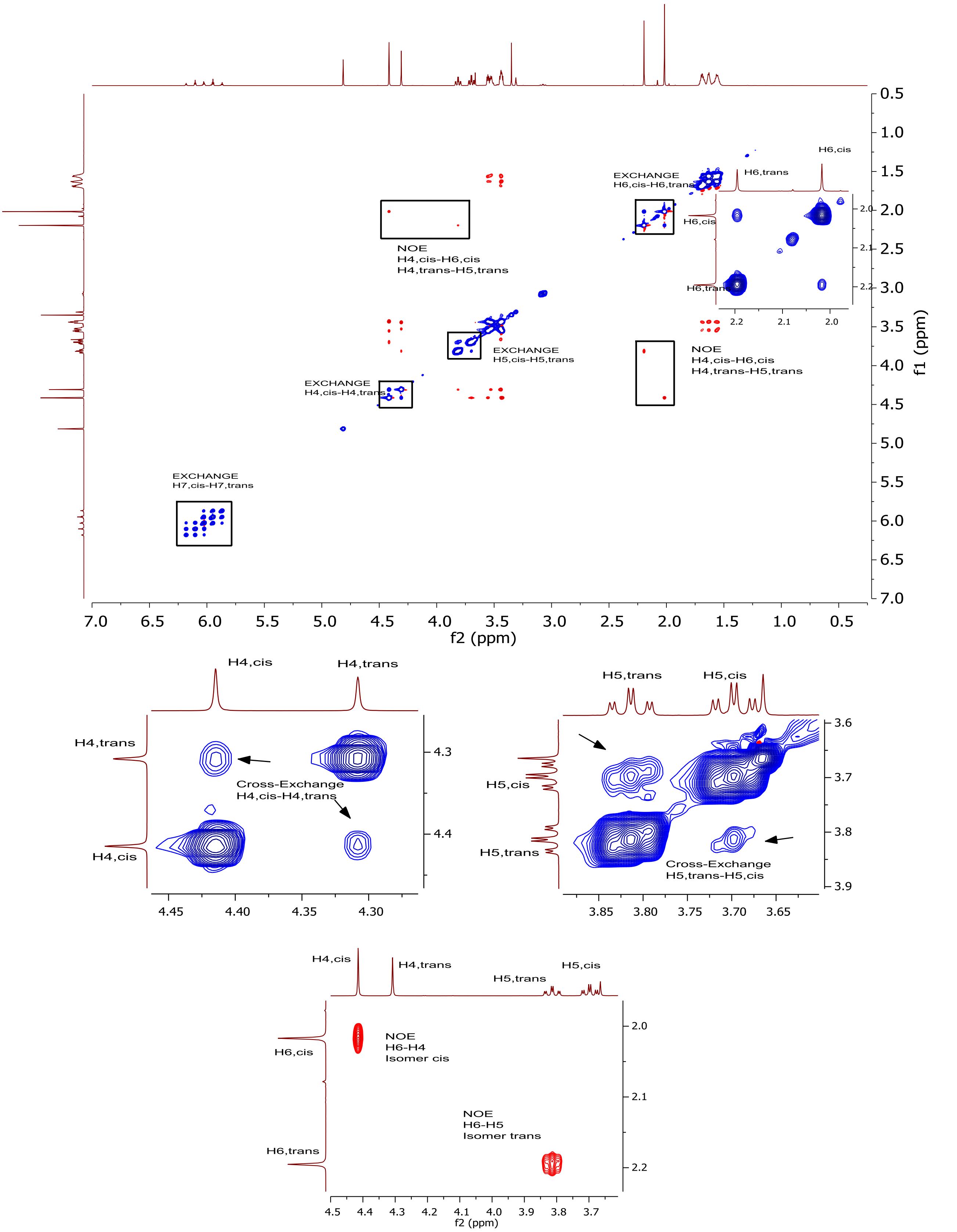

Figure S-60. ^{1}H-^{1}H NOESY spectrum of compound **12** recorded in *methanol-d$_4$* at room temperature. Inset showing the specific spatial contacts that are characteristic of each isomer and allowed the identification of *cis* and *trans* signals.

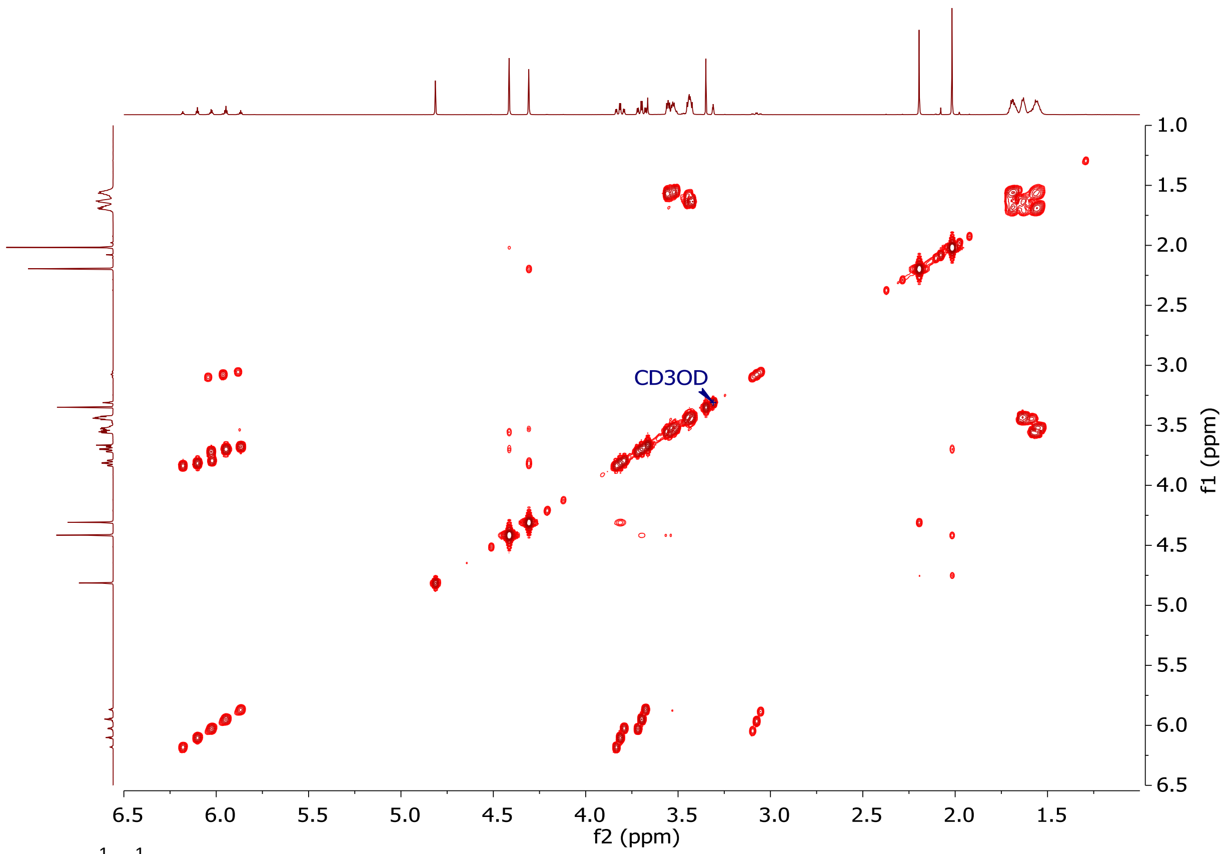

Figure S-61. ^{1}H-^{1}H COSY spectrum of compound **12** recorded in *methanol-d$_4$* at room temperature.

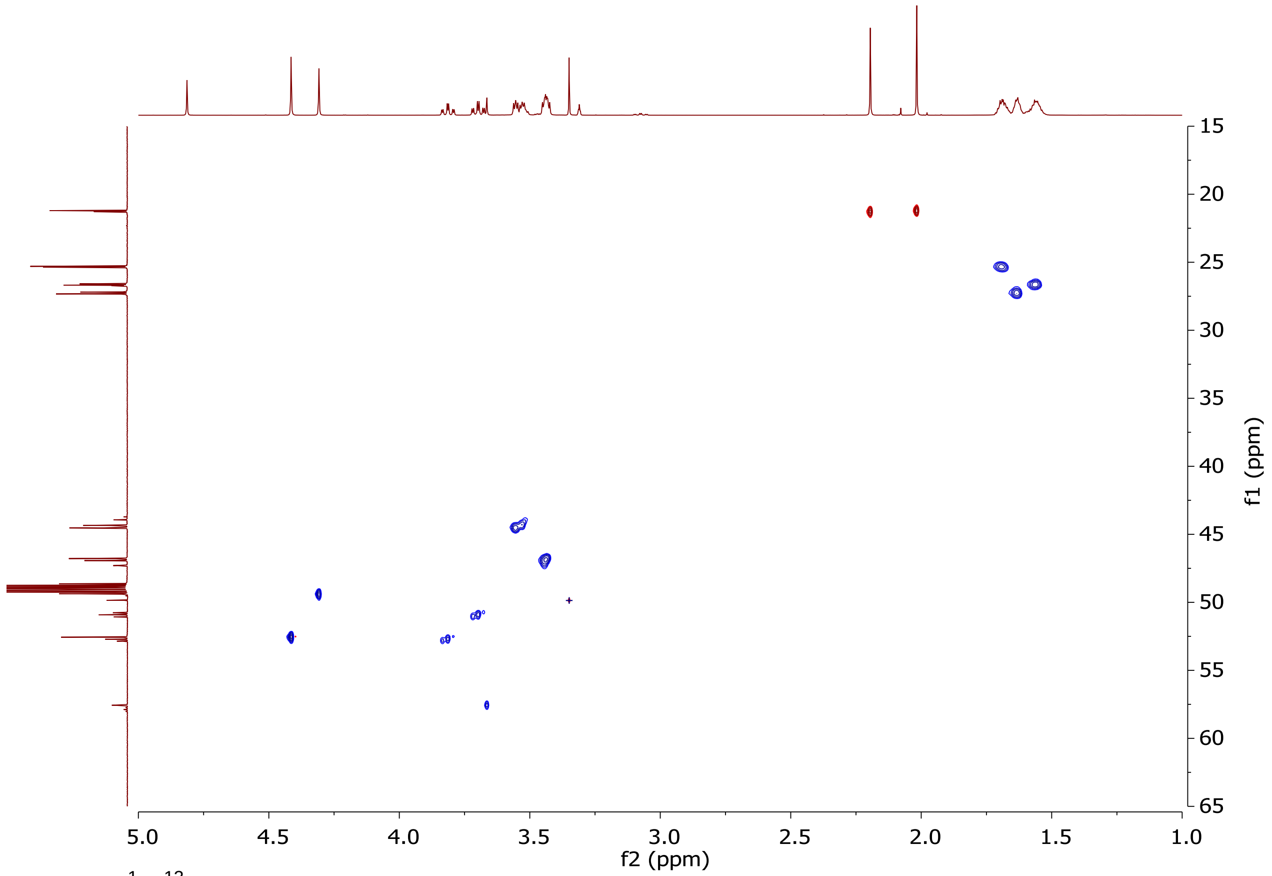

Figure S-62. ^{1}H-^{13}C HSQC spectrum of compound **12** recorded in *methanol-d$_4$* at room temperature.

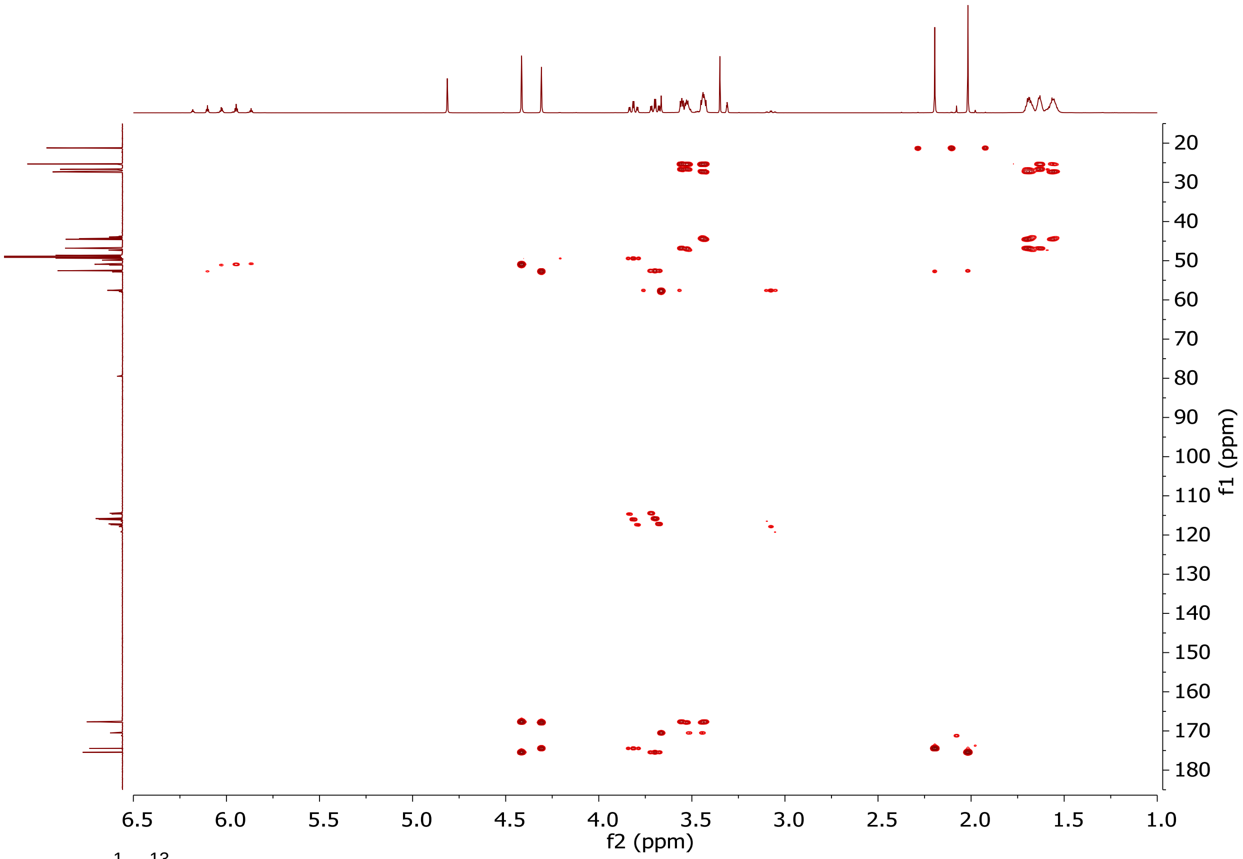

Figure S-63. ^{1}H-^{13}C HMBC spectrum of compound **12** recorded in *methanol-d$_4$* at room temperature.

^{1}H NMR (600 MHz, Chloroform-*d*) Rotamer *trans*: δ ^{1}H NMR: 5.92 (ddt, *J* = 55.1, 53.6, 3.6 Hz, 1H, $H_{7,trans}$), 4.16 (s, 2H, $H_{4,trans}$), 3.69 (td, *J* = 14.5, 3.5 Hz, 2H, $H_{5,trans}$), 3.46-3.28 (m, 4H, $H_{3,ax+eq,trans}$), 2.11 (s, 3H, $H_{6,trans}$), 1.64-1.41 (m, 6H, $H_{1-2,ax+eq,trans}$); Rotamer *cis*: δ ^{1}H NMR: 5.86 (ddt, *J* = 56.2, 54.2, 4.3 Hz, 1H, $H_{7,cis}$), 4.09 (s, 2H, $H_{4,cis}$), 3.60 (td, *J* = 14.4, 4.6 Hz, 2H, $H_{5,cis}$), 3.50-3.23 (m, 4H, $H_{3,ax+eq,cis}$), 1.94 (s, 3H, $H_{6,cis}$), 1.64-1.41 (m, 6H, $H_{1-2,\ ax+eq,cis}$).
^{13}C NMR (151 MHz, Chloroform-*d*) Rotamer *trans*: δ ^{13}C NMR: 114.30 (t, *J* = 243.0 Hz, CH, $C_{7,trans}$); 51.55 (t, *J* = 25.5 Hz, CH_2, $C_{5,trans}$), 47.73 (CH_2, $C_{4,trans}$), 45.81, 43.07 (CH_2, $C_{3,trans}$), 26.14, 25.34, 24.29 (CH_2, $C_{1-2,trans}$), 21.16 (CH_3, $C_{6,trans}$); Rotamer *cis*: δ ^{13}C NMR: 114.45 (t, *J* = 241.6 Hz, CH, $C_{7,cis}$), 51.29 (CH_2, $C_{4,cis}$), 49.78 (t, *J* = 26.9 Hz, CH_2, $C_{5,cis}$), 45.64, 43.37 (CH_2, $C_{3,cis}$), 26.31, 25.44, 24.21 (CH_2, $C_{1-2,cis}$), 21.08 (CH_3, $C_{6,cis}$); Quaternary carbons: δ ^{13}C NMR: 172.34 (**C**O-$CH_{3,cis}$), 171.45 (**C**O-$CH_{3,trans}$), 165.73 (**C**O-$pip_{,trans}$), 165.13 (**C**O-$pip_{,cis}$).
^{19}F NMR (564 MHz, Chloroform-*d*) Rotamer *trans*: δ ^{19}F NMR: -121.47 (dt, *J* = 55.0, 14.6 Hz); Rotamer *cis*: δ: -120.76 (dt, *J* = 56.2, 14.3 Hz).

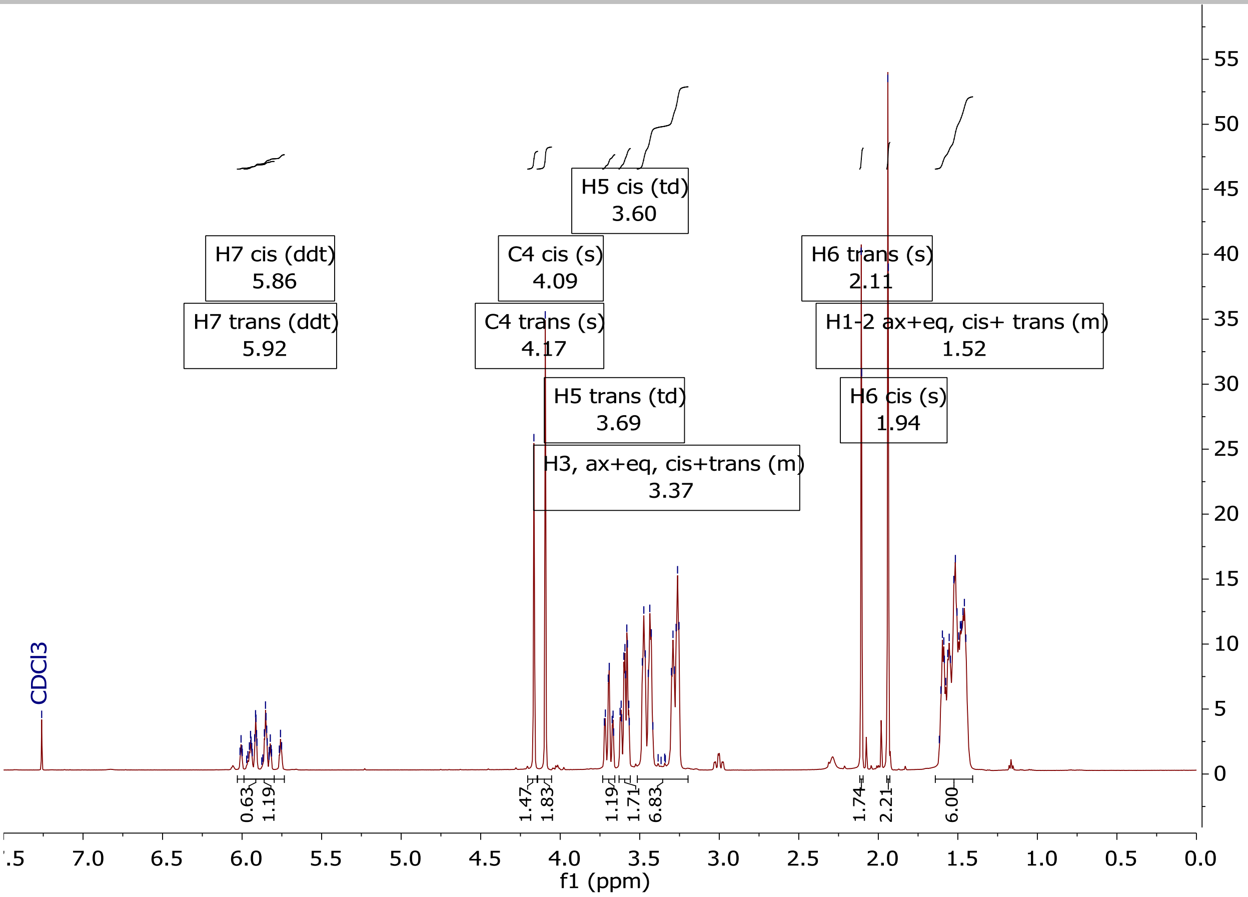

Figure S-64. 1H-NMR spectrum of compound **12** recorded in chloroform-*d* at room temperature.

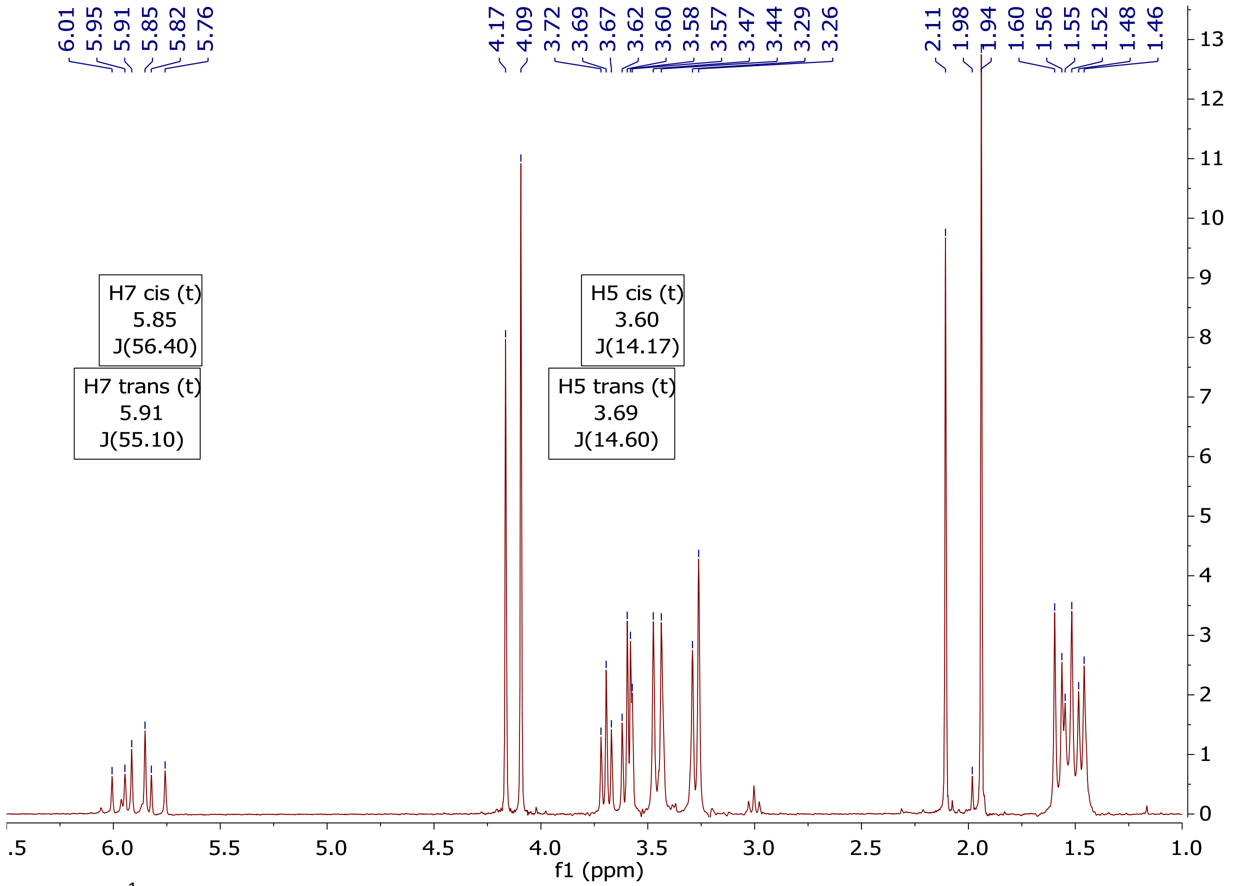

Figure S-65. ^{1}H-*psyche* spectrum of compound **12** recorded in chloroform-*d* at room temperature.

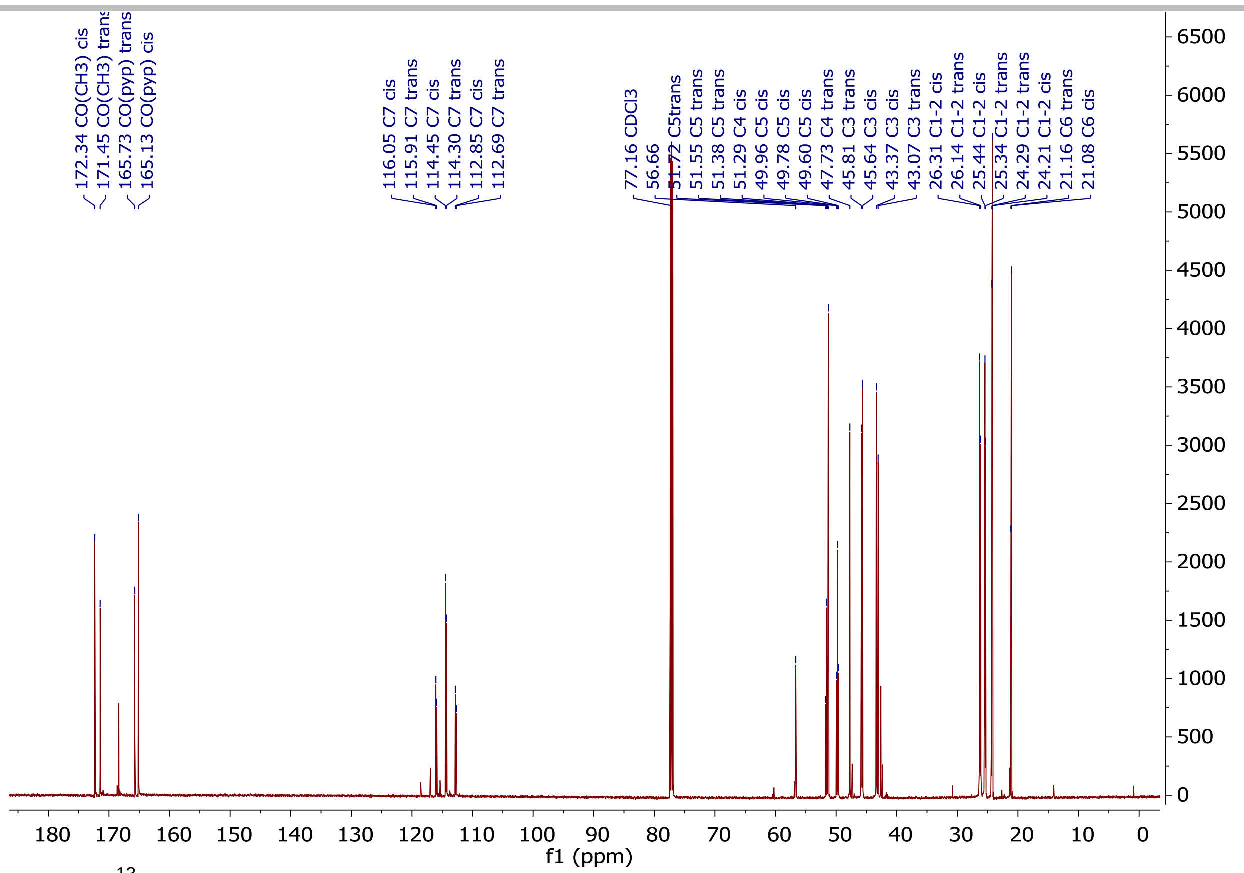

Figure S-66. ^{13}C NMR spectrum of compound **12** recorded in chloroform-*d* at room temperature.

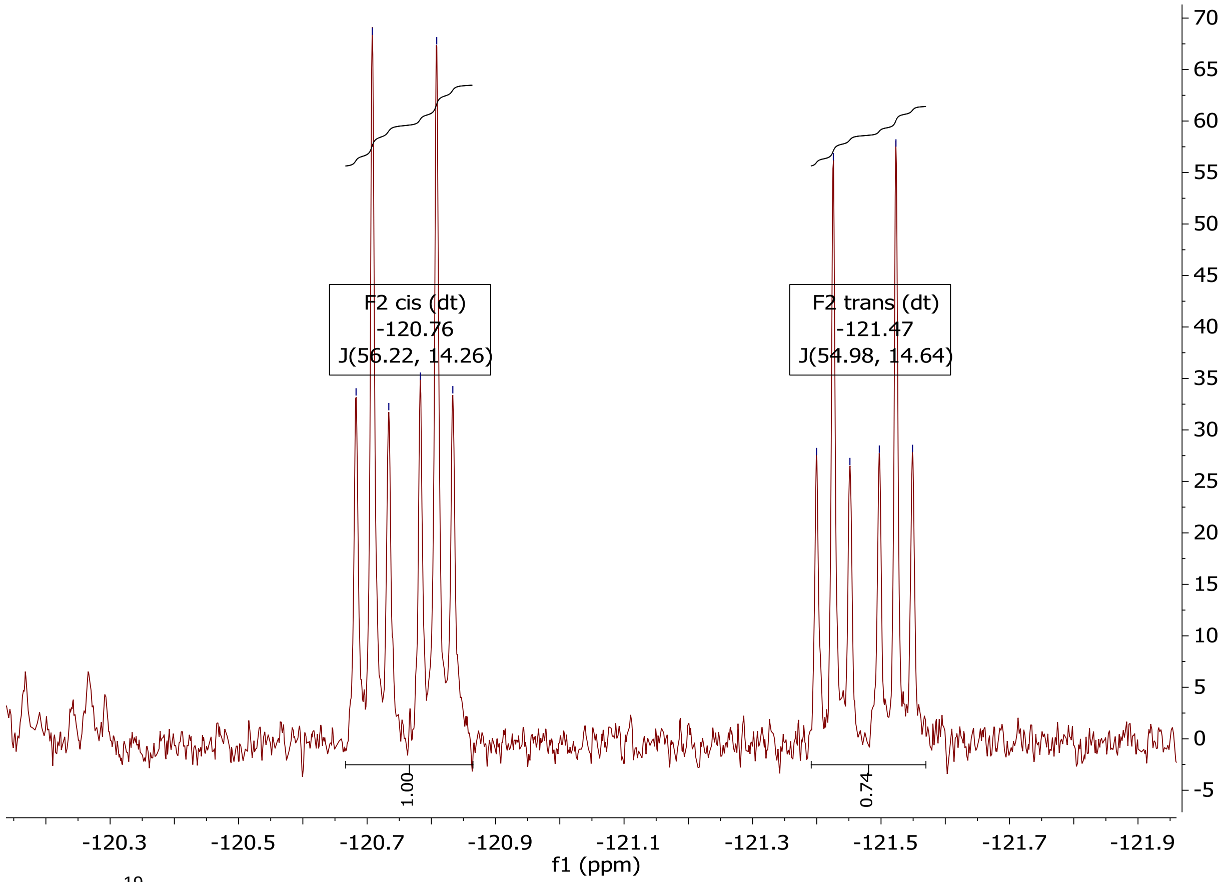

Figure S-67. ^{19}F-NMR spectrum of compound **12** recorded in chloroform-*d* at room temperature.

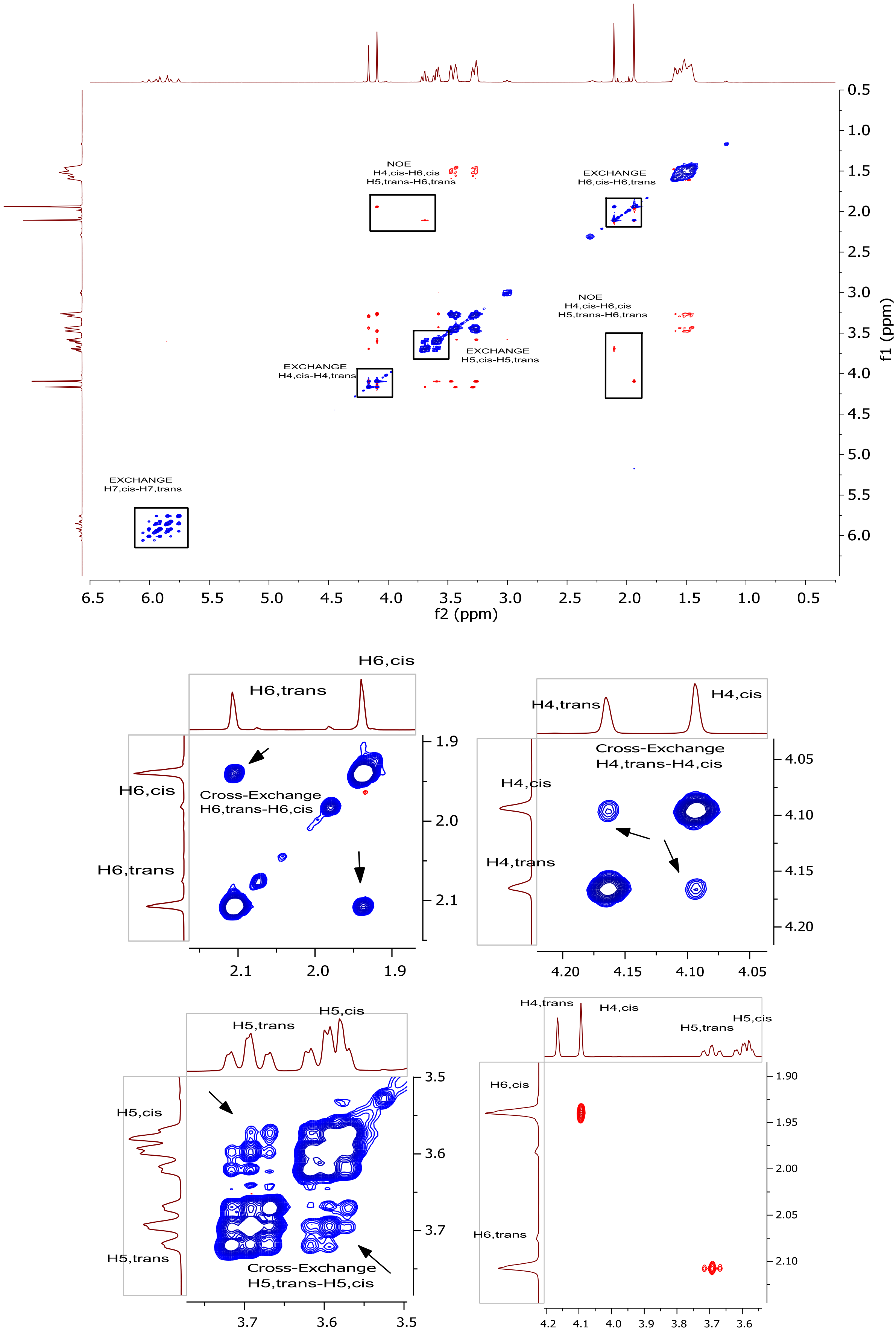

Figure S-68. ^{1}H-^{1}H NOESY spectrum of compound **12** recorded in chloroform-*d* at room temperature. Insets showing the specific spatial contacts that are characteristic of each isomer and allowed the identification of *cis* and *trans* signals.

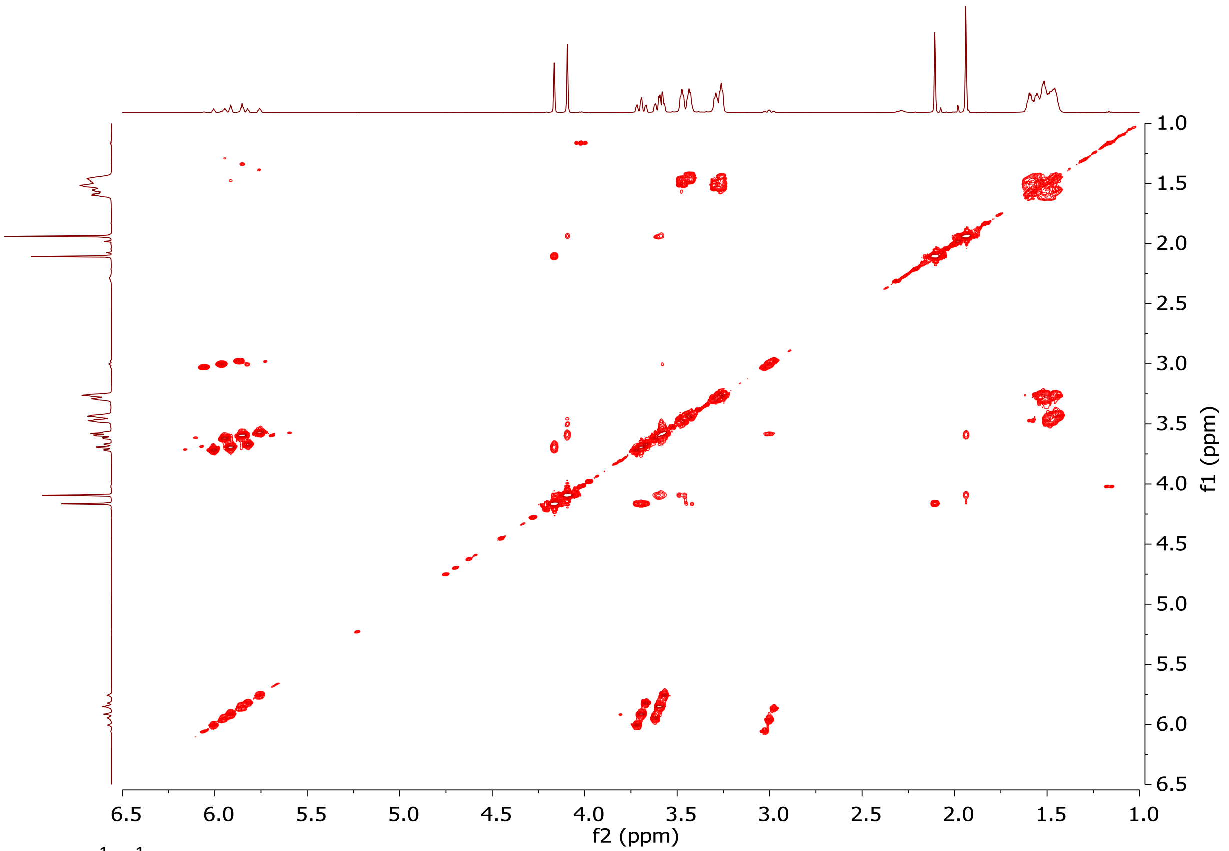

Figure S-69. ^{1}H-^{1}H COSY spectrum of compound **12** recorded in chloroform-*d* at room temperature.

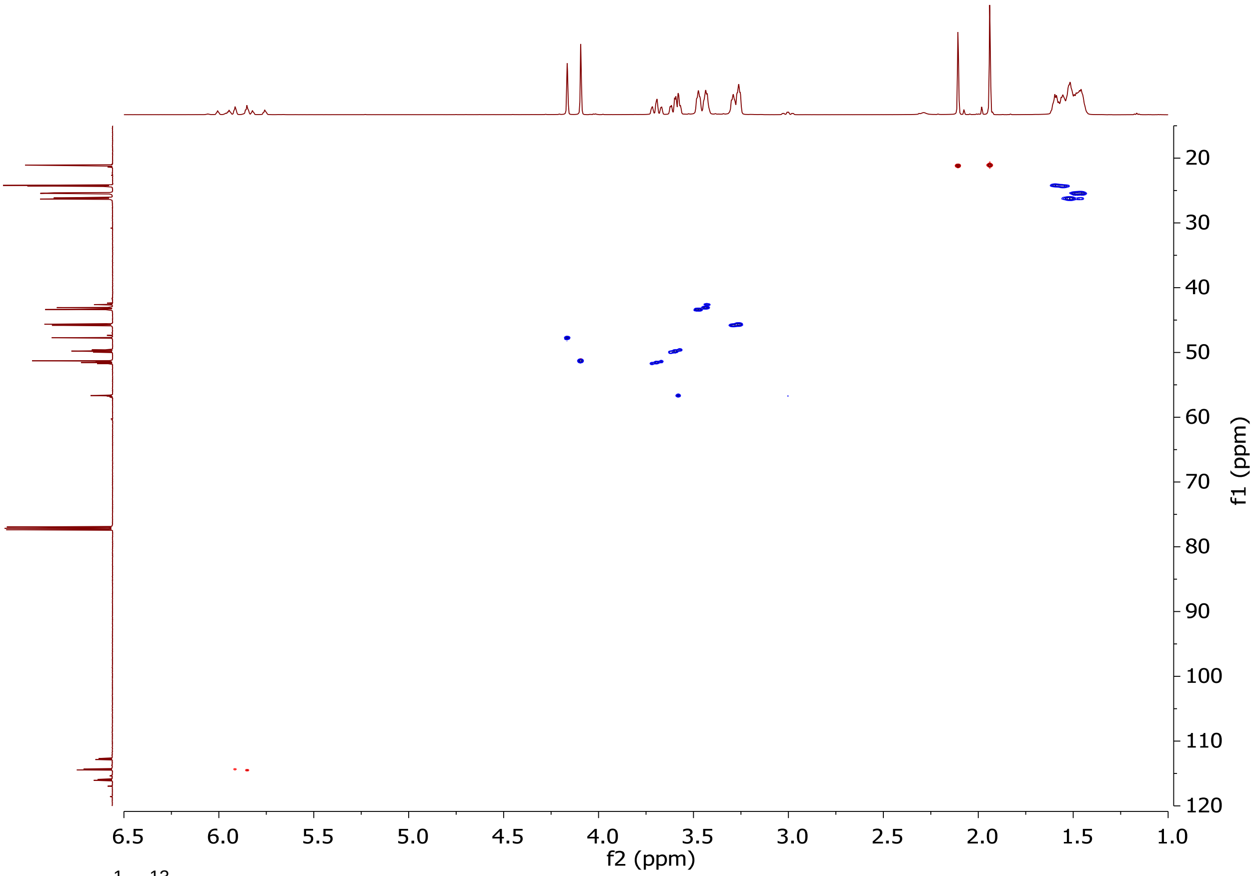

Figure S-70. ^{1}H-^{13}C HSQC spectrum of compound **12** recorded in chloroform-*d* at room temperature

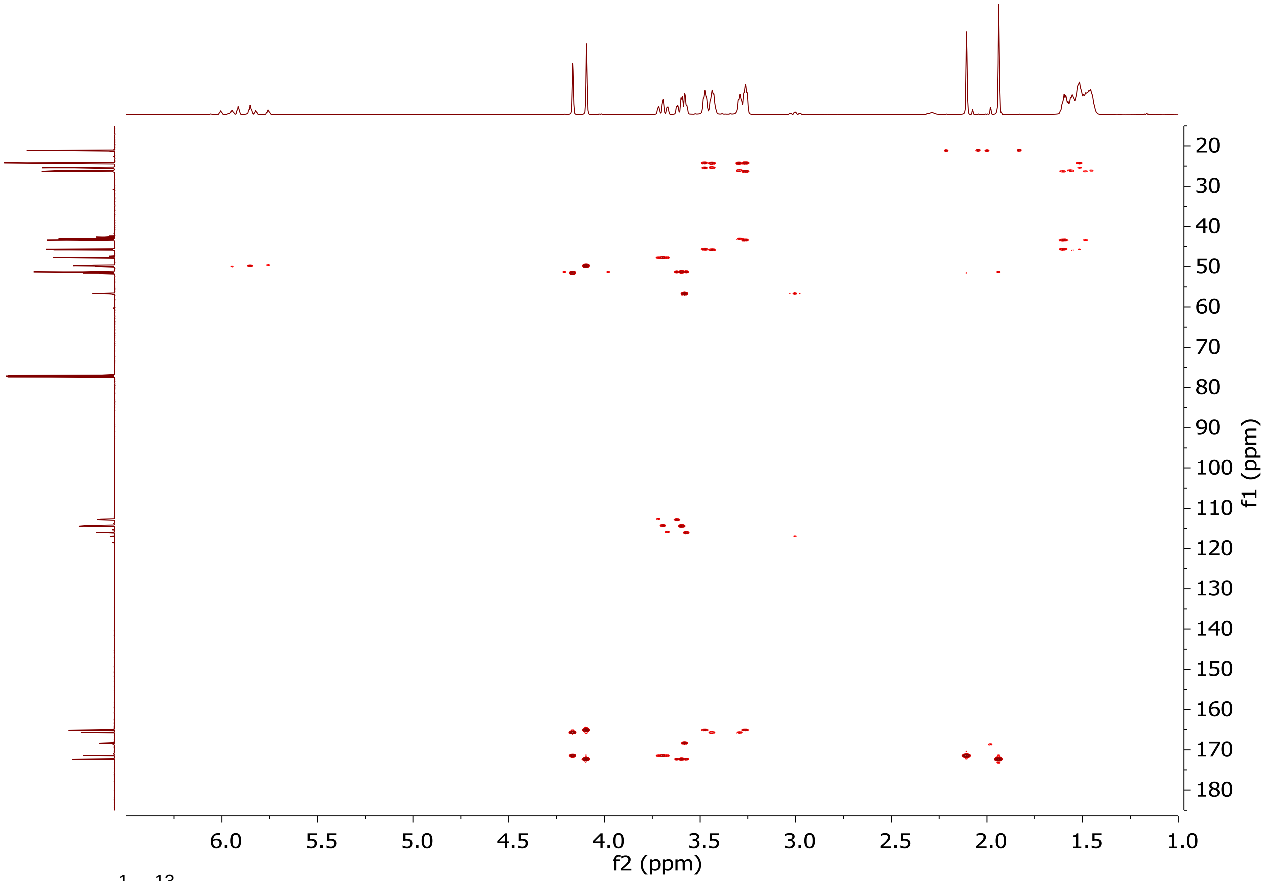

Figure S-71. ^{1}H-^{13}C HMBC spectrum of compound **12** recorded in chloroform-*d* at room temperature.

13: Ac-^{3f}Et-pip.

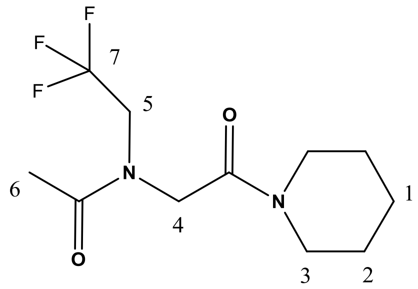

HRMS: calculated for $C_{11}H_{18}F_3N_2O_2$ 267.1320 [M+H]+, found: 267.1344 Da.

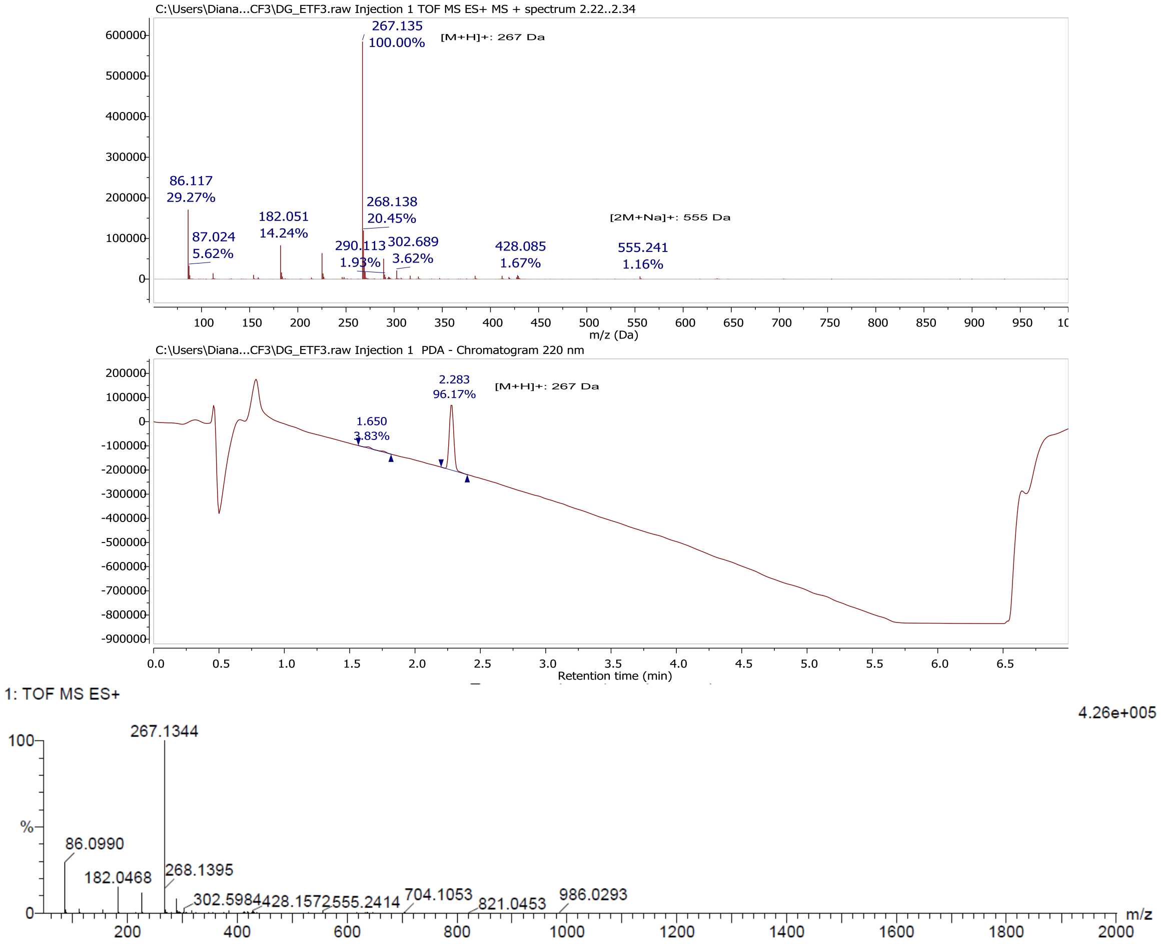

Figure S-72. Structure, HR (QToF)-LC/MS trace and elemental composition of model piperinidyl acetamide **13**.

^{1}H NMR (700 MHz, Acetonitrile-*d*$_3$) <u>Rotamer *trans*</u>: δ ^{1}H NMR: 4.20 (s, 2H, $H_{4,trans}$), 4.05 (q, *J* = 9.1 Hz, 2H, $H_{5,trans}$), 3.47-3.31 (m, 4H, $H_{3,ax+eq,trans}$), 2.12 (s, 3H, $H_{6,trans}$), 1.67-1.41 (m, 6H, $H_{1\text{-}2,ax+eq,trans}$); <u>Rotamer *cis*</u>: δ ^{1}H NMR: 4.25 (s, 2H, $H_{4,cis}$), 3.99 (q, *J* = 9.5 Hz, 2H, $H_{5,cis}$), 3.51-3.31 (m, 4H, $H_{3,ax+eq,cis}$), 1.94 (s, 3H, $H_{6,cis}$), 1.67-1.41 (m, 6H, $H_{1\text{-}2,\ ax+eq,cis}$).
^{13}C NMR (151 MHz, Acetonitrile-*d*$_3$) <u>Rotamer *trans*</u>: δ ^{13}C NMR: 50.92 (q, *J* = 32.6 Hz, CH_2, $C_{5,trans}$), 48.60 (CH_2, $C_{4,trans}$), 46.26, 43.61 (CH_2, $C_{3,trans}$), 26.94, 26.35, 25.13 (CH_2, $C_{1\text{-}2,trans}$), 21.43 (CH_3, $C_{6,trans}$); <u>Rotamer *cis*</u>: δ ^{13}C NMR: 51.74 (CH_2, $C_{4,cis}$), 47.58 (q, *J* = 33.1 Hz, CH_2, $C_{5,cis}$), 46.24, 43.88 (CH_2, $C_{3,cis}$), 26.95, 26.38, 25.07 (CH_2, $C_{1\text{-}2,cis}$), 21.45 (CH_3, $C_{6,cis}$); <u>Quaternary carbons</u>: δ ^{13}C NMR: 126.17 (q, *J* = 279.5, $C_{7,cis}$), 126.02 (q, *J* = 280.0 Hz, $C_{7,trans}$), 173.60 (**C**O-$CH_{3,cis}$), 172.48 (**C**O-$CH_{3,trans}$), 166.36 (**C**O-$pip_{,cis}$), 166.31 (**C**O-$pip_{,trans}$).
^{19}F NMR (376 MHz, Chloroform-*d*) <u>Rotamer *trans*</u>: δ ^{19}F NMR: -71.72 (t, *J* = 9.0 Hz); <u>Rotamer *cis*</u>: δ: -71.30 (t, *J* = 9.6 Hz).

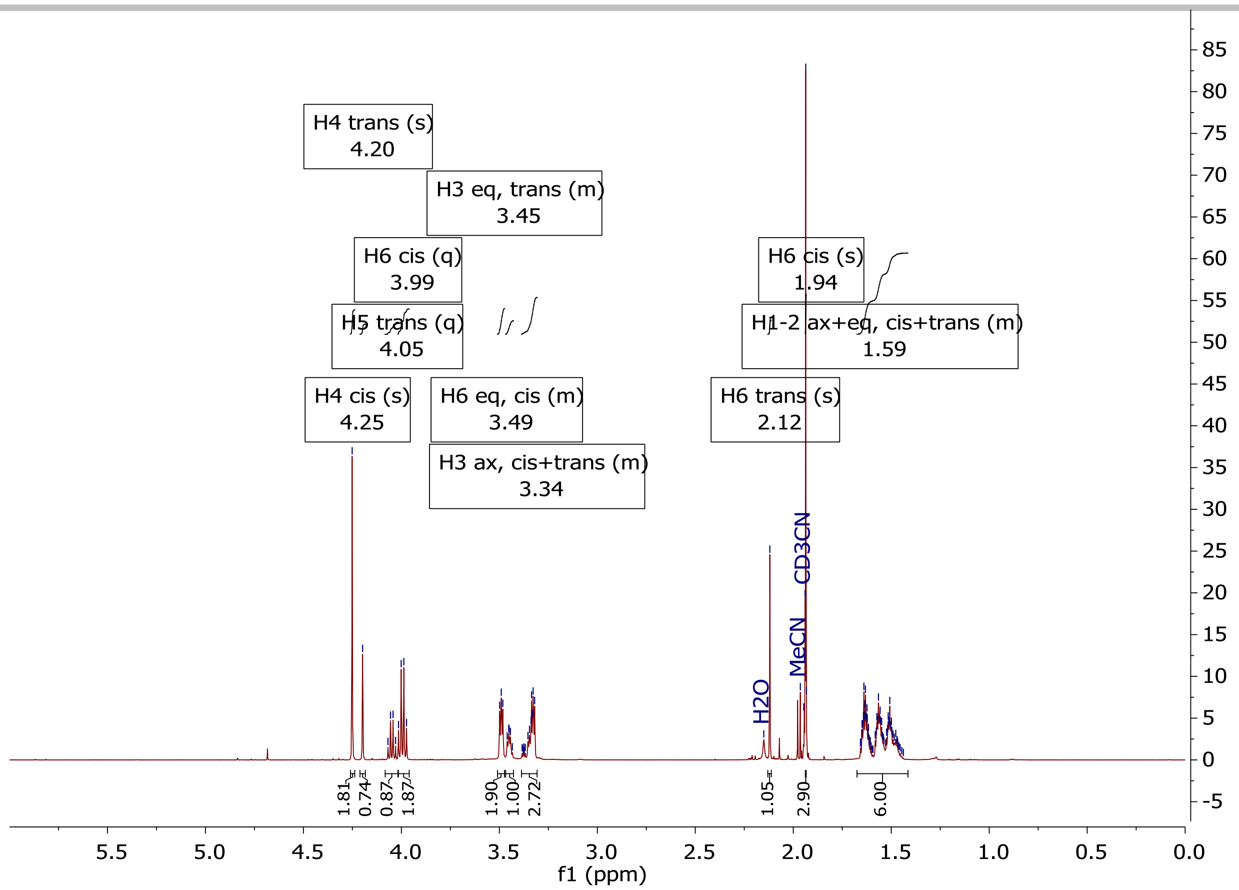

Figure S-73. 1H-NMR spectrum of compound **13** recorded in *acetonitrile-d*$_3$ at room temperature.

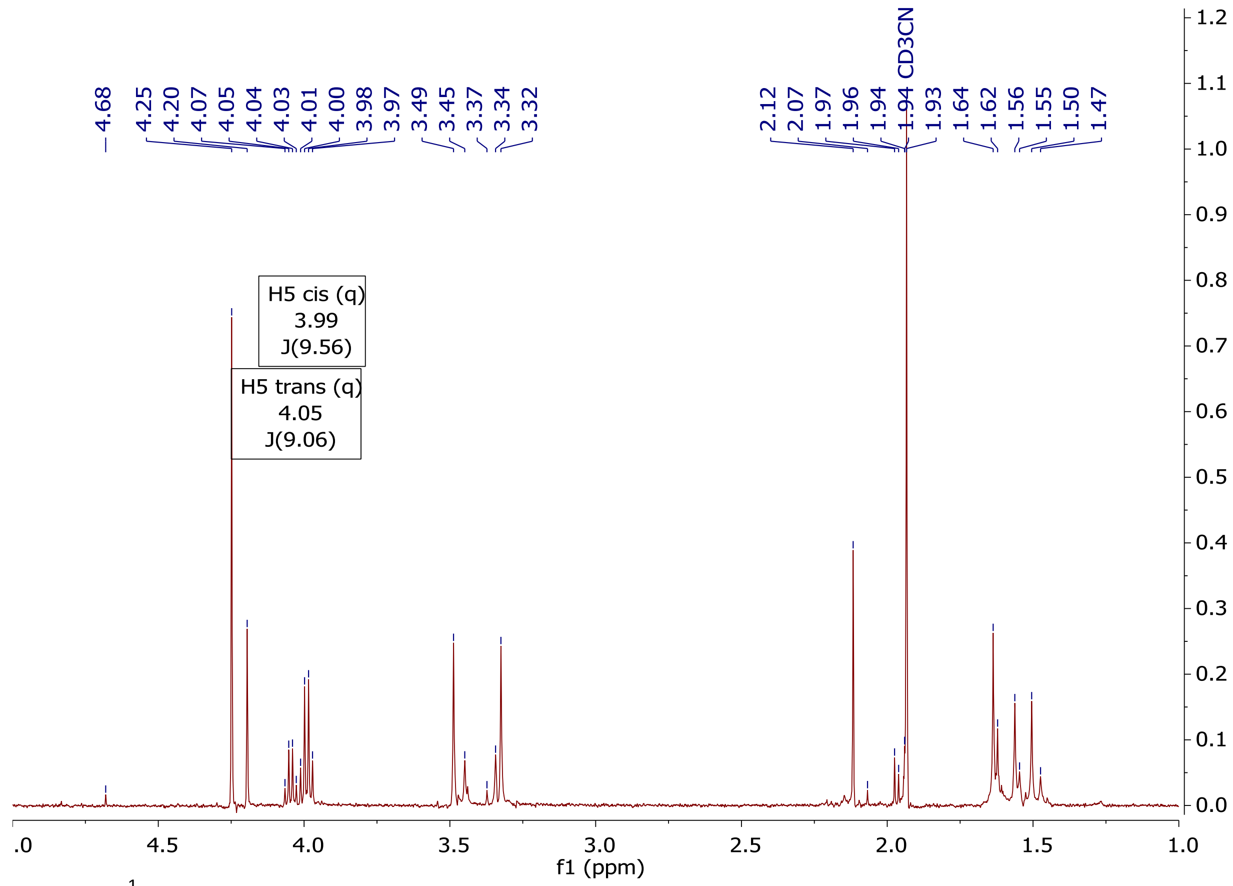

Figure S-74. ^{1}H-*psyche* spectrum of compound **13** recorded in *acetonitrile-d*$_3$ at room temperature.

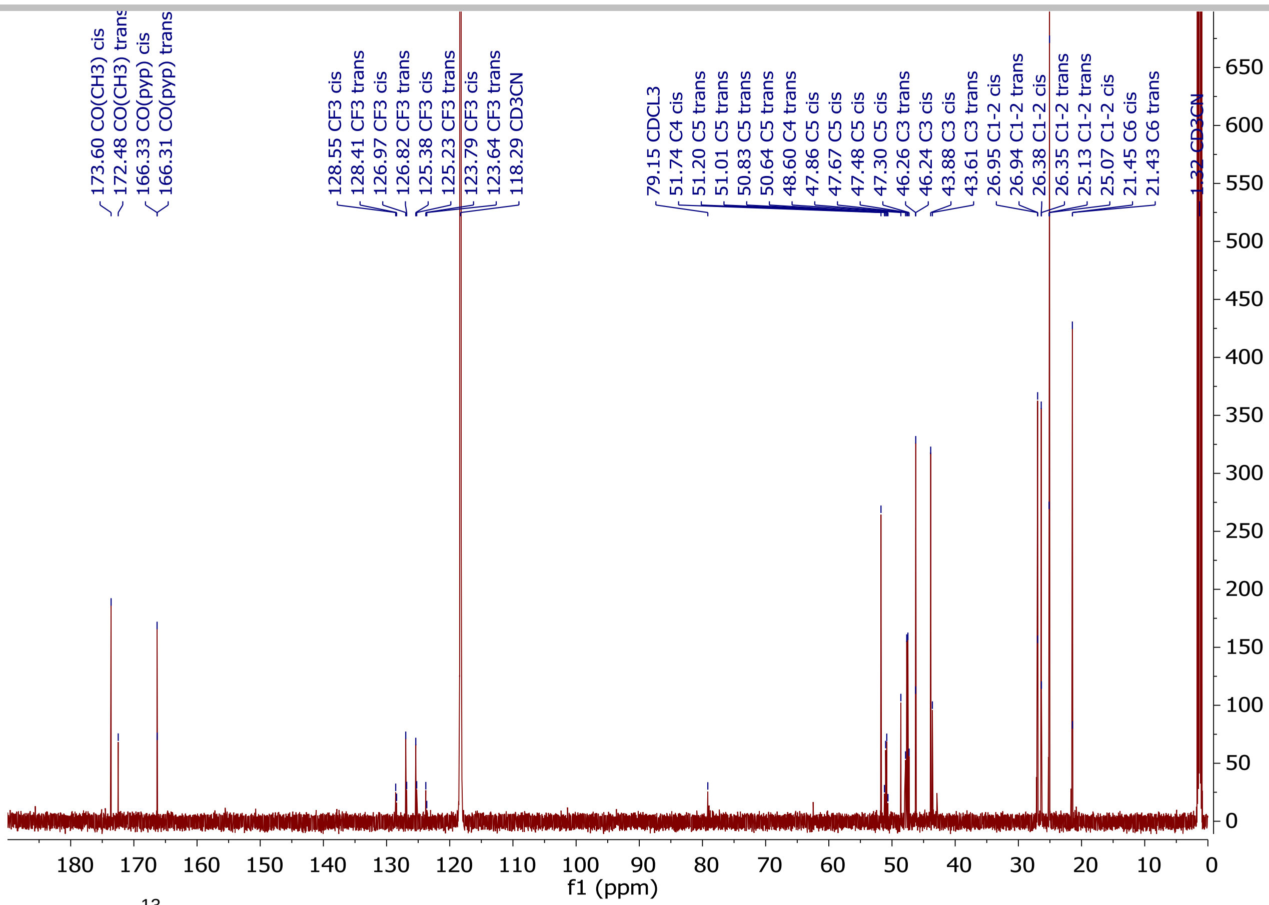

Figure S-75. ^{13}C NMR spectrum of compound **13** recorded in *acetonitrile-d_3* at room temperature.

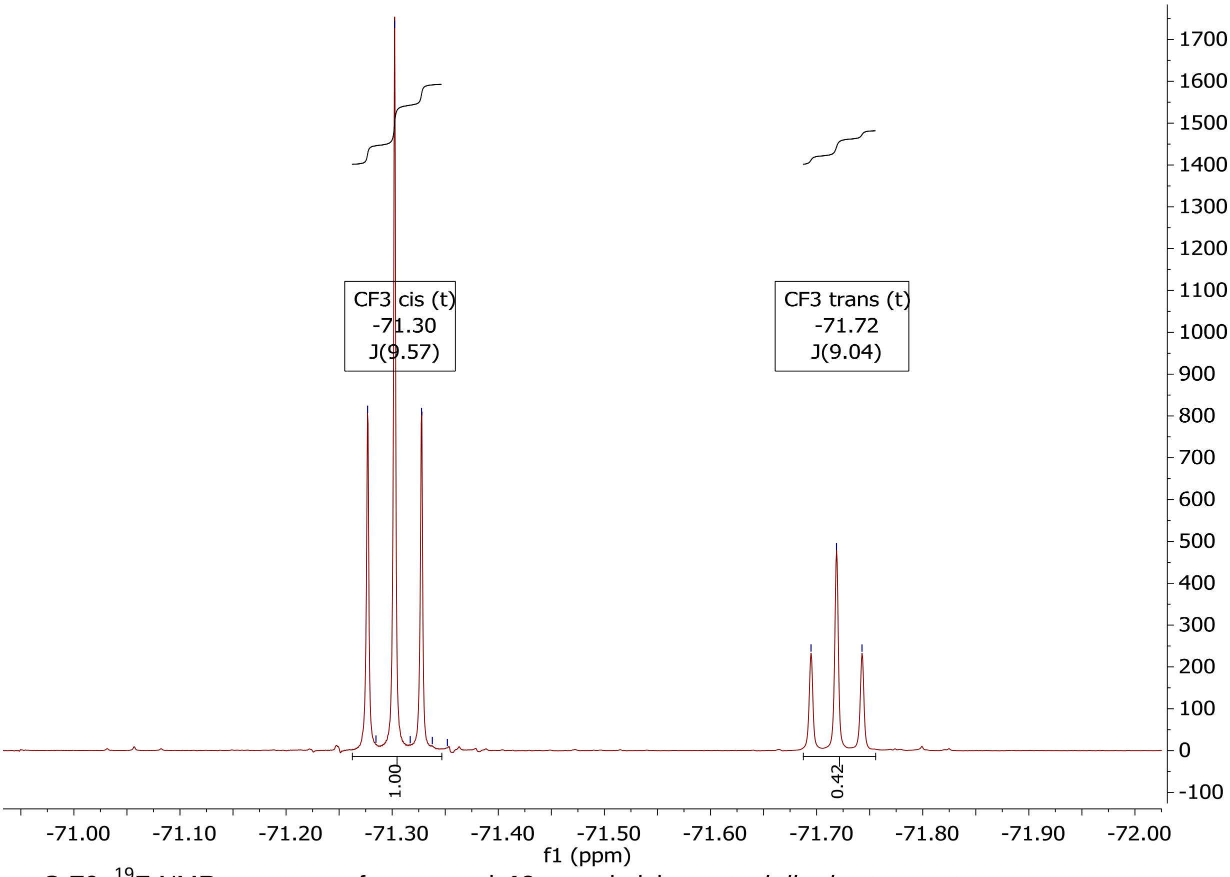

Figure S-76. ^{19}F-NMR spectrum of compound **13** recorded in *acetonitrile-d_3* at room temperature.

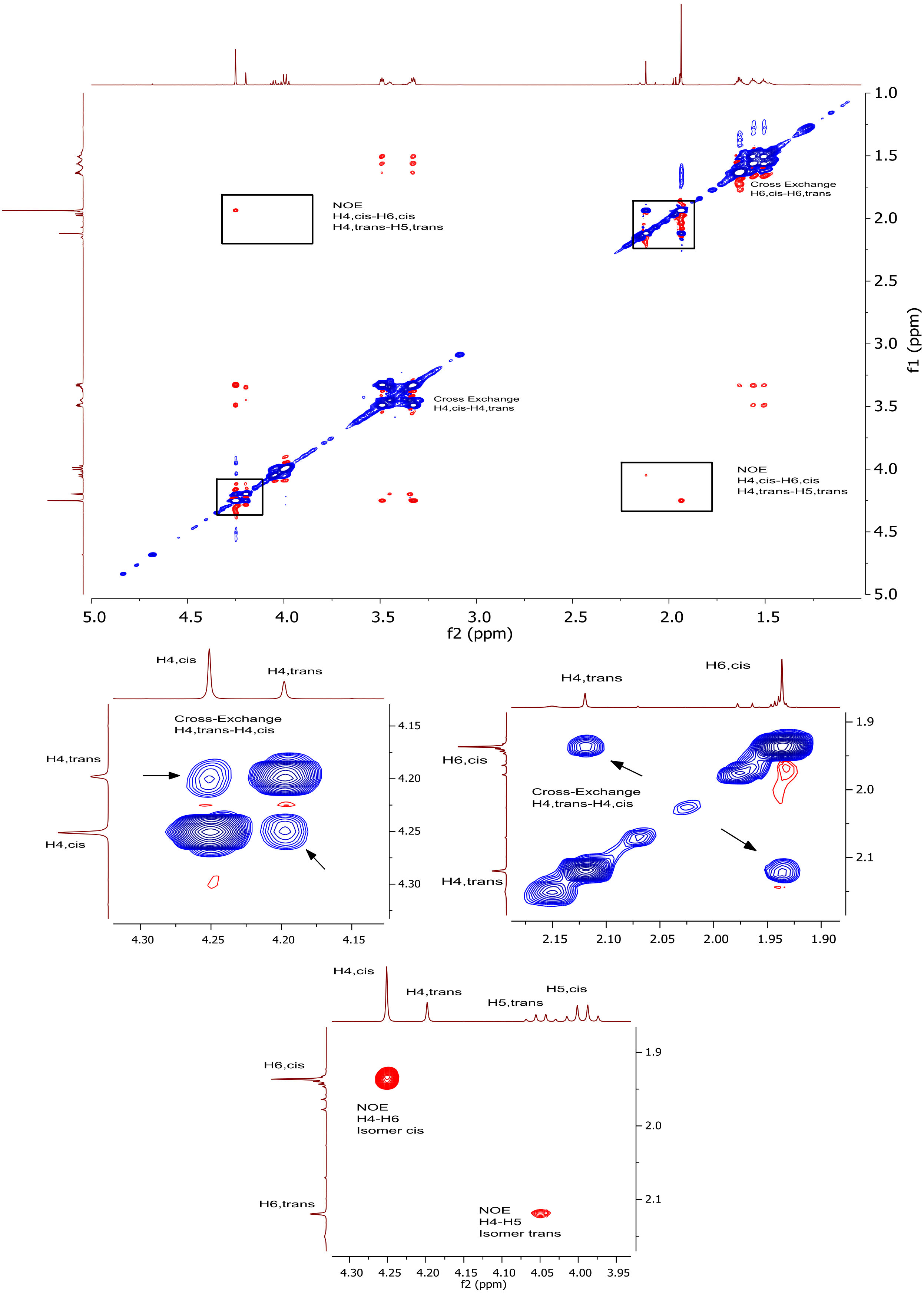

Figure S-77. ^{1}H-^{1}H NOESY spectrum of compound **13** recorded in *acetonitrile-d$_3$* at room temperature. Inset showing the specific spatial contacts that are characteristic of each isomer and allowed the identification of *cis* and *trans* signals.

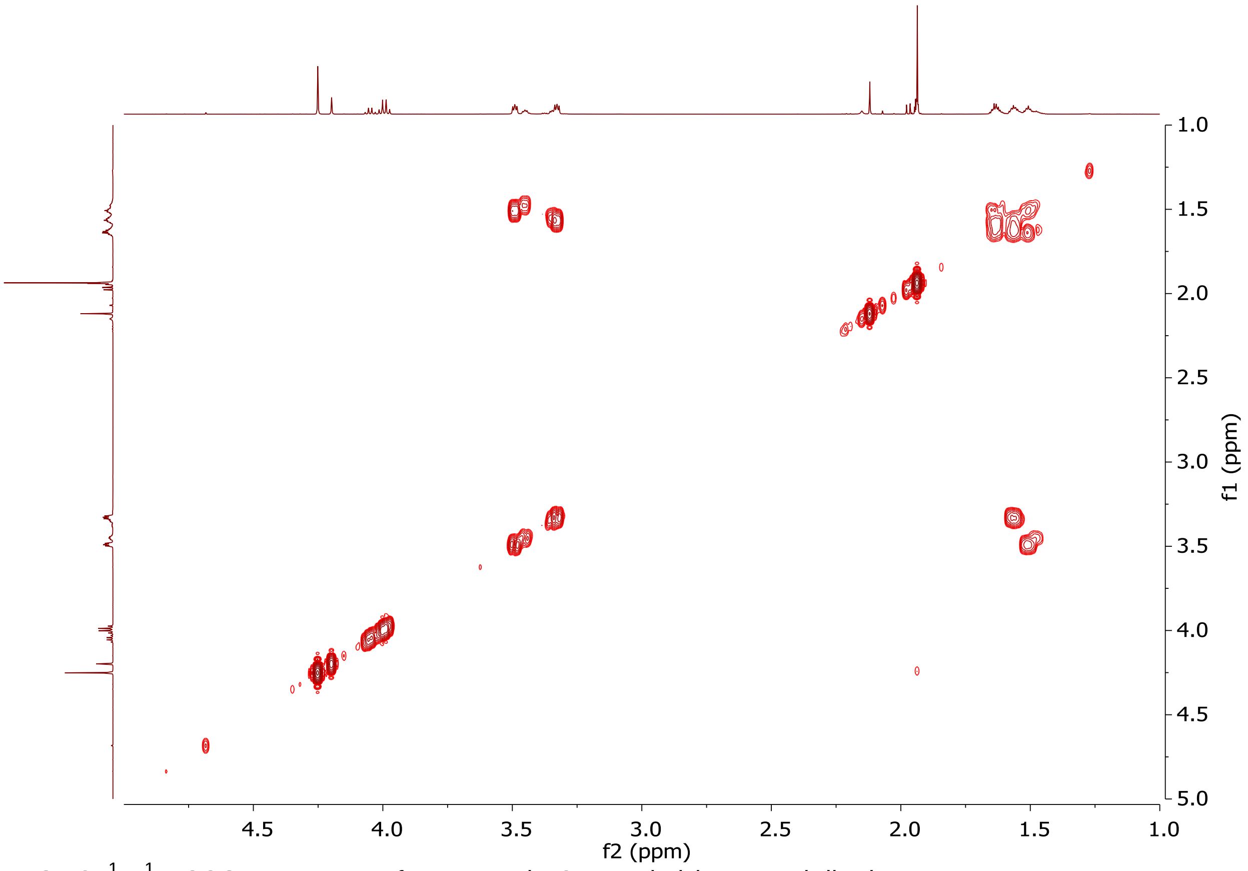

Figure S-78. ^{1}H-^{1}H COSY spectrum of compound **13** recorded in *acetonitrile-d$_3$* at room temperature.

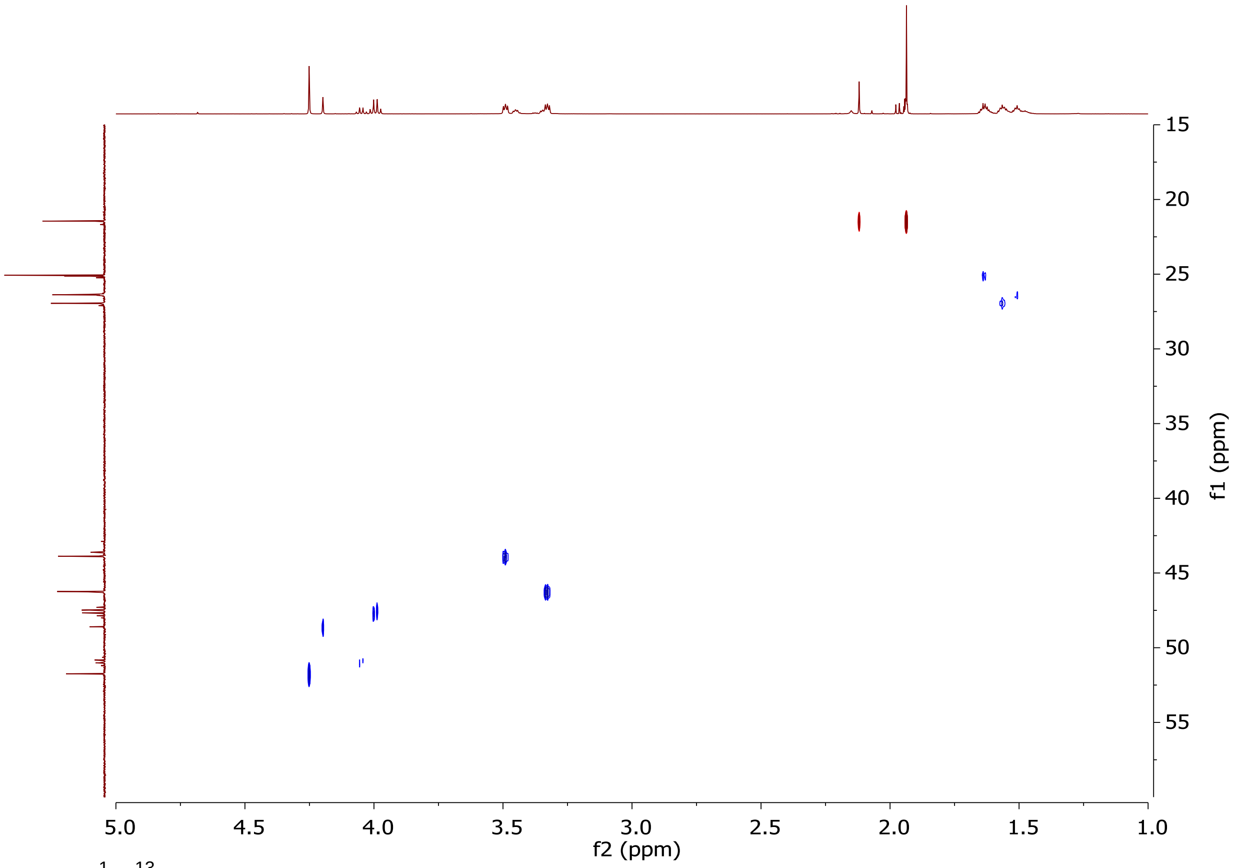

Figure S-79. ^{1}H-^{13}C HSQC spectrum of compound **13** recorded in *acetonitrile-d$_3$* at room temperature

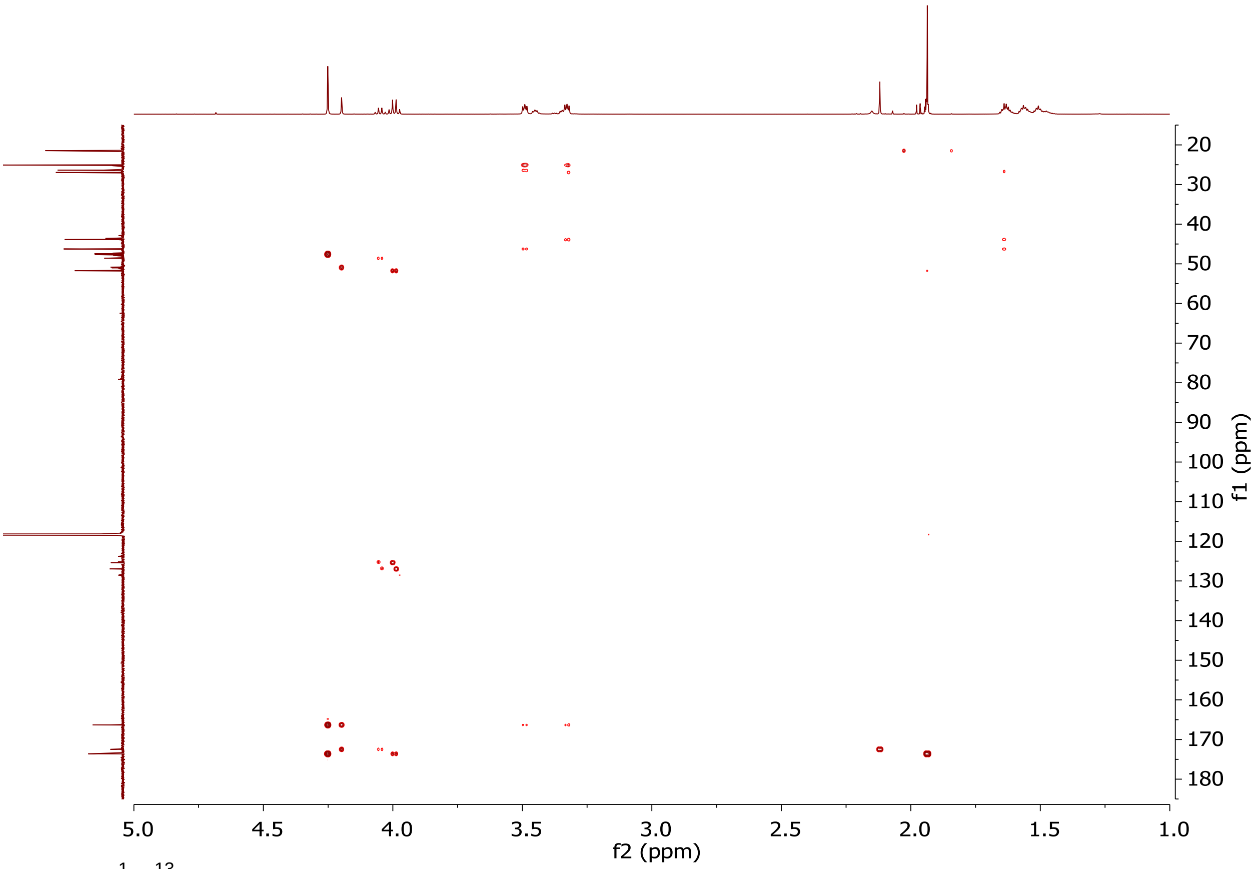

Figure S-80. ^{1}H-^{13}C HMBC spectrum of compound **13** recorded in *acetonitrile-d$_3$* at room temperature.

^{1}H NMR (700 MHz, Methanol-*d$_4$*) Rotamer *trans*: δ ^{1}H NMR: 4.31 (s, 2H, $H_{4,trans}$), 4.19 (q, *J* = 8.9 Hz, 2H, $H_{5,trans}$), 3.54-3.39 (m, 4H, $H_{3,ax+eq,trans}$), 2.21 (s, 3H, $H_{6,trans}$), 1.75-1.49 (m, 6H, $H_{1\text{-}2,ax+eq,trans}$); Rotamer *cis*: δ ^{1}H NMR: 4.45 (s, 2H, $H_{4,cis}$), 4.08 (q, *J* = 9.3 Hz, 2H, $H_{5,cis}$), 3.58-3.39 (m, 4H, $H_{3,ax+eq,cis}$), 2.03 (s, 3H, $H_{6,cis}$), 1.75-1.49 (m, 6H, $H_{1\text{-}2,\ ax+eq,cis}$).
^{13}C NMR (176 MHz, Methanol-*d$_4$*) Rotamer *trans*: δ ^{13}C NMR: 51.35 (q, *J* = 33.0 Hz, CH_2, $C_{5,trans}$), 49.85 (CH_2, $C_{4,trans}$), 46.94, 44.41 (CH_2, $C_{3,trans}$), 27.19, 26.59, 25.38 (CH_2, $C_{1\text{-}2,trans}$), 21.11 (CH_3, $C_{6,trans}$); Rotamer *cis*: δ ^{13}C NMR: 52.00 (CH_2, $C_{4,cis}$), 48.15 (q, *J* = 33.5 Hz, CH_2, $C_{5,cis}$), 46.79, 44.56 (CH_2, $C_{3,cis}$), 27.32, 26.68, 25.31 (CH_2, $C_{1\text{-}2,cis}$), 21.14 (CH_3, $C_{6,cis}$); Quaternary carbons: δ ^{13}C NMR: 126.21 (q, *J* = 279.5, $C_{7,cis}$), 126.17 (d, *J* = 279.8 Hz, $C_{7,trans}$), 175.49 (**C**O-$CH_{3,cis}$), 174.66 (**C**O-$CH_{3,trans}$), 167.50 (**C**O-$pip_{,trans}$), 167.40 (**C**O-$pip_{,cis}$).
^{19}F NMR (376 MHz, Methanol-*d$_4$*) Rotamer *trans*: δ ^{19}F NMR: -72.46 (t, *J* = 8.9 Hz); Rotamer *cis*: δ: -72.01 (t, *J* = 9.3 Hz).

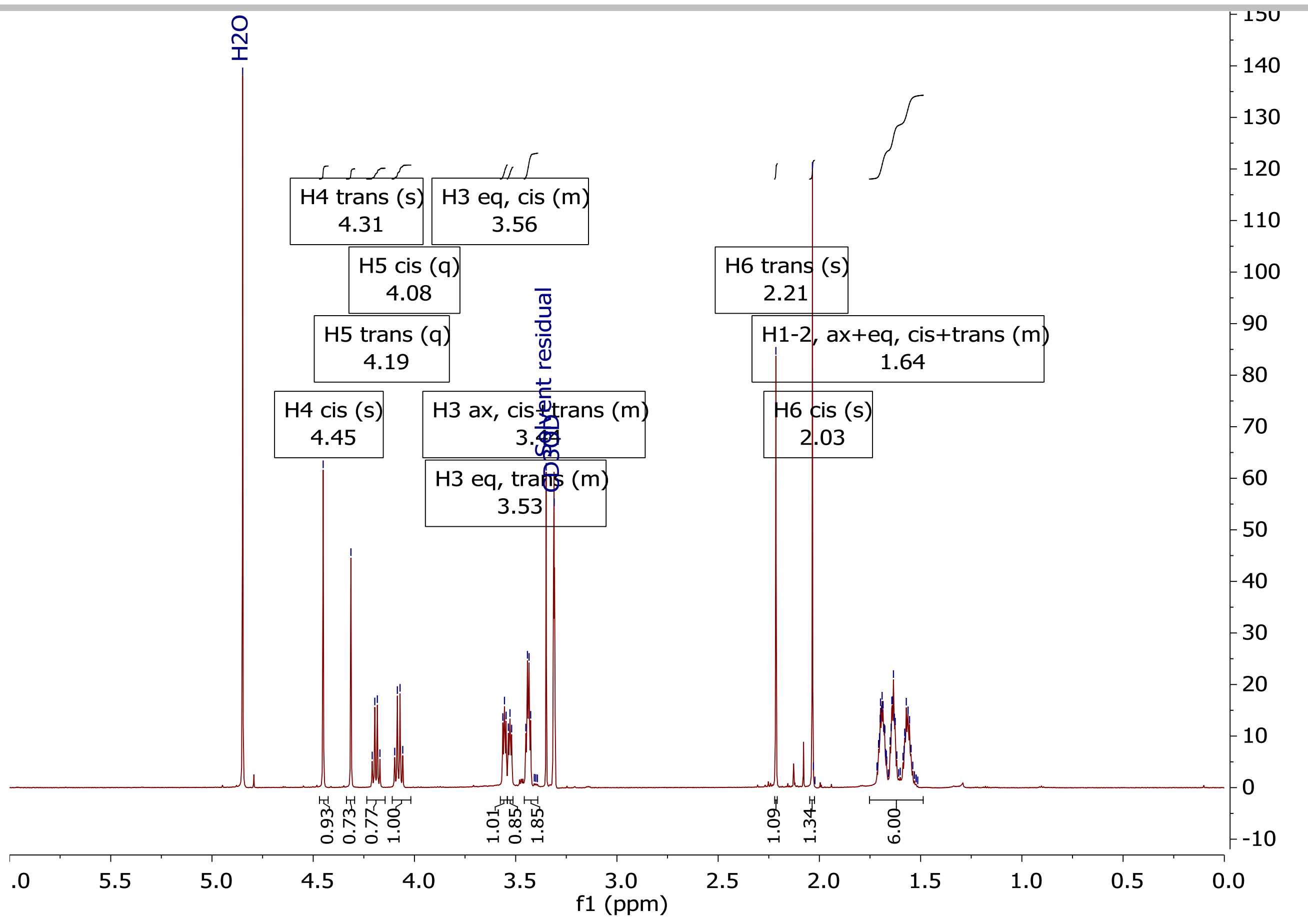

Figure S-81. 1H-NMR spectrum of compound **13** recorded in *methanol-d_4* at room temperature.

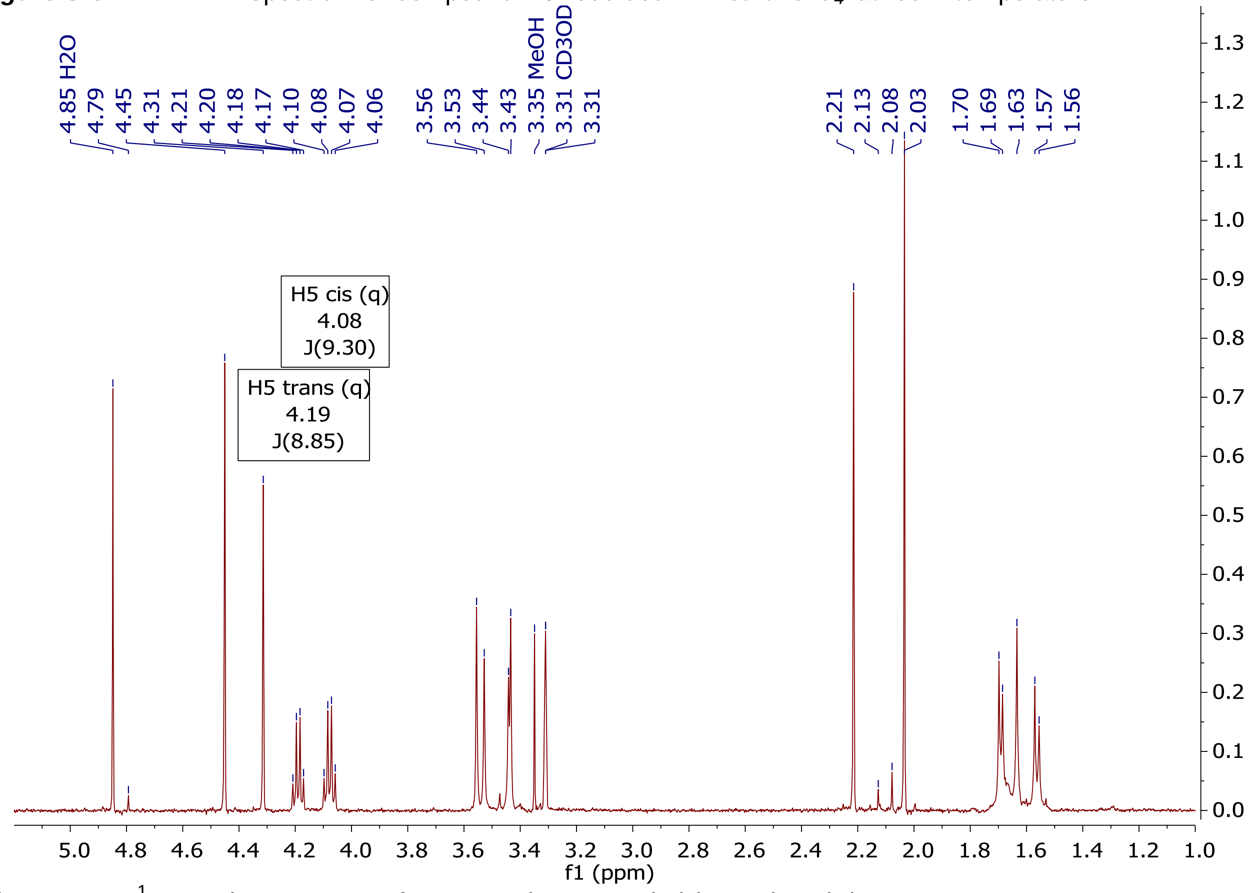

Figure S-82. ^{1}H-*psyche* spectrum of compound **13** recorded in *methanol-d_4* at room temperature.

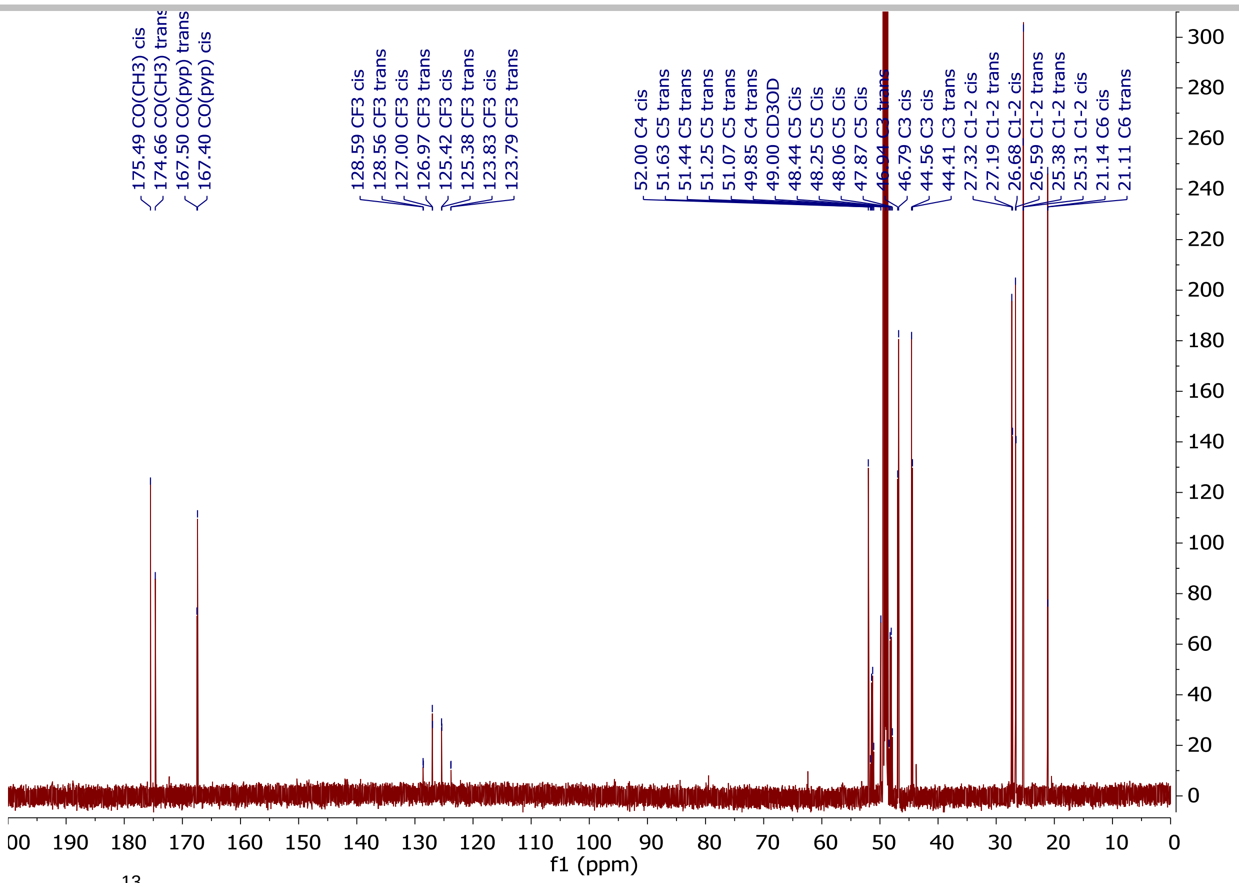

Figure S-83. ^{13}C NMR spectrum of compound **13** recorded in *methanol-d$_4$* at room temperature.

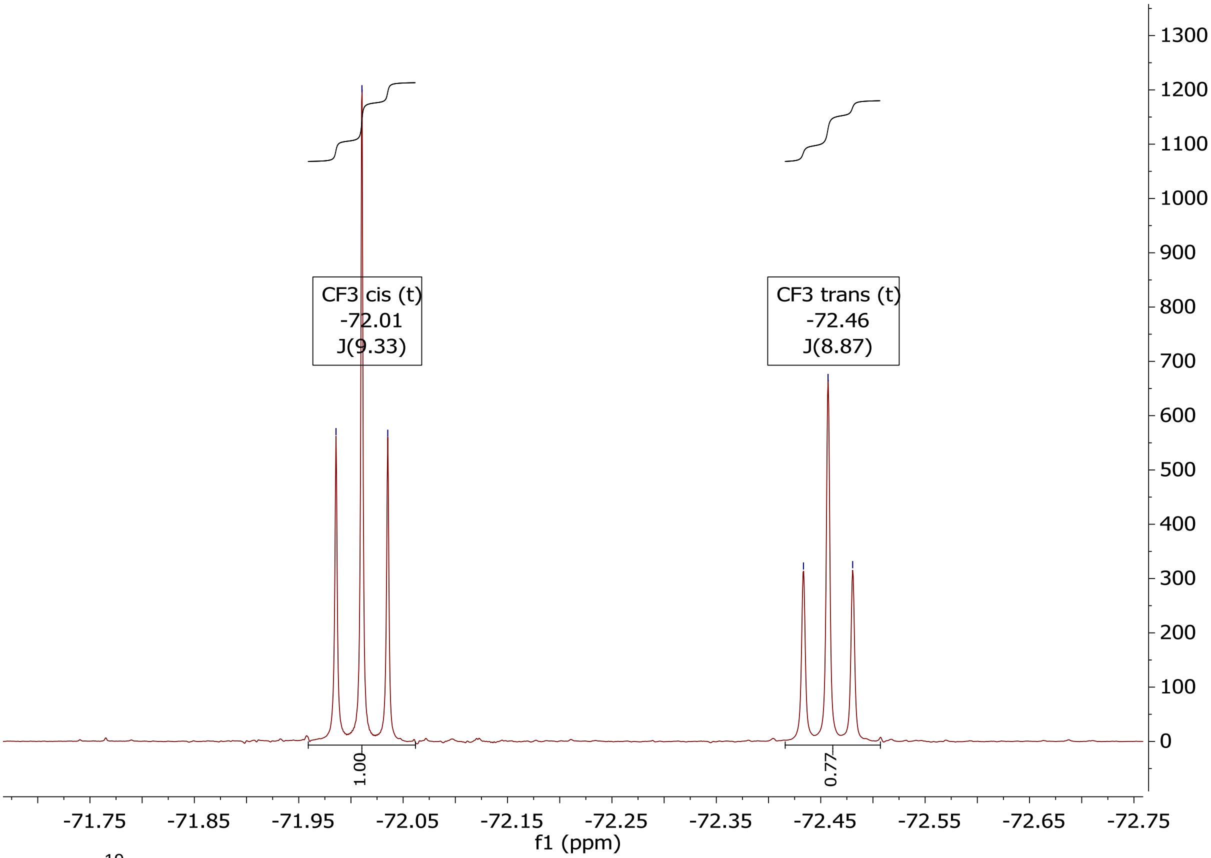

Figure S-84. ^{19}F-NMR spectrum of compound **13** recorded in *methanol-d$_4$* at room temperature.

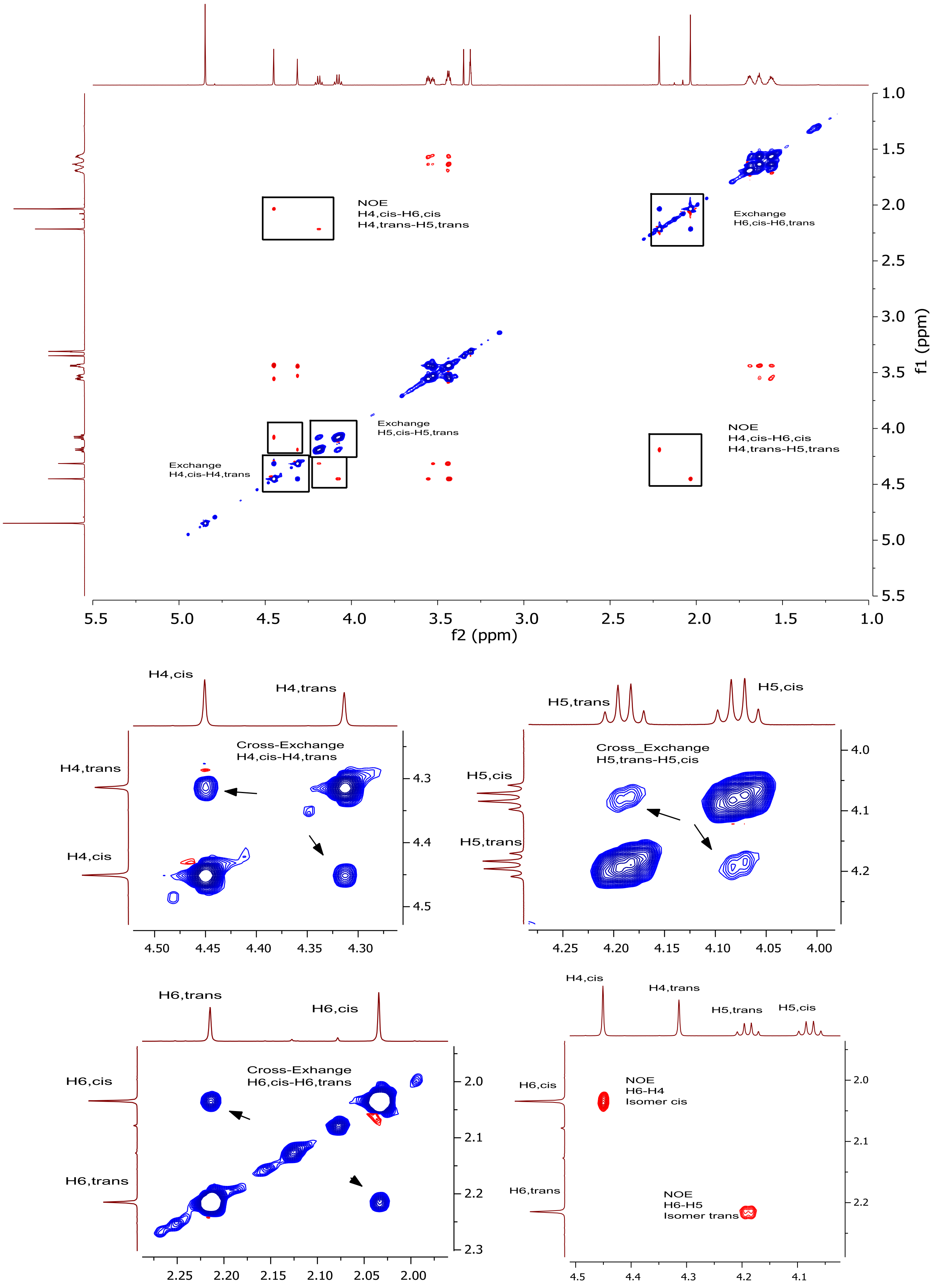

Figure S-85. ^{1}H-^{1}H NOESY spectrum of compound **13** recorded in *methanol-d$_4$* at room temperature. Inset showing the specific spatial contacts that are characteristic of each isomer and allowed the identification of *cis* and *trans* signals.

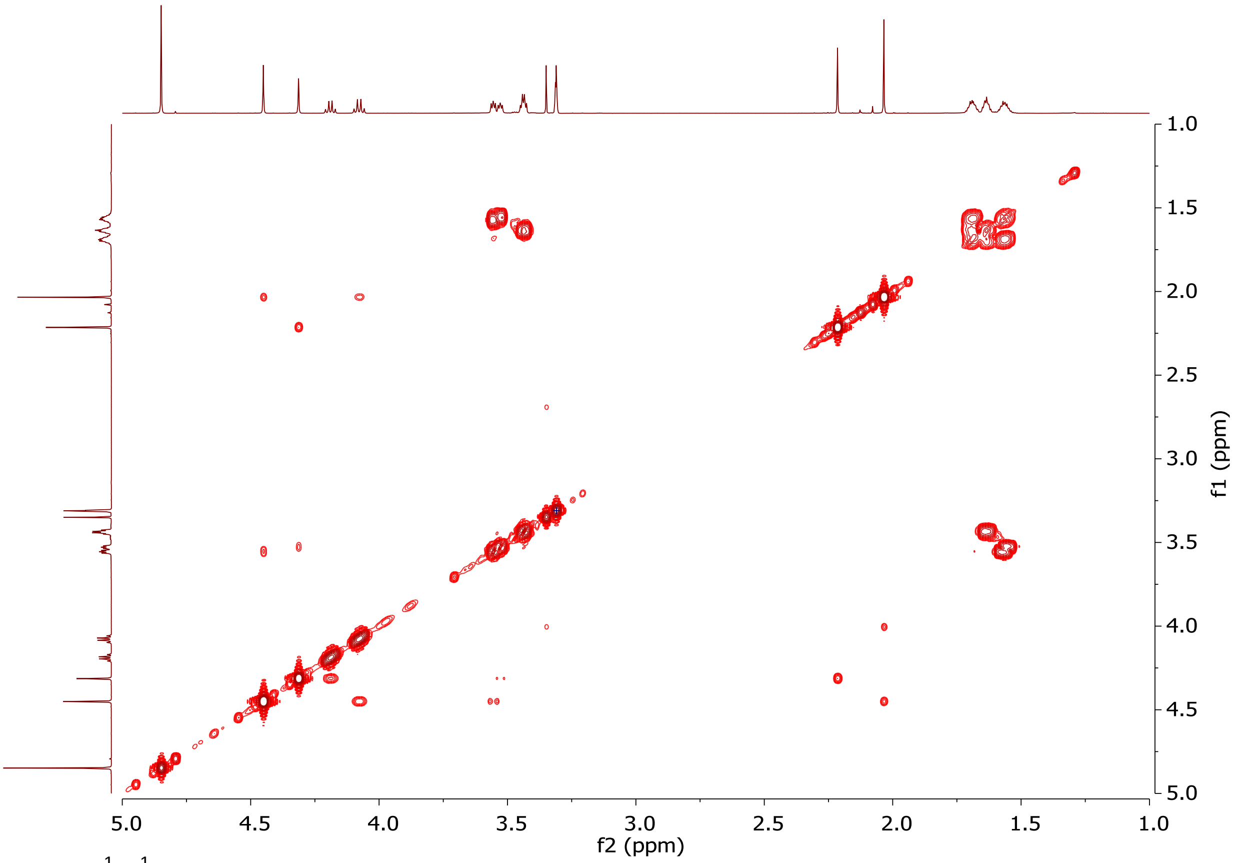

Figure S-86. ¹H-¹H COSY spectrum of compound **13** recorded in *methanol-d*$_4$ at room temperature.

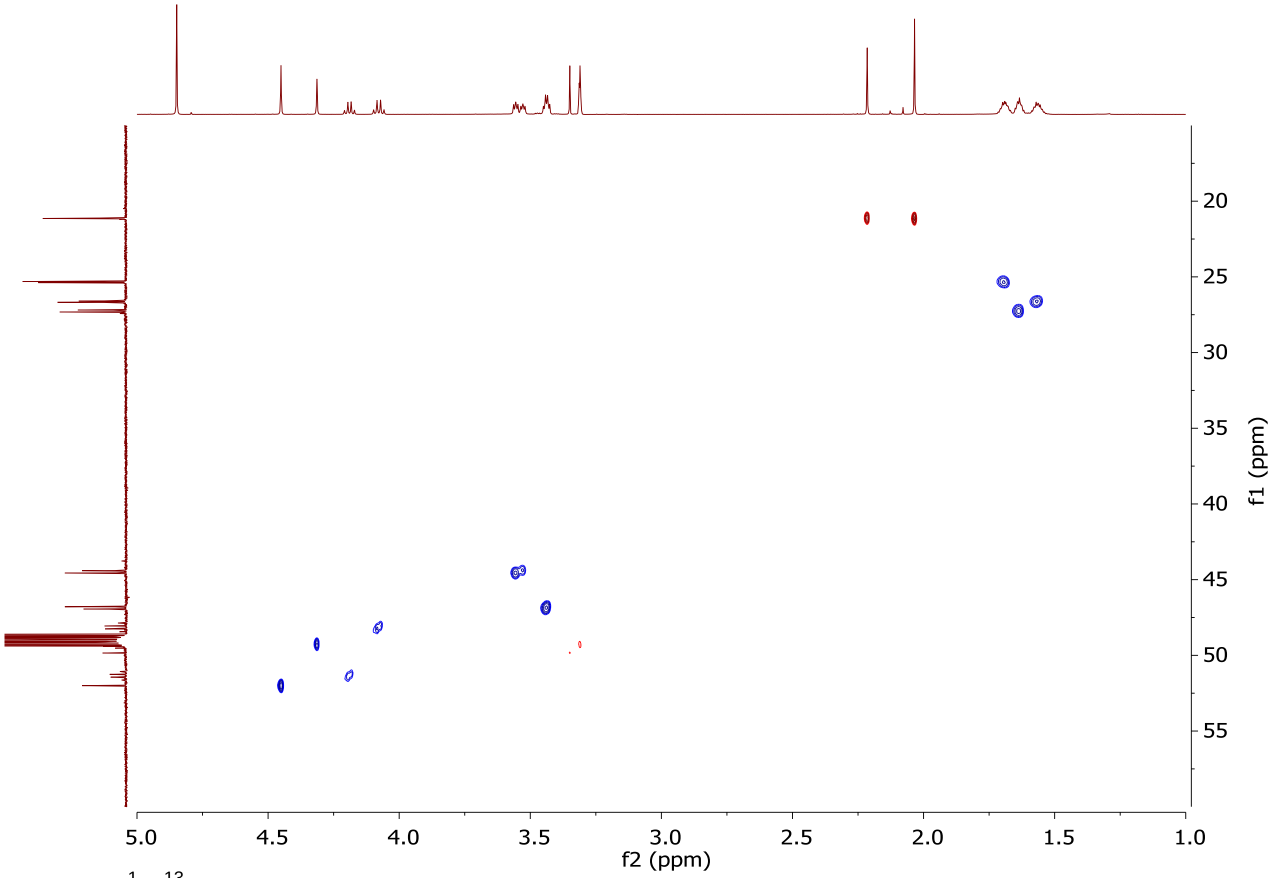

Figure S-87. ¹H-¹³C HSQC spectrum of compound **13** recorded in *methanol-d*$_4$ at room temperature.

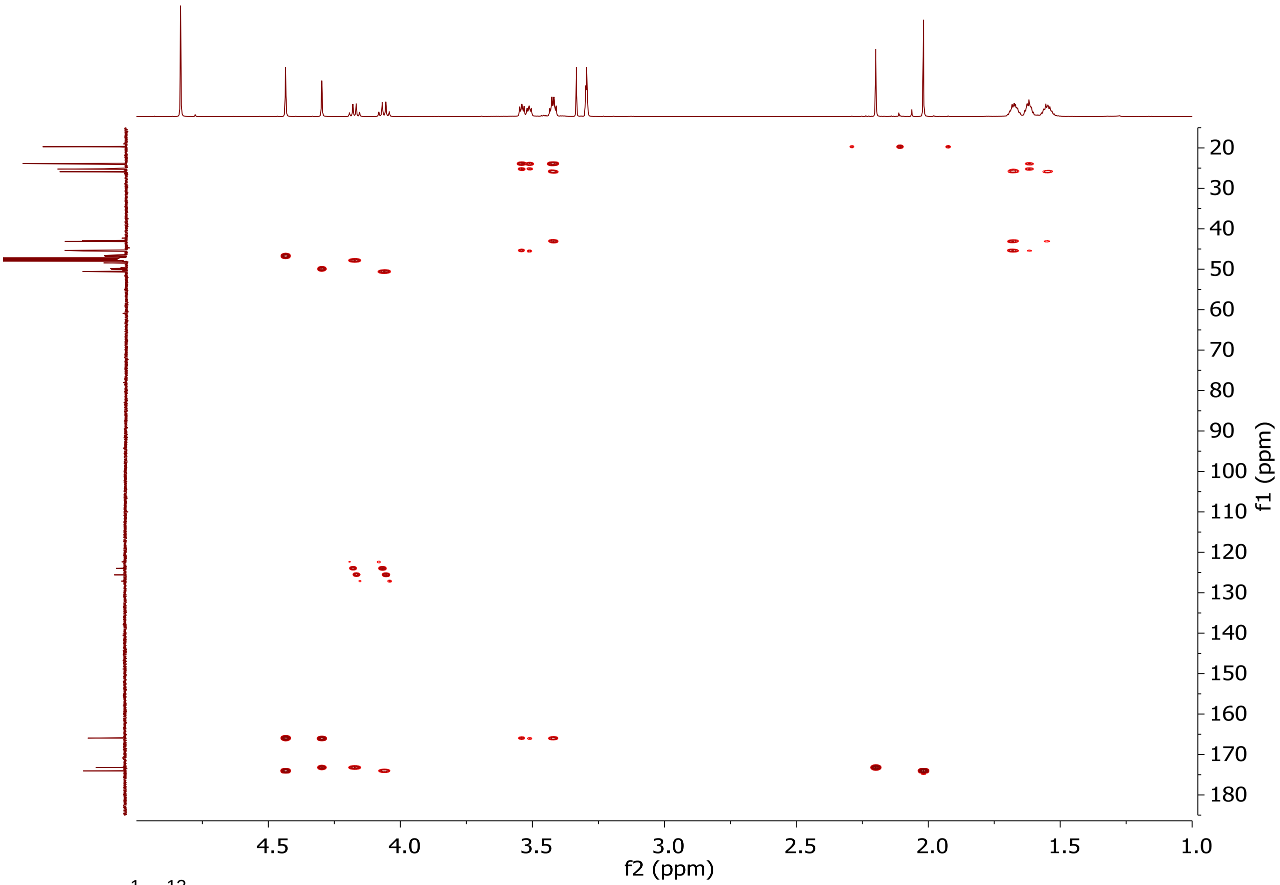

Figure S-88. ^{1}H-^{13}C HMBC spectrum of compound **13** recorded in *methanol-d$_4$* at room temperature.

^{1}H NMR (600 MHz, Chloroform-*d*) Rotamer *trans*: δ ^{1}H NMR: 4.28 (s, 2H, $H_{4,trans}$), 4.02 (q, *J* = 9.2 Hz, 2H, $H_{5,trans}$), 3.54-3.35 (m, 4H, $H_{3,ax+eq,trans}$), 2.21(s, 3H, $H_{6,trans}$), 1.72-1.49 (m, 6H, $H_{1-2,ax+eq,trans}$); Rotamer *cis*: δ ^{1}H NMR: 4.19 (s, 2H, $H_{4,cis}$), 4.05 (q, *J* = 9.2 Hz, 2H, $H_{5,cis}$), 3.60-3.32 (m, 4H, $H_{3,ax+eq,cis}$), 2.04 (s, 3H, $H_{6,cis}$), 1.72-1.49 (m, 6H, $H_{1-2, ax+eq,cis}$).
^{13}C NMR (151 MHz, Chloroform-*d*) Rotamer *trans*: δ ^{13}C NMR: 50.54 (q, *J* = 33.1 Hz, CH_2, $C_{5,trans}$), 46.94 (CH_2, $C_{4,trans}$), 45.99, 43.31 (CH_2, $C_{3,trans}$), 26.35, 25.53, 24.49 (CH_2, $C_{1-2,trans}$), 21.17 (CH_3, $C_{6,trans}$); Rotamer *cis*: δ ^{13}C NMR: 50.30 (CH_2, $C_{4,cis}$), 46.91 (q, *J* = 33.3 Hz, CH_2, $C_{5,cis}$), 45.85, 43.61 (CH_2, $C_{3,cis}$), 26.58, 25.65, 24.41 (CH_2, $C_{1-2,cis}$), 21.23 (CH_3, $C_{6,cis}$); Quaternary carbons: δ ^{13}C NMR: 124.91 (q, *J* = 280.1 Hz, $C_{7,cis}$), 124.64 (q, *J* = 280.5 Hz, $C_{7,trans}$), 172.19 (**C**O-$CH_{3,cis}$), 171.87 (**C**O-$CH_{3,trans}$), 165.67 (**C**O-$pip_{,trans}$), 164.91 (**C**O-$pip_{,cis}$).
^{19}F NMR (376 MHz, Chloroform-*d*) Rotamer *trans*: δ ^{19}F NMR: -70.72 (t, *J* = 8.7 Hz); Rotamer *cis*: δ: -70.16 (t, *J* = 9.3 Hz).

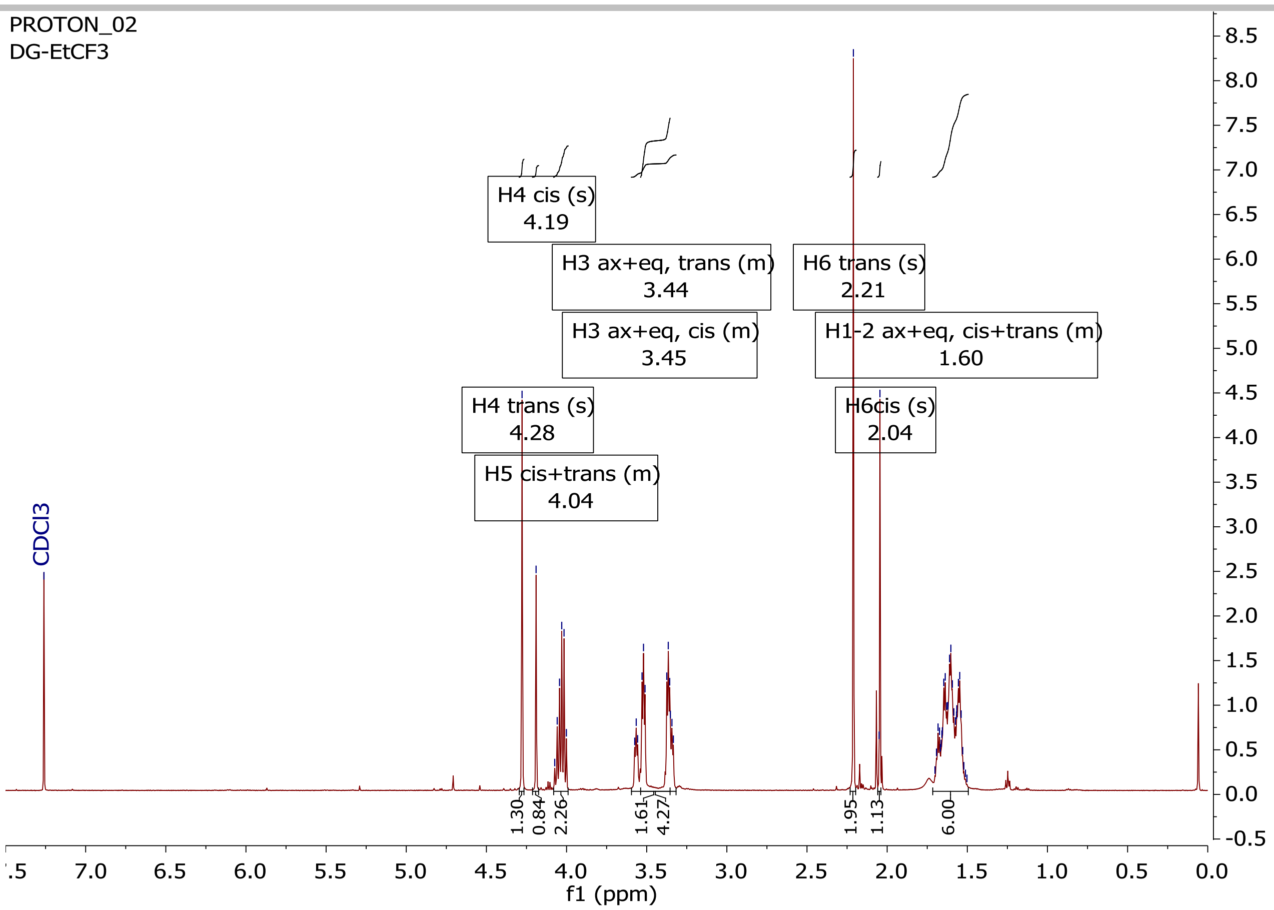

Figure S-89. 1H-NMR spectrum of compound **13** recorded in chloroform-*d* at room temperature.

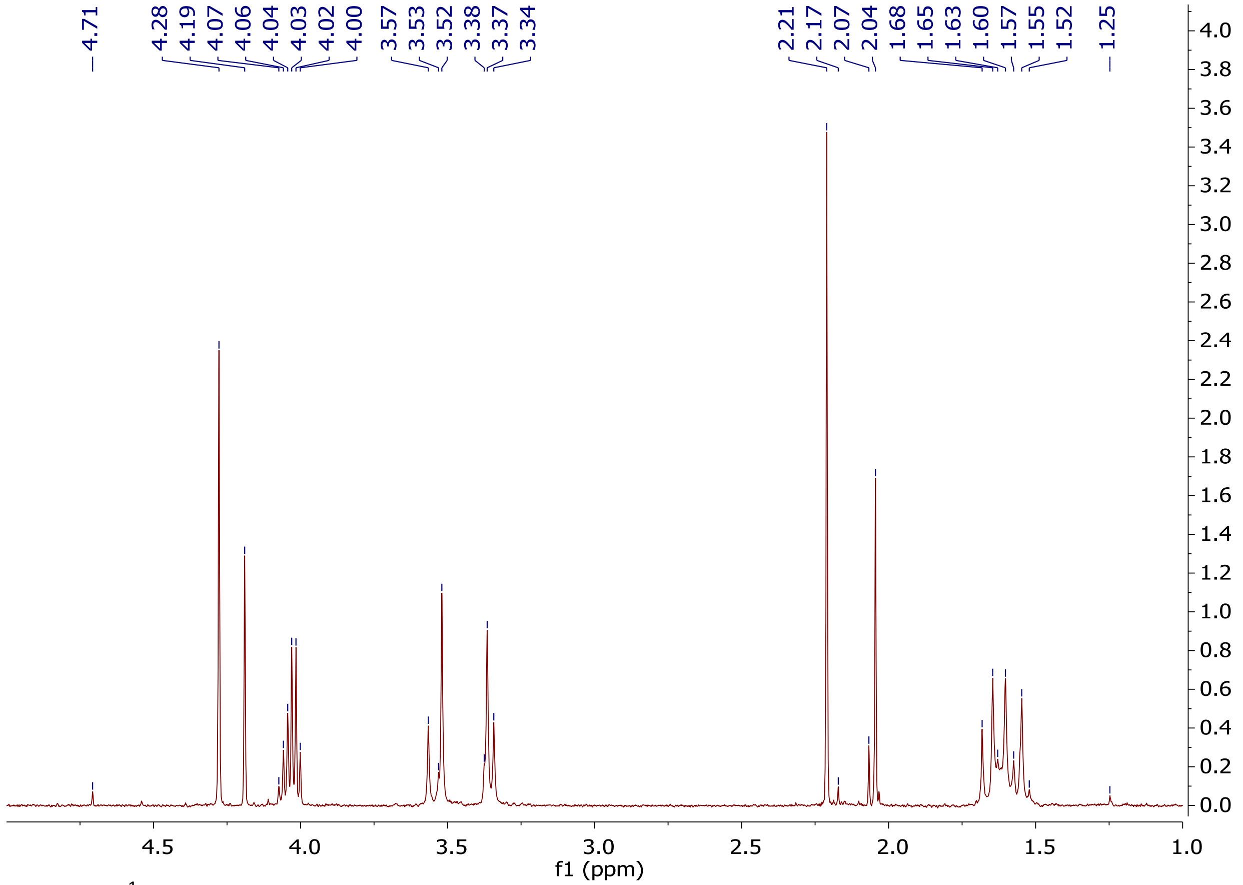

Figure S-90. ^{1}H-*psyche* spectrum of compound **13** recorded in chloroform-*d* at room temperature.

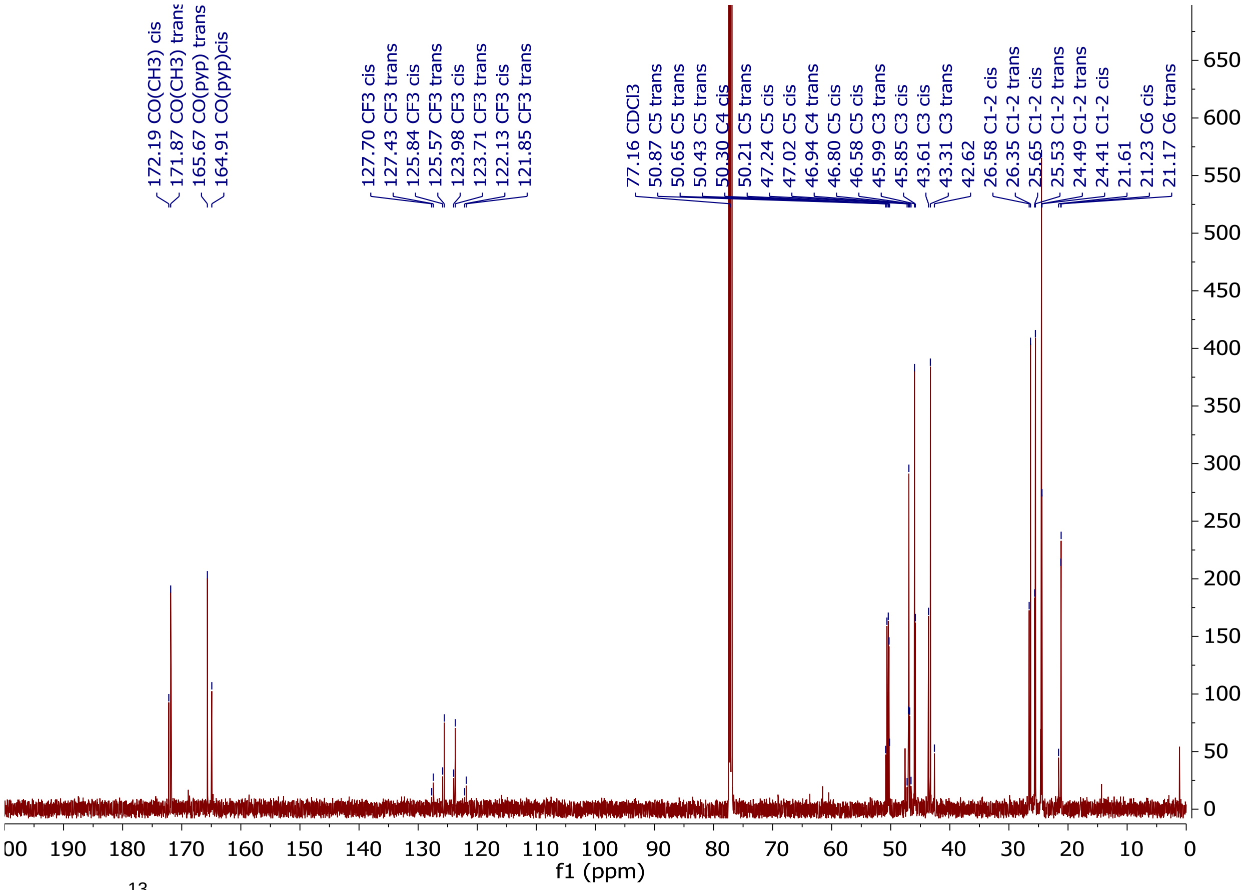

Figure S-91. ^{13}C NMR spectrum of compound **13** recorded in chloroform-*d* at room temperature.

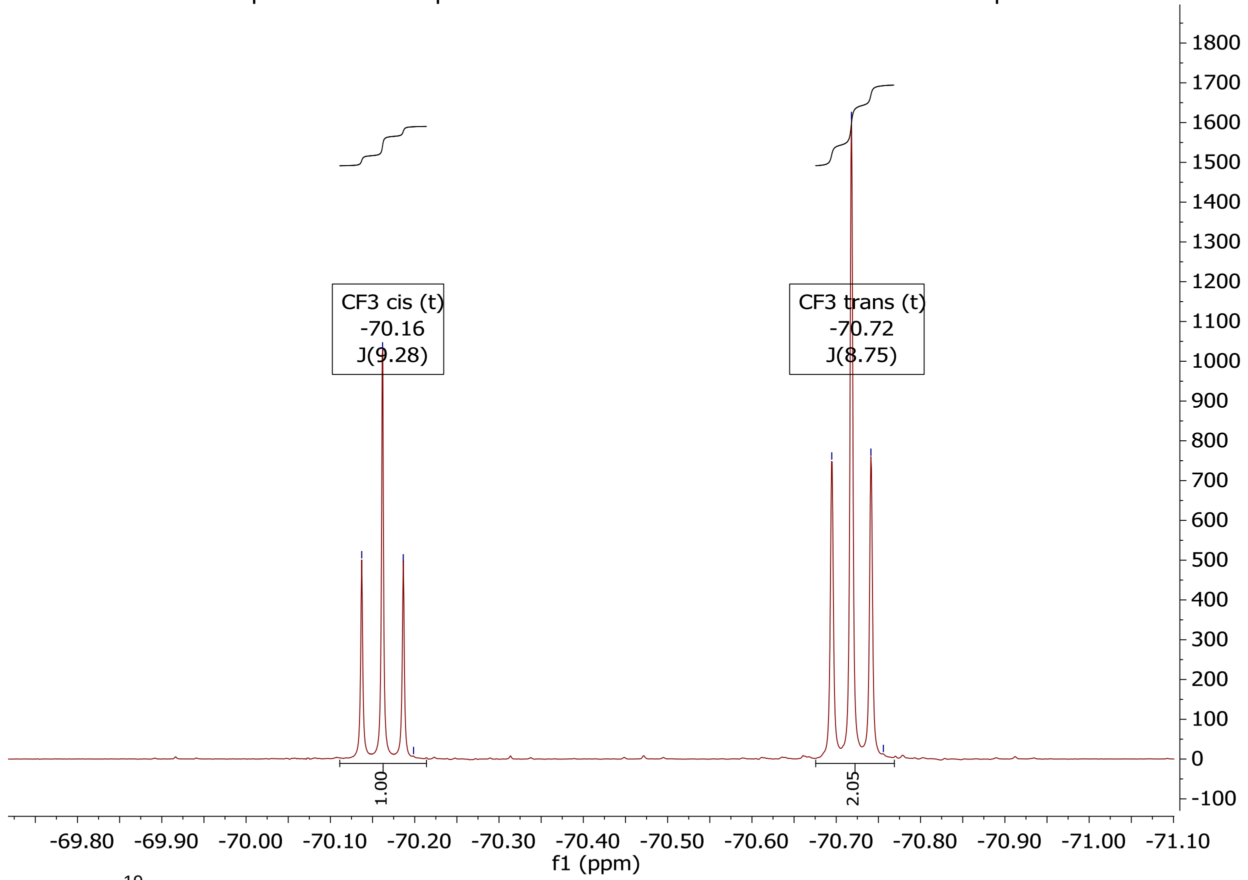

Figure S-92. ^{19}F-NMR spectrum of compound **13** recorded in chloroform-*d* at room temperature.

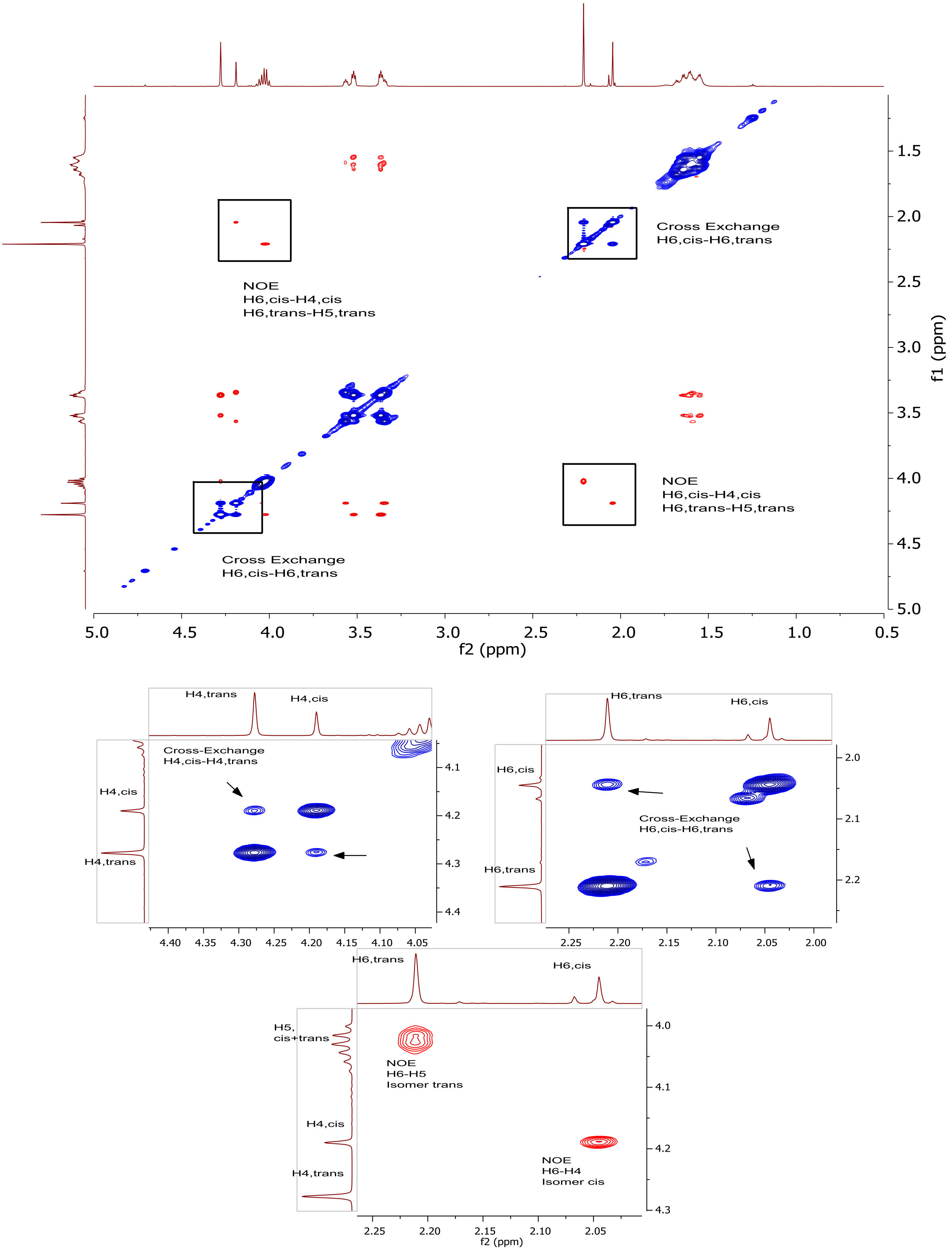

Figure S-93. ^1H-^1H NOESY spectrum of compound **13** recorded in chloroform-*d* at room temperature. Insets showing the specific spatial contacts that are characteristic of each isomer and allowed the identification of *cis* and *trans* signals.

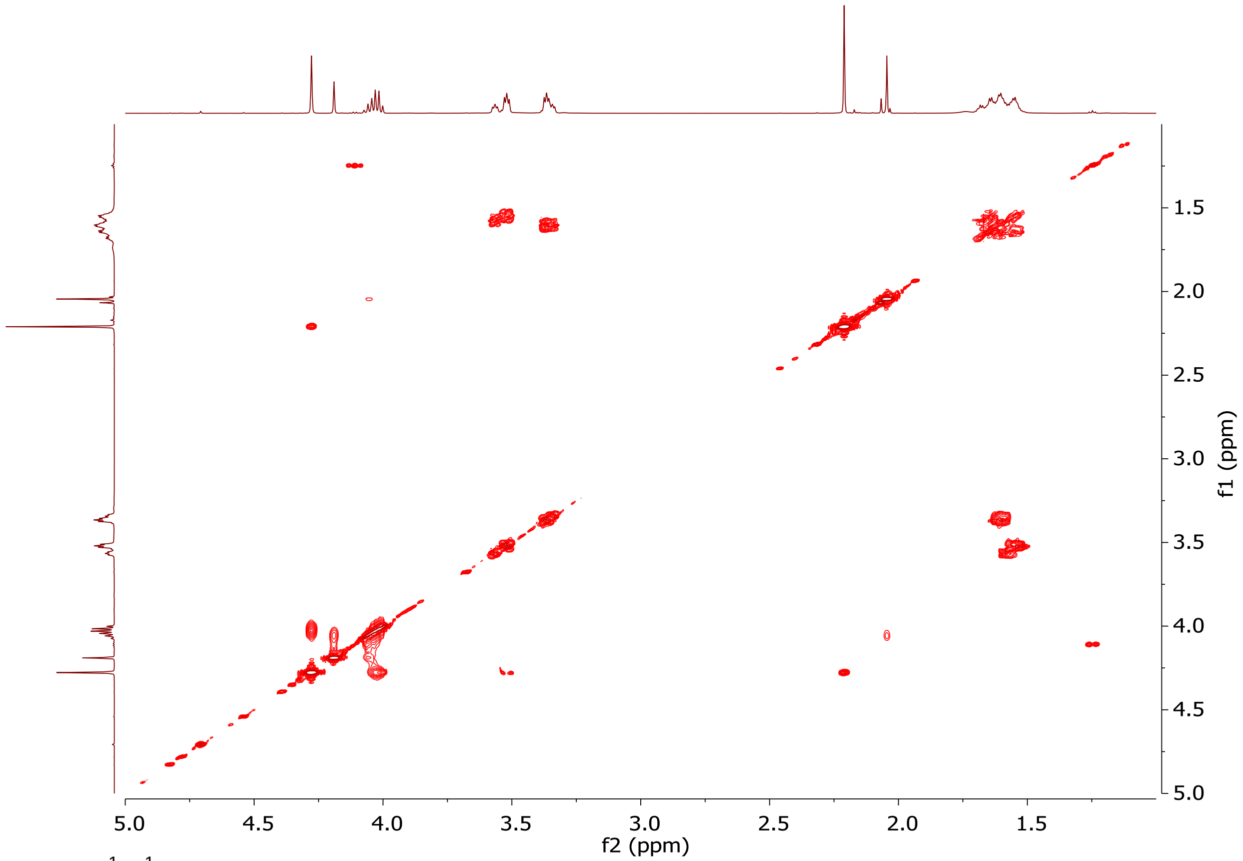

Figure S-94. ^{1}H-^{1}H COSY spectrum of compound **13** recorded in chloroform-*d* at room temperature.

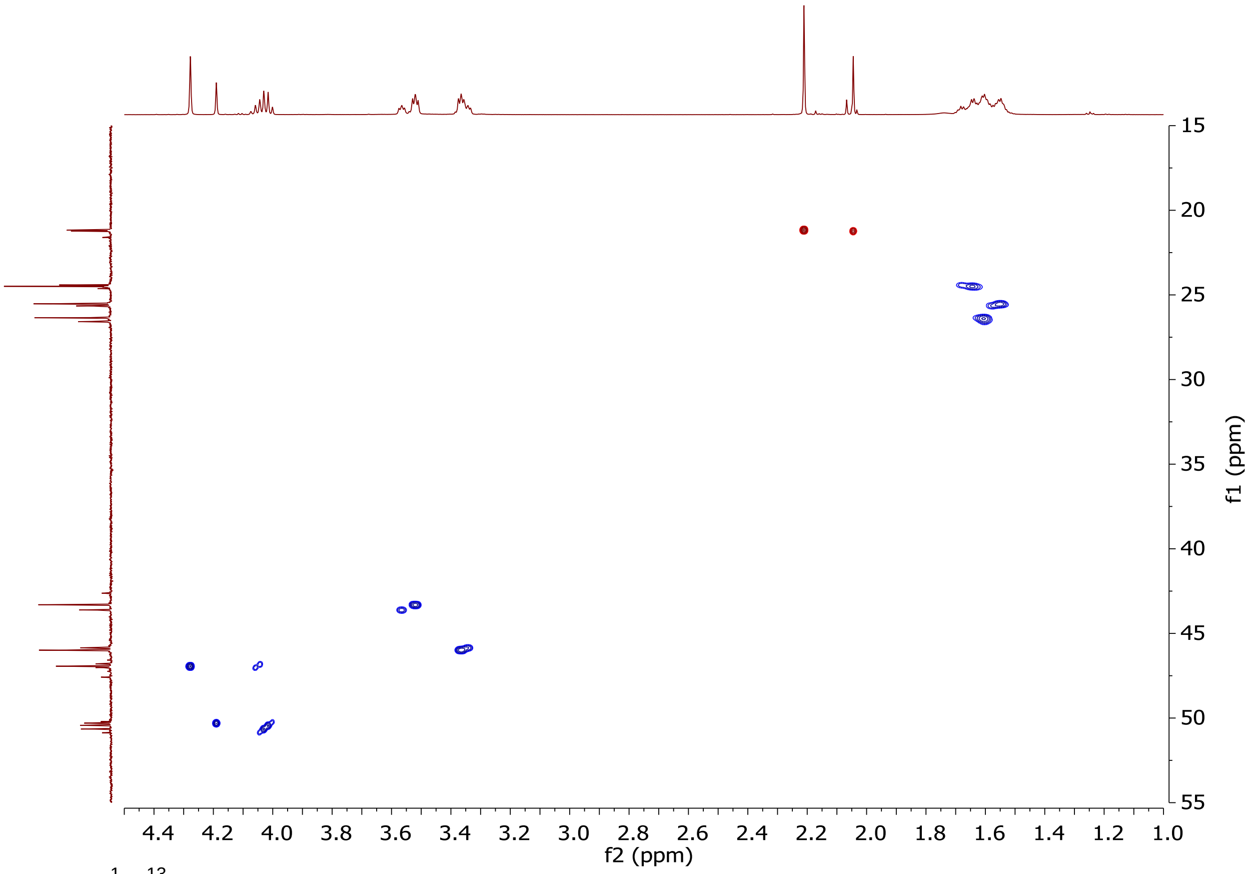

Figure S-95. ^{1}H-^{13}C HSQC spectrum of compound **13** recorded in chloroform-*d* at room temperature

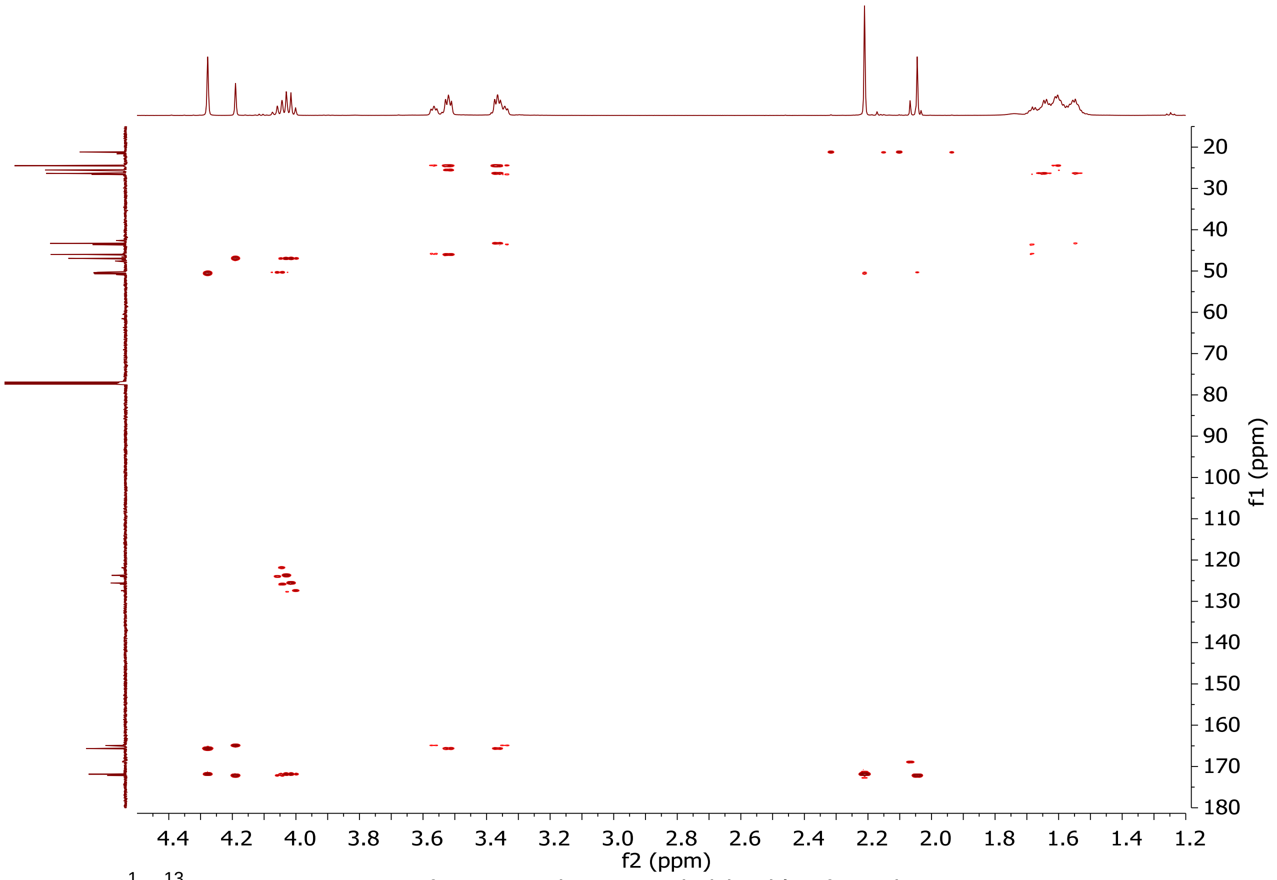

Figure S-96. ^{1}H-^{13}C HMBC spectrum of compound **13** recorded in chloroform-*d* at room temperature.

14: Ac-*Rpe*-pip.

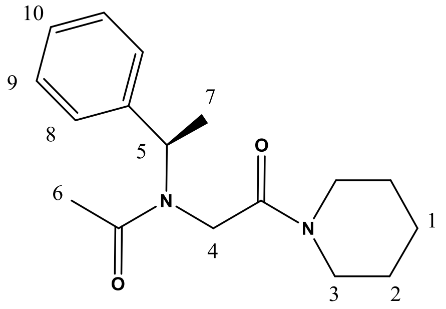

HRMS: calculated for $C_{17}H_{25}N_2O_2$ 289.1916 [M+H]+, found: 289.1928 Da.

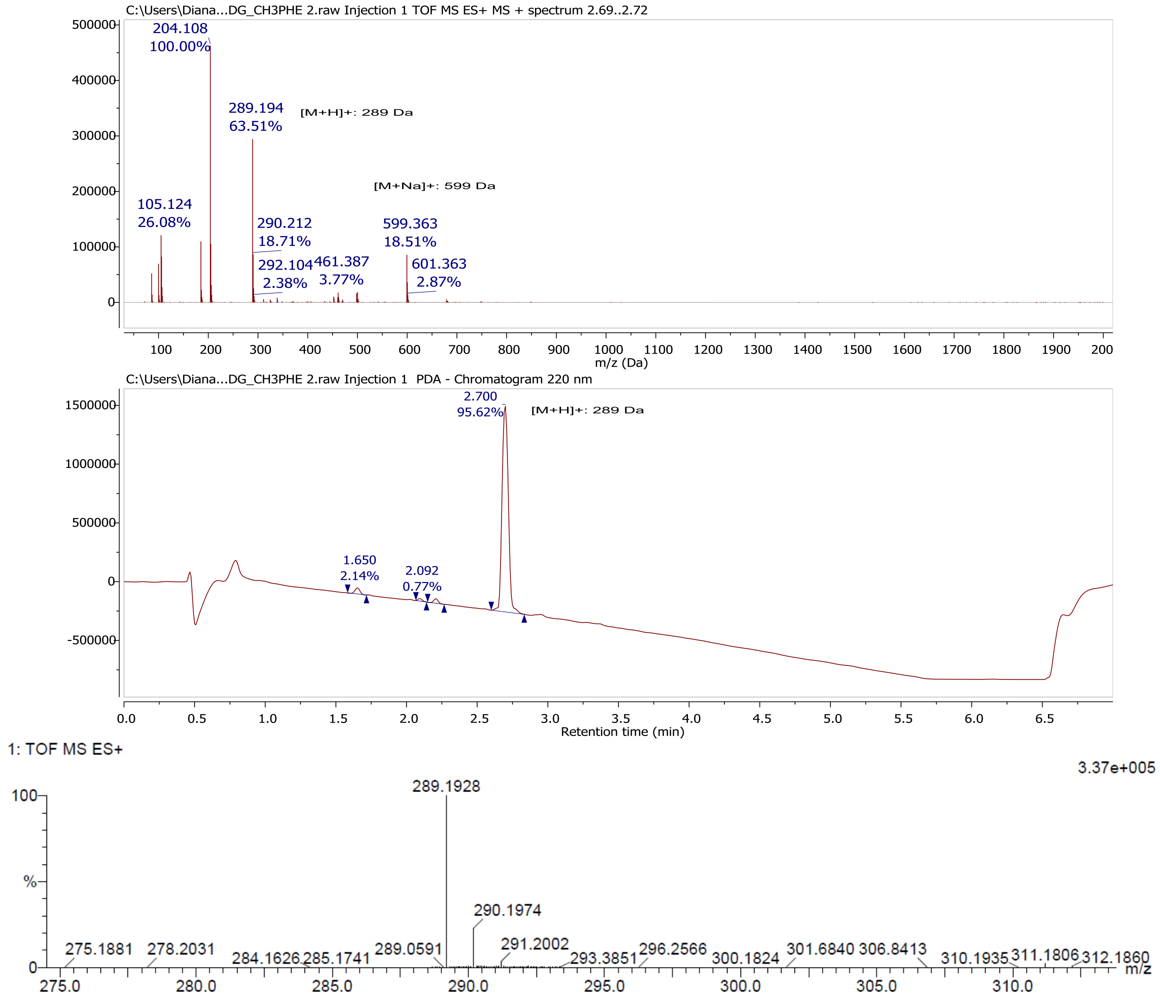

Figure S-97. Structure, HR (QToF)-LC/MS trace and elemental composition of model piperinidyl acetamide **14**.

^{1}H NMR (700MHz, Acetonitrile-*d_3*): Rotamer *trans*: δ ^{1}H NMR: 7.38-7.22 (m, 5H, $H_{8\text{-}10,trans}$), 5.19 (q, *J* = 7.0 Hz, 1H, $H_{5,trans}$), 4.29, 3.54 (AB peak, *J* = 16.3 Hz, 2H, $H_{4,trans}$), 3.50-3.15 (m, 4H, $H_{3,ax+eq,trans}$), 2.12 (s, 3H, $H_{6,trans}$), 1.63-1.42 (m, 6H, $H_{1\text{-}2,ax+eq,trans}$), 1.54 (d, *J* = 7.0 Hz, 3H, $H_{7,trans}$). Rotamer *cis*: δ ^{1}H NMR: 7.38-7.22 (m, 5H, $H_{8\text{-}10,trans}$), 5.87 (q, *J* = 7.2 Hz, 1H, $H_{5,cis}$), 4.03, 3.78 (AB peak, *J* = 18.0 Hz, 2H, $H_{4,cis}$), 3.50-3.15 (m, 4H, $H_{3,ax+eq,cis}$), 1.93 (s, 3H, $H_{6,cis}$), 1.63-1.42 (m, 6H, $H_{1\text{-}2,ax+eq,cis}$), 1.37 (d, *J* = 7.2 Hz, 3H, $H_{7,cis}$).
^{13}C NMR (176 MHz, Acetonitrile-*d_3*): Rotamer *trans*: δ ^{13}C NMR: 129.47, 128.20, 127,70 (CH, $C_{8\text{-}10,trans}$), 57.14 (CH, $C_{5,trans}$), 46.42, 43.65 (CH_2, $C_{3,trans}$), 44.48 (CH_2, $C_{4,trans}$), 26.97, 26.39, 25.19 (CH_2, $C_{1\text{-}2,trans}$), 22.11 (CH_3, $C_{6,trans}$), 18.97 (CH_3, $C_{7,trans}$); Rotamer *cis*: δ ^{13}C NMR: 129.20,128.32, 127.98 (CH, $C_{8\text{-}10,cis}$), 52,00 (CH, $C_{5,cis}$), 46.33, 43.90 (CH_2, $C_{3,cis}$), 46.29 (CH_2, $C_{4,cis}$), 26.97, 26.39, 25.08 (CH_2, $C_{1\text{-}2,cis}$), 22.44 (CH_3, $C_{5,cis}$), 16.97 (CH_3, $C_{7,cis}$); Quaternary carbons: δ ^{13}C NMR: 172.36 (**C**O-$CH_{3,cis}$), 171.12 (**C**O-$CH_{3,trans}$), 167.33 (**C**O-pip$_{,cis}$), 167.15 (**C**O-pip$_{,trans}$), 142.92 (**C**$_{Ar,trans}$), 142.69 (**C**$_{Ar,cis}$).

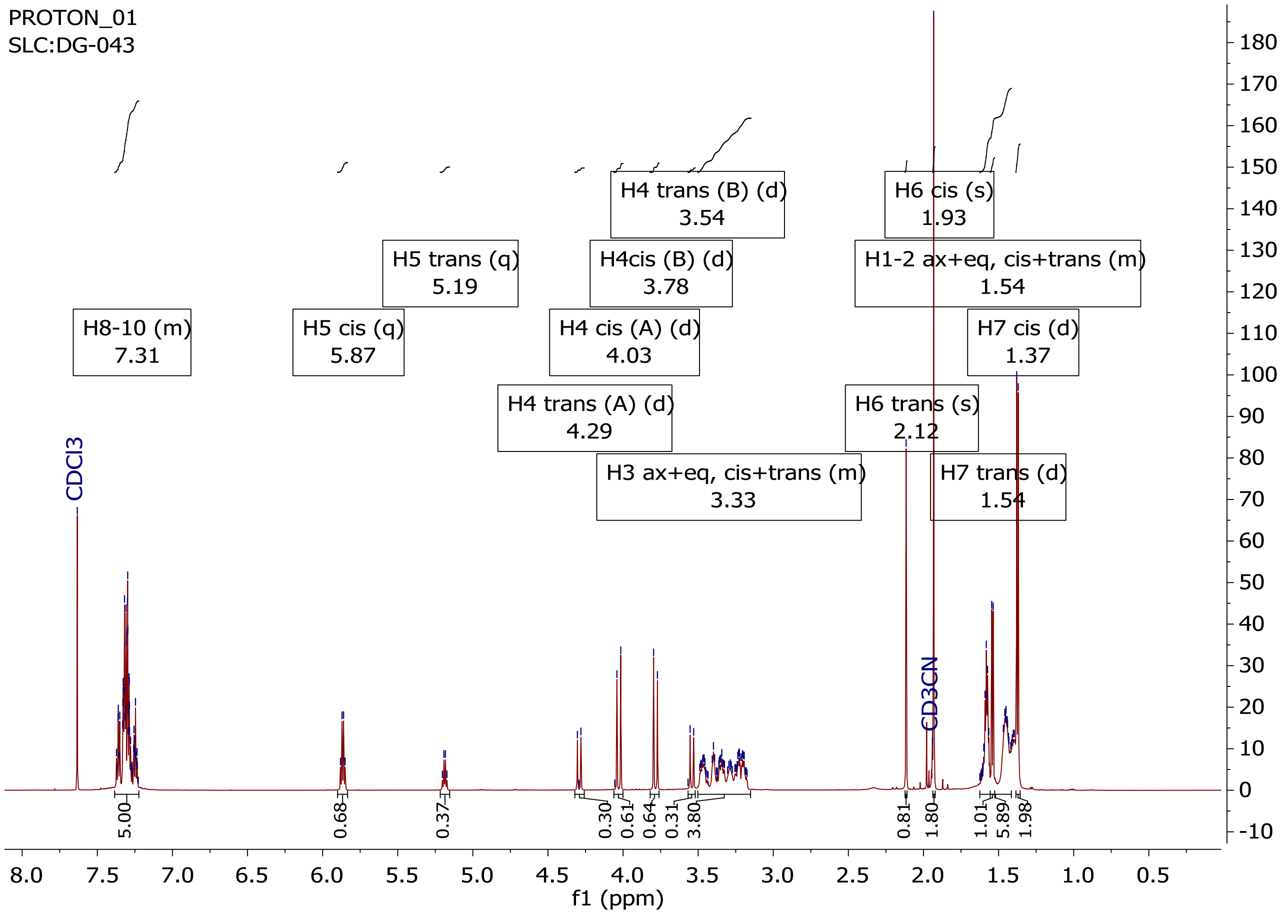

Figure S-98. 1H-NMR spectrum of compound **14** recorded in *acetonitrile-d_3* at room temperature.

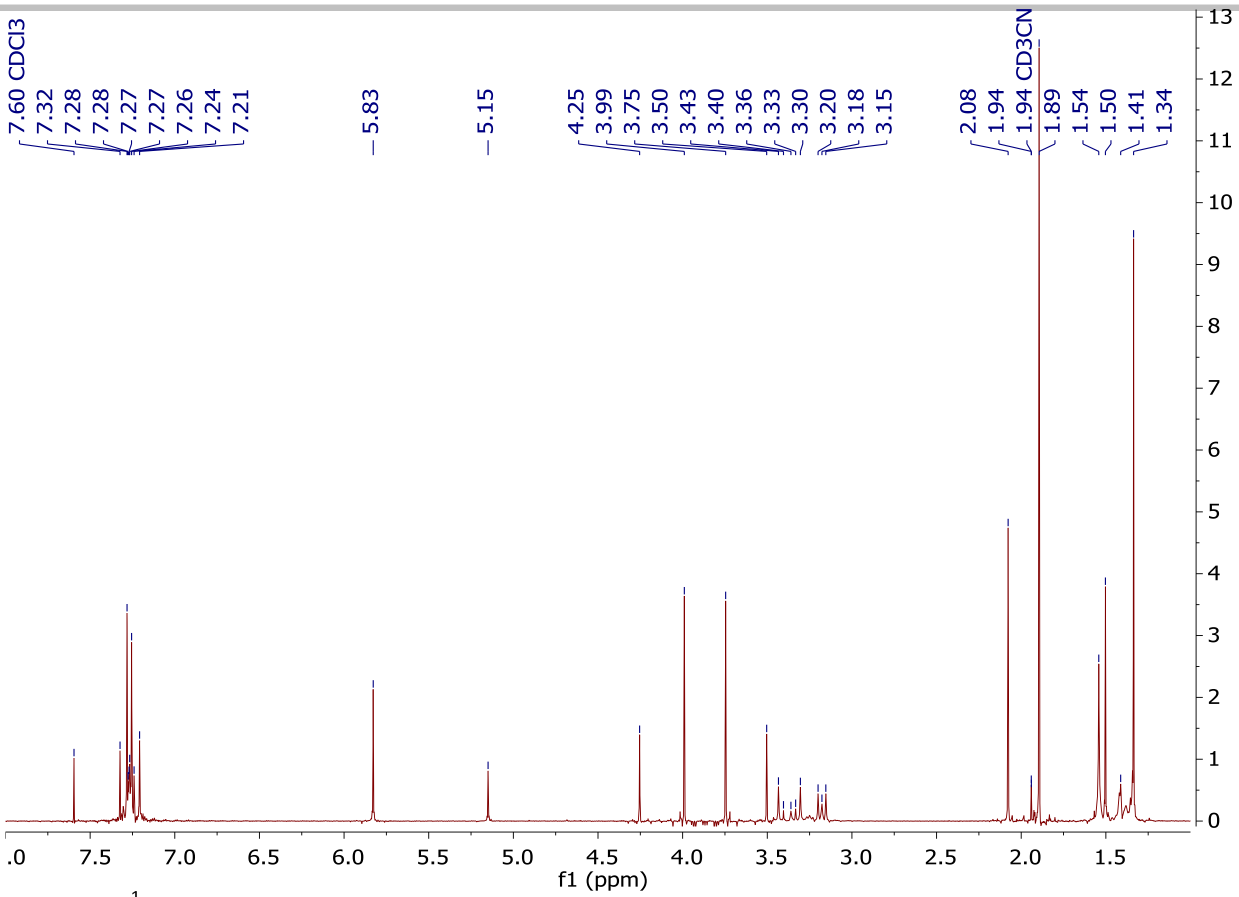

Figure S-99. ^{1}H-*psyche* spectrum of compound **14** recorded in *acetonitrile-d$_3$* at room temperature.

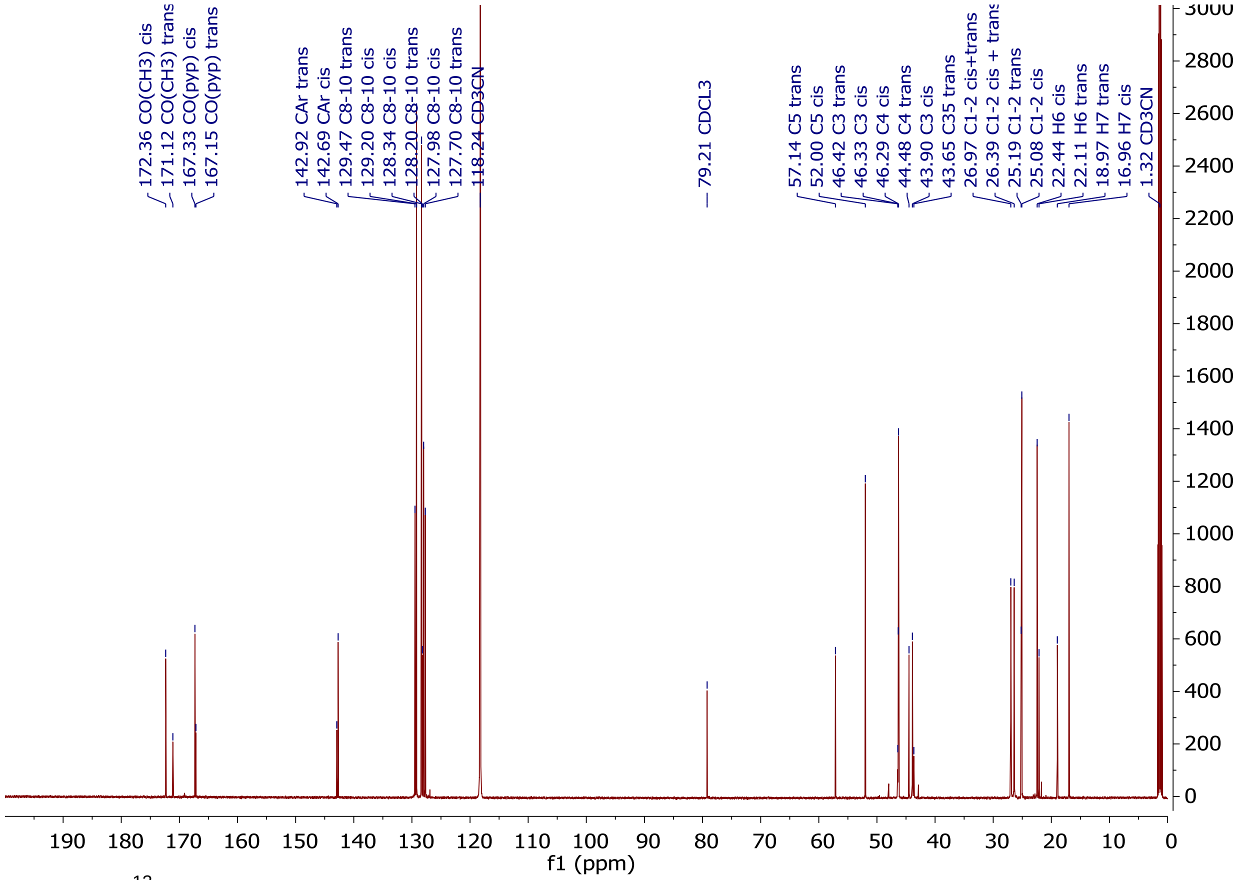

Figure S-100. ^{13}C NMR spectrum of compound **14** recorded in *acetonitrile-d$_3$* at room temperature.

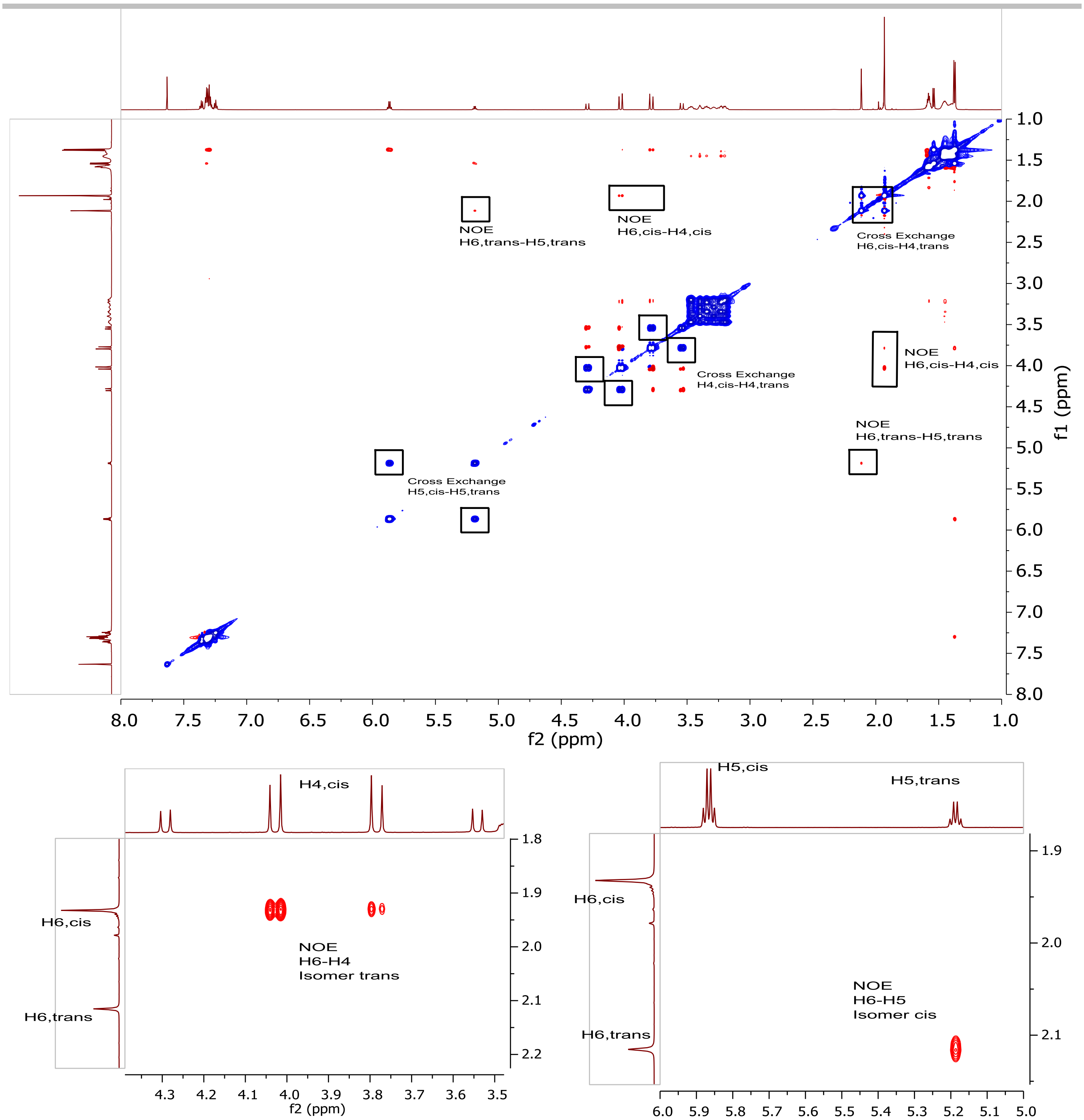

Figure S-101. ^{1}H-^{1}H NOESY spectrum of compound **14** recorded in *acetonitrile-d*$_3$ at room temperature. Inset showing the specific spatial contacts that are characteristic of each isomer and allowed the identification of *cis* and *trans* signals.

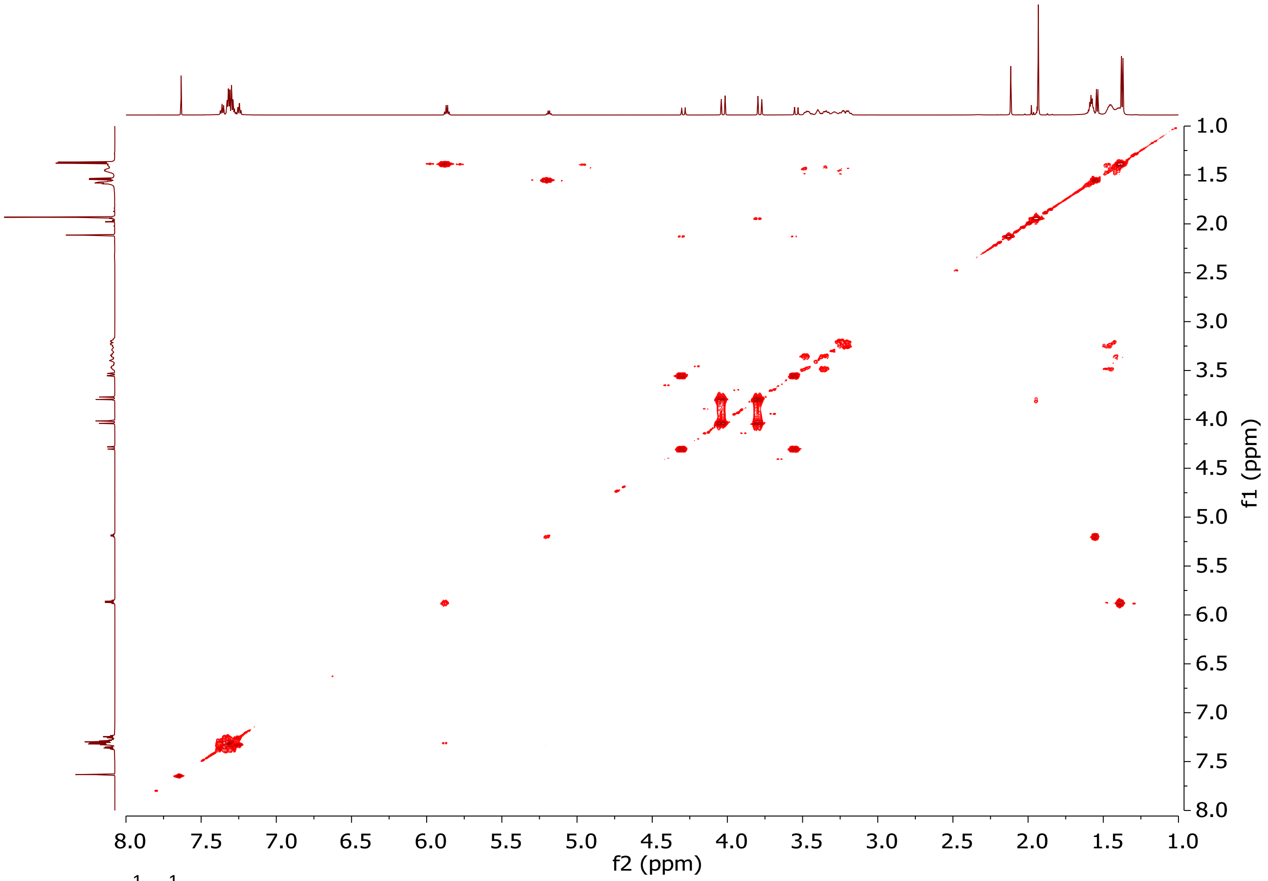

Figure S-102. ^{1}H-^{1}H COSY spectrum of compound **14** recorded in *acetonitrile-d_3* at room temperature.

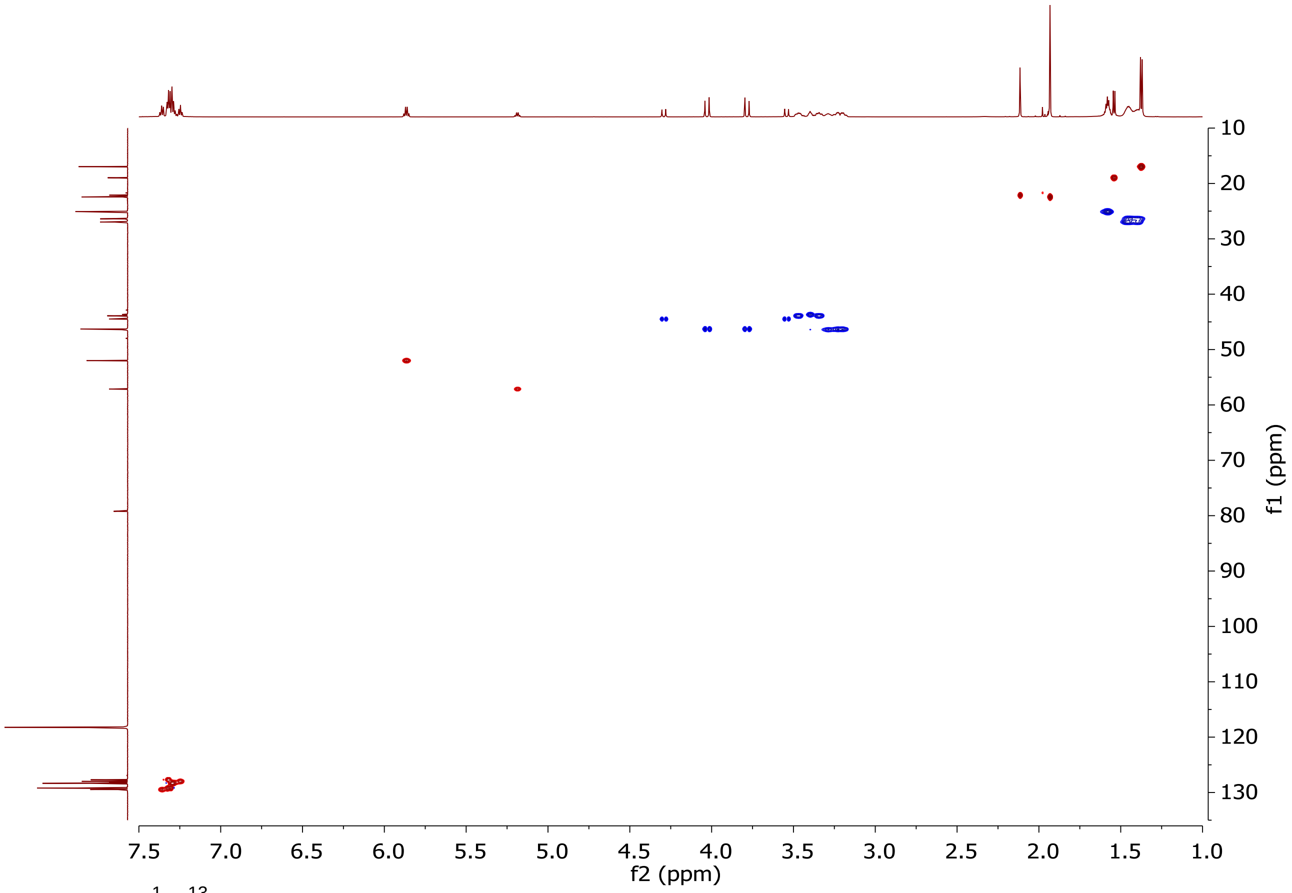

Figure S-103. ^{1}H-^{13}C HSQC spectrum of compound **14** recorded in *acetonitrile-d_3* at room temperature.

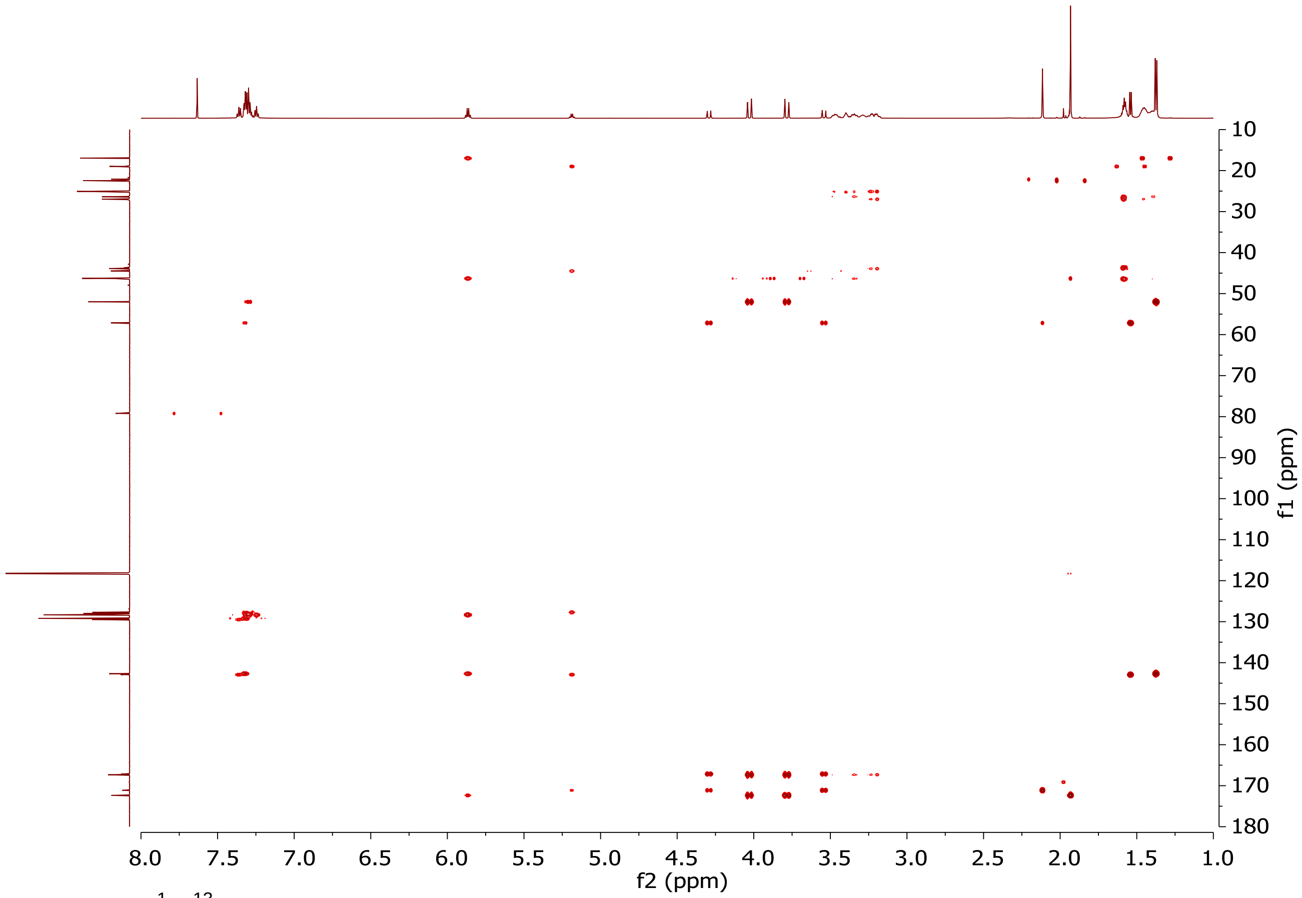

Figure S-104. ^{1}H-^{13}C HMBC spectrum of compound **14** recorded in *acetonitrile-d$_3$* at room temperature.

^{1}H NMR (700MHz, Methanol-*d*$_4$): Rotamer *trans*: δ ^{1}H NMR: 7.40-7.23 (m, 5H, H$_{8\text{-}10,trans}$), 5.27 (q, *J* = 7.0 Hz, 1H, H$_{5,trans}$), 4.37, 3.73 (AB peak, *J* = 16.3 Hz, 2H, H$_{4,trans}$), 3.57-3.19 (m, 4H, H$_{3,ax+eq,trans}$), 2.23 (s, 3H, H$_{6,trans}$), 1.70-1.31 (m, 6H, H$_{1\text{-}2,ax+eq,trans}$), 1.59 (d, *J* = 7.0 Hz, 3H, H$_{7,trans}$). Rotamer *cis*: δ ^{1}H NMR: 7.40-7.23 (m, 5H, H$_{8\text{-}10,trans}$), 5.93 (q, *J* = 7.1 Hz, 1H, H$_{5,cis}$), 4.16, 3.90 (AB peak, *J* = 18.0 Hz, 2H, H$_{4,cis}$), 3.59-3.17 (m, 4H, H$_{3,ax+eq,cis}$), 2.04 (s, 3H, H$_{6,cis}$), 1.70-1.31 (m, 6H, H$_{1\text{-}2,ax+eq,cis}$), 1.44 (d, *J* = 7.2 Hz, 3H, H$_{7,cis}$).
^{13}C NMR (176 MHz, Methanol-*d*$_4$): Rotamer *trans*: δ ^{13}C NMR: 129.78, 128.63, 127.79 (CH, C$_{8\text{-}10,trans}$), 57.99 (CH, C$_{5,trans}$), 46.99, 44.42 (CH$_2$, C$_{3,trans}$), 44.83 (CH$_2$, C$_{4,trans}$), 27.15, 26.56, 25.37 (CH$_2$, C$_{1\text{-}2,trans}$), 21.73 (CH$_3$, C$_{6,trans}$), 18.86 (CH$_3$, C$_{7,trans}$); Rotamer *cis*: δ ^{13}C NMR: 129.47, 128.89, 128.57 (CH, C$_{8\text{-}10,cis}$), 52.98 (CH, C$_{5,cis}$), 46.80, 44.49 (CH$_2$, C$_{3,cis}$), 46.34 (CH$_2$, C$_{4,\ cis}$), 27.19, 26.61, 25.23 (CH$_2$, C$_{1\text{-}2,cis}$), 22.19 (CH$_3$, C$_{6,cis}$), 16.62 (CH$_3$, C$_{7,cis}$); Quaternary carbons: δ ^{13}C NMR: 174.60 (**C**O-CH$_{3,cis}$), 173.64 (**C**O-CH$_{3,trans}$), 168.04 (**C**O-pip$_{,trans}$), 167.91 (**C**O-pip$_{,cis}$), 142.33 (**C**$_{Ar,cis}$), 141.48 (**C**$_{Ar,trans}$).

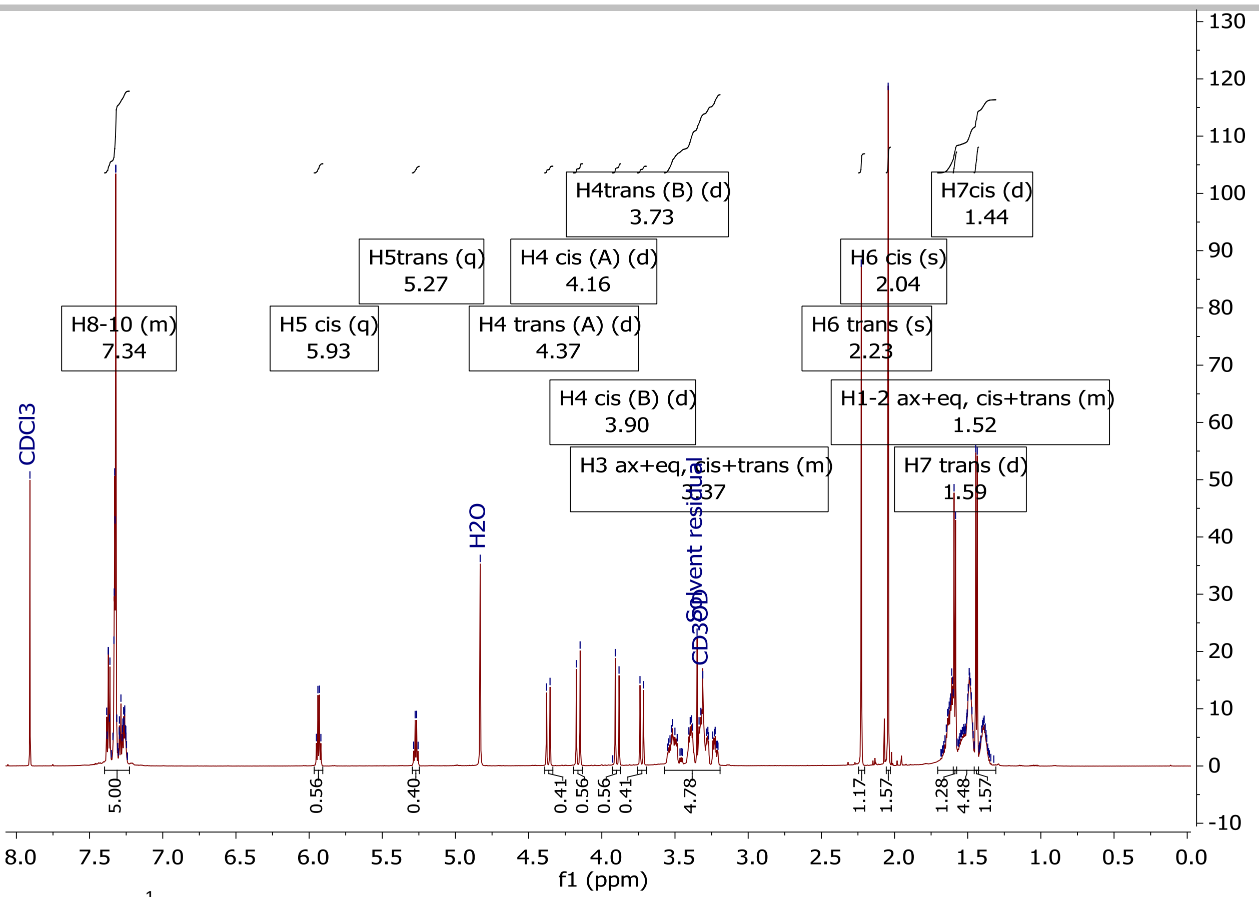

Figure S-105. ^{1}H-NMR spectrum of compound **14** recorded in *methanol-d$_4$* at room temperature.

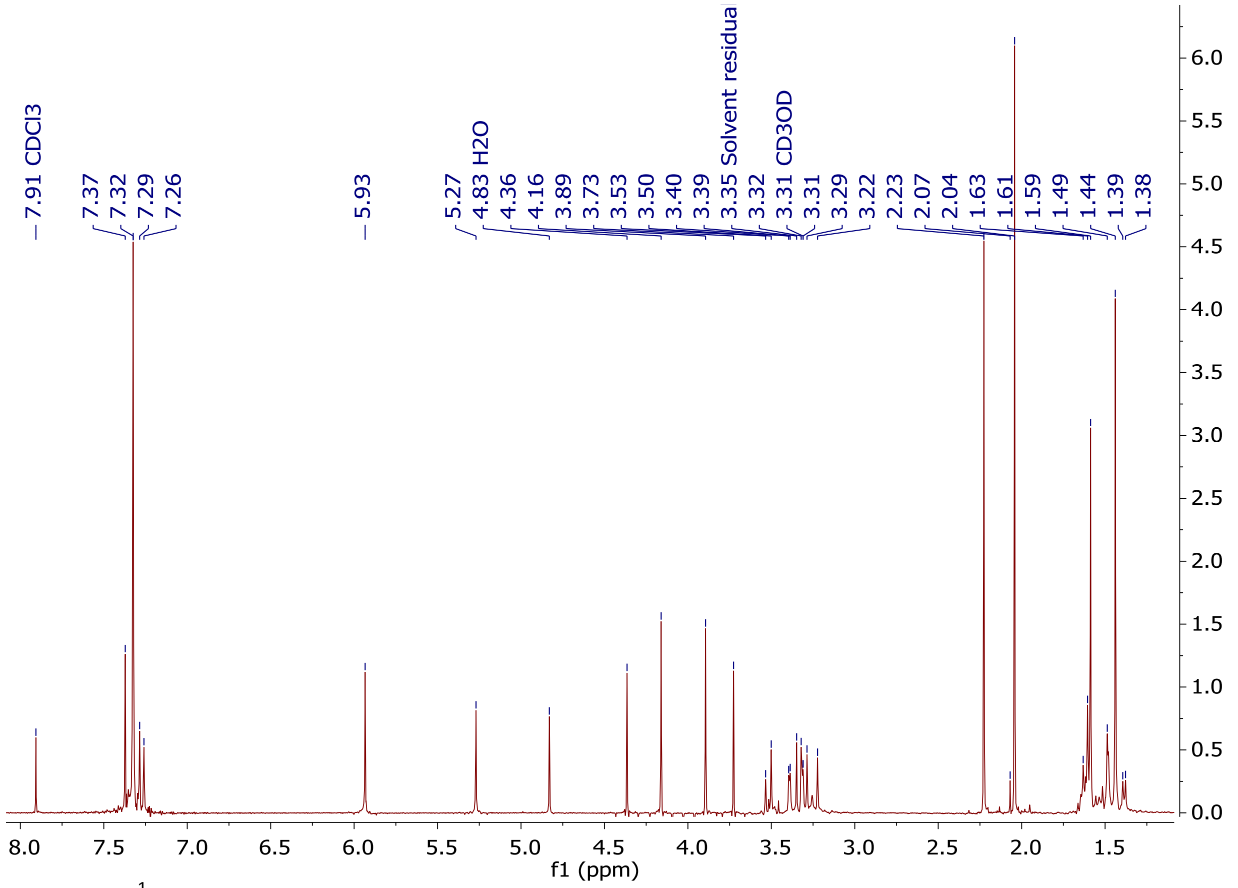

Figure S-106. ^{1}H-*psyche* spectrum of compound **14** recorded in *methanol-d$_4$* at room temperature.

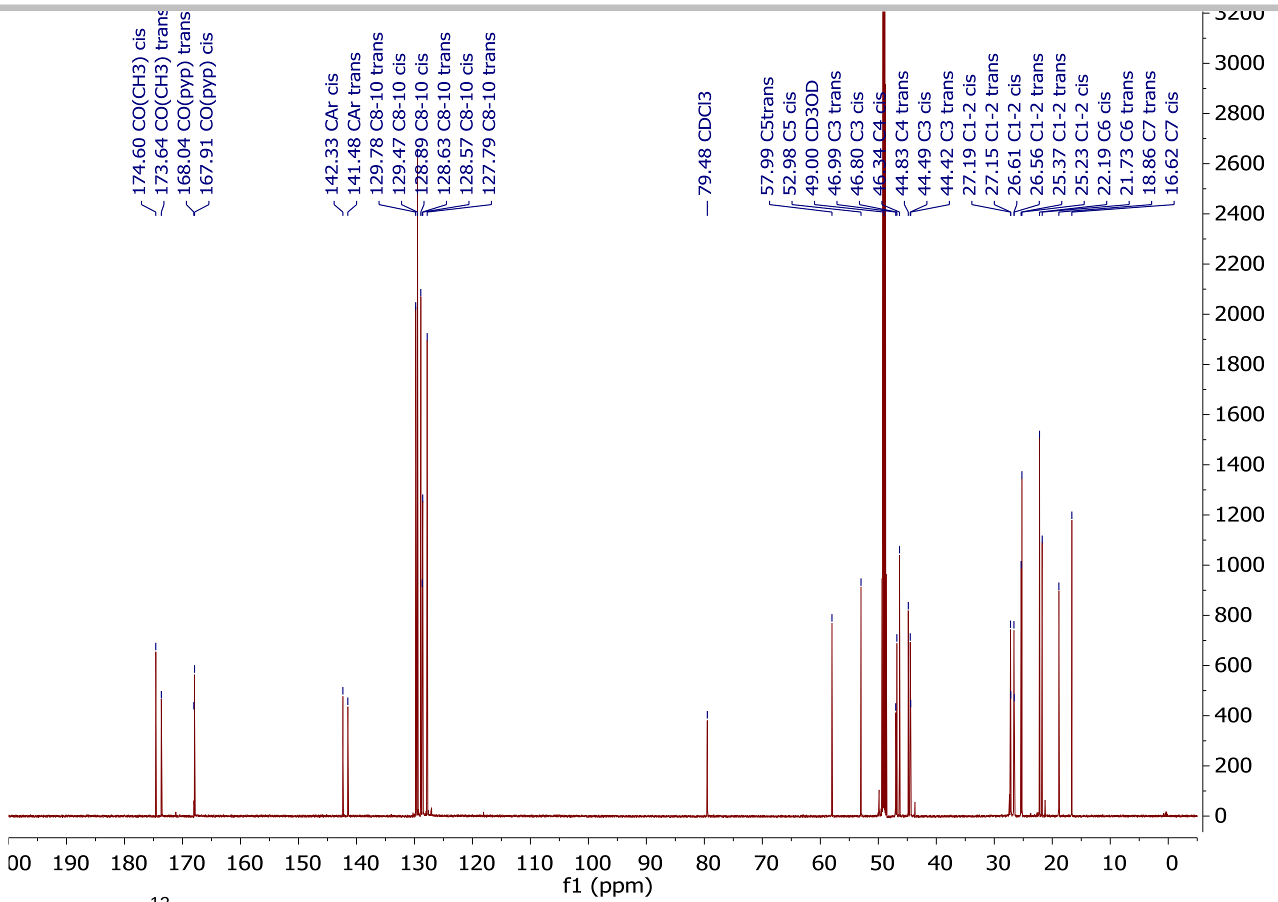

Figure S-107. ^{13}C NMR spectrum of compound **14** recorded in *methanol-d_4* at room temperature.

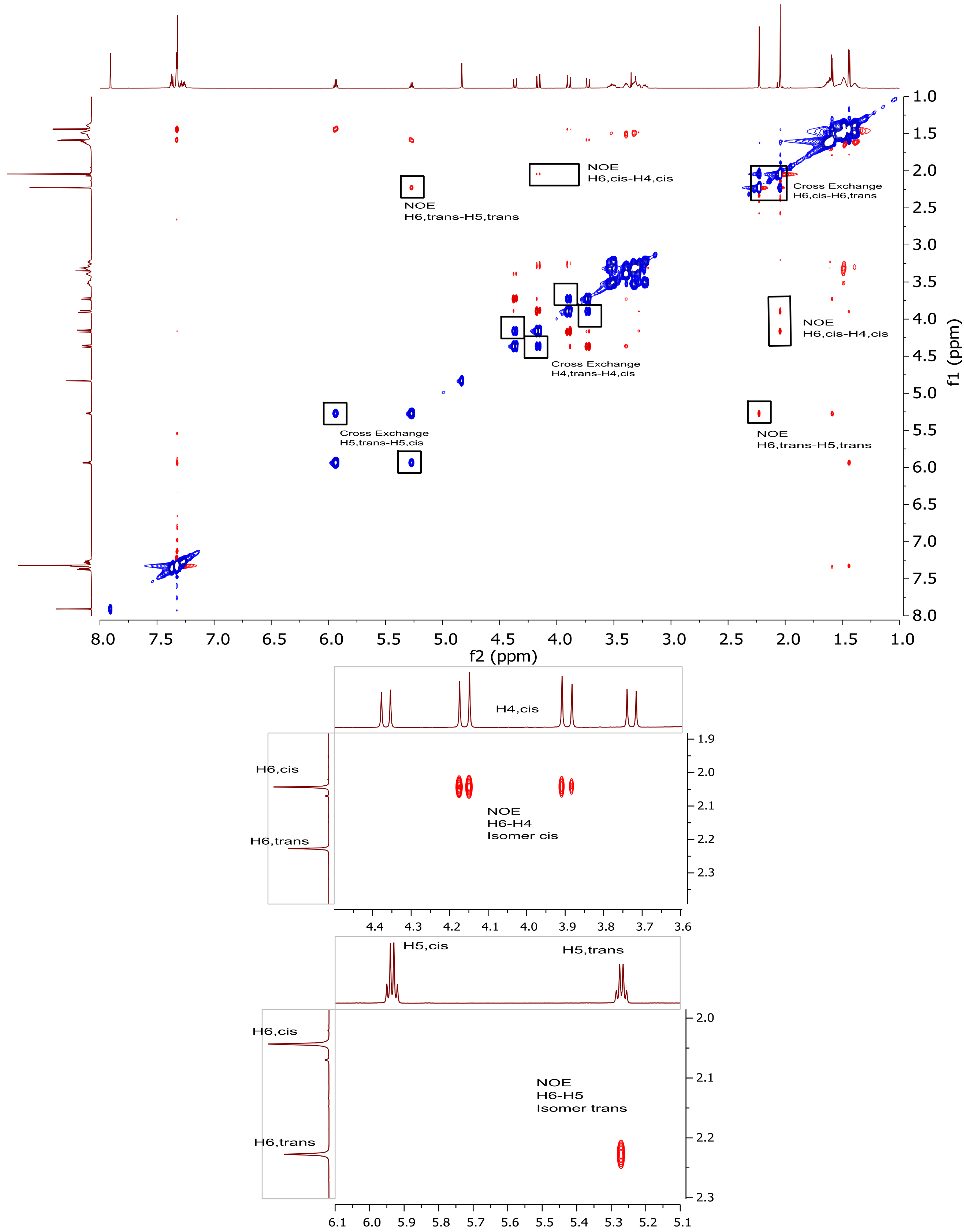

Figure S-108. ^{1}H-^{1}H NOESY spectrum of compound **14** recorded in *methanol-d$_4$* at room temperature. Inset showing the specific spatial contacts that are characteristic of each isomer and allowed the identification of *cis* and *trans* signals.

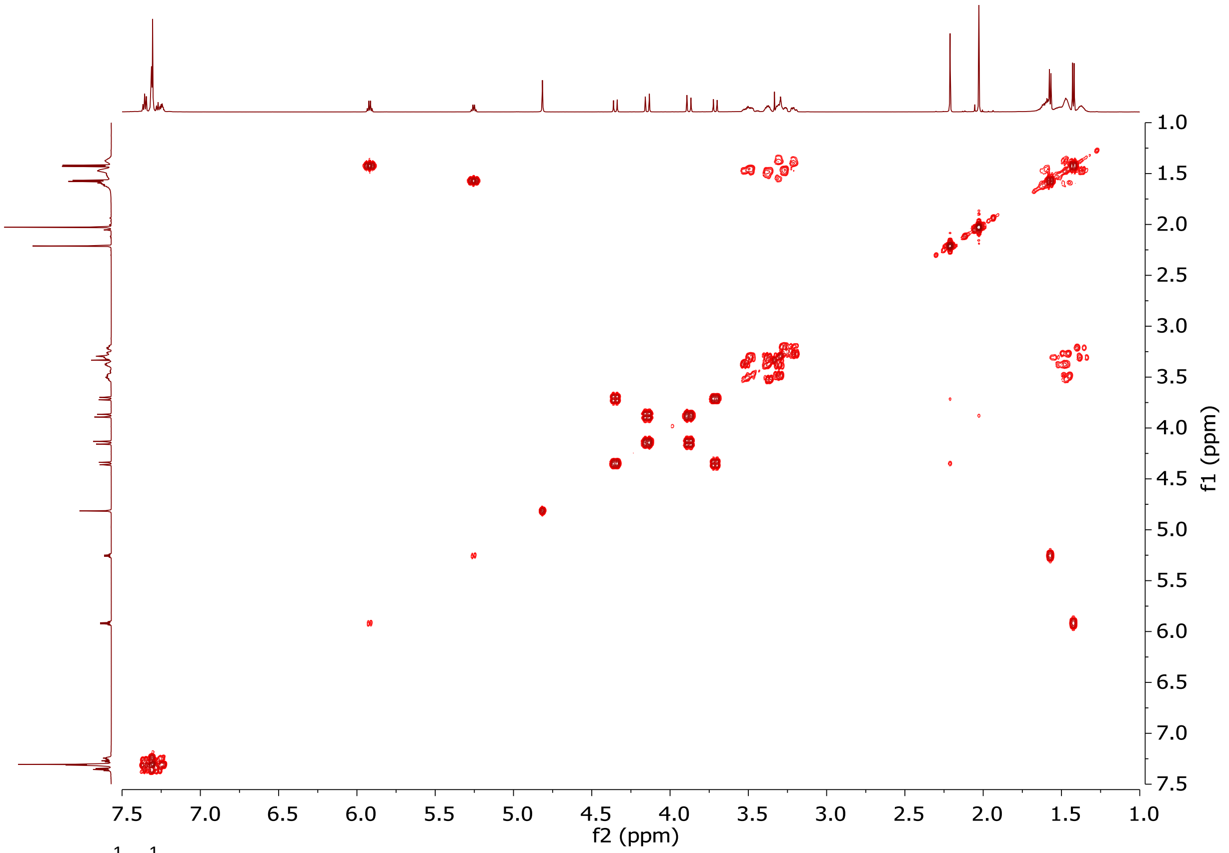

Figure S-109. ^{1}H-^{1}H COSY spectrum of compound **14** recorded in *methanol-d_4* at room temperature.

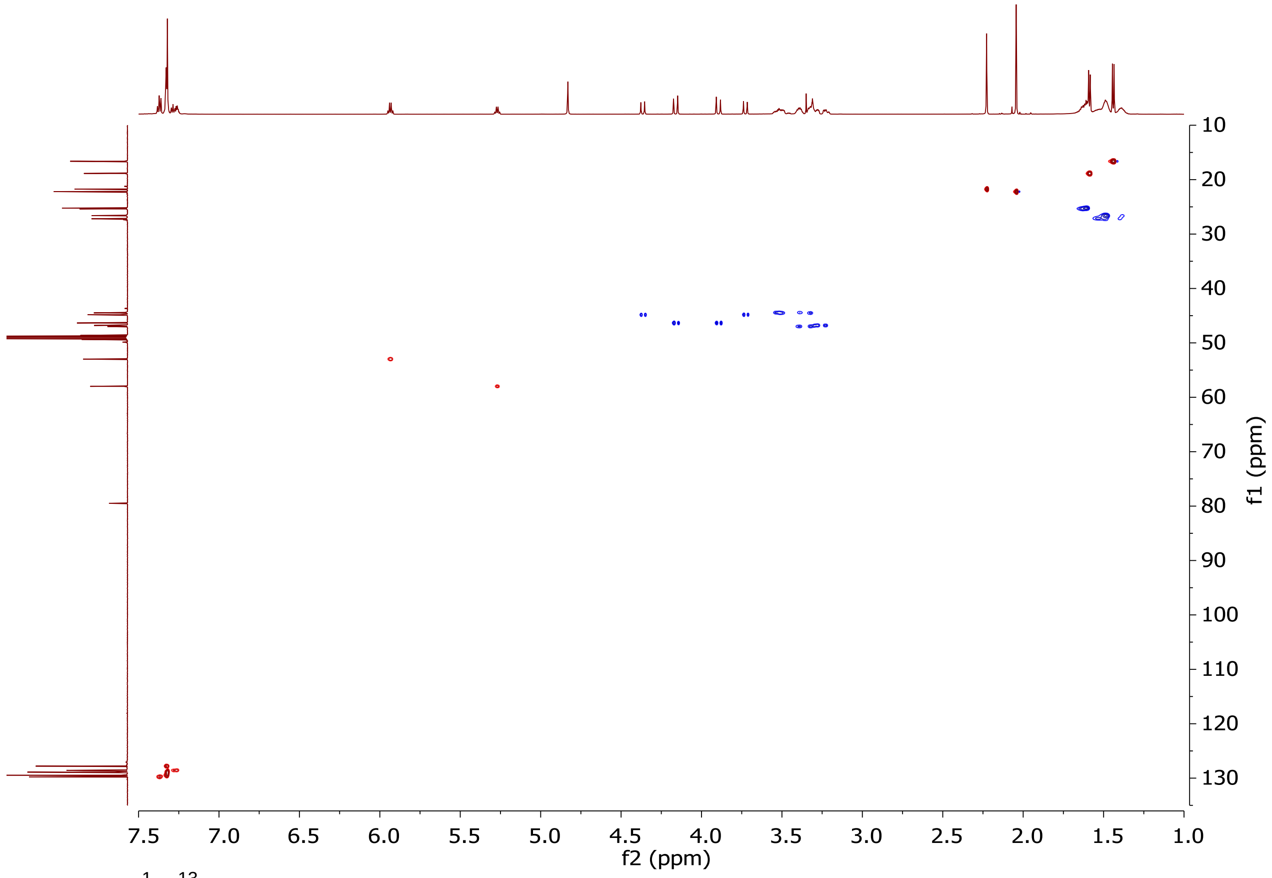

Figure S-110. ^{1}H-^{13}C HSQC spectrum of compound **14** recorded in *methanol-d_4* at room temperature.

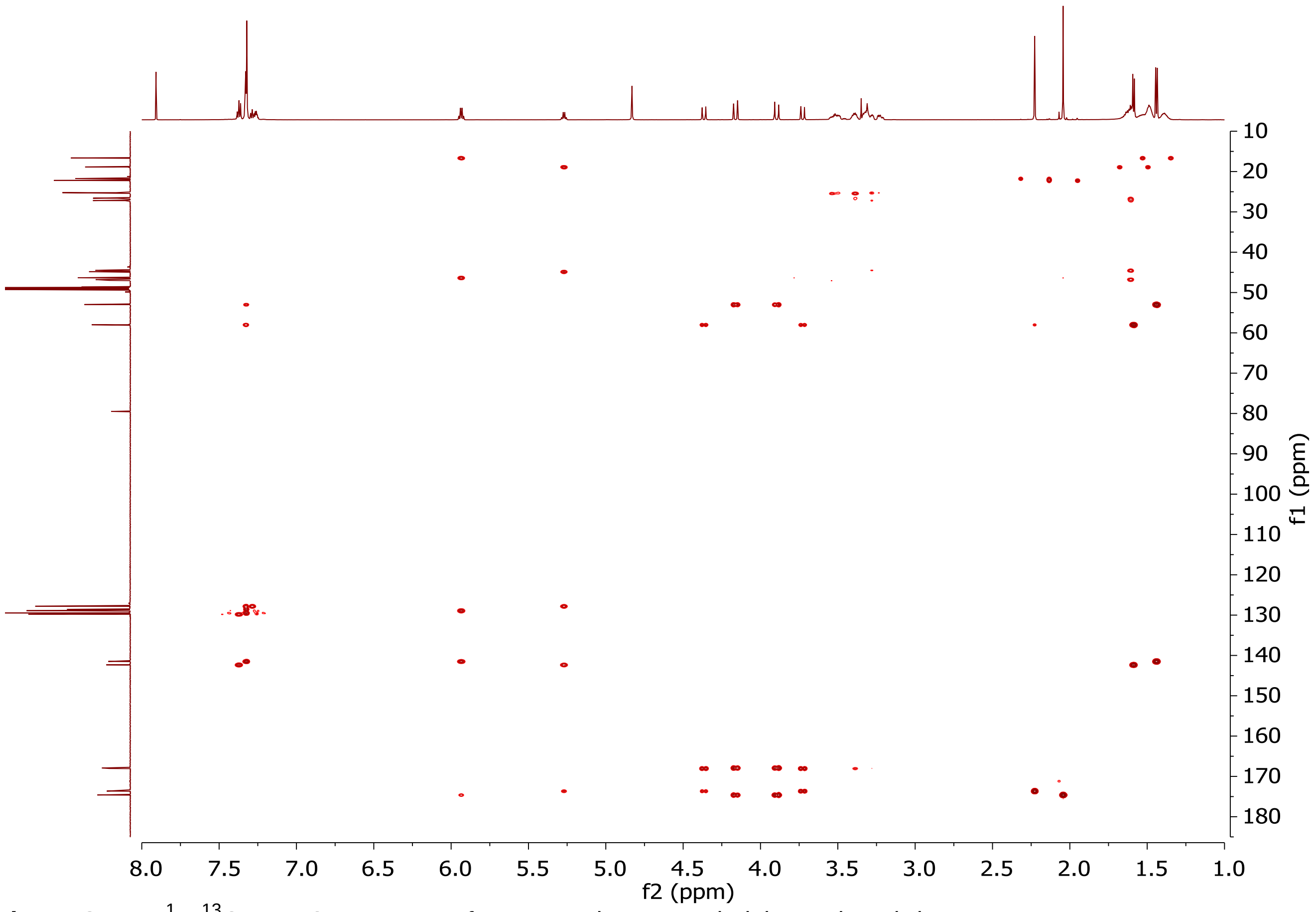

Figure S-111. ^{1}H-^{13}C HMBC spectrum of compound **14** recorded in *methanol-d_4* at room temperature.

^{1}H NMR (700MHz, Chloroform-*d*): <u>Rotamer *trans*</u>: δ ^{1}H NMR: 7.35-7.18 (m, 5H, $H_{8\text{-}10,trans}$), 5.13 (q, *J* = 6.9 Hz, 1H, $H_{5,trans}$), 4.37, 3.34 (AB peak, *J* = 15.9 Hz, 2H, $H_{4,trans}$), 3.53-3.07 (m, 4H, $H_{3,ax+eq,trans}$), 2.20 (s, 3H, $H_{6,trans}$), 1.60-1.40 (m, 6H, $H_{1\text{-}2,ax+eq,trans}$), 1.60 (d, *J* = 7.0 Hz, 3H, $H_{7,trans}$). <u>Rotamer *cis*</u>: δ ^{1}H NMR: 7.35-7.18 (m, 5H, $H_{8\text{-}10,trans}$), 6.07 (q, *J* = 7.0 Hz, 1H, $H_{5,cis}$), 3.81, 3.62 (AB peak, *J* = 17.7 Hz, 2H, $H_{4,cis}$), 3.53-3.07 (m, 4H, $H_{3,ax+eq,cis}$), 2.02 (s, 3H, $H_{6,cis}$), 1.60-1.40 (m, 6H, $H_{1\text{-}2, ax+eq,cis}$), 1.39 (d, *J* = 7.1 Hz, 3H, $H_{7,cis}$).

^{13}C NMR (176 MHz, Chloroform-*d*): <u>Rotamer *trans*</u>: δ ^{13}C NMR: 128.63, 127.42, 126.38 (CH, $C_{8\text{-}10,trans}$), 56.25 (CH, $C_{5,trans}$), 45.63, 43.28 (CH_2, $C_{3,trans}$), 43.59 (CH_2, $C_{4,trans}$), 26.15 25.39, 24.36 (CH_2, $C_{1\text{-}2,trans}$), 21.62 (CH_3, $C_{6,trans}$), 18.16 (CH_3, $C_{7,trans}$); <u>Rotamer *cis*</u>: δ ^{13}C NMR: 128.26, 127.61, 127.24 (CH, $C_{8\text{-}10,cis}$), 50.84 (CH, $C_{5,cis}$), 45.89, 43.15 (CH_2, $C_{3,cis}$), 45.02 (CH_2, $C_{4,cis}$), 26.07, 25.32, 24.18 (CH_2, $C_{1\text{-}2,cis}$), 21.96 (CH_3, $C_{6,cis}$), 15.93 (CH_3, $C_{7,cis}$); <u>Quaternary carbons</u>: δ ^{13}C NMR: 171.66 (**C**O-$CH_{3,cis}$), 170.76 (**C**O-$CH_{3,trans}$), 166.09 (**C**O-$pip_{,trans}$), 165.86 (**C**O-$pip_{,cis}$), 140.99 (**C**$_{Ar,trans}$), 140.60 (**C**$_{Ar,cis}$).

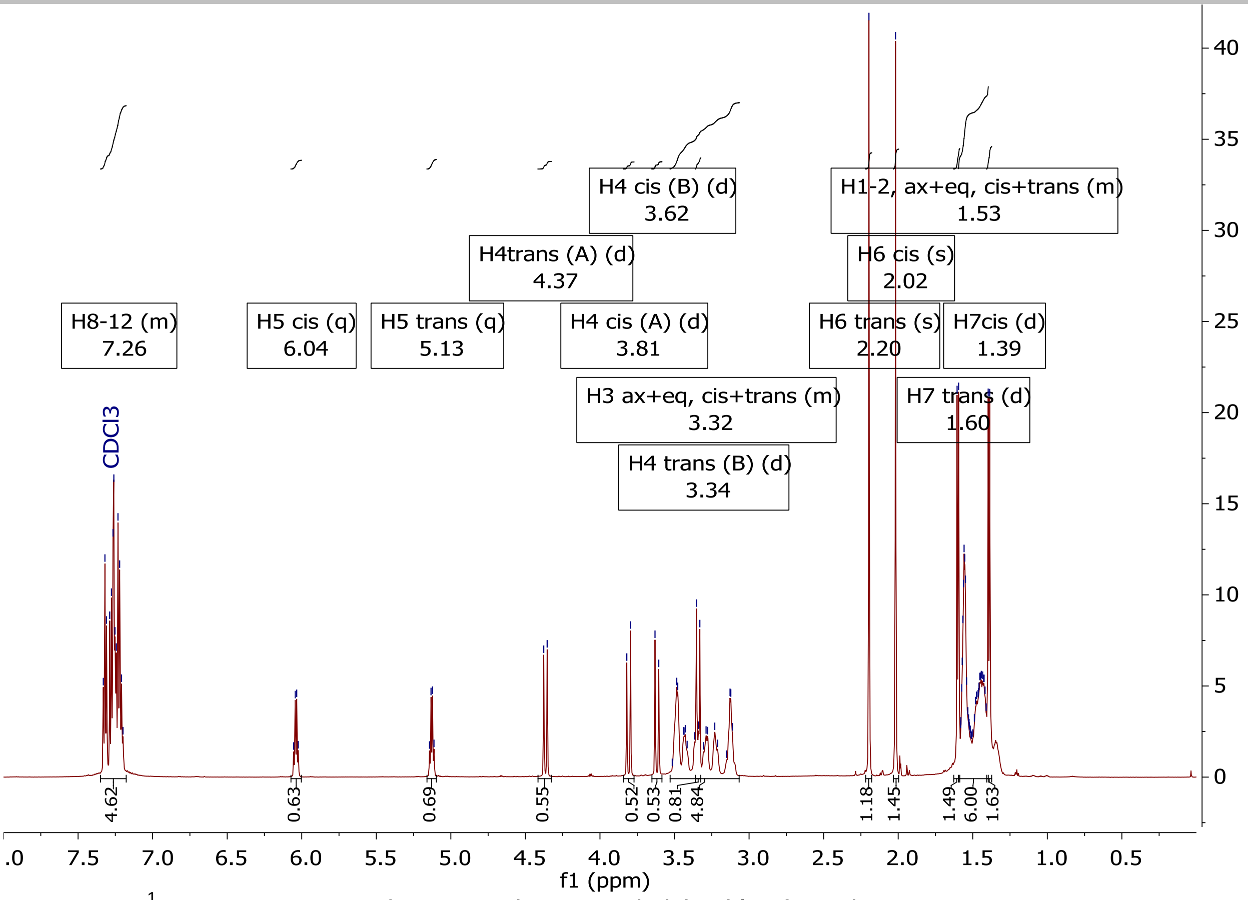

Figure S-112. ^{1}H-NMR spectrum of compound **14** recorded in chloroform-*d* at room temperature.

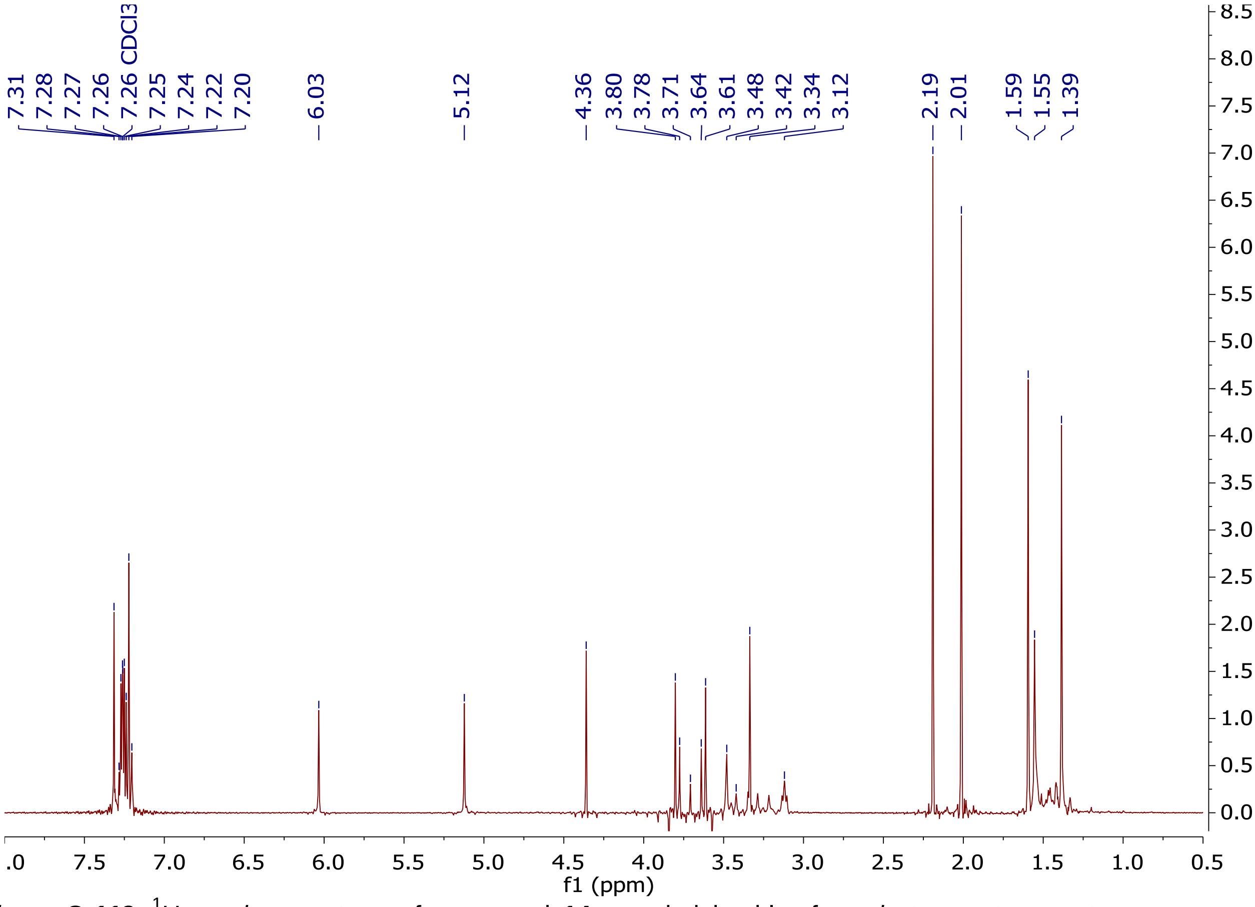

Figure S-113. ^{1}H-*psyche* spectrum of compound **14** recorded in chloroform-*d* at room temperature.

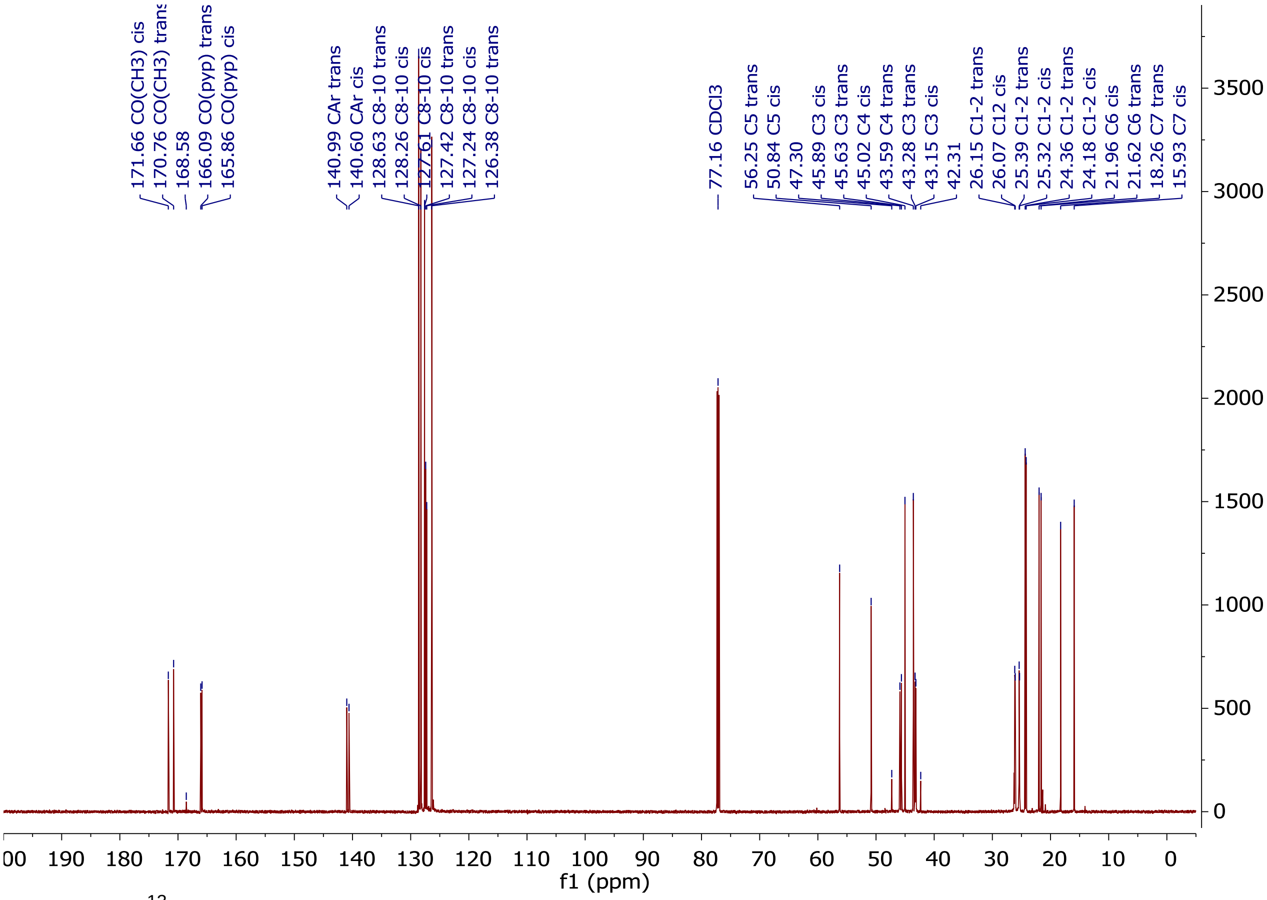

Figure S-114. ^{13}C NMR spectrum of compound **14** recorded in chloroform-*d* at room temperature.

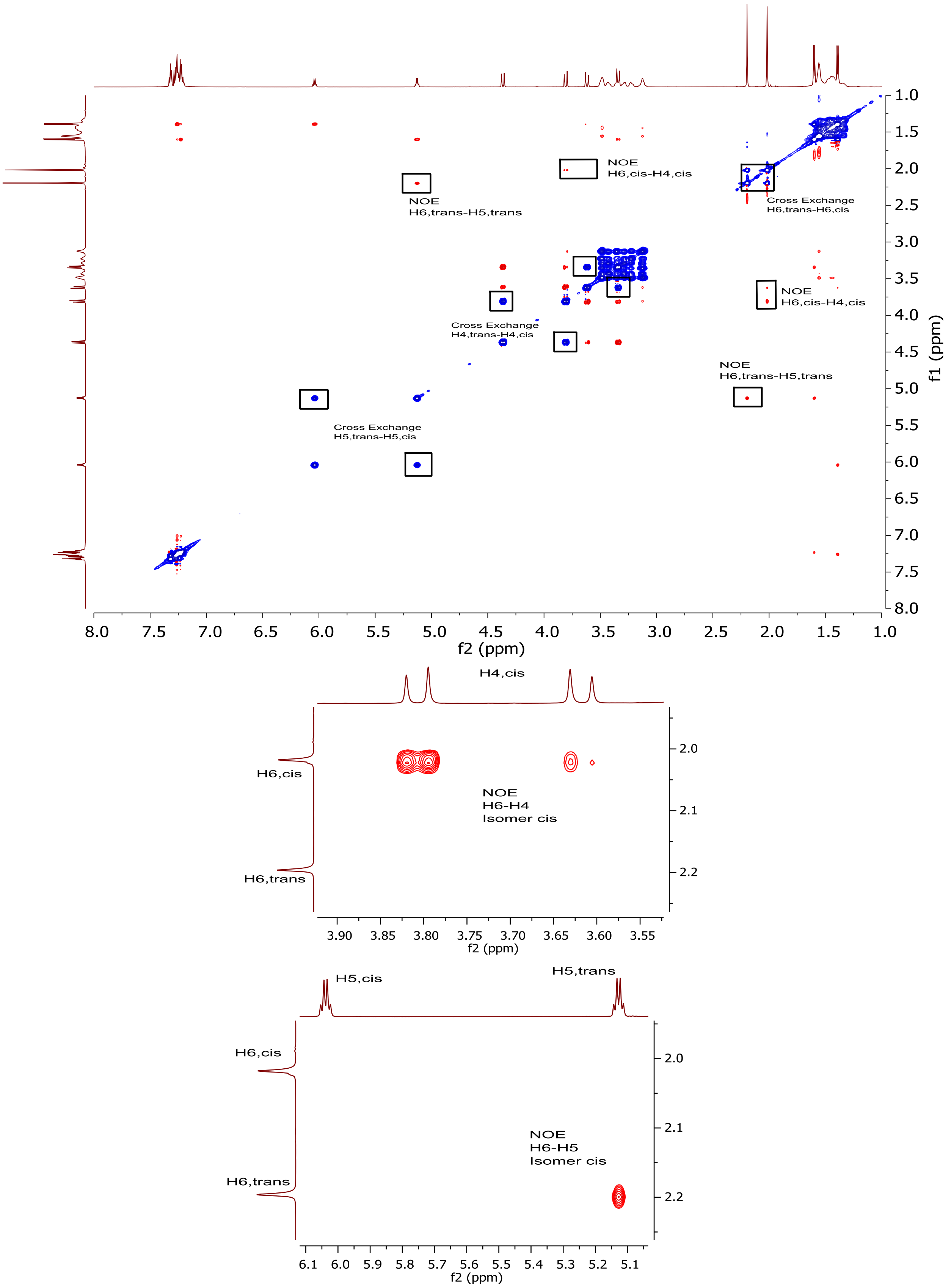

Figure S-115. ^{1}H-^{1}H NOESY spectrum of compound **14** recorded in chloroform-*d* at room temperature. Inset showing the specific spatial contacts that are characteristic of each isomer and allowed the identification of *cis* and *trans* signals.

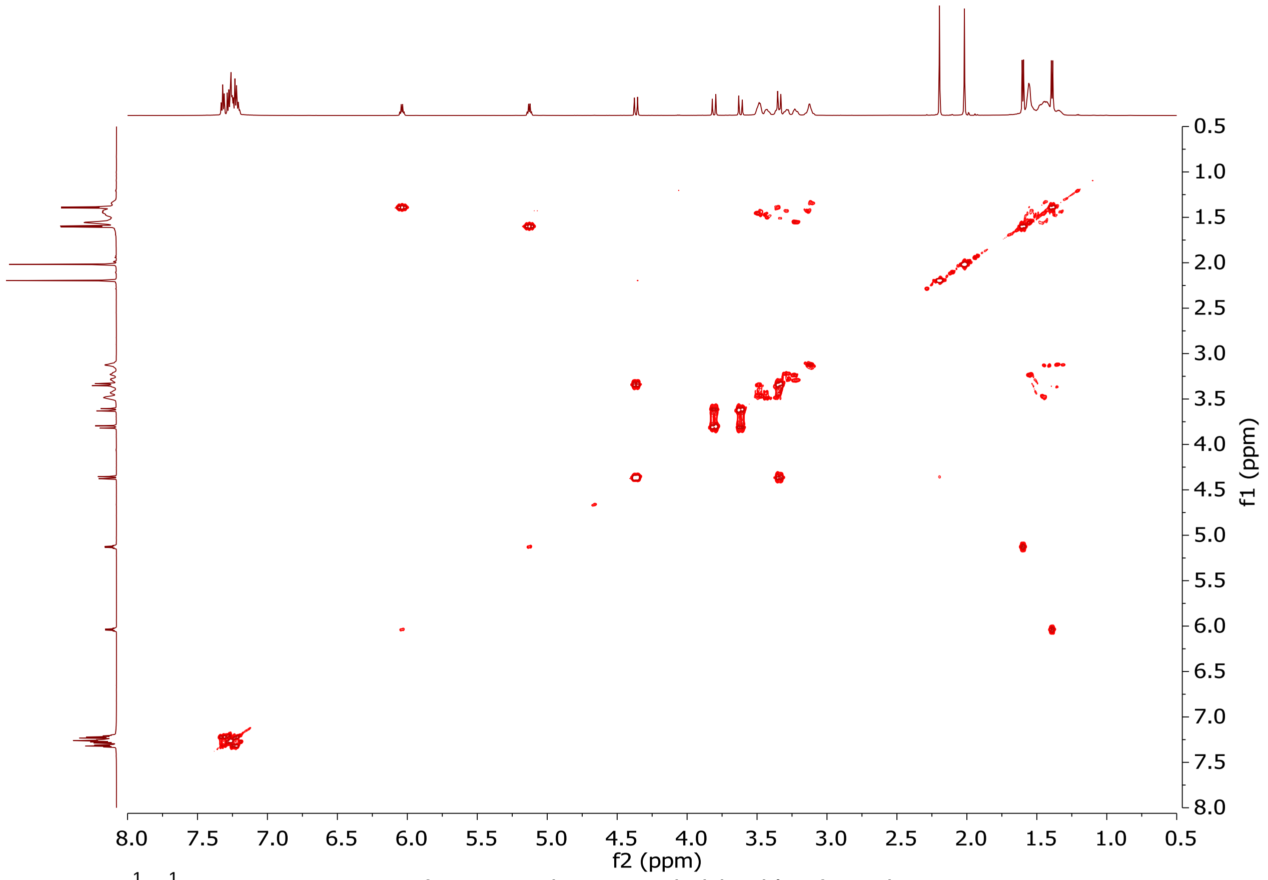

Figure S-116. ^{1}H-^{1}H COSY spectrum of compound **14** recorded in chloroform-*d* at room temperature.

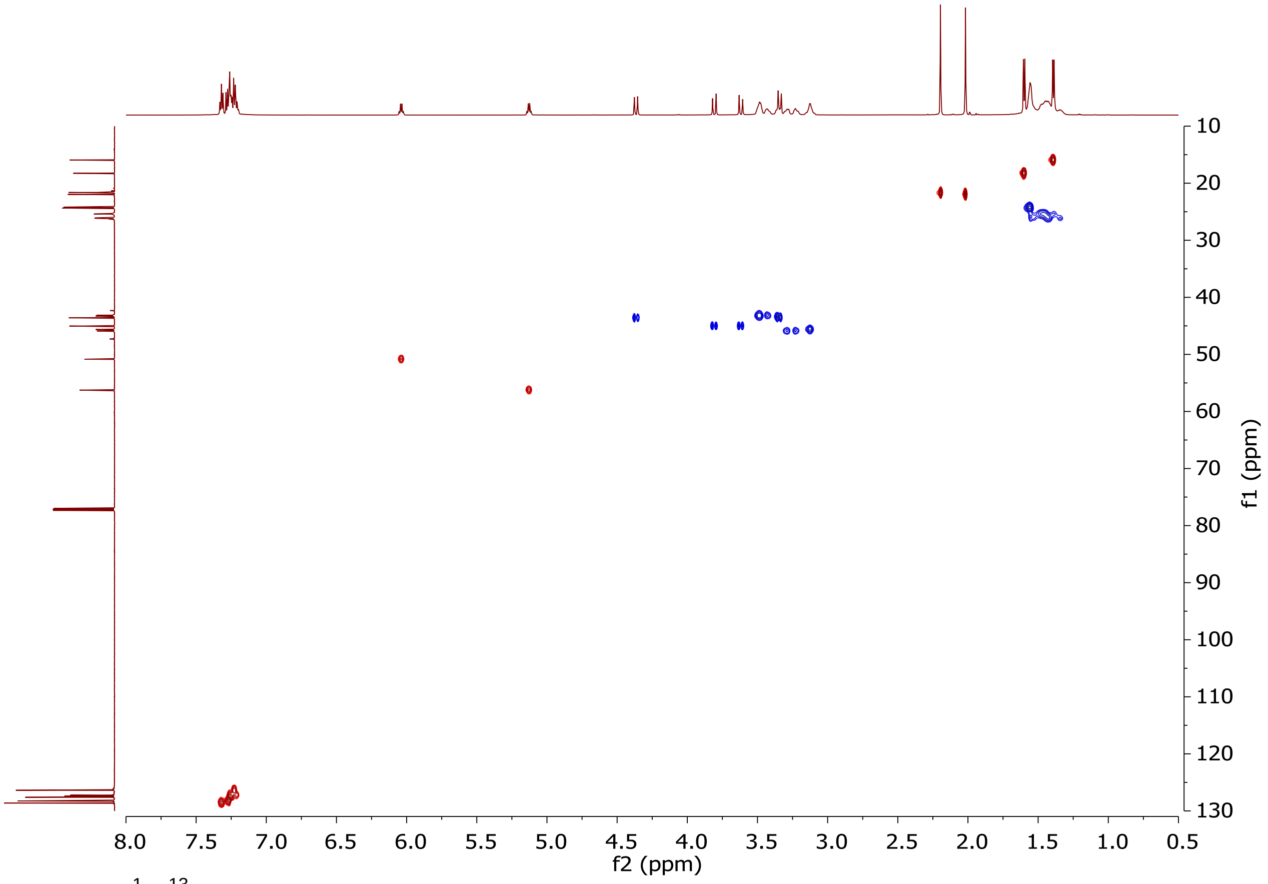

Figure S-117. ^{1}H-^{13}C HSQC spectrum of compound **14** recorded in chloroform-*d* at room temperature

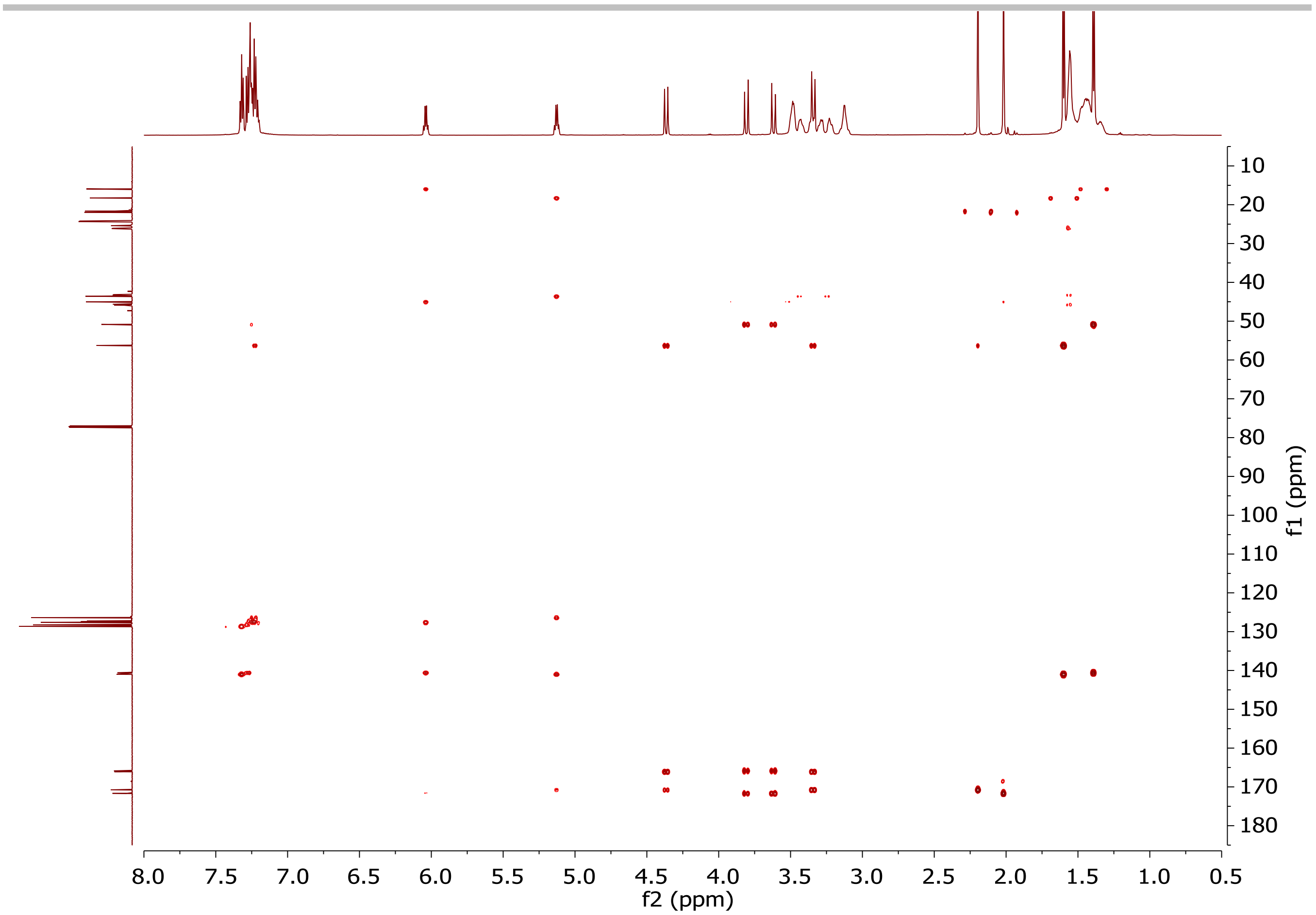

Figure S-118. ^{1}H-^{13}C HMBC spectrum of compound **14** recorded in chloroform-*d* at room temperature.

$K_{cis/trans}$ numeric evaluation:

General procedure for NMR $K_{cis/trans}$ determination: $K_{cis/trans}$ has been in all cases determined using at least two ^{1}H-NMR spectrums, and it is reported as the average value of the ratio between the *cis* and *trans* pairs of signals arising from the three main protons involved in the isomerization process: the backbone methylene protons (in general denoted as H4 through all the structures), the side chain methylene protons (H5) and the methyl-acetyl hydrogens (H6). Mean values are given with the corresponding standard deviation for n=6, otherwise the corresponding sampling numbers has been noted in the main text. In fluorinated systems, where $K_{cis/trans}$ could also be evaluated independently from ^{19}F-NMR experiments, a second average value of the equilibrium constant is reported separately for comparison and verification purposes. Good agreement was obtained in all cases. A complete set of the Individually assessed and averaged values observed in each solvent is given in following supporting Tables S1-S3.

Supplementary Table 1. NMR (CD_3CN) spectroscopic evaluation of the amide *cis/trans* equilibrium in peptoid dimers 10-14

Peptoid	^{1}H-NMR (CD_3CN)						$^{AVG}K_{cis/trans}$	$^{AVG}\Delta G_{cis/trans}$ (kcal/mol)	^{19}F-NMR (CD_3CN) $K_{cis/trans}$
	$^1H_{4,cis/trans}$ (1)	$^1H_{4,cis/trans}$ (2)	$^1H_{5,cis/trans}$ (1)	$^1H_{5,cis/trans}$ (2)	$^1H_{6,cis/trans}$ (1)	$^1H_{6,cis/trans}$ (2)			
**10**	0.65	0.67	0.48	0.58	0.69	0.68	0.66±0.07	0.28±0.08	-
**11**	1.19	1.11	1.32	1.22	1.08	1.08	1.15±0.09	-0.09±0.04	1.07
**12**	2.00	2.21	2.11	1.95	2.10	1.83	2.05±0.12	-0.42±0.04	2.02
**13**	2.45	2.33	2.15	2.15	-	-	2.24±0.12	-0.48±0.03	2.36
**14**	2.05	2.17	1.84	2.05	2.22	2.11	2.08±0.12	-0.43±0.04	-

Supplementary Table 2. NMR (CD_3OD) spectroscopic evaluation of the amide *cis/trans* equilibrium in peptoid dimers 10-14

Product	^{1}H-NMR (CD_3OD)						$^{AVG}K_{cis/trans}$	$^{AVG}\Delta G_{cis/trans}$ (kcal/mol)	^{19}F-NMR (CD_3OD) $K_{cis/trans}$
	$^1H_{4,cis/trans}$ (1)	$^1H_{4,cis/trans}$ (2)	$^1H_{5,cis/trans}$ (1)	$^1H_{5,cis/trans}$ (2)	$^1H_{6,cis/trans}$ (1)	$^1H_{6,cis/trans}$ (2)			
**10**	0.48	0.50	0.52	0.52	0.41	0.52	0.51±0.04	0.43±0.05	-
**11**	0.76	0.70	0.80	0.91	0.72	0.73	0.74±0.07	0.16±0.05	1.07
**12**	1.21	1.16	1.22	1.11	1.15	1.18	1.17±0.04	-0.09±0.02	2.02
**13**	1.27	1.23	1.30	1.24	1.23	1.22	1.23±0.03	-0.13±0.01	2.36
**14**	1.37	1.30	1.40	1.40	1.34	1.32	1.35±0.04	-0.18±0.02	-

Supplementary Table 3. NMR($CDCl_3$) spectroscopic evaluation of the amide *cis/trans* equilibrium in peptoid dimers 10.14

Product	^{1}H-NMR ($CDCl_3$)						$^{AVG}K_{cis/trans}$	$^{AVG}\Delta G_{cis/trans}$ (kcal/mol)	^{19}F-NMR ($CDCl_3$) $K_{cis/trans}$
	$^1H_{4,cis/trans}$ (1)	$^1H_{4,cis/trans}$ (2)	$^1H_{5,cis/trans}$ (1)	$^1H_{5,cis/trans}$ (2)	$^1H_{6,cis/trans}$ (1)	$^1H_{6,cis/trans}$ (2)			
**10**	0.20	0.20	-	-	0.19	0.19	0.19±0.01[a]	0.97±0.01	-
**11**	0.80	0.72	-	-	0.77	0.74	0.76±0.03	0.16±0.02	0.72
**12**	1.24	1.24	1.44	1.41	1.27	1.30	1.28±0.08	-0.16±0.03	1.20
**13**	0.65	0.50	-	-	0.58	0.49	0.54±0.06	0.35±0.007	1.30
**14**	0.96	0.94	0.91	0.98	0.81	0.94	0.94±0.05	0.05±0.04	-

Analysis of the NMR vicinal $^3J_{H\text{-}F}$ coupling constants:

In order to gain further evidence regarding its possible *gauche* structural disposition, a qualitative analysis was performed based in the comparison of the observed vicinal $^3J_{HF}$ coupling constants and the theoretical limit values of the corresponding possible staggered conformations (no conformational bias), as reported through the main text and schematized in **Figure 3**. As highlighted, this analysis may be considered merely an indicative of the structure in solution rather than an evidence, even through in cases where the coupling constants between the model staggered conformations show a high difference in value, it provides acurate information regarding the overall preferred disposition of the F atoms.[3,4] For the calculations, we have employed in all cases the limit values as taken from Ihrig and Smith,[5] and used by O'Hagan and co-workers in similar systems.[6] Limit values are: $^3J_{HF,anti}$= 32 Hz and $^3J_{HF,gauche}$ = 8 Hz. Note that the values may be understood as a qualitative approximation. The complete set of observed vicinal coupling $^3J_{HF,obs}$ for each *cis/trans* structure and solvent is summarized in following supplementary Table S4.

$^3J_{HF,calc}$ (**11-*gauche***)= [$^3J_{HF,anti}$ + 2 x $^3J_{HF,gauche}$] / 2 = 20 Hz
$^3J_{HF,calc}$ (**11-*anti***)= [2 x $^3J_{HF,gauche}$] / 2 = 8 Hz

$^3J_{HF,calc}$ (**12-(+/-)*gauche***)= [$^3J_{HF,anti}$ + 2 x $^3J_{HF,gauche}$] / 2 = 20 Hz
$^3J_{HF,calc}$ (**12-(*anti, (+/-)gauche)***)= [$^3J_{HF,anti}$ + 3 x $^3J_{HF,gauche}$] / 4 = 14 Hz

$^3J_{HF,calc}$ (**13-(+*gauche, anti, -gauche)***)= [2 x $^3J_{HF,anti}$ + 4 x $^3J_{HF,gauche}$] / 6 = 16 Hz

Entry	$^3J_{H\text{-}F}$ (CDCl$_3$)				$^3J_{H\text{-}F}$ (CD$_3$CN)				$^3J_{H\text{-}F}$ (CD$_3$OD)			
	^{1}H-*psyche*		^{19}F-NMR		^{1}H- *psyche*		^{19}F-NMR		^{1}H- *psyche*		^{19}F-NMR	
	cis	*trans*	*cis*	*trans*	*cis*	*trans*	*cis*	*trans*	*cis*	*trans*	*cis*	*trans*
**11**	28.78	25.79	28.80	25.78	25.57	25.83	25.68	25.89	26.17	26.12	26.11	26.13
**12**	14.17	14.60	14.26	14.64	14.83	14.82	14.87	14.82	14.49	14.76	14.49	14.77
**13**	-	-	9.28	8.75	9.56	9.06	9.57	9.04	9.30	8.85	9.33	8.87

Table S-4. Experimental vicinal $^3J_{H\text{-}F}$ coupling constants determined for *cis* and *trans* isomers of model acetamides **11-13**, in each corresponding solvent. For its comparison, the evaluation in the basis of ^{1}H-NMR (*psyche*) and ^{19}F-NMR signals are given separately.[7,8]

X-Ray Crystal structure data:

Sample crystallization: 20 mg of the purified compounds were dissolved using a minimum amount of AcOEt and then hexane was added dropwise until precipitation was observed. Further AcOEt was then added to clear solutions. Samples were then left for slow evaporation at room temperature.

Crystallographic data of compound 12: Ac-^{2F}Et-pip

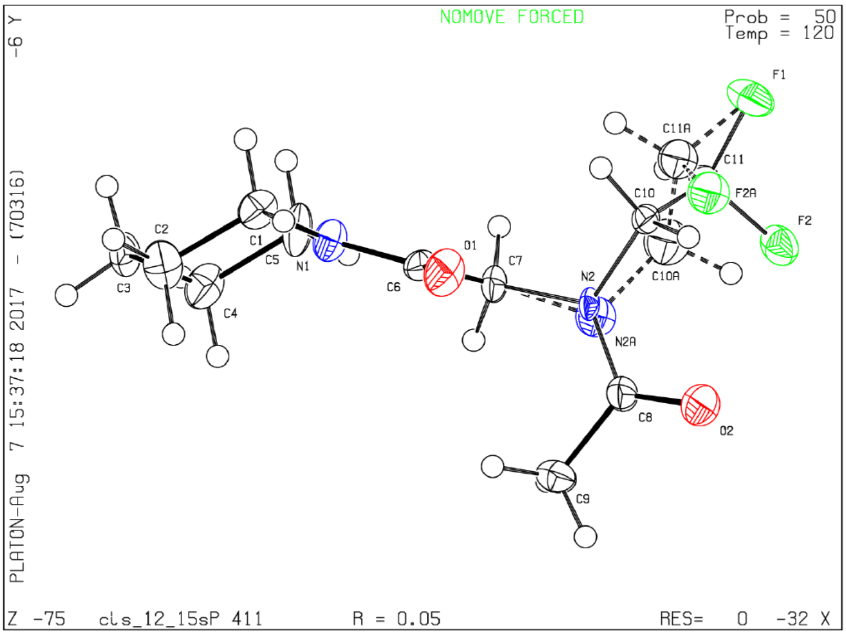

Figure S-119. Crystal structure of peptoid dimer **12** (CCDC-1567326) with thermal ellipsoids drawn at the 50 % probability level.

Table S-5. Crystal data and structure refinement parameters of peptoid dimer **12**

**Empirical formula**	$C_{11}H_{18}F_2N_2O_2$	**μ/mm^{-1}**	0.113
**Formula weight**	248.27	**F(000)**	528.0
**Temperature/K**	120.0	**Crystal size/mm^3**	0.29 × 0.28 × 0.12
**Crystal system**	tetragonal	**Radiation**	MoKα (λ = 0.71073)
**Space group**	$P4_1$	**2Θ range for data collection/°**	4.56 to 57
**a/Å**	8.9275(4)	**Index ranges**	-11 ≤ h ≤ 11, -11 ≤ k ≤ 11, -20 ≤ l ≤ 20
**b/Å**	8.9275(4)	**Reflections collected**	24571
**c/Å**	15.3498(8)	**Independent reflections**	1600 [R_{int} = 0.0394, R_{sigma} = 0.0172]
**α/°**	90.00	**Data/restraints/parameters**	1600/11/172
**β/°**	90.00	**Goodness-of-fit on F^2**	1.076
**γ/°**	90.00	**Final R indexes [I>=2σ (I)]**	R_1 = 0.0546, wR_2 = 0.1417
**Volume/Å^3**	1223.38(10)	**Final R indexes [all data]**	R_1 = 0.0562, wR_2 = 0.1432
**Z**	4	**Largest diff. peak/hole / e Å^{-3}**	0.43/-0.28
**ρ_{calc}g/cm^3**	1.348		

Crystallographic data of compound 13: Ac-^{3F}Et-pip

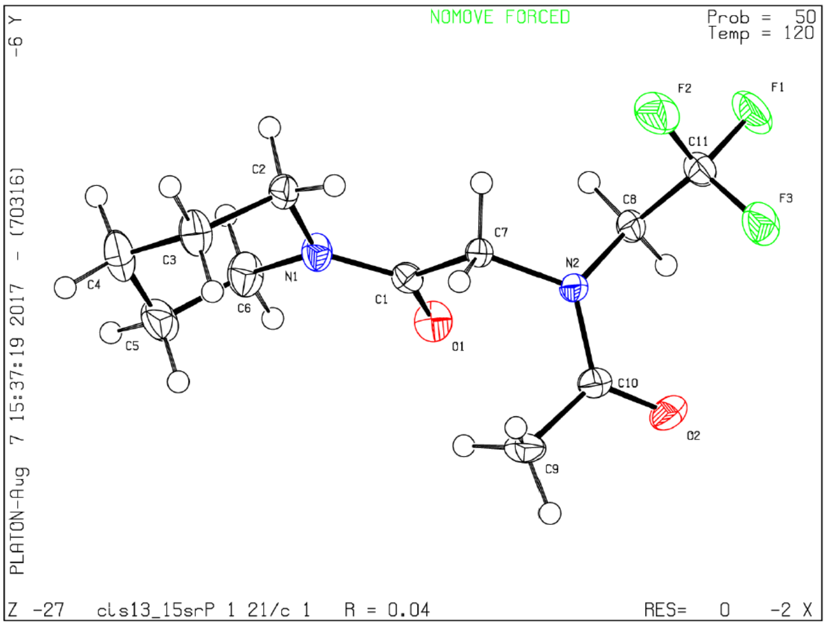

Figure S-120. Crystal structure of peptoid dimer **13** (CCDC-1567327) with thermal ellipsoids drawn at the 50 % probability level.

Table S-6. Crystal data and structure refinement parameters of peptoid dimer **13**

**Empirical formula**	$C_{11}H_{17}F_3N_2O_2$	**μ/mm^{-1}**	0.121
**Formula weight**	266.26	**F(000)**	560.0
**Temperature/K**	120	**Crystal size/mm^3**	0.361 × 0.257 × 0.056
**Crystal system**	monoclinic	**Radiation**	MoKα (λ = 0.71073)
**Space group**	$P2_1/c$	**2Θ range for data collection/°**	4.994 to 60.18
**a/Å**	11.6819(5)	**Index ranges**	-16 ≤ h ≤ 16, -16 ≤ k ≤ 16, -13 ≤ l ≤ 13
**b/Å**	11.6693(5)	**Reflections collected**	26521
**c/Å**	9.8470(4)	**Independent reflections**	3857 [R_{int} = 0.0361, R_{sigma} = 0.0261]
**α/°**	90	**Data/restraints/parameters**	3857/0/165
**β/°**	102.4584(16)	**Goodness-of-fit on F^2**	1.025
**γ/°**	90	**Final R indexes [I>=2σ (I)]**	R_1 = 0.0390, wR_2 = 0.0967
**Volume/Å^3**	1310.73(10)	**Final R indexes [all data]**	R_1 = 0.0510, wR_2 = 0.1037
**Z**	4	**Largest diff. peak/hole / e Å^{-3}**	0.35/-0.29
**ρ_{calc}g/cm^3**	1.349		

Model peptoid synthesis and characterization

Synthesis of peptoid oligomers Pep1-7: All peptoids were synthetized using the well described sub-monomer approach. Fmoc-protected Rink Amide resin (50mg, loading 0.84mol g^{-1}) was swollen in DMF and de-protected with piperidine for 15 minutes at room temperature (20% in DMF, 2 mL). The resin was then treated with bromoacetyl bromide (1 mL, 0.6M in DMF) and DIC (0.2 mL, 50% v/v in DMF) for 30 min and washed with DMF (4X, 2 mL) before the desired amine sub-monomer was added (1 mL, 1.2M in DMF), the mixture shaken for 60-120 min, and the resin washed again (DMF, 2mL, 4X). Bromoacetylation and bromine displacement steps were repeated until the target sequence was achieved. Final peptoids were cleaved off the resin by treatment with a 95:2.5:2.5% v/v TFA:TIPS:H_2O solution (1mL) for 15 min while shaking at RT, after what the cleavage cocktail was filtered over diethyl ether (15mL) and the solid residue let for precipitation overnight at -20°C. The crude peptoids obtained by decanting the ether layer were further washed with chilled Et_2O prior to be dissolved in water and freeze-dried overnight. Purification of the peptoids was achieved by reverse phase semi-preparative HPLC and their identity confirmed by Maldi-TOF and HR-(QToF)-LC/MS. Final purity of the target compounds was found to be in all cases >95%, as assessed by analytical HPLC at λ=220nm.

HPLC, Maldi-TOF and HR-MS Characterization

Table S-7. Characterization data of peptoid oligomers **Pep.1-Pep.7.**

Peptoid	Sequence	Analytical HPLC		Estimated Yields		Acc. Mass spectrometry	
		Retention time (min)	Purity (%)	Isolated mass (mg)	Yield (%)	Calc. Mass (Da)	Obs. Mass (Da)
**Pep.1**	[NLys-NEt-NSpe][NLys-NEt-NEt]$_4$	10.15	$\geq$95%	21.2	26.7	$[M+2H]^{2+}$, 793.0369	793.0380
**Pep.2**	[NLys-N*1f*Et-NSpe][NLys-N*1f*Et-NEt]$_4$	10.40	$\geq$95%	16.8	20.1	$[M+H]^{+}$, 1675.0210	1675.0192
**Pep.3**	[NLys-N*2f*Et-NSpe][NLys-N*2f*Et-NEt]$_4$	10.99	$\geq$98%	13.7	15.6	$[M+2H]^{2+}$, 882.9908	882.9882
**Pep.4**	[NLys-N*3f*Et-NSpe][NLys-N*3f*Et-NEt]$_4$	12.88	$\geq$98%	8.2	8.8	$[M+2H]^{2+}$, 927.9673	927.9654
**Pep.5**	[NLys-N*1f*Et-NSpe][NLys-N*1f*Et-N*1f*Et]$_4$	9.48	$\geq$98%	13.3	15.2	$[M+H]^{+}$, 1746.9833	1746.9822
**Pep.6**	[NLys-N*2f*Et-NSpe][NLys-N*2f*Et-N*2f*Et]$_4$	12.43	$\geq$95%	7.7	8.1	$[M+H]^{+}$, 1908.8986	1908.8895
**Pep.7**	[NLys-N*3f*Et-NSpe][NLys-N*3f*Et-N*3f*Et]$_4$	14.16	$\geq$98%	17.9	17.3	$[M+2H]^{2+}$, 1035.9108	1035.9092

Pep.1: [NLysNEtNSpe] [NLysNEtNEt]$_4$.

Analytical HPLC:

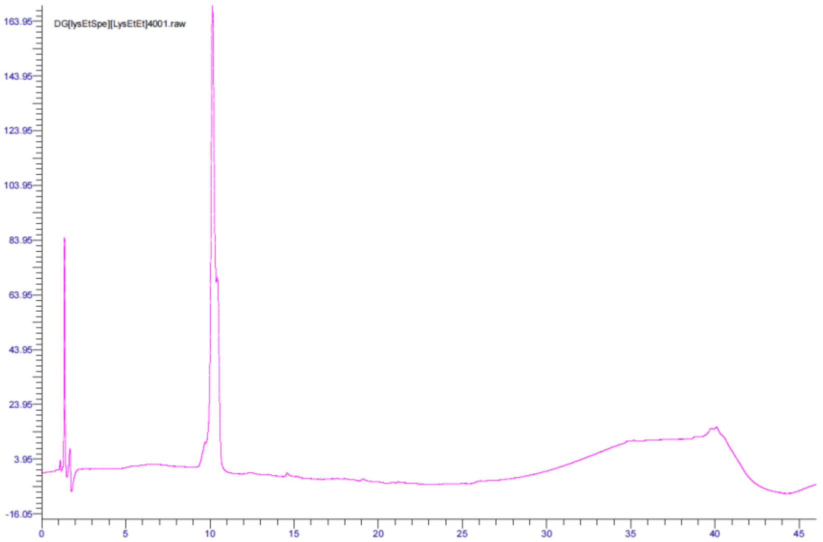

Figure S-120. Analytical HPLC trace of model peptoid **Pep.1** at λ=220nm.

Maldi-ToF:

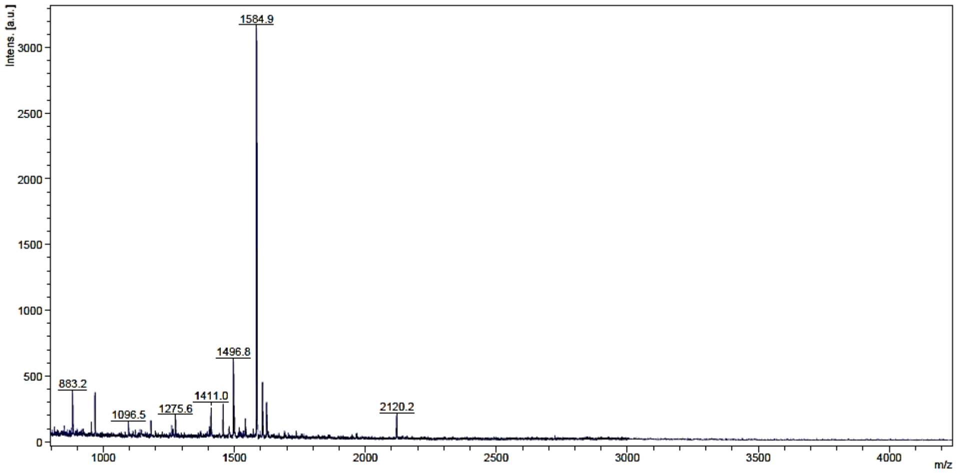

Figure S-121. Maldi-ToF spectra acquired for model peptoid **Pep.1**.

HRMS:

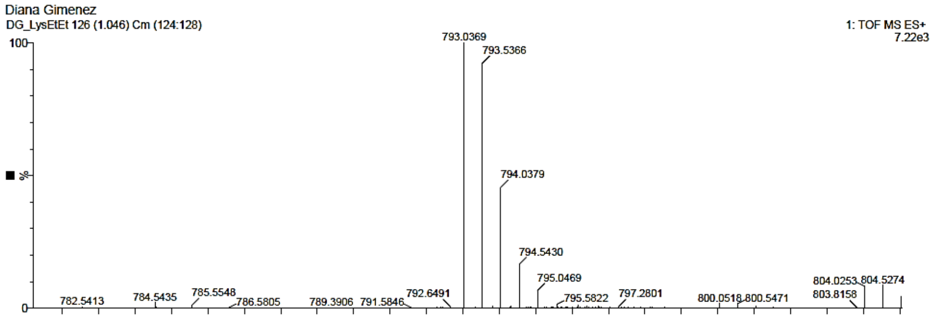

Figure S-122. HRMS (QToF-MS-ES+) trace of **Pep.1** .

Pep.2: [NLysN1fEtNSpe] [NLysN1fEtNEt]$_4$.

Analytical HPLC:

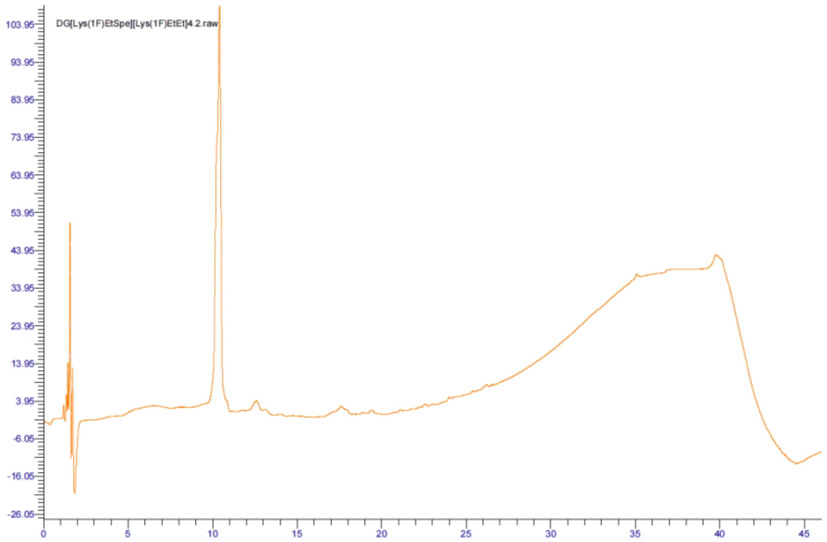

Figure S-123. Analytical HPLC trace of model peptoid **Pep.2** at λ=220nm.

Maldi-ToF:

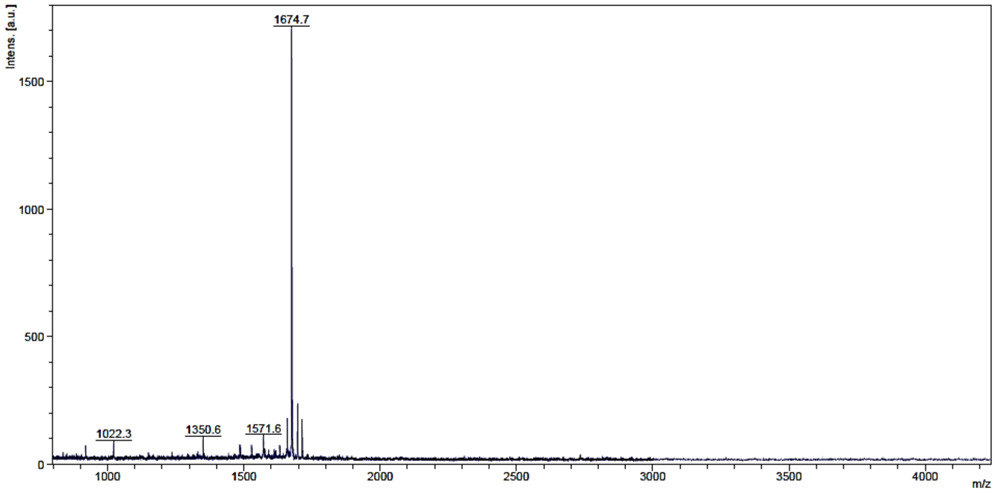

Figure S-124. Maldi-ToF spectra acquired for model peptoid **Pep.2**.

HRMS:

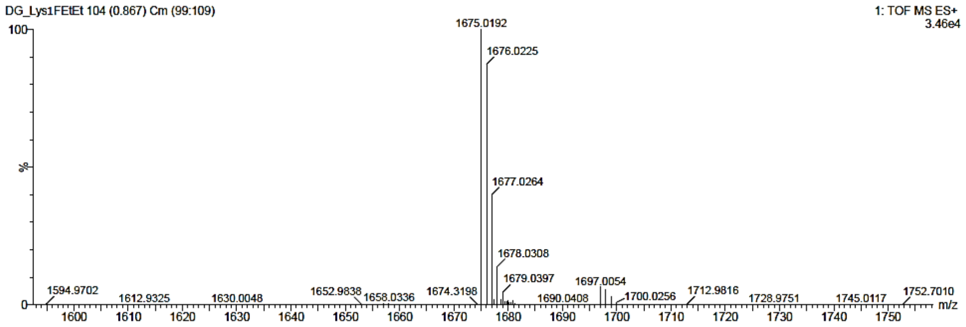

Figure S-125. Experimentally observed exact mass measured **Pep.2** .

Pep.3: [NLysN2fEtNSpe] [NLysN2fEtNEt]$_4$.

Analytical HPLC:

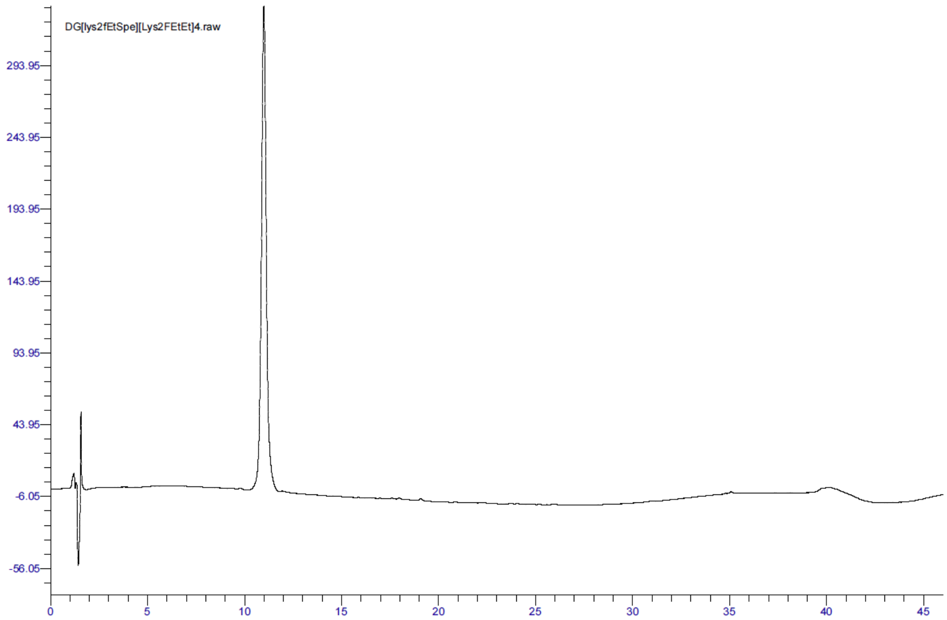

Figure S-126. Analytical HPLC trace of model peptoid **Pep.3** at λ=220nm.

Maldi-ToF:

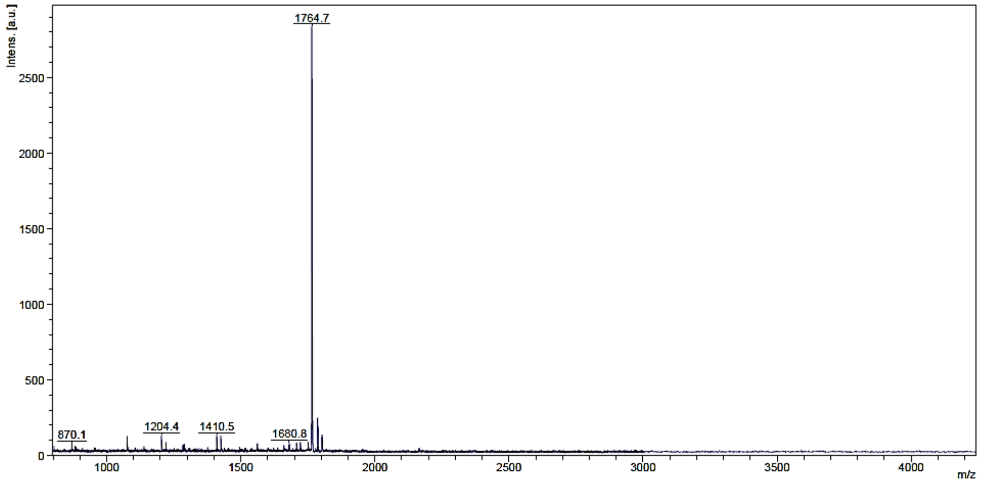

Figure S-127. Maldi-ToF spectra acquired for model peptoid **Pep.3**.

HRMS:

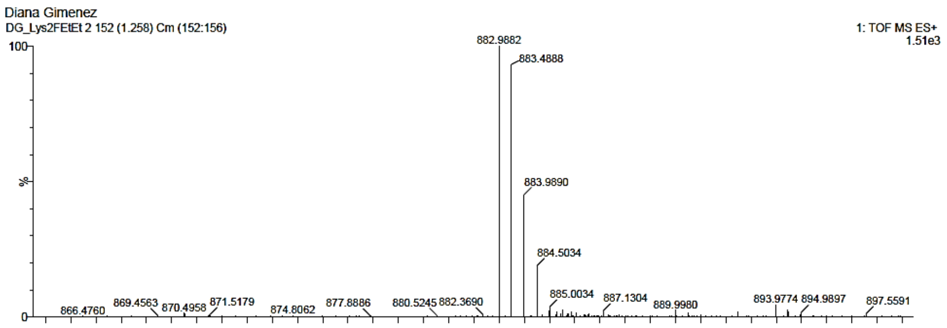

Figure S-128. HRMS (QToF-MS-ES+) trace of **Pep.3** .

Pep.4: [NLysN3fEtNSpe] [NLysN3fEtNEt]$_4$.

Analytical HPLC:

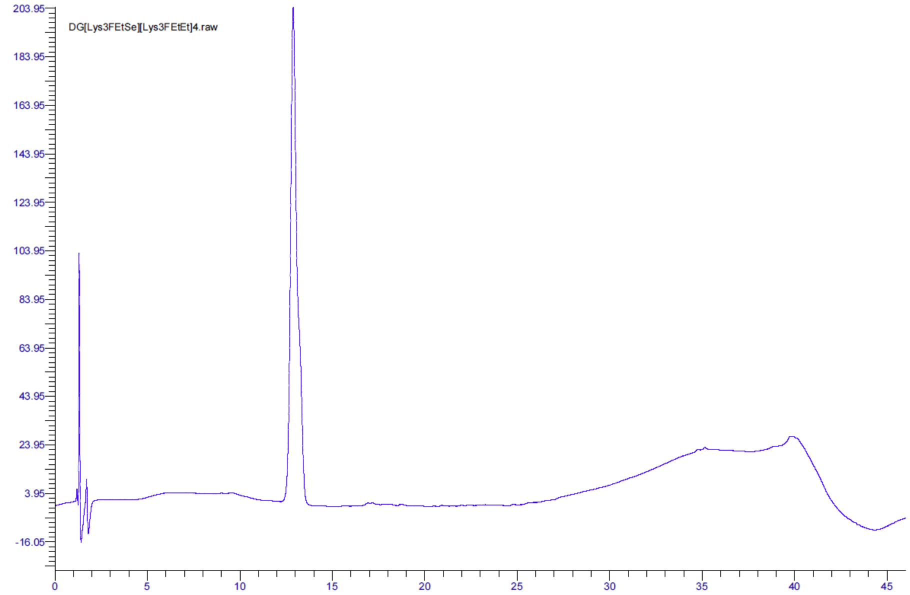

Figure S-129. Analytical HPLC trace of model peptoid **Pep.4** at λ=220nm.

Maldi-ToF:

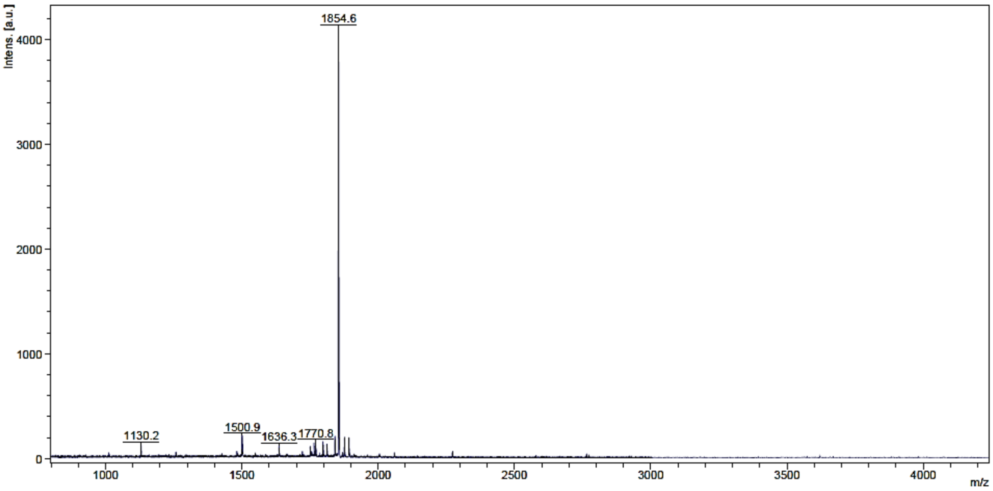

Figure S-130. Maldi-ToF spectra acquired for model peptoid **Pep.4**.

HRMS:

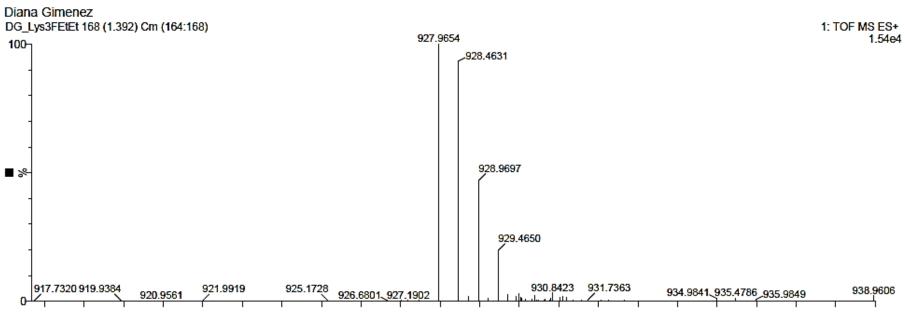

Figure S-131. HRMS (QToF-MS-ES+) trace of **Pep.4** .

Pep.5: [NLysN1fEtNSpe] [NLysN1fEtN1fEt]$_4$.

Analytical HPLC:

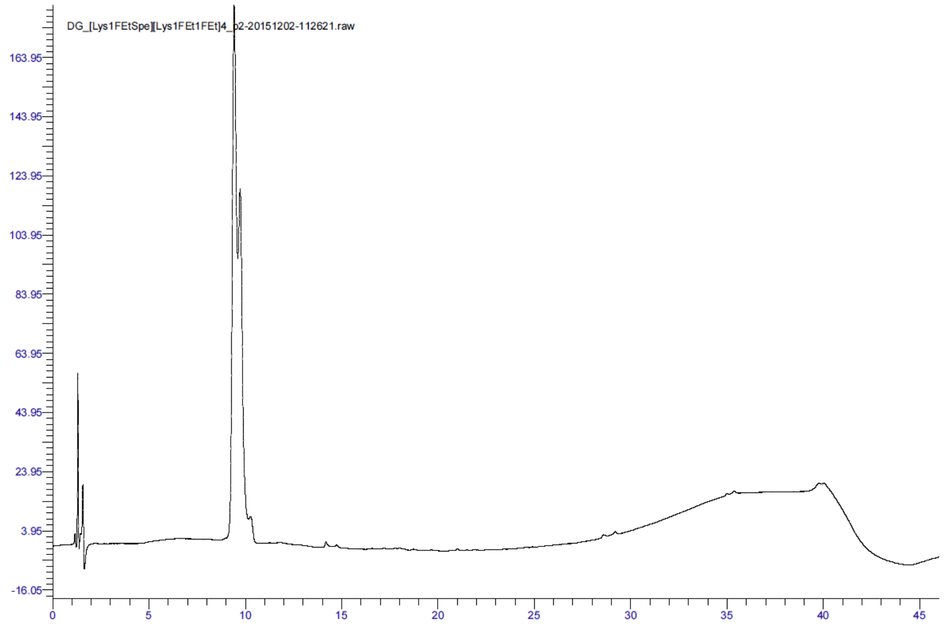

Figure S-132. Analytical HPLC trace of model peptoid **Pep.5** at λ=220nm.

Maldi-ToF:

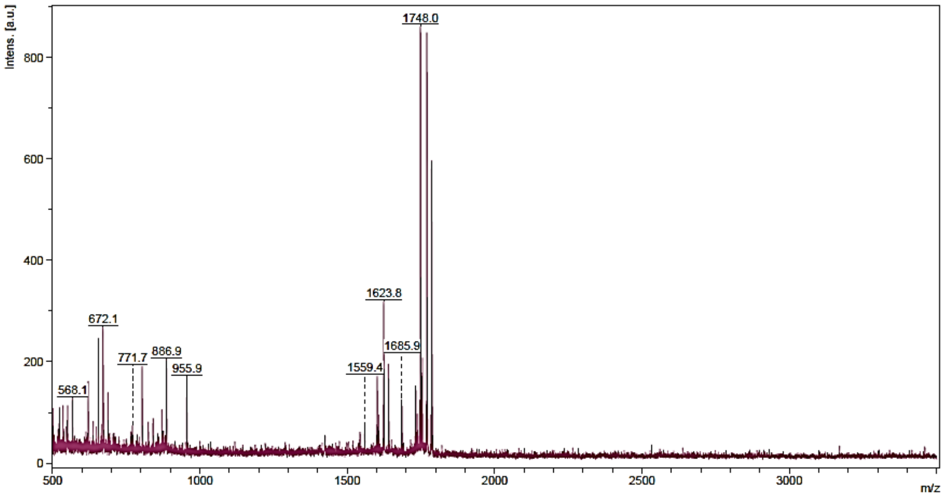

Figure S-133. Maldi-ToF spectra acquired for model peptoid **Pep.5**.

HRMS:

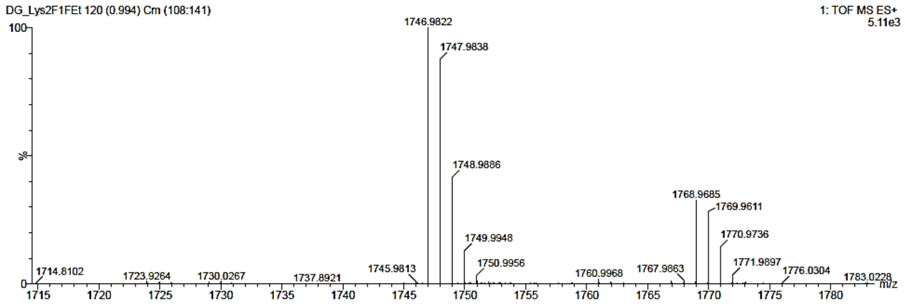

Figure S-134. HRMS (QToF-MS-ES+) trace of **Pep.5** .

Pep.6: [NLysN2fEtNSpe] [NLysN2fEtN2fEt]$_4$.

Analytical HPLC:

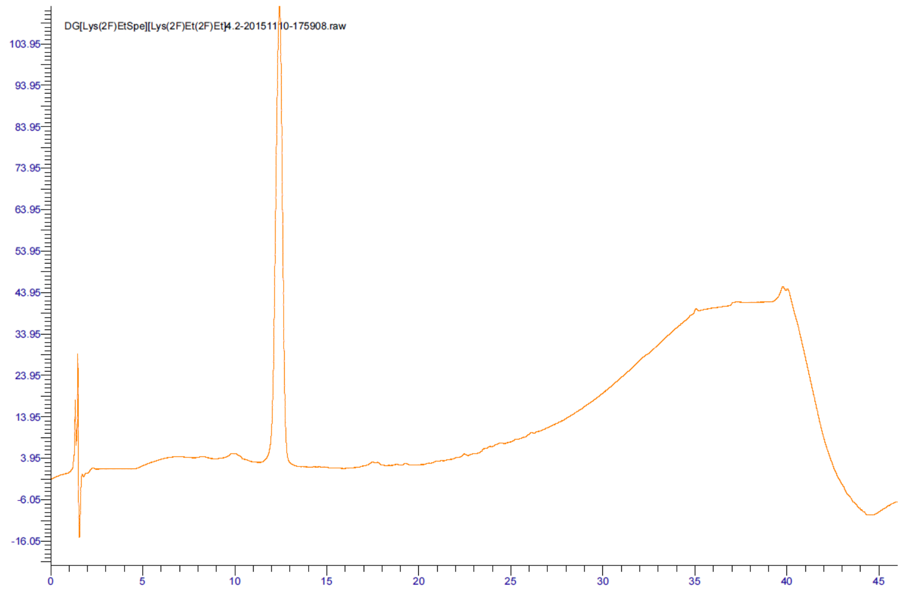

Figure S-135. Analytical HPLC trace of model peptoid **Pep.6** at λ=220nm.

Maldi-ToF:

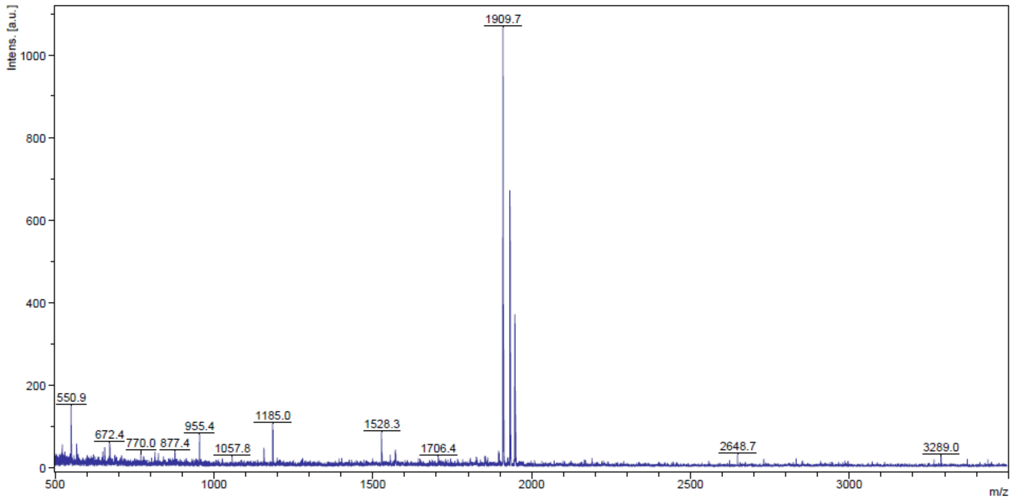

Figure S-136. Maldi-ToF spectra acquired for model peptoid **Pep.6**.

HRMS:

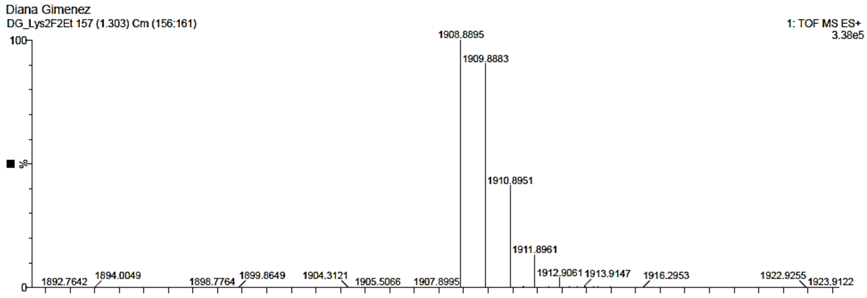

Figure S-137. HRMS (QToF-MS-ES+) trace of **Pep.6** .

Pep.7: [NLysN3fEtNSpe] [NLysN3fEtN3fEt]$_4$.

Analytical HPLC:

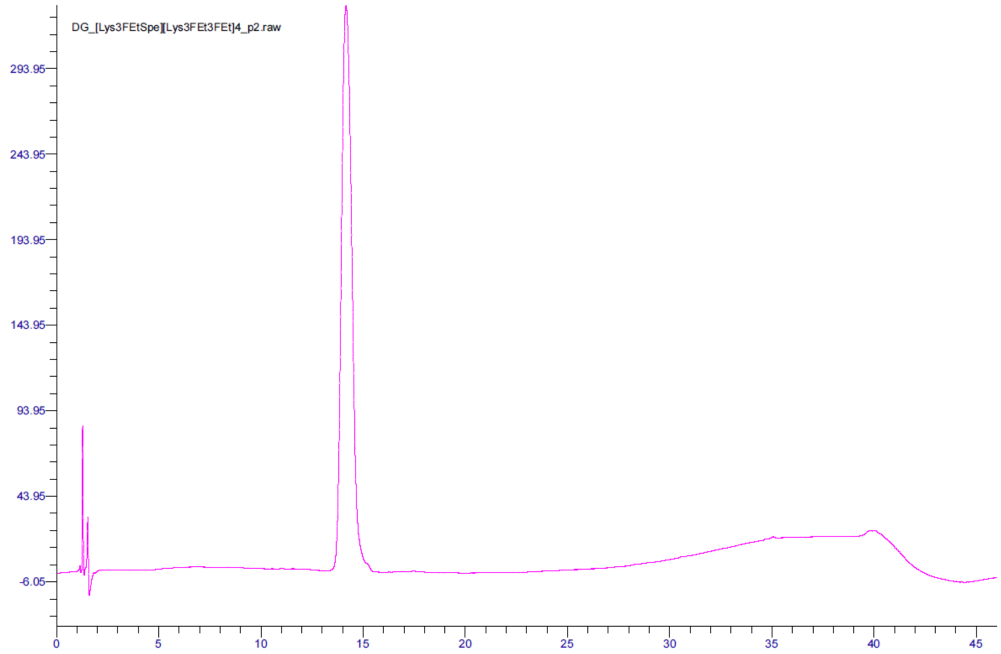

Figure S-138. Analytical HPLC trace of model peptoid **Pep.7** at λ=220nm.

Maldi-ToF:

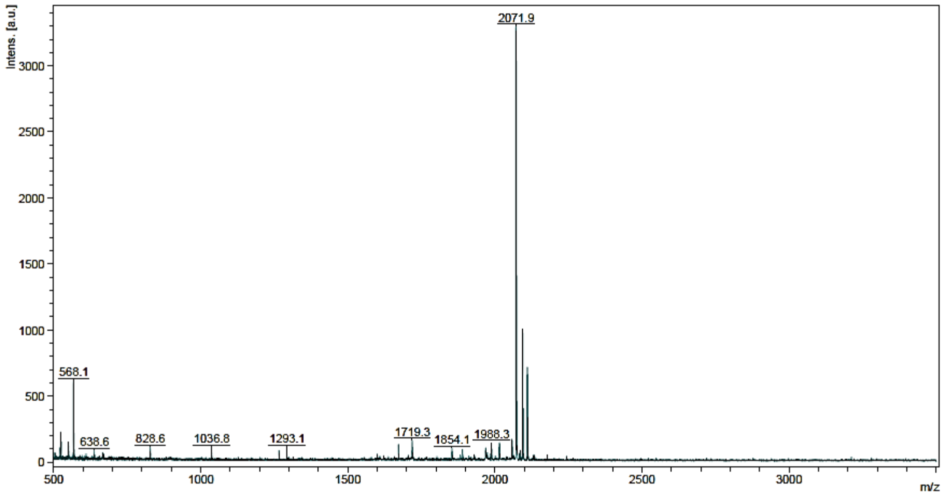

Figure S-139. Maldi-ToF spectra acquired for model peptoid **Pep.7**.

HRMS:

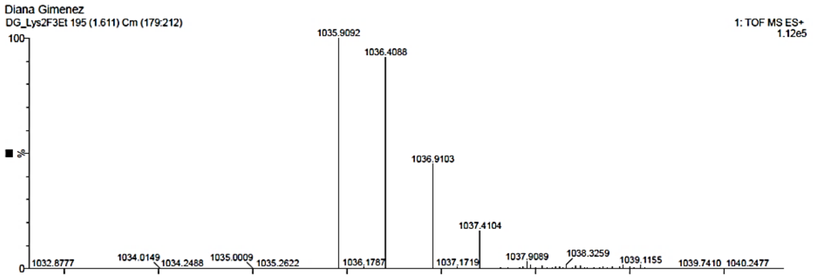

Figure S-140. HRMS (QToF-MS-ES+) trace of **Pep.7** .

CD spectra studies:

TFA content evaluation: Prior to CD spectra studies, 10mg of each peptoid were subject to 3 consecutive cycles of freeze-drying in deionized water over several days (50ml). Then the content of TFA present in each sample was assessed by ^{19}F-NMR (H_2O/MeOD 90:10). In fluorinated peptoids the signals arising from the side chains was employed as an internal standard of the number of florine present, giving a direct measure of the TFA/Peptoid ratio. In control peptoid **Pep.1**, 5 µl of a hexafluorobenze reference solution were spiked in the sample (10µl/ml, HFB), and the TFA content evaluated in its basis. In all cases a total of 6 TFA counterions per peptoid molecule was found, in agreement with the presence of 6 positive charges (Figure S-141).

General method for sample preparation and analysis: For CD spectra studies, a 2 mg/ml stock solution in water was made for every peptoid, and their individual concentrations corrected in the basis of the amount of trifluoroacetate present. Two exceptions were made to this general protocol: a more diluted 1mg/ml stock solution was prepared for the less soluble in water [NLys-N^{1f}Et-NSpe][NLys-N^{1f}Et -NEt]$_4$ peptoid and 1.5X more concentrated samples were measured for the negative control [NLys-NEt-NSpe][NLys-NEt-NEt]$_4$ due to the weak signal observed. To every raw recorded spectra the signal arising from the solvent was substracted and the resulting spectra in ellipticity units (mdeg) converted into the corresponding molar ellipticity $M_{\Theta,218}$ (deg.cm^2.dmol^{-1}). Finally, all curves were smoothed using Origin® Software (second order polynomial Savitzky-Golay filter). All numerical data is reported as the average value calculated from at least three different concentrations for each peptoid; corresponding standard deviations are given for a sampling number of n=3. Standard deviation for relative increases (i.e. $\Delta M_{\theta,218}/nf$) have been calculated according to the error propagation theory. All conditions where peptoid aggregation has been observed have been obviated in the analysis. For representation purposes through the paper, the average curve from the three measures has been employed. All main CD spectra parameters for peptoids **Pep.1-Pep.7** are summarized in following Table S-8.

Table S-8. Main CD spectra parameters of peptoid oligomers **Pep.1-Pep.7**.

Peptoid	[a] $M_{\theta,218}$ *10^{-3} (deg.cm^2.dmol^{-1})	[b] $\Delta M_{\theta,218}/n_f$ *10^{-3} (deg.cm^2.dmol^{-1})	[c] $M_{\theta,218,RES}$*10^{-3} (deg.cm^2.dmol^{-1}.residue^{-1})
**Pep.1**	-9.63±0.45	0.000	0.64±0.03
**Pep. 2**	-16.29±1.04	1.33±0.23	1.09±0.07
**Pep. 3**	-22.27±2.45	2.53±0.50	1.48±0.16
**Pep. 4**	-26.63±1.56	3.40±0.32	1.76±0.10
**Pep. 5**	-23.56±0.47	1.55±0.07	1.57±0.03
**Pep. 6**	-35.09±1.10	2.83±0.13	2.34±0.07
**Pep. 7**	-33.69±1.14	2.67±0.14	2.25±0.08

Average main CD spectra parameters as determined for peptoids **Pep.1-7** from three different concentrations in H_2O. [a] $M_{\theta,218}$: Molar ellipticity; [b] $M_{\theta,218}/nf$: Increase in molar ellipticity per fluorinated residue incorporated; [c] $M_{\theta,218,RES}$: per amide mean molar ellipticity. Corresponding standard deviation given in all cases in the basis of n=3. n_f = number of ethylamine residues replaced.

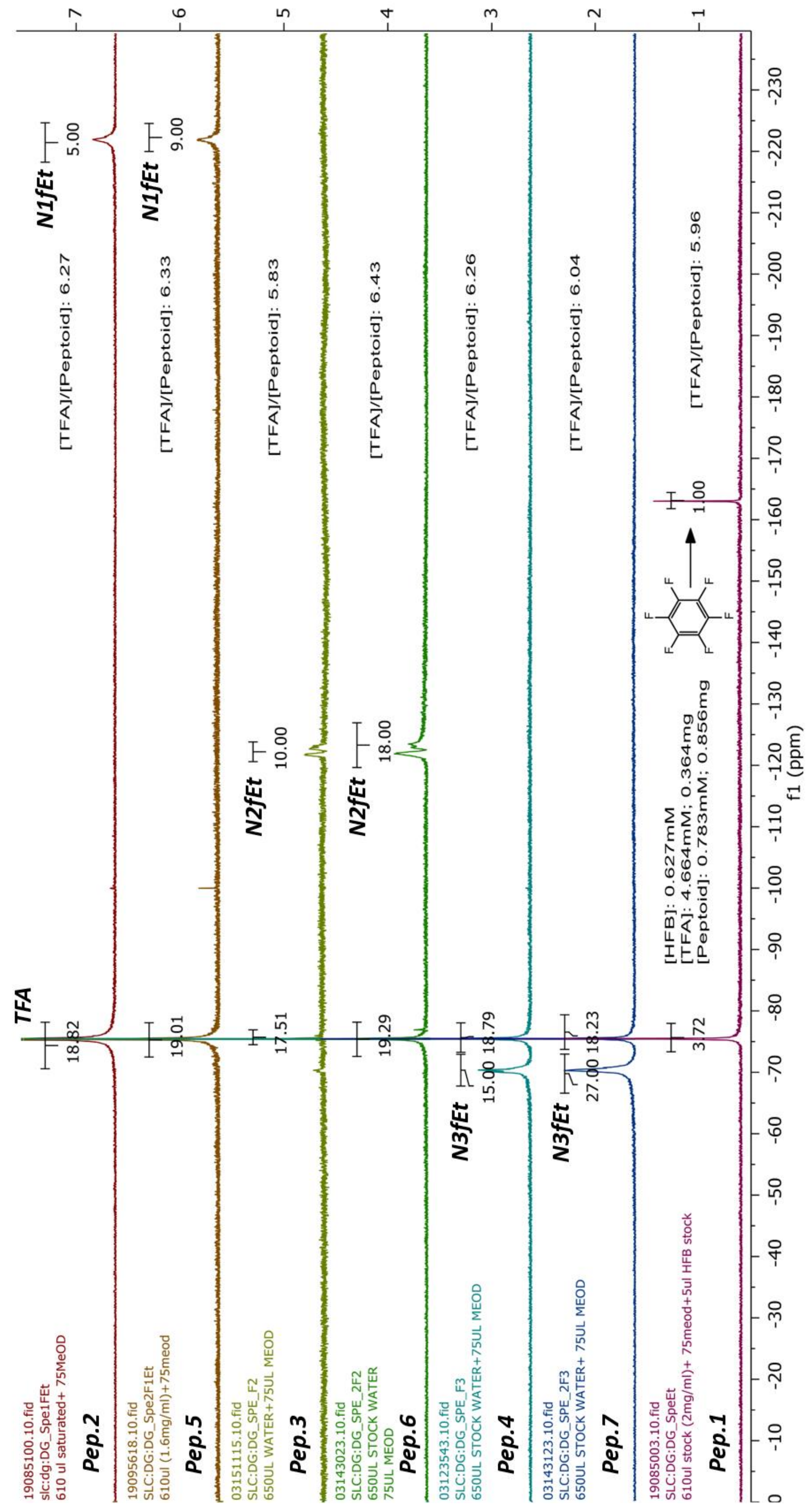

Figure S-141. ^{19}F-NMR analysis of the relative TFA content present in model peptoids **Pep.1-7**.

CD spectra analysis:

Pep.1: [NLysNEtNSpe] [NLysNEtNEt]$_4$.

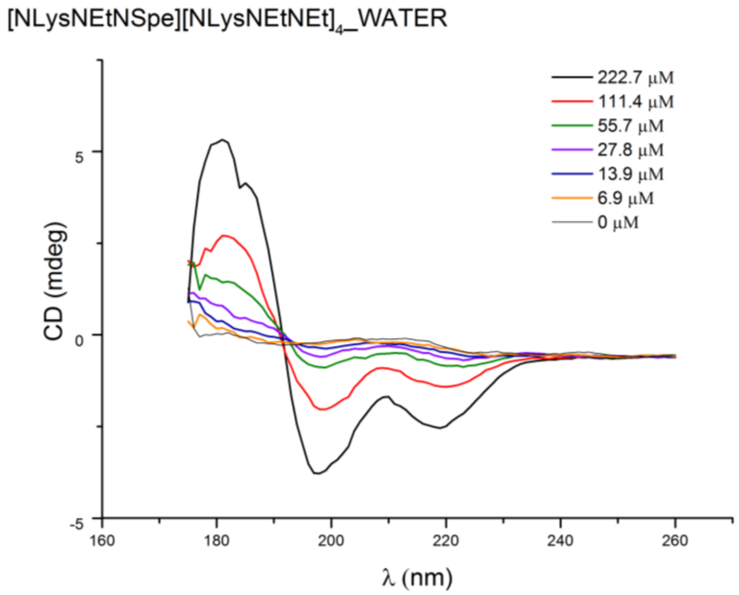

Figure S-142. Non-processed CD spectrums recorded in water by using increasing concentrations of model peptoid **Pep.1** at room temperature.

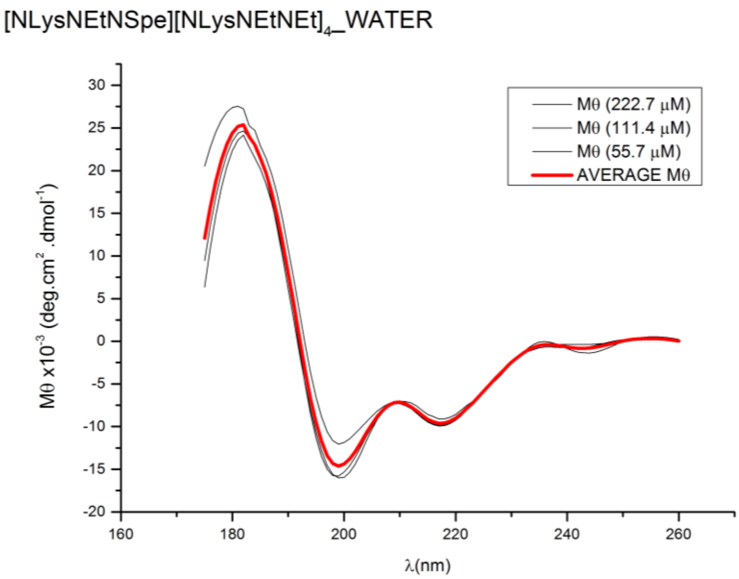

Figure S-143. Finally converted and smoothed M_Θ vs. λ spectrums at 3 different concentrations of model peptoid **Pep.1** selected for the estimation of the reported data in water (solid gray lines). Averaged curve employed for representation purposes in red.

Table S-9. Individual and averaged CD spectra parameters of **Pep.1** in H_2O.[a]

$M_{\theta,218\,(1)}$ *10^{-3} deg.cm^2.dmol^{-1}	$M_{\theta,220\,(1)}$ *10^{-3} deg.cm^2.dmol^{-1}	$M_{\theta,220\,(1)}$ *10^{-3} deg.cm^2.dmol^{-1}	$M_{\theta,220\,(1)}$ *10^{-3} deg.cm^2.dmol^{-1}	$M_{\theta,220\,(1)}$ *10^{-3} deg.cm^2.dmol^{-1}	$M_{\theta,220\,(1)}$ *10^{-3} deg.cm^2.dmol^{-1}	$^{AVG}M_{\theta,218}$ *10^{-3} deg.cm^2.dmol^{-1}	± SD *10^{-3} deg.cm^2.dmol^{-1}
-9.865	-15.792	-9.923	-16.015	-9.114	-12.049	-9.63	0.45

[a] M_θ = Mean molar ellipticity at the given wavelegnt.

Pep.2: [NLysN^{1f}EtNSpe] [NLysN^{1f} EtNEt]$_4$.

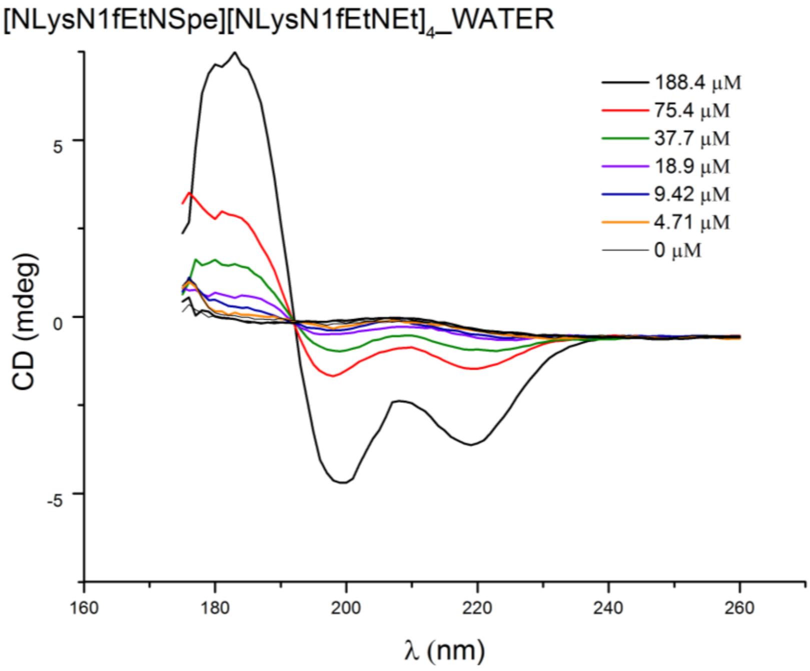

Figure S-144. Non-processed CD spectrums recorded in water by using increasing concentrations of model peptoid **Pep.2** at room temperature

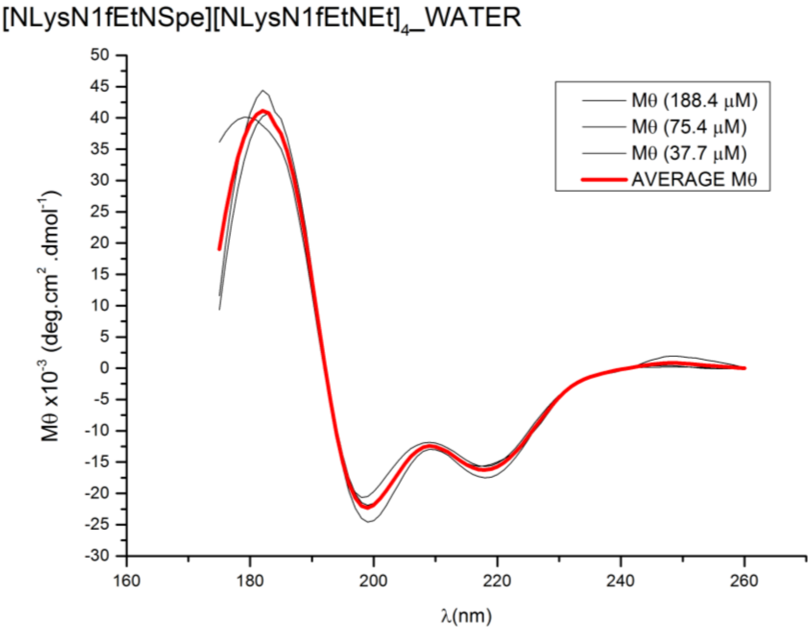

Figure S-145. Finally converted and smoothed M_{Θ} vs. λ spectrums at 3 different concentrations of model peptoid **Pep.2** selected for the estimation of the reported data in water (solid gray lines). Averaged curve employed for representation purposes in red.

Table S-10. Individual and averaged CD spectra parameters of **Pep.2** in H_2O.[a]

$M_{\theta,218}$ (1) *10^{-3} deg.cm^2.dmol^{-1}	$M_{\theta,220}$ (1) *10^{-3} deg.cm^2.dmol^{-1}	$M_{\theta,220}$ (1) *10^{-3} deg.cm^2.dmol^{-1}	$M_{\theta,220}$ (1) *10^{-3} deg.cm^2.dmol^{-1}	$M_{\theta,220}$ (1) *10^{-3} deg.cm^2.dmol^{-1}	$M_{\theta,220}$ (1) *10^{-3} deg.cm^2.dmol^{-1}	$^{AVG}M_{\theta,218}$ *10^{-3} deg.cm^2.dmol^{-1}	± SD *10^{-3} deg.cm^2.dmol^{-1}
-17.490	-24.557	-15.688	-20.630	-15.689	-21.890	-16.29	1.04

[a] M_{θ} = Mean molar ellipticity at the given wavelegnt.

Pep.3: [NLysN^{2f} EtNSpe] [NLysN^{2f} EtNEt]$_4$.

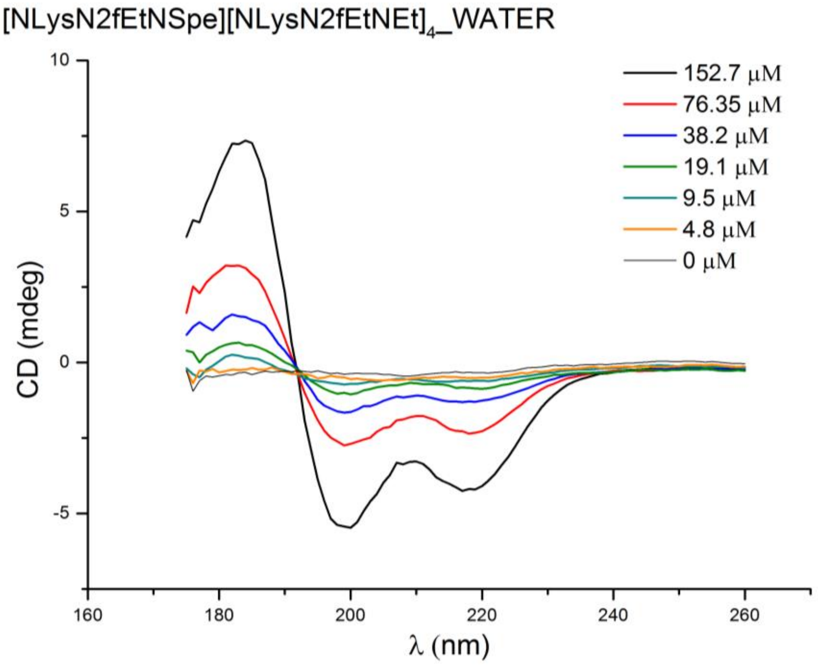

Figure S-146. Non-processed CD spectrums recorded in water by using increasing concentrations of model peptoid **Pep.3** at room temperature

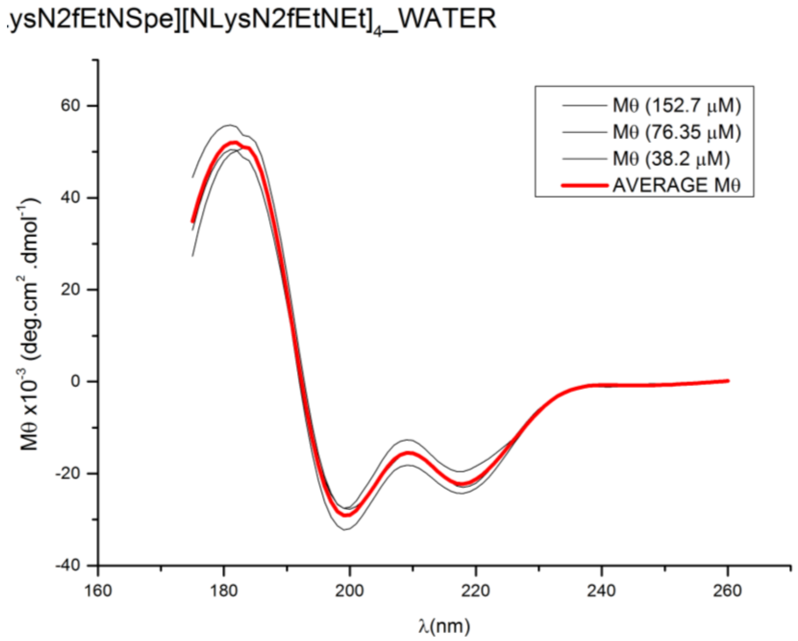

Figure S-147. Finally converted and smoothed M_{Θ} vs. λ spectrums at 3 different concentrations of model peptoid **Pep.3** selected for the estimation of the reported data in water (solid gray lines). Averaged curve employed for representation purposes in red.

Table S-11. Individual and averaged CD spectra parameters of **Pep.3** in H_2O.[a]

$M_{\theta,218\,(1)}$ *10^{-3} deg.cm^2.dmol^{-1}	$M_{\theta,220\,(1)}$ *10^{-3} deg.cm^2.dmol^{-1}	$M_{\theta,220\,(1)}$ *10^{-3} deg.cm^2.dmol^{-1}	$M_{\theta,220\,(1)}$ *10^{-3} deg.cm^2.dmol^{-1}	$M_{\theta,220\,(1)}$ *10^{-3} deg.cm^2.dmol^{-1}	$M_{\theta,220\,(1)}$ *10^{-3} deg.cm^2.dmol^{-1}	$^{AVG}M_{\theta,218}$ *10^{-3} deg.cm^2.dmol^{-1}	± SD *10^{-3} deg.cm^2.dmol^{-1}
-24.289	-32.206	-22.973	-27.732	-19.546	-27.527	-22.27	2.45

[a] M_{θ} = Mean molar ellipticity at the given wavelegnt.

Pep.4: [NLysN^{3f}EtNSpe] [NLysN^{3f}EtNEt]$_4$.

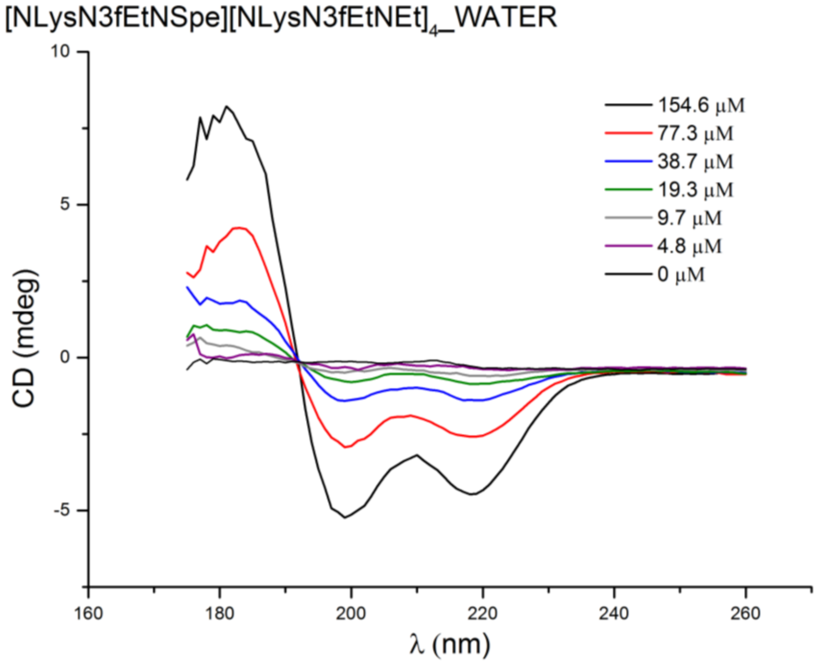

Figure S-148. Non-processed CD spectrums recorded in water by using increasing concentrations of model peptoid **Pep.4** at room temperature.

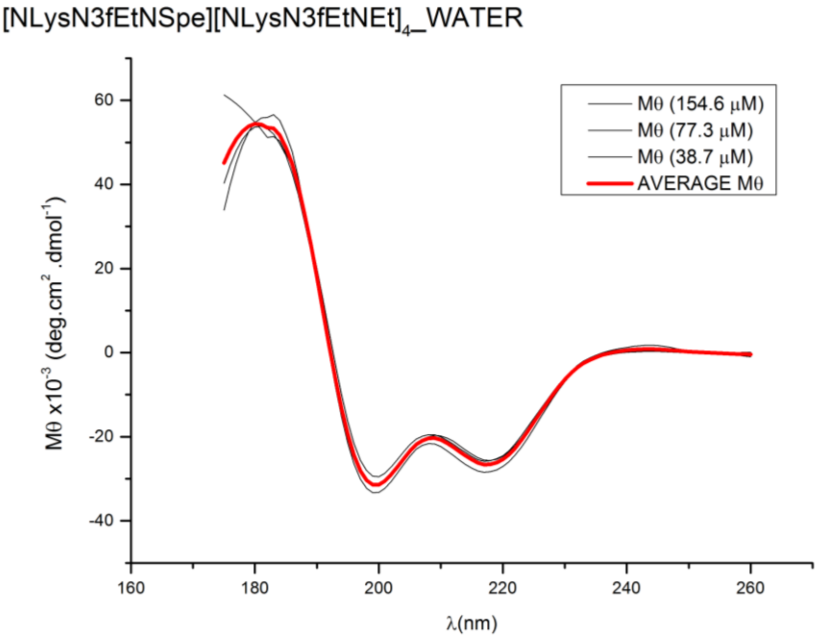

Figure S-149. Finally converted and smoothed M_Θ vs. λ spectrums at 3 different concentrations of model peptoid **Pep.4** selected for the estimation of the reported data in water (solid gray lines). Averaged curve employed for representation purposes in red.

Table S-12. Individual and averaged CD spectra parameters of **Pep.4** in H_2O.[a]

$M_{\theta,218\,(1)}$ *10^{-3} deg.cm^2.dmol^{-1}	$M_{\theta,220\,(1)}$ *10^{-3} deg.cm^2.dmol^{-1}	$M_{\theta,220\,(1)}$ *10^{-3} deg.cm^2.dmol^{-1}	$M_{\theta,220\,(1)}$ *10^{-3} deg.cm^2.dmol^{-1}	$M_{\theta,220\,(1)}$ *10^{-3} deg.cm^2.dmol^{-1}	$M_{\theta,220\,(1)}$ *10^{-3} deg.cm^2.dmol^{-1}	$^{AVG}M_{\theta,218}$ *10^{-3} deg.cm^2.dmol^{-1}	± SD *10^{-3} deg.cm^2.dmol^{-1}
-25.645	-31.623	-28.424	-33.273	-25.824	-29.487	-26.63	1.56

[a] M_θ = Mean molar ellipticity at the given wavelegnt.

Pep.5: [NLysN1fEtNSpe] [NLysN1fEtN1fEt]$_4$.

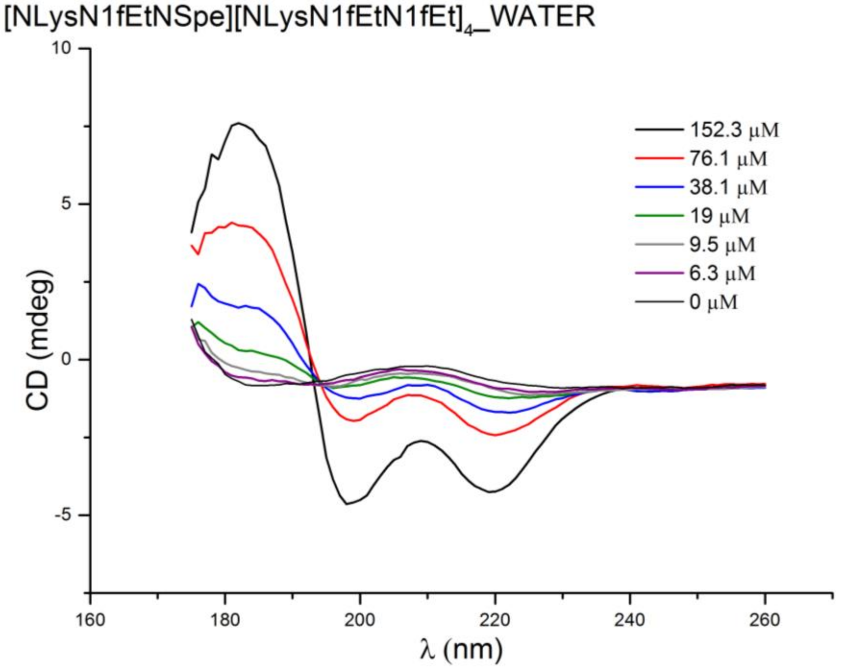

Figure S-150. Non-processed CD spectrums recorded in water by using increasing concentrations of model peptoid **Pep.5** at room temperature

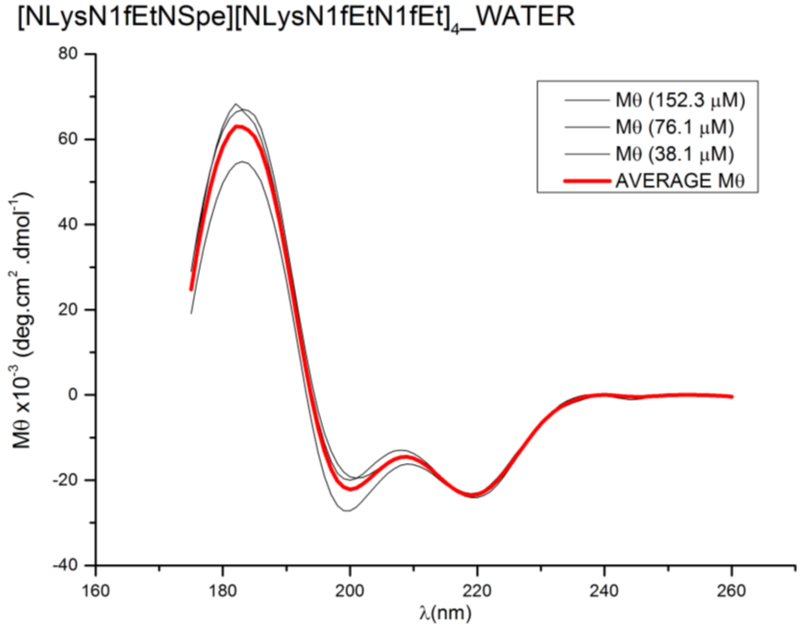

Figure S-151. Finally converted and smoothed M_Θ vs. λ spectrums at 3 different concentrations of model peptoid **Pep.5** selected for the estimation of the reported data in water (solid gray lines). Averaged curve employed for representation purposes in red.

Table S-13. Individual and averaged CD spectra parameters of **Pep.5** in H_2O.[a]

$M_{\theta,218}$ (1) *10^{-3} deg.cm^2.dmol^{-1}	$M_{\theta,220}$ (1) *10^{-3} deg.cm^2.dmol^{-1}	$M_{\theta,220}$ (1) *10^{-3} deg.cm^2.dmol^{-1}	$M_{\theta,220}$ (1) *10^{-3} deg.cm^2.dmol^{-1}	$M_{\theta,220}$ (1) *10^{-3} deg.cm^2.dmol^{-1}	$M_{\theta,220}$ (1) *10^{-3} deg.cm^2.dmol^{-1}	$^{AVG}M_{\theta,218}$ *10^{-3} deg.cm^2.dmol^{-1}	± SD *10^{-3} deg.cm^2.dmol^{-1}
-23.434	-27.163	-23.166	-20.019	-24.078	-19.536	-23.56	0.47

[a] M_θ = Mean molar ellipticity at the given wavelegnt.

Pep.6: [NLysN^{2f}EtNSpe] [NLysN^{2f}EtN^{2f}Et]$_4$.

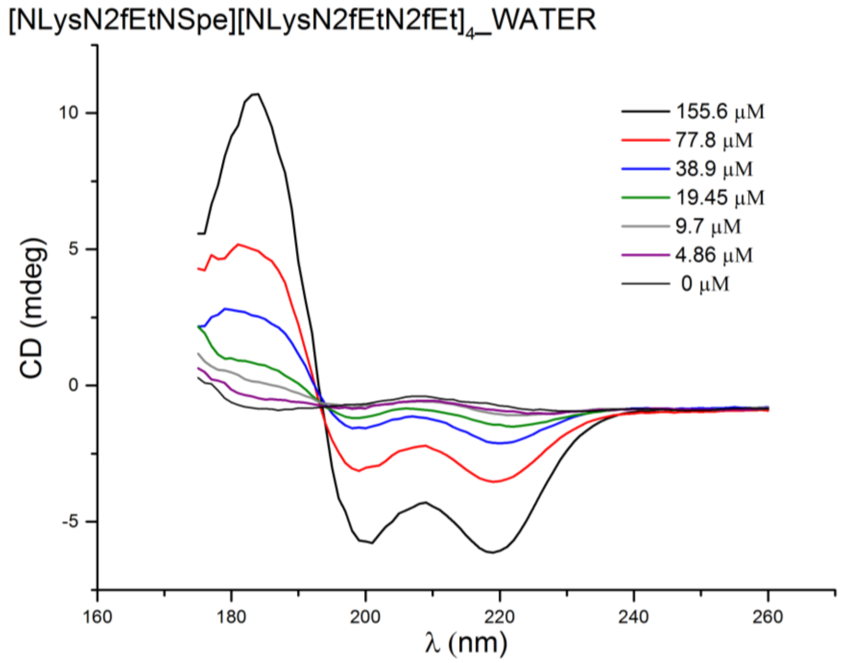

Figure S-152. Non-processed CD spectrums recorded in water by using increasing concentrations of model peptoid **Pep.6** at room temperature
.

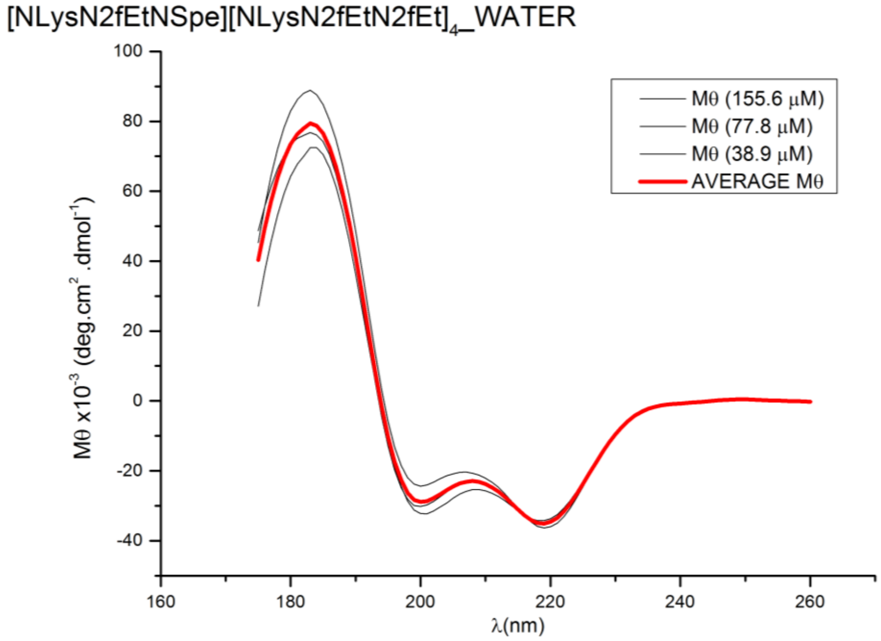

Figure S-153. Finally converted and smoothed M_Θ vs. λ spectrums at 3 different concentrations of model peptoid **Pep.6** selected for the estimation of the reported data in water (solid gray lines). Averaged curve employed for representation purposes in red.

Table S-14. Individual and averaged CD spectra parameters of **Pep.6** in H_2O.[a]

$M_{\theta,218\,(1)}$ $*10^{-3}$ deg.cm^2.dmol^{-1}	$M_{\theta,220\,(1)}$ $*10^{-3}$ deg.cm^2.dmol^{-1}	$M_{\theta,220\,(1)}$ $*10^{-3}$ deg.cm^2.dmol^{-1}	$M_{\theta,220\,(1)}$ $*10^{-3}$ deg.cm^2.dmol^{-1}	$M_{\theta,220\,(1)}$ $*10^{-3}$ deg.cm^2.dmol^{-1}	$M_{\theta,220\,(1)}$ $*10^{-3}$ deg.cm^2.dmol^{-1}	$^{AVG}M_{\theta,218}$ $*10^{-3}$ deg.cm^2.dmol^{-1}	± SD $*10^{-3}$ deg.cm^2.dmol^{-1}
-34.233	-32.252	-34.707	-30.189	-36.339	-24.355	-35.09	1.10

[a] M_θ = Mean molar ellipticity at the given wavelegnt.

Pep.7: [NLysN^{3f}EtNSpe] [NLysN^{3f}EtN^{3f}Et]$_4$.

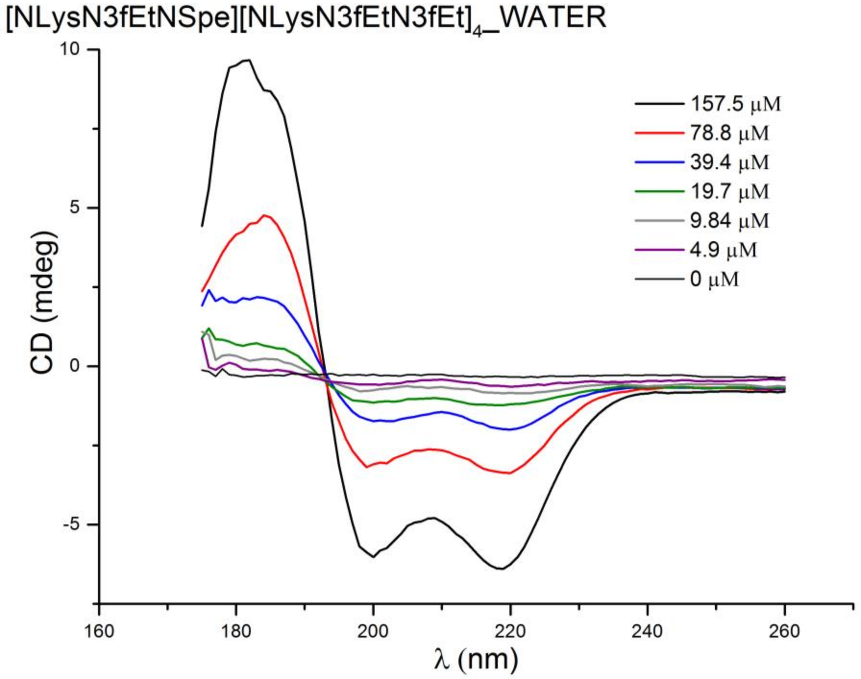

Figure S-154. Non-processed CD spectrums recorded in water by using increasing concentrations of model peptoid **Pep.7** at room temperature.

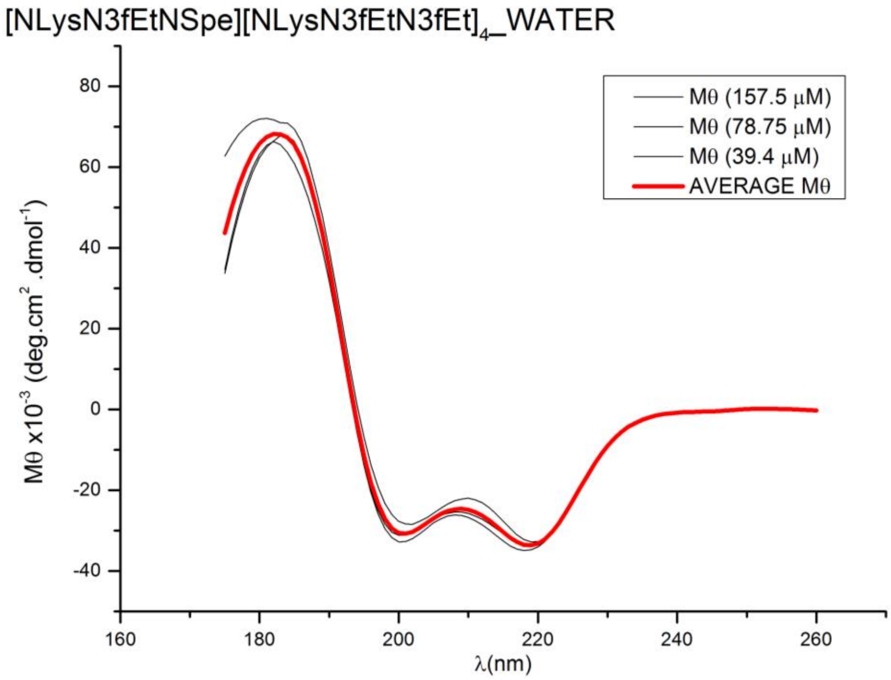

Figure S-155. Finally converted and smoothed M_Θ vs. λ spectrums at 3 different concentrations of model peptoid **Pep.7** selected for the estimation of the reported data in water (solid gray lines). Averaged curve employed for representation purposes in red.

Table S-15. Individual and averaged CD spectra parameters of **Pep.7** in H_2O.[a]

$M_{\theta,218}$ (1) *10^{-3} deg.cm^2.dmol^{-1}	$M_{\theta,220}$ (1) *10^{-3} deg.cm^2.dmol^{-1}	$M_{\theta,220}$ (1) *10^{-3} deg.cm^2.dmol^{-1}	$M_{\theta,220}$ (1) *10^{-3} deg.cm^2.dmol^{-1}	$M_{\theta,220}$ (1) *10^{-3} deg.cm^2.dmol^{-1}	$M_{\theta,220}$ (1) *10^{-3} deg.cm^2.dmol^{-1}	$^{AVG}M_{\theta,218}$ *10^{-3} deg.cm^2.dmol^{-1}	± SD *10^{-3} deg.cm^2.dmol^{-1}
-33.330	-31.200	-32.774	-28.424	-34.975	-32.814	-33.69	1.14

[a] M_θ = Mean molar ellipticity at the given wavelegnt.

References

[1] O. V. Dolomanov, L. J. Bourhis, R. J. Gildea, J. a. K. Howard, H. Puschmann, *J. Appl. Crystallogr.* **2009**, *42*, 339–341.
[2] G. M. Sheldrick, *Acta Crystallogr. A* **2008**, *64*, 112–122.
[3] L. Hunter, A. M. Z. Slawin, P. Kirsch, D. O'Hagan, *Angew. Chem. Int. Ed.* **2007**, *46*, 7887–7890.
[4] L. Hunter, P. Kirsch, J. T. G. Hamilton, D. O'Hagan, *Org. Biomol. Chem.* **2008**, *6*, 3105.
[5] A. M. Ihrig, S. L. Smith, *J. Am. Chem. Soc.* **1972**, *94*, 34–41.
[6] D. O'Hagan, H. S. Rzepa, M. Schüler, A. M. Slawin, *Beilstein J. Org. Chem.* **2006**, *2*, 19.
[7] J. A. Aguilar, G. A. Morris, A. M. Kenwright, *RSC Adv.* **2014**, *4*, 8278.
[8] M. Foroozandeh, R. W. Adams, N. J. Meharry, D. Jeannerat, M. Nilsson, G. A. Morris, *Angew. Chem. Int. Ed.* **2014**, *53*, 6990–6992.
